# A novel class of C14-sulfonate-tetrandrine derivatives as potential chemotherapeutic agents for hepatocellular carcinoma

**DOI:** 10.3389/fchem.2022.1107824

**Published:** 2023-01-10

**Authors:** Taibai Jiang, Guangtong Xie, Zhirui Zeng, Junjie Lan, Hanfei Liu, Jinyu Li, Hai Ren, Tengxiang Chen, Weidong Pan

**Affiliations:** ^1^ School of Basic Medicine/State Key Laboratory of Functions and Applications of Medicinal Plants, Key Laboratory of Chemistry for Natural Products of Guizhou Province and Chinese Academy of Sciences, Guizhou Medical University, Guiyang, China; ^2^ School of Pharmacy, Guizhou University of Traditional Chinese Medicine, Guiyang, China; ^3^ Guizhou Provincial Key Laboratory of Pathogenesis and Drug Research on Common Chronic Diseases, Department of Physiology, School of Basic Medical Sciences, Guizhou Medical University, Guiyang, China; ^4^ Department of Pharmacy, Guizhou Provincial People’s Hospital, Guiyang, China; ^5^ Precision Medicine Research Institute of Guizhou, Affiliated Hospital of Guizhou Medical University, Guiyang, China; ^6^ School of Pharmaceutical Sciences, Guizhou University, Guiyang, China

**Keywords:** tetrandrine derivatives, sulfonate derivatives, anti-migration, apoptosis, anti-HCC

## Abstract

Hepatocellular carcinoma (HCC), the most common malignancy of the liver, exhibits high recurrence and metastasis. Structural modifications of natural products are crucial resources of antitumor drugs. This study aimed to synthesize C-14 derivatives of tetrandrine and evaluate their effects on HCC. Forty C-14 sulfonate tetrandrine derivatives were synthesized and their *in vitro* antiproliferative was evaluated against four hepatoma (HepG-2, SMMC-7721, QGY-7701, and SK-Hep-1) cell lines. For all tested cells, most of the modified compounds were more active than the lead compound, tetrandrine. In particular, 14-O-(5-chlorothiophene-2-sulfonyl)-tetrandrine (**33**) exhibited the strongest antiproliferative effect, with half-maximal inhibitory concentration values of **1.65**, **2.89**, **1.77**, and **2.41** μM for the four hepatoma cell lines, respectively. Moreover, **33** was found to induce apoptosis *via* a mitochondria-mediated intrinsic pathway *via* flow cytometry and western blotting analysis. In addition, colony formation, wound healing, and transwell assays demonstrated that **33** significantly inhibited HepG-2 and SMMC-7721 cell proliferation, migration, and invasion, indicating that it might potentially be a candidate for an anti-HCC therapy in the future.

## 1 Introduction

Liver cancer exhibits the second highest mortality rate among all cancers worldwide (Sung et al., 2021; Siegel et al., 2022). Hepatocellular carcinoma (HCC) accounts for >90% of all liver cancer cases. According to the treatment guidelines, liver resection and transplantation are recommended for early stage, and chemotherapy and radiofrequency ablation are recommended for advanced stage patients with HCC (Liu C. et al., 2015; Dimitroulis et al., 2017). Currently, the commonly used liver cancer treatment drugs in clinical practice include chemotherapy drugs (5-fluorouracil, oxaliplatin, and doxorubicin) and targeted drugs (sorafenib and lenvatinib) (Kim et al., 2017). Despite remarkable progress in the treatment of HCC with chemotherapy and targeted therapy, numerous patients experience relapse, toxic side effects, and drug resistance (Anwanwan et al., 2020). In addition, high cost of targeted drugs imposes a heavy burden on patients with HCC. Therefore, it is necessary to develop new, effective, and inexpensive anti-HCC drugs. Natural small-molecular compounds are characterized by their structural and functional diversities. They are important sources for new drug development (Rishton, 2008). A significant proportion of commonly used clinical drugs is derived directly or indirectly from natural compounds. Drugs derived from natural products and their derivatives have accounted for 10% and 23% of the total global small-molecule antitumor drugs, respectively, in the past 30 years (Newman and Cragg, 2020). Natural products remain one of the most promising avenues for future antitumor drug development.

Tetrandrine, isolated and identified from Stephania tetrandra Moore in the 1930s, is an alkaloid consisting of two benzylisoquinoline fragments coupled by two ether bonds to form an irregular eighteen-membered ring (Bhagya and Chandrashekar, 2016). It is a potential bioactive molecule with anticancer (Bhagya and Chandrashekar, 2022), anti-inflammatory (Gao et al., 2016), antioxidant (Shi et al., 1995), antibacterial (Yi et al., 2022), and other pharmacological properties (Heister and Poston, 2020). Studies have shown that tetrandrine can induce tumor cell apoptosis for its antitumor effects, including reactive oxygen species activation (Lin et al., 2016), inhibition of the AKT/forkhead box O3a signaling pathway (Zhang et al., 2015) and signal transducer and activator of transcription three phosphorylation (Ma et al., 2015). Owing to its excellent antitumor activities, many researchers have focused their efforts on the structural modifications of tetrandrine to discover new antitumor drug candidates. The structural modification of tetrandrine is mainly focused on two sites, C-5 and C-14, and the antitumor activity of some derivatives can be enhanced by introducing halogens and alkyl groups at these positions (Wu et al., 2013; Wei et al., 2016). The same effect can be achieved by converting tertiary amine nitrogen to quaternary amine nitrogen to form quaternary salts at these two sites (Li et al., 2017).

Previously, our group synthesized a series of C-14 tetrandrine derivatives with substituents, including urea, thiourea, and amide fragments. *In vitro* screening for antitumor activity revealed that these compounds showed favorable inhibitory effects on various tumor cell lines, including HCC (MHCC97L and PLC/PRF/5) (Lan et al., 2017), leukemia (HEL and K562), breast cancer (MDA-MB-231), prostate cancer (PC3), and melanoma (WM9) (Lan et al., 2018; Song et al., 2018) cell lines. However, all the active compounds we synthesized were structurally derived from C14-amino-tetrandrine. Despite their potential as anticancer agents, these derivatives had low bioavailability and poor water solubility, which limited their further development. Therefore, we hope to change these adverse situations by modifying the C-14 substitutions. Sulfonyl groups are generally known for their stable structure and high polarity. The introduction of sulfonyl groups could reduce the *pKa* value and increase the bioavailability of the target derivatives while changing their biological activities (Jamieson et al., 2006). In addition, sulfonyl groups can provide two hydrogen bond acceptors, which is beneficial for enhancing ligand-protein interaction. Recently, Gao et al. reported that C7-O-sulfonate-d-tetrandrine derivatives showed potential anticancer properties in human lung cancer cells *in vitro* (Gao et al., 2021).

Encouraged by the findings of our earlier research and relevant literature, we kept working to modify the C-14 position of tetrandrine to develop novel anti-HCC drug candidates. In this study, we designed and synthesized a series of sulfonate-substituted tetrandrine derivatives and evaluated their proliferative inhibitory effects on HepG-2, SMMC-7721, QGY-7701, and SK-Hep-1 HCC cell lines. Derivative **33** showed a significantly higher anti-HCC activity compared to tetrandrine. We also performed a preliminary study to investigate the probable anticancer mechanism of **33**.

## 2 Materials and methods

### 2.1 Chemistry

#### 2.1.1 General information

Tetrandrine (purity ≥98%) was purchased from Nanjing Jingzhu Biotechnology Co., Ltd. (Nanjing, China). All reagents and solvents were sourced from commercial companies, Adamas (Shanghai, China) and Energy & Chemical (Shanghai, China), and used without additional purification unless otherwise stated. Anhydrous dichloromethane (DCM) was dried over calcium hydride prior to distillation under an argon atmosphere. Column chromatograph was filled with 300–400 mesh silica gel (Qingdao Marine Chemical Co., Ltd, Qingdao, China) and eluted using a mixture of dichloromethane, methanol, and diethylamine. Thin layer chromatography (TLC; 0.25 mm; GF254; Qingdao Marine Chemical Co., Ltd, Qingdao, China) was used to monitor the reactions, and UV light (254 nm) or 10% phosphomolybdic acid/ethanol was used for visualization. Bruker AVANCE III 600 MHz spectrometers (Bruker Biospin, Billerica, United States) were used to collect the nuclear magnetic resonance (NMR) spectra (^1^H, ^13^C, and ^19^F). High-resolution mass spectra were obtained using Thermo Scientific Q Exactive Focus (Thermo Scientific Q Exactive Focus). Melting points were determined using a WRX-4 micro melting point instrument (Tansoole, Shanghai, China).

### 2.2 Methods of synthesis

#### 2.2.1 Synthesis procedure for the preparation of C14-nitro-tetrandrine (M1)

Concentrated nitric acid (68%, 7 ml, 56 mmol) was added dropwise to acetic anhydride (10 ml, 105.6 mmol) at 0°C to obtain a mixed acid. Then, the mixed acid (15 ml) was slowly added to a solution of tetrandrine (5 g, 8.03 mmol) in DCM (25 ml) at 0°C. The reaction was monitored *via* TLC every 10 min until completion. The reaction was quenched with saturated aqueous sodium bicarbonate and extracted with DCM (3 ml × 300 ml). Anhydrous sodium sulfate was used to dry the combined organic fractions, which was followed by filtration and vacuum concentration. C14-nitro-tetrandrine was obtained *via* silica gel column chromatography using DCM/MeOH (50/1 v/v, 0.5% DEA). Light yellow amorphous solid (95% yield, purity >95%, HPLC). Mp: 176.2°C. ^1^H-NMR (600 MHz, CDCl_3_) δ: 7.41 (s, 1H), 7.37 (dd, *J* = 8.4, 2.4 Hz, 1H), 7.12 (dd, *J* = 8.4, 2.4 Hz, 1H), 6.77 (dd, *J* = 8.4, 2.4 Hz, 1H), 6.54 (s, 1H), 6.52 (s, 1H), 6.30–6.28 (2H, m), 5.98 (s, 1H), 3.98 (s, 3H), 3.90 (dd, *J* = 10.8, 6.0 Hz, 1H), 3.75 (s, 3H), 3.66 (dd, *J* = 13.8, 10.8 Hz, 1H), 3.50 (d, *J* = 10.8 Hz, 1H), 3.45–3.40 (m, 1H), 3.37 (s, 3H), 3.30–3.23 (m, 2H), 3.18 (s, 3H), 2.98–2.92 (m, 1H), 2.90–2.82 (m, 3H), 2.76–2.68 (m, 2H),2.63 (s, 3H), 2.54 (dd, *J* = 13.2, 1.8 Hz, 1H), 2.33 (dd, *J* = 16.8, 4.8 Hz, 1H), 2.21 (s, 3H) ppm. ^13^C-NMR (CDCl_3_, 150 MHz) δ: 152.4, 152.2, 151.5, 148.7, 148.3, 146.6, 144.3, 143.6, 137.6, 136.5, 133.1, 130.5, 130.5, 129.0, 128.2, 127.8, 121.6, 121.5, 121.4, 119.9, 117.3, 112.6, 108.3, 105.9, 63.7, 61.8, 60.4, 56.4, 55.8, 55.8, 45.4, 43.3, 42.7, 41.6, 38.0, 36.9, 25.4, 21.6 ppm. HRESI-MS: *m/z* 668.2987 [M + H]^+^, calculated for C_38_H_42_N_3_O_8_: 668.2966.

#### 2.2.2 Synthesis procedure for the preparation of C14-amino-tetrandrine (M2)

To a solution of C14-nitro-tetrandrine (5g, 7.5 mmol) in methanol was added palladium 5% on carbon (200 mg, wet with ca. 55% water), and the mixture was heated to 80°C under the protection of argon. Hydrazine hydrate (85%, 12.5 ml, 37.5 mmol) was added and the reaction mixture was stirred for 60 min. After complete conversion, the palladium on carbon was filtered through cotton and methanol was removed *in vacuo*. The residue was dissolved in DCM, washed with saturated sodium chloride solution (3 ml × 200 ml), and dried over anhydrous sodium sulfate. The organic fractions were then concentrated *in vacuo*, and C14-amino-tetrandrine was obtained *via* silica gel column chromatography using DCM/MeOH (30/1 v/v, 2% DEA). White amorphous solid (93% yield, purity >95%, HPLC). Mp: 164.7°C. ^1^H-NMR (600 MHz, CDCl_3_) δ: 7.28–7.27 (m,1H), 7.18 (dd, *J* = 8.4, 2.4 Hz, 1H), 6.60 (dd, *J* = 8.4, 2.4 Hz, 1H), 6.50 (s, 1H), 6.46 (s, 1H), 6.31 (s, 1H), 6.29 (s, 1H), 6.12 (dd, *J* = 8.4, 2.4 Hz, 1H), 5.87 (s, 1H), 3.95 (d, *J* = 9.0 Hz, 1H), 3.88 (s, 3H), 3.77 (dd, *J* = 11.4, 5.4 Hz, 1H), 3.74 (s, 3H), 3.68–3.62 (m, 1H), 3.46–3.41 (m, 1H), 3.34 (s, 3H), 3.21 (dd, *J* = 12.6, 5.4 Hz, 1H), 3.11 (s, 3H), 3.05 (dd, *J* = 14.4, 9.0 Hz, 1H), 2.94–2.83 (m, 4H), 2.74 (t, *J* = 11.4 Hz, 1H), 2.67–2.65 (m, 1H), 2.59 (s, 3H), 2.42 (s, 3H), and 2.39–2.33 (m, 2H) ppm. ^13^C-NMR (CDCl_3_, 150 MHz) δ: 156.7, 151.8, 149.5, 148.8, 148.6, 144.4, 142.1, 141.0, 138.2, 133.4, 132.7, 129.4, 128.3, 127.9, 127.5, 122.8, 122.2, 121.5, 121.0, 120.6, 120.4, 112.4, 105.9, 100.7, 64.4, 61.7, 60.0, 56.3, 55.7, 55.6, 45.1, 43.4, 42.5, 41.0, 40.1, 39.0, 24.8, 20.7 ppm. HRESI-MS: *m/z* 638.3225 [M + H]^+^, calculated for C_38_H_44_N_3_O_6_: 638.3245.

#### 2.2.3 Synthesis procedure for the preparation of C14-hydroxyl-tetrandrine (M3)

To a solution of C14-amino-tetrandrine (10 mmol, 1.0 equiv.) in H2SO4 (2 M, 50 ml), NaNO2 (10.5 mmol, 1.05 eq) was slowly added to prepare diazo salt at 0°C. The reaction was monitored using a potassium iodide starch test paper until the reaction was complete. Fresh diazo salt was slowly dropped into H2SO4 (10 M, 50 ml) at 110°C, monitored *via* TLC, and the reaction was completed in 30 min. Excess H2SO4 was neutralized with 20% NaOH in an ice-water bath, and the pH was adjusted to 8 and 9. The aqueous phase was extracted thrice with DCM, and the organic phase was combined and washed thrice with water. The organic phase was recovered *via* dehydration and drying with anhydrous sodium sulfate, filtered, and concentrated under reduced pressure. C14-hydroxyl-tetrandrine was obtained *via* silica gel column chromatography using DCM/MeOH (50/1 v/v, 0.5% DEA). White amorphous solid (61% yield, purity >95%, HPLC). Mp: 216.9°C. ^1^H-NMR (600 MHz, CDCl_3_) δ: 12.54 (s, 1H), 7.28 (dd, *J* = 8.4, 2.4 Hz, 1H), 7.20 (dd, *J* = 8.4, 2.4 Hz, 1H), 6.57 (dd, *J* = 8.4, 2.4 Hz, 1H), 6.53 (s, 1H), 6.48 (s, 1H), 6.37 (s, 1H), 6.31 (s, 1H), 6.15 (dd, *J* = 8.4, 2.4 Hz, 1H), 5.90 (s, 1H), 3.92 (d, *J* = 9.6 Hz, 1H), 3.89 (s, 3H), 3.77–3.74 (m, 4H), 3.64–3.58 (m, 3H), 3.48–3.43 (m, 1H), 3.39 (s, 3H), 3.22 (dd, *J* = 12.6, 5.4 Hz, 1H), 3.14 (s, 3H), 3.08 (dd, *J* = 14.4, 9.6 Hz, 1H), 3.02–2.84 (m, 4H), 2.76 (t, *J* = 12.0 Hz, 1H), 2.72–2.68 (m, 1H), 2.58 (s, 3H), 2.49 (s, 3H), 2.48–2.42 (m, 2H) ppm. ^13^C-NMR (CDCl_3_, 150 MHz) δ: 157.3, 152.5, 152.2, 149.2, 149.0, 148.6, 143.7, 141.9, 138.0, 133.5, 132.8, 129.3, 128.5, 127.8, 126.9, 121.6, 121.1, 120.9, 120.7, 120.1, 119.8, 112.2, 105.5, 101.6, 64.5, 61.4, 60.3, 56.2, 55.8, 55.5, 53.4, 45.1, 42.9, 42.5, 40.3, 39.7, 39.0, 24.7, 20.6 ppm. HRESI-MS: *m/z* 639.3062 [M + H]^+^, calculated for C_38_H_43_N_2_O_7_: 639.3065.

#### 2.2.4 General procedure for the preparation of C14-O-sulfonyl-tetrandrine derivatives 1–40

C14-hydroxyl-tetrandrine (0.079 mmol, 1.0 equiv.), 1-ethyl-(3-dimethylaminopropyl) carbodiimide hydrochloride (EDCI, 0.1185 mmol, 1.5 equiv.), and 4-dimethylaminopyridine (DMAP, 0.0158 mmol, 0.2 equiv.) were dissolved in anhydrous DCM (2 ml) under an argon atmosphere, and the mixture was cooled to 0°C. Sulfonyl chloride (0.1185 mmol, 1.5 equiv.) was slowly added at this temperature, and the reaction mixture was stirred for 30 min. Then, the reaction mixture was warmed to 25°C and stirred for 16 h. The reaction was quenched by the addition of iced water (5 ml). Subsequently, the organic phase was recovered *via* dehydration and drying with anhydrous sodium sulfate, filtered, and concentrated under reduced pressure. The desired product was obtained *via* silica gel rapid column chromatography by purification of the residues from DCM/MeOH (60/1 v/v, 1% DEA). Detailed NMR (^1^H, ^13^C, and ^19^F) and high-resolution mass spectrometry data for 40 tetrandrine derivatives can be found in Supplementary Material.

### 2.3 Biology assays

#### 2.3.1 Cell cultures

All HCC cell lines (HepG-2, SMMC-7721, SK-Hep-1, and QGY-7701) were acquired from Procell Life Science & Technology Co., Ltd. (Wuhan, China). All cell lines were grown in 10% fetal bovine serum (FBS) Dulbecco’s modified Eagle’s medium (DMEM; Gibco, United States) supplemented with streptomycin and penicillin (Solarbio, Beijing, China) in a humidified 5% CO2 incubator at 37°C.

#### 2.3.2 Cell viability assay

A 3-(4,5-dimethyl-thiazol-2-yl)-2,5-diphenyltetrazolium bromide (MTT) assay was conducted to determine the half-maximal inhibitory concentration (IC_50_) values of HepG-2, SMMC-7721, SK-Hep-1, and QGY-7701 cells. Four logarithmic-growing cell lines were seeded in a 96-well culture plate at 5,000 cells/well and allowed to adhere for 12 h before the test drug was added. The cells were treated without or with different concentrations (0.625, 1.25, 2.5, 5, 10, and 20 µM) of compounds for 48 h, after which 20 µL of MTT reagent was added to each well and incubated for further 4 h at 37°C. The medium in the 96-well plate was discarded, formazan crystals were dissolved in dimethyl sulfoxide (150 µL), and the resulting solution was evaluated for absorbance at 490 nm.

#### 2.3.3 Colony formation assay

HepG-2 and SMCC-7721 (1.0 × 10^3^) cells were inoculated into a six-well plate and treated with different concentrations (0, 1, 2, and 4 μM) of **33**. It was incubated at 37°C for 2–3 weeks with 5% CO2 and saturated humidity and removed when macroscopic clones were formed on the plate. Immediately following the removal of the supernatant, the cells were washed twice with phosphate-buffered saline (PBS), and paraformaldehyde (Servicebio, Wuhan, China) fixation was performed for 15 min. After staining with 0.1% crystal violet for 5 min at room temperature, colonies were visualized under a light microscope (×4 magnification).

#### 2.3.4 Wound healing assay

HepG-2 and SMMC-7721 cells were inoculated in a six-well plate and grown until a confluent monolayer was observed. The cells were serum-starved overnight before treatment with a 200 μL spearhead to create a wound in a monolayer cell. The debris was removed by washing with PBS, and fresh low-serum (2% FBS) DMEM was replaced with **33** (0, 1, 2, and 4 μM). Wound closure images were obtained using an optical microscope from 0 to 48 h.

#### 2.3.5 Transwell assay

Cultured HepG-2 and SMMC-7721 cells were resuspended in the upper transwell chamber with Matrigel membrane at a density of 5 × 10^4^/well with 400 μL serum-free DMEM, and the lower transwell chamber was filled with 600 μL of DMEM with 10% FBS. The two cell lines were then treated with different concentrations (0, 1, 2, and 4 μM) of **33**. After 48 h, the cells migrating to the bottom of the insertion membrane were fixed and stained with 0.5% crystal violet for 20 min. Finally, using an inverted microscope, photographs of the invasive cells were taken.

#### 2.3.6 Flow cytometry analysis

Annexin V-fluorescein isothiocyanate (FITC)/propidium iodide (PI) apoptosis assay kit from Absin (Shanghai, China) was used to measure the number of apoptotic cells. HepG-2 and SMMC-7721 cells in the six-well plate were treated with different concentrations (0, 1, 2, and 4 μM) of compound **33**. After 24 h incubation, the cells were washed thrice with PBS and stained with PI and Annexin V-FITC after 24 h. The results were determined using an Agilent Technologies flow cytometer (Agilent, United States) and analyzed using NovoExpress (version 1.6.0).

#### 2.3.7 Western blotting

Total proteins in HepG-2 and SMMC-7721 cells were extracted using a radioimmunoassay precipitation lysis buffer containing 1% phenylmethylsulfonyl fluoride lysis buffer (Servicebio, Wuhan, China). Bicinchoninic acid (Solarbio, Beijing, China) protein assay was used to determine the protein concentrations of the samples. Proteins were separated using 10% sodium dodecyl sulfate-polyacrylamide gel electrophoresis (Servicebio, Wuhan, China) and transferred onto polyvinylidene difluoride membranes (Thermo Scientific, United States). The membrane was blocked with 5% skimmed milk powder, primary antibodies were added, and overnight incubation was carried out at 4°C. The primary antibodies used were Bcl-2-associated X (Bax), cell lymphoma-2 (Bcl-2), caspase-3, cytochrome C, and anti-glyceraldehyde 3-phosphate dehydrogenase. The membrane was further incubated with the secondary antibody at room temperature, followed by washing with PBS. A Bio-Rad ECL chemiluminescence staining kit (Bio-Rad) was used to visualize the protein bands and determine their density.

### 2.4 Statistical analysis

All experimental data were reported as the mean value of three independent experiments plus the standard error of mean and analyzed using GraphPad Prism software (version 8.0.2). Differences between the experimental and control groups were analyzed using one-way analysis of variance. *p* < 0.05 was considered to be statistically significant.

## 3 Results and discussion

### 3.1 Synthesis of tetrandrine derivatives

**SCHEME 1:**
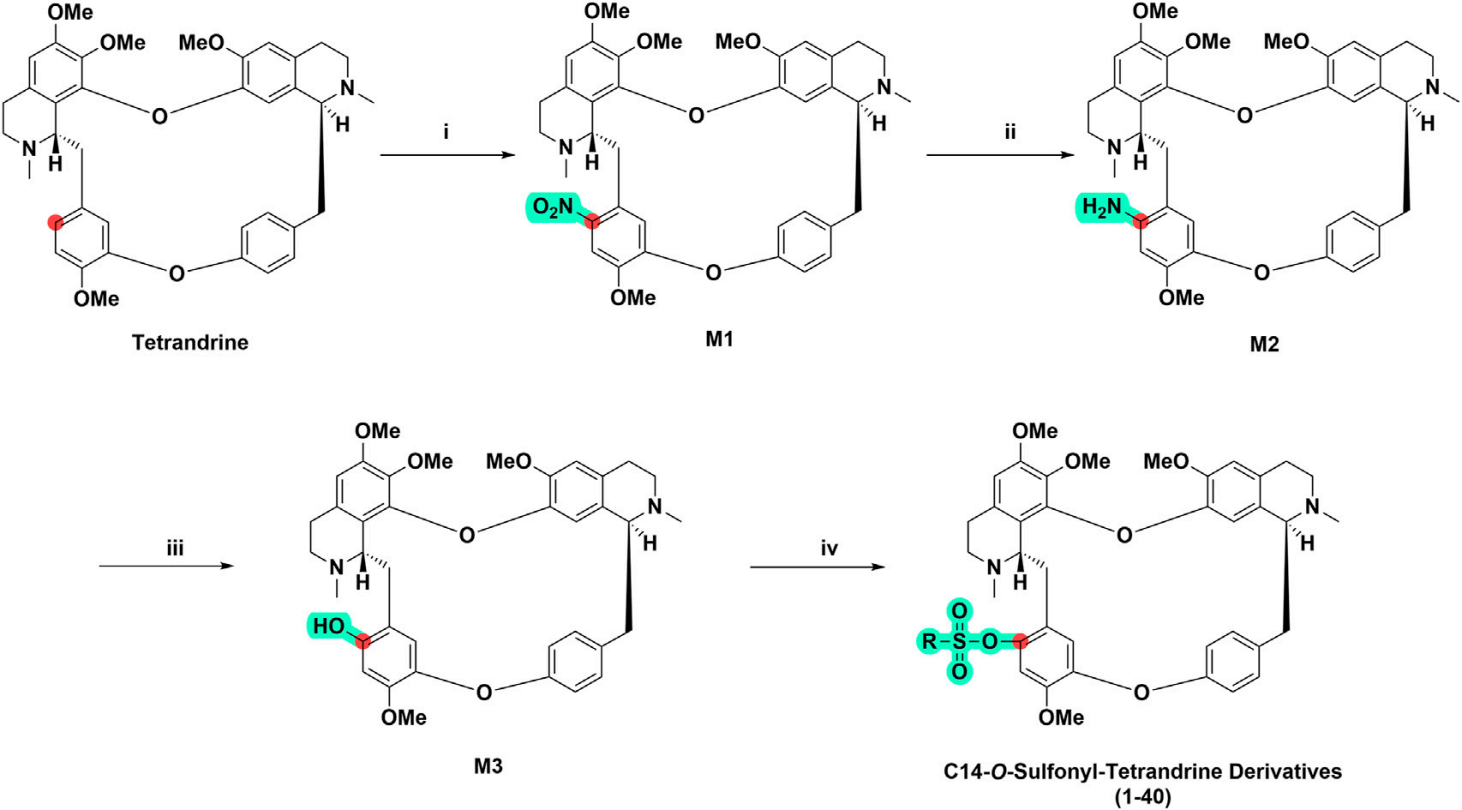
General synthetic route of tetrandrine derivatives. (i) Mixed acid HNO_3_: (CH_3_CO)_2_O = 3:5, v/v), CH_2_Cl_2_, 0°C, 45 min (ii) Pd/C (10%), N_2_H_4_⋅H_2_O, MeOH, 80°C, 1 h (iii) 1) 2 M H_2_SO_4_, NaNO_2_, 0°C, 10 min; 2) 10 M H_2_SO_4_, 110°C, 1 h (iv) Sulfonyl chloride (RSO2Cl), 1-ethyl-(3-dimethylaminopropyl) carbodiimide hydrochloride (EDCI), 4-dimethylaminopyridine (DMAP), CH2Cl2, 0°C, 8 h.

In this study, we focused on the design and synthesis of tetrandrine derivatives with sulfonic acid esters substitution at C-14 and explored their biological activities against four HCC cell lines. As shown in Scheme 1, 40 derivatives of tetrandrine were synthesized. The benzene ring of tetrandrine contains multiple methoxy groups, making it electron rich and prone to electrophilic reactions. First, selective C14-nitrification of tetrandrine was performed by mixing the concentrated nitric acid and acetic anhydride at 0°C to form a nitro derivative of tetrandrine (**M1**). Then, rapid and highly efficient reduction of **M1** to **M2** was achieved in methanol using hydrazine hydrate as the reducing agent and palladium carbon as the catalyst. Subsequently, **M3** was derived from **M2** *via* oxidative hydrolysis of diazo salts intermediate. **M3** was then reacted with sulfonyl chloride, EDCI, and DMAP to give C14-sulfonyl-tetrandrine derivative in good to moderate yields (29%–89%).

### 3.2 Biological evaluation

#### 3.2.1 *In vitro* anticancer activity

**FIGURE 1:**
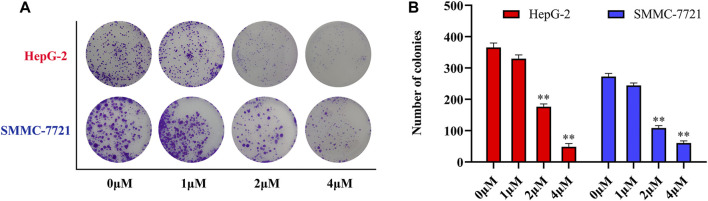
Compound **33** inhibits hepatocellular carcinoma (HCC) cell proliferation *in vitro*. **(A)** HepG-2 and SMMC-7721 cells were treated with different concentrations (0, 1, 2, and 4 μM) of compound **33** to analyze colony formation. **(B)** Data are represented as the mean ± standard error of the mean (SEM) of three independent experiments. *p < 0.05, **p < 0.01.

Antitumor activity was evaluated using MTT assay for the synthetic tetrandrine derivatives (1–40) against four human HCC cell lines, namely HepG-2, Sk-Hep-1 SMMC-7721, and QGY-7701 cell lines. 5-Fluorouracil and tetrandrine were used as standard for positive control. As shown in Table 1, compared with tetrandrine, 70% of the derivatives showed significant inhibitory activity against the four HCC lines. Compound **33** was the most effective, with IC_50_ values of 1.65 ± 0.05, 2.89 ± 0.12, 1.77 ± 0.05, and 2.41 ± 0.08 μM against the four HCC cell lines, respectively. Notably, **33** had more than 20 times greater anti-HCC proliferation effect compared to that of 5-fluorouracil, a commonly used anticancer drug in the clinical setting. Therefore, **33** was chosen for further testing of its biological activity. To verify the proliferative inhibition of **33** on hepatocellular tumors, we selected HepG-2 and SMCC-7721 cells for the colony formation assay. From Figure 1, it can be seen that the ability of both hepatocellular tumors to form colonies decreases significantly as the concentration of administration increases, indicating that **33** has antiproliferative effects on HepG-2 and SMCC-7721 cells.

**TABLE 1:** Cytotoxic activity of the examined compounds against HepG2, Sk-Hep-1, SMMC-7721, QGY-7701 cells.

Com	R	Yield (%)	IC_50_ (μM)			
			HepG-2	SK-Hep-1	SMMC-7721	QGY-7701
1	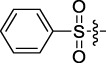	76.4	5.20 ± 0.08	3.66 ± 0.17	4.14 ± 0.09	6.15 ± 0.24
2	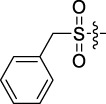	70.5	3.02 ± 0.08	8.69 ± 0.27	6.03 ± 0.14	12.64 ± 0.65
3	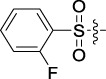	88.9	5.05 ± 0.08	3.19 ± 0.06	4.64 ± 0.11	6.25 ± 0.31
4	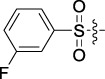	84.4	4.70 ± 0.15	2.92 ± 0.13	4.95 ± 0.08	5.86 ± 0.15
5	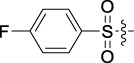	65.2	11.39 ± 0.36	10.08 ± 0.41	8.26 ± 0.09	15.97 ± 0.39
6	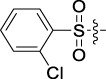	79.1	5.36 ± 0.13	3.08 ± 0.12	5.03 ± 0.13	6.08 ± 0.17
7	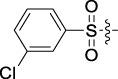	85.8	3.67 ± 0.09	2.866 ± 0.06	3.436 ± 0.13	6.25 ± 0.14
8	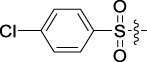	65.7	5.33 ± 0.12	3.05 ± 0.13	4.64 ± 0.12	9.15 ± 0.41
9	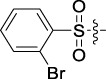	88.2	3.78 ± 0.03	4.12 ± 0.09	2.802 ± 0.08	6.42 ± 0.15
10	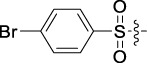	80.9	4.20 ± 0.09	3.78 ± 0.07	4.03 ± 0.12	8.91 ± 0.25
11	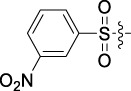	82.6	4.90 ± 0.20	4.58 ± 0.28	5.54 ± 0.21	7.79 ± 0.24
12	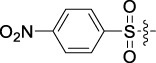	84.0	6.12 ± 0.07	4.11 ± 0.14	5.97 ± 0.19	8.01 ± 0.28
13	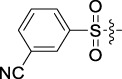	86.9	8.62 ± 0.33	3.86 ± 0.09	3.11 ± 0.06	5.61 ± 0.17
14	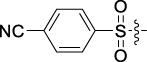	86.8	5.48 ± 0.19	3.92 ± 0.11	5.10 ± 0.13	7.50 ± 0.18
15	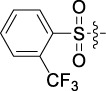	72.5	3.17 ± 0.09	4.77 ± 0.23	8.43 ± 0.17	5.68 ± 0.11
16	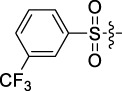	79.5	3.51 ± 0.07	3.33 ± 0.17	4.17 ± 0.10	5.27 ± 0.22
17	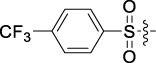	74.4	3.27 ± 0.09	3.09 ± 0.08	3.13 ± 0.10	13.93 ± 0.33
18	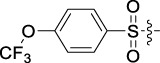	83.6	3.58 ± 0.06	10.63 ± 0.25	4.83 ± 0.17	9.65 ± 0.31
19	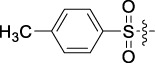	81.2	5.71 ± 0.11	6.58 ± 0.27	6.20 ± 0.18	8.89 ± 0.17
20	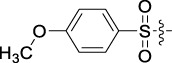	79.8	4.99 ± 0.08	3.70 ± 0.10	4.32 ± 0.22	6.54 ± 0.13
21	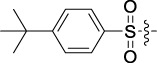	73.0	3.92 ± 0.11	2.95 ± 0.13	4.51 ± 0.08	5.25 ± 0.19
22	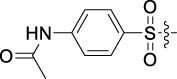	81.5	8.83 ± 0.33	3.21 ± 0.14	5.54 ± 0.10	8.08 ± 0.33
23	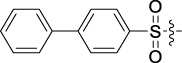	82.2	3.50 ± 0.07	4.06 ± 0.15	7.69 ± 0.22	>20
24	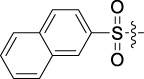	71.1	5.55 ± 0.13	8.07 ± 0.18	6.23 ± 0.15	5.06 ± 0.19
25	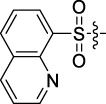	77.4	9.80 ± 0.21	8.06 ± 0.21	6.09 ± 0.16	7.114 ± 0.27
26	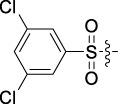	77.5	4.19 ± 0.06	4.75 ± 0.21	4.32 ± 0.25	10.49 ± 0.35
27	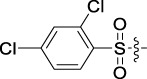	88.7	5.09 ± 0.09	3.27 ± 0.07	3.17 ± 0.13	6.21 ± 0.18
28	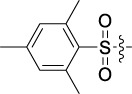	68.6	2.26 ± 0.07	3.08 ± 0.10	3.41 ± 0.09	4.49 ± 0.11
29	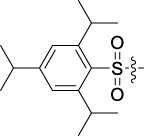	55.9	13.14 ± 0.19	12.47 ± 0.36	5.36 ± 0.17	>20
30	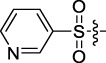	59.6	9.36 ± 0.44	6.81 ± 0.12	7.60 ± 0.27	7.46 ± 0.25
31	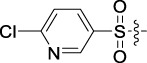	75.9	3.06 ± 0.15	5.73 ± 0.10	3.23 ± 0.15	6.54 ± 0.27
32	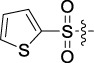	61.8	4.40 ± 0.22	3.08 ± 0.17	5.04 ± 0.06	5.92 ± 0.24
33	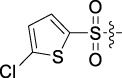	87.3	1.65 ± 0.05	2.89 ± 0.12	1.77 ± 0.05	2.41 ± 0.08
34	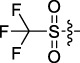	86.7	12.37 ± 0.38	7.58 ± 0.34	8.12 ± 0.24	13.95 ± 0.42
35	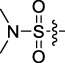	28.5	13.83 ± 0.40	3.45 ± 0.17	11.46 ± 0.54	>20
36	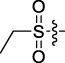	88.1	10.22 ± 0.34	5.78 ± 0.27	9.59 ± 0.24	14.62 ± 0.42
37	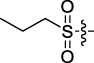	91.4	7.83 ± 0.19	5.96 ± 0.25	8.75 ± 0.34	12.23 ± 0.27
38	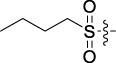	85.6	7.54 ± 0.15	5.95 ± 0.12	7.29 ± 0.08	12.58 ± 0.56
39	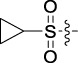	80.4	11.86 ± 0.55	9.00 ± 0.19	8.79 ± 0.18	12.55 ± 0.44
40	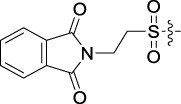	87.3	11.56 ± 0.48	12.16 ± 0.58	15.16 ± 0.57	>20
Tet	-	-	9.68 ± 0.24	16.01 ± 0.26	11.24 ± 0.34	14.98 ± 0.11
M3	-	62.5	9.35 ± 0.26	10.84 ± 0.60	11.86 ± 0.44	16.96 ± 0.48
5-Fluorouracil	-	-	57.86 ± 2.94	74.707 ± 2.42	82.12 ± 4.61	50.84 ± 1.55

Cells were treated with compounds by MTT, assay for 48 h. IC_50_ values are indicated as mean IC_50_ ± SEM (μM).

Based on the IC_50_ results of the above 40 derivatives, the preliminary structure–activity relationship (SAR) analysis of C14-sulfonate-tetrandrine was analyzed. In this study, we replaced sulfonyl amide with a sulfonate group for the first time at the C-14 position of tetrandrine according to the bioisostere principle and found that the antitumor activities of these novel derivatives were significantly enhanced compared to their prototype compounds. We hypothesized that different substituents on sulfonate groups may affect the anti-HCC activity of compounds. However, the replacement of the electron-withdrawing or electron-donating substituents on the benzene ring of the sulfonate substituents had little effect on the antitumor activities of the derivatives. Interestingly, compound **29**, with three isopropyl substituents on the benzene ring on the sulfonate group, showed selectivity for SMCC-7721 cells among the four HCC cell lines, while compound **28** with a similar substitution structure, showed significant inhibitory activity against the four HCC cell lines. For compounds **30**–**33**, sulfonate derivatives with heterocyclic ring substitutions significantly enhanced the antitumor activity, and the 5-chlorothiazole sulfonate derivative, compound **33**, showed the best inhibitory activity among the 40 derivatives against the four HCC cell lines. Unfortunately, aliphatic substituents in sulfonate groups have a limited effect on the enhancement of antitumor activity. Indeed, the more details of structure–activity relationship of these compounds require further elucidation in future studies.

#### 3.2.2 Migration and invasion assays

**FIGURE 2:**
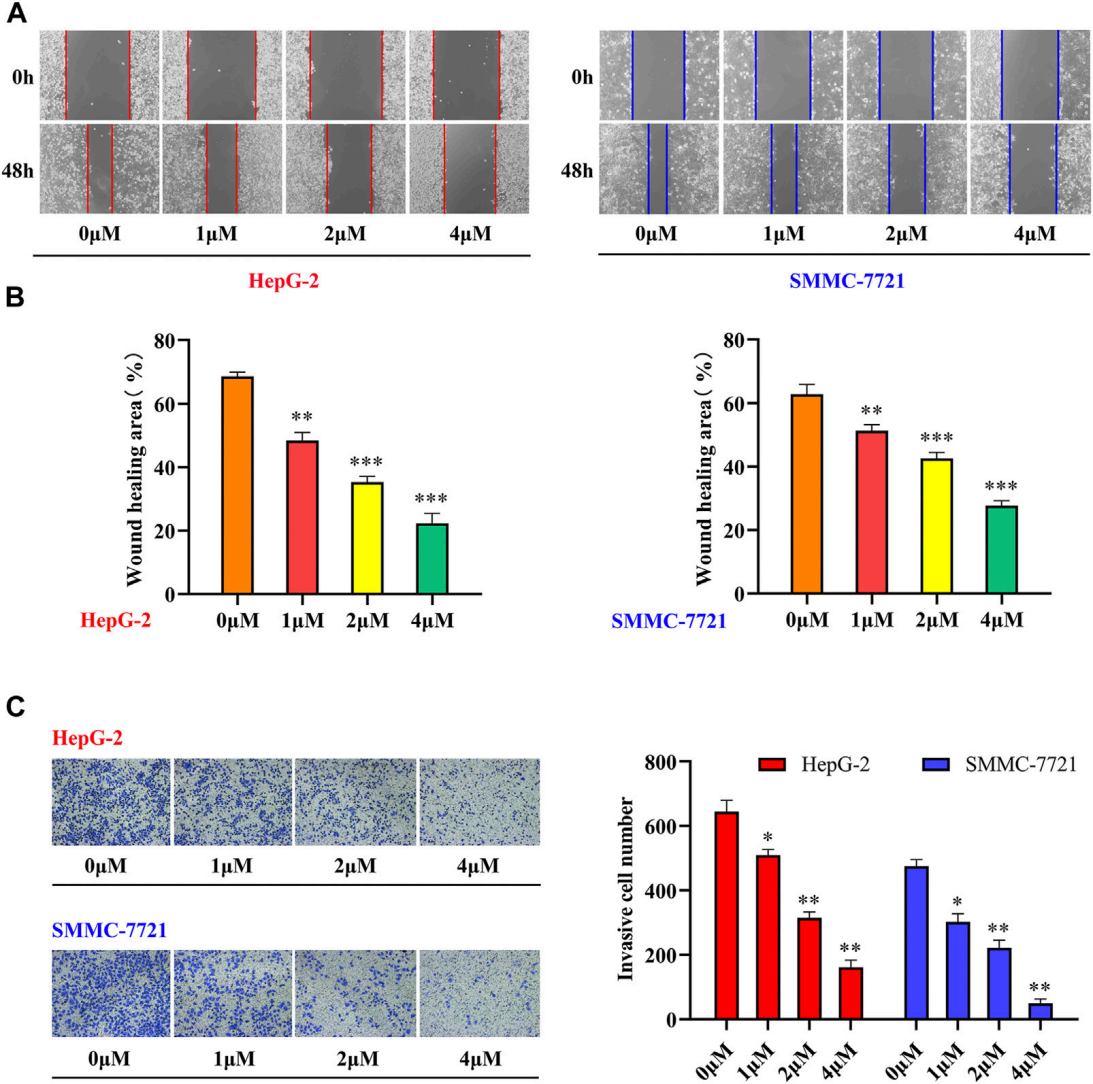
Compound **33** suppresses the motility of HCC cells *in vitro*. **(A,B)** HepG-2 and SMMC-7721 cells were treated with different concentrations (0, 1, 2, and 4 μM) of **33** to analyze wound healing. **(C)** Invasion cell numbers were analyzed using a transwell assay in HepG-2 and SMMC-7721 cells treated with different concentrations (0, 1, 2, and 4 μM) of compound **33**. *p < 0.05, **p < 0.01, ***p < 0.001.

Tumor metastasis is a multistep, multifactorial, and extremely complex process. Although the metastatic characteristics of different tumors vary, some of the key steps in the metastatic process are essentially the same, with migration and invasion being the most common features (Zeeshan and Mutahir, 2017). There is evidence that tetrandrine can not only inhibit the metastasis of human HCC cells but also inhibit the migration and invasion of different human tumor cells, such as prostate cancer, kidney cancer, and colorectal adenocarcinoma (Liu W. et al., 2015; Chen et al., 2017). To further investigate the effects of tetrandrine derivatives on HCC metastasis, the effects of compound **33** on the migration and invasion of HepG-2 and 7721 cells were investigated. A wound-healing assay was conducted to examine the inhibition of SMMC-7721 and HepG-2 cell migration by tetrandrine derivatives. As shown in Figure 2, the scratch area was virtually covered after 48 h in the control group, with wound healing rates of 69 ± 1.4 and 63 ± 3.1%, respectively. In contrast, cells treated with **33** showed slow recovery of scratched areas at increasing concentrations (1, 2, and 4 μM). Furthermore, cell invasion was assessed using a standard transwell assay of HepG-2 and SMMC-7721 cells. Among all concentrations tested, **33** significantly suppressed the invasion of HepG-2 and SMMC-7721 cells. Compared to the control group, the addition of 4 μM **33** significantly reduced the number of invasive HepG-2 and SMMC-7721 cells from 644 ± 35 and 475 ± 21 to 161 ± 22 and 49 ± 14, respectively. These results suggest that **33** inhibits the migration and invasion of HepG-2 and SMMC-7721 HCC cells *in vitro*.

#### 3.2.3 Apoptosis analysis

**FIGURE 3:**
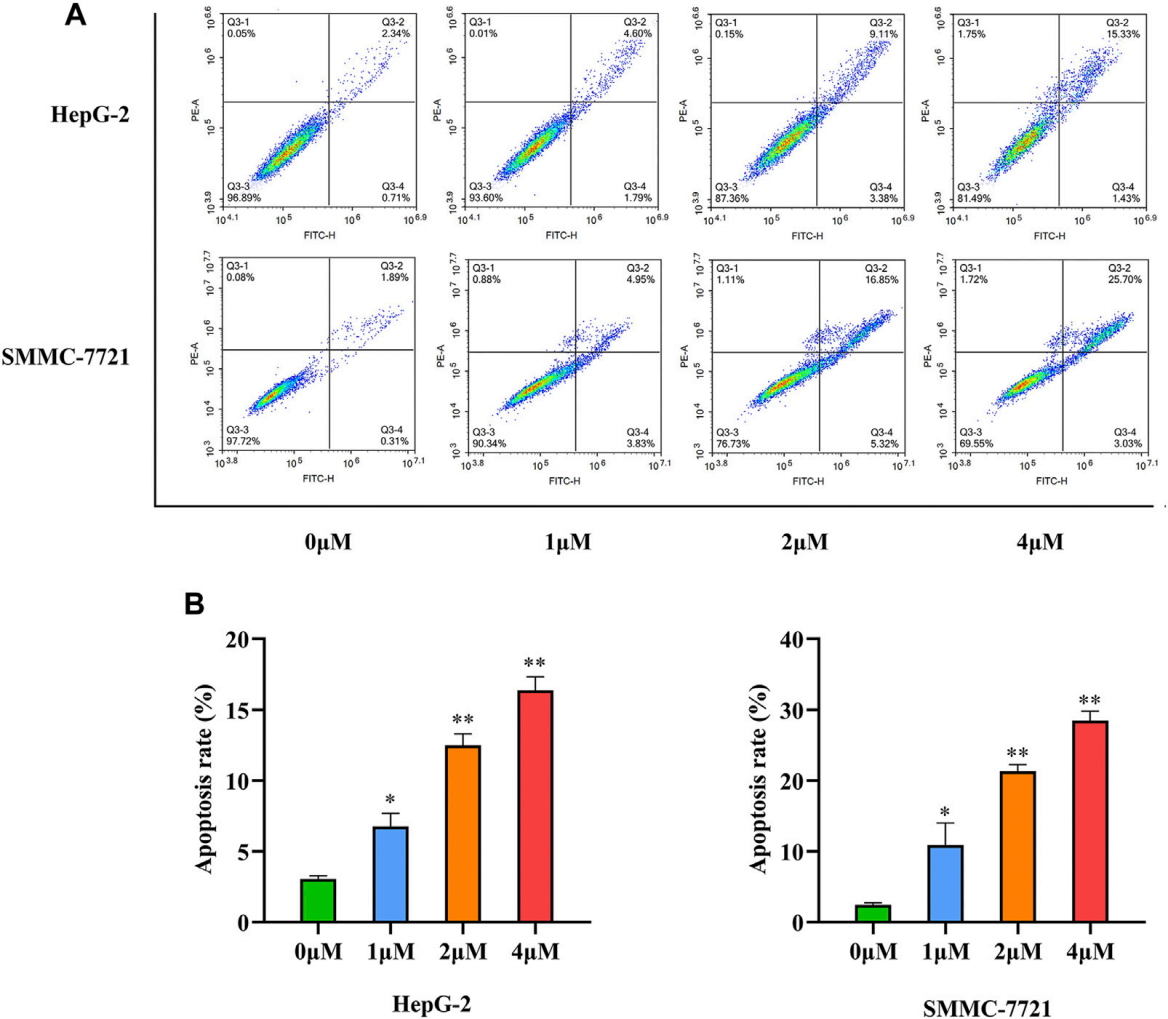
Compound **33** induces the apoptosis of HCC cells *in vitro*. **(A)** Apoptosis of HepG-2 and SMMC-7721 cells with different concentrations (0, 1, 2, and 4 μM) of **33** was analyzed *via* flow cytometry. **(B)**. Based on three independent experiments, GraphPad Prism software (version 8.0.2) was used to quantify the percentage of apoptotic cells. Results of three independent experiments are presented as the mean ± SEM. *p < 0.05, **p < 0.01.

Cells actively participate in the process of apoptosis, which plays a crucial role in the destruction of tumor cells, under gene control. Most natural medicines and their derivatives induce cancer cell apoptosis (Sadeghi et al., 2019; Ashrafizadeh et al., 2020). To examine the apoptotic activity of compound **33** in HepG-2 and SMMC-7721 cells. Annexin V-FITC/PI double staining was performed. As shown in Figure 3, a dose-dependent increase in apoptosis was observed in the two cell lines after treatment with **33**, with the apoptotic proportion of HepG-2 cells changing from 3.05 to 6.39, 12.49, and 16.76%, and the apoptotic proportion of SMCC-7721 changing from 2.25 to 8.78, 22.17, and 28.73%, respectively. These results indicate that inducing apoptosis may be a potential mechanism of action of **33** for its antiproliferative activity.

#### 3.2.4 Western blotting

**FIGURE 4:**
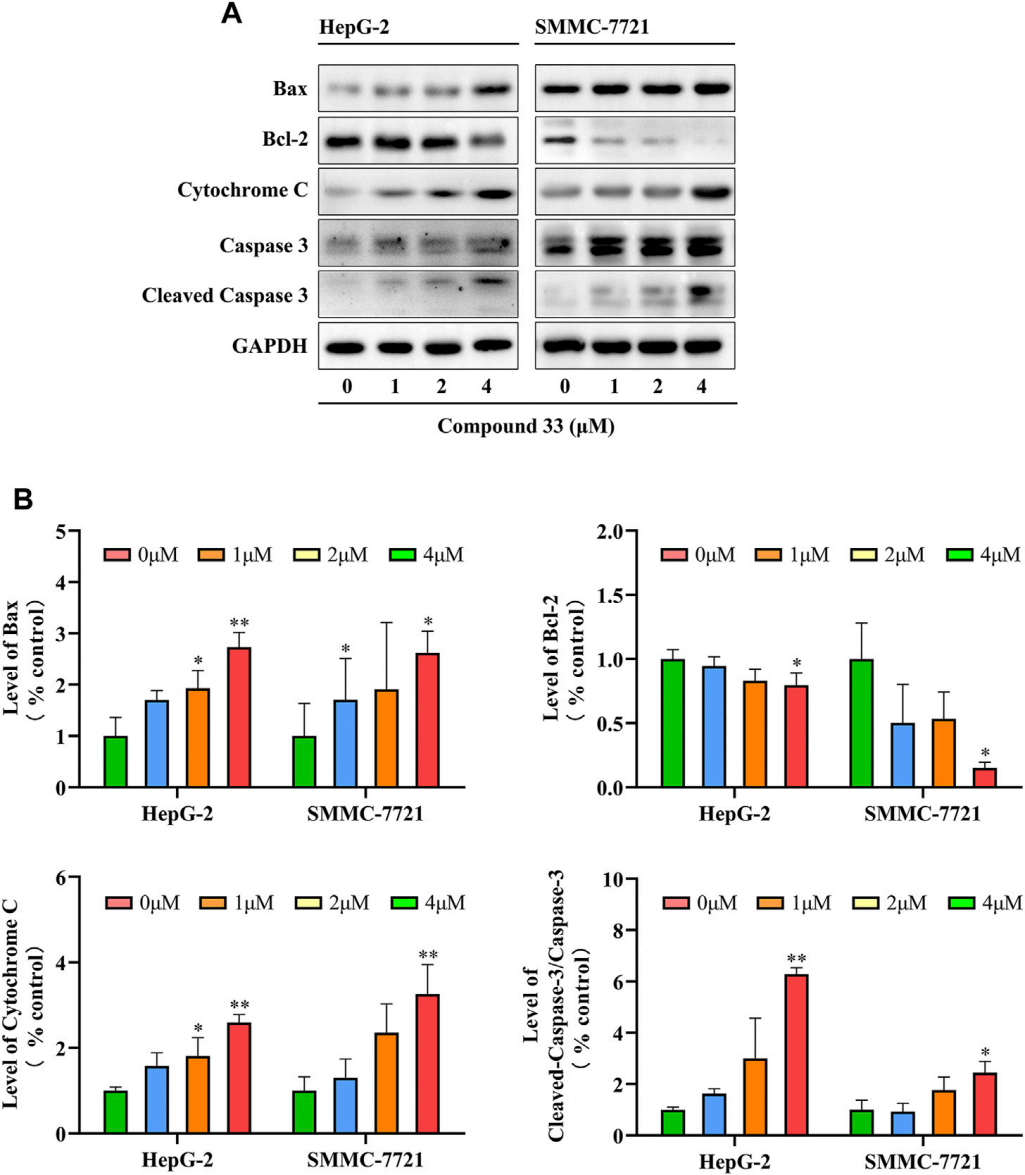
Compound **33** induces the apoptosis of HepG-2 and SMMC-7721 cells *via* the mitochondrial pathway. **(A)** Western blotting revealed that 33 increases the mitochondrial pathway protein expression in HepG-2 and SMMC-7721 cells. **(B)** Western blot results statistics were performed using GraphPad Prim software (version 8.0.2). Results of three independent experiments are presented as the mean ± SEM. *p < 0.05, **p < 0.01.

Based on the flow cytometry results, compound **33** could induce the apoptosis of HCC cells. Interestingly, multiple proteins can participate in the apoptotic signaling pathways. According to the initiation of caspase, apoptosis can be divided into three basic pathways, death receptor-, mitochondria-, and endoplasmic reticulum-mediated apoptosis (Elmore, 2007). Pro-apoptotic factor, cytochrome C, plays a major role (Pistritto et al., 2016). In addition, mitochondrial apoptosis is regulated by the pro-apoptotic protein Bax and anti-apoptotic protein Bcl-2. To elucidate the mechanism of apoptosis by **33** in HepG-2 and SMMC-7721 cells, levels of apoptosis-inducing proteins were measured *via* western blotting. Total cell lysates were prepared for western blotting analysis after treatment with **33** (0, 1, 2, and 4 μM) for 48 h. As shown in Figure 4A, a reduction in Bcl-2 protein expression levels and an increase in Bax and cytochrome C protein expression levels was observed after **33** treatments, indicating that mitochondrial and endoplasmic reticulum functions may be disrupted, resulting in cytochrome C release. Through mitochondrial-mediated mechanisms, **33** regulated apoptosis in HepG-2 and SMMC-7721 cells. By releasing cytochrome C, caspase-9 is activated, which then activates other downstream molecules, such as caspase-3, leading to the final execution phase of cell death. To investigate whether cleaved caspase-3 was also involved in the apoptosis of HepG-2 and SMMC-7721 cells induced by **33**, western blotting analysis was conducted to determine the levels of cleaved caspase-3. As illustrated in Figure 4B, the cleaved caspase-3 levels increased in a dose-dependent manner. Moreover, **33** induced the apoptosis of HepG-2 and SMCC-7721 cells is a caspase-mediated apoptotic pathway.

## 4 Conclusion

In conclusion, C14-hydroxyl-tetrandrine intermediate compound was obtained for the first time, 40 tetrandrine derivatives were designed and synthesized and their antitumor activities were evaluated against four HCC cell lines *in vitro*. Introduction of sulfonate groups improved the cytotoxicity of tetrandrine against the four HCC cell lines. Among them, compound **33** exerted the best activity on HCC cells, with IC_50_ values of 1.65 ± 0.05, 2.89 ± 0.12, 1.77 ± 0.05, and 2.41 ± 0.08 μM for the four HCC cell lines, respectively, which is more than 20 times higher than that of 5-fluorouracil, a commonly used anti-HCC drug in clinical practice. **33** induced the apoptosis of HepG-2 and SMMC-7721 cells, which may be related to the caspase-dependent apoptosis pathway caused by the mitochondria. In addition, **33** inhibited the proliferation, migration, and invasion of HepG-2 and SMMC-7721 cells *via* clone formation, scratch, and invasion assays. More details of the mechanism of action of **33** against HepG-2 and SMMC-7721 cells were obtained *via* proliferation, migration, and invasion assays. Thus, C14-sulfonate-tetrandrine derivatives **33** may be a potential candidate for the development of novel anti-human HCC cell therapies.

## Data Availability

The original contributions presented in the study are included in the article/Supplementary Material, further inquiries can be directed to the corresponding authors.

## References

[B1] Anwanwan D. Singh S. K. Singh S. Saikam V. Singh R. (2020). Challenges in liver cancer and possible treatment approaches. Biochim. Biophys. Acta Rev. Cancer 1873, 188314. 10.1016/j.bbcan.2019.188314 31682895 PMC6981221

[B2] Ashrafizadeh M. Najafi M. Makvandi P. Zarrabi A. Farkhondeh T. Samarghandian S. (2020). Versatile role of curcumin and its derivatives in lung cancer therapy. J. Cell. Physiol. 235, 9241–9268. 10.1002/jcp.29819 32519340

[B3] Bhagya N. Chandrashekar K. R. (2022). Autophagy and cancer: Can tetrandrine be a potent anticancer drug in the near future? Biomed. Pharmacother. 148, 112727. 10.1016/j.biopha.2022.112727 35219119

[B4] Bhagya N. Chandrashekar K. R. (2016). Tetrandrine – a molecule of wide bioactivity. Phytochemistry 125, 5–13. 10.1016/j.phytochem.2016.02.005 26899361

[B5] Chen S. Liu W. Wang K. Fan Y. Chen J. Ma J. (2017). Tetrandrine inhibits migration and invasion of human renal cell carcinoma by regulating Akt/NF-κB/MMP-9 signaling. PLOS One 12, e0173725. 10.1371/journal.pone.0173725 28288190 PMC5348026

[B6] Dimitroulis D. Damaskos C. Valsami S. Davakis S. Garmpis N. Spartalis E. (2017). From diagnosis to treatment of hepatocellular carcinoma: An epidemic problem for both developed and developing world. World J. Gastroenterol. 23, 5282–5294. 10.3748/wjg.v23.i29.5282 28839428 PMC5550777

[B7] Elmore S. (2007). Apoptosis: A review of programmed cell death. Toxicol. Pathol. 35, 495–516. 10.1080/01926230701320337 17562483 PMC2117903

[B8] Gao L. N. Feng Q. S. Zhang X. F. Wang Q. S. Cui Y. L. (2016). Tetrandrine suppresses articular inflammatory response by inhibiting pro-inflammatory factors via NF-κB inactivation. J. Orthop. Res. 34, 1557–1568. 10.1002/jor.23155 26748661

[B9] Gao X. Z. Lv X. T. Zhang R. R. Luo Y. Wang M. X. Chen J. S. (2021). Design, synthesis and *in vitro* anticancer research of novel tetrandrine and fangchinoline derivatives. Bioorg. Chem. 109, 104694. 10.1016/j.bioorg.2021.104694 33601141

[B10] Heister P. M. Poston R. N. (2020). Pharmacological hypothesis: TPC2 antagonist tetrandrine as a potential therapeutic agent for COVID‐19. Pharmacol. Res. Perspect. 8, e00653. 10.1002/prp2.653 32930523 PMC7503088

[B11] Jamieson C. Moir E. M. Rankovic Z. Wishart G. (2006). Medicinal chemistry of hERG optimizations: Highlights and hang-ups. J. Med. Chem. 49, 5029–5046. 10.1021/jm060379l 16913693

[B12] Kim D. W. Talati C. Kim R. (2017). Hepatocellular carcinoma (HCC): Beyond sorafenib—chemotherapy. J. Gastrointest. Oncol. 8, 1968–1980. 10.21037/jgo.2016.09.07 PMC5401857 28480065

[B13] Lan J. Huang L. Lou H. Chen C. Liu T. Hu S. (2018). Design and synthesis of novel C14-urea-tetrandrine derivatives with potent anti-cancer activity. Eur. J. Med. Chem. 143, 1968–1980. 10.1016/j.ejmech.2017.11.007 29133049

[B14] Lan J. Wang N. Huang L. Liu Y. Ma X. Lou H. (2017). Design and synthesis of novel tetrandrine derivatives as potential anti-tumor agents against human hepatocellular carcinoma. Eur. J. Med. Chem. 127, 554–566. 10.1016/j.ejmech.2017.01.008 28109948

[B15] Li D. Liu H. Liu Y. Zhang Q. Liu C. Zhao S. (2017). Design, synthesis and biological activities of tetrandrine and fangchinoline derivatives as antitumer agents. Bioorg. Med. Chem. Lett. 27, 533–536. 10.1016/j.bmcl.2016.12.029 28057423

[B16] Lin Y. J. Peng S. F. Lin M. L. Kuo C. L. Lu K. W. Liao C. L. (2016). Tetrandrine induces apoptosis of human nasopharyngeal carcinoma NPC-TW 076 cells through reactive oxygen species accompanied by an endoplasmic reticulum stress signaling pathway. Molecules 21, 1353. 10.3390/molecules21101353 27754332 PMC6273859

[B17] Liu C. Chen K. Chen P. (2015a). Treatment of liver cancer. Cold Spring Harb. Perspect. Med. 5, a021535. 10.1101/cshperspect.a021535 26187874 PMC4561392

[B18] Liu W. Kou B. Ma Z. K. Tang X. S. Lv C. Ye M. (2015b). Tetrandrine suppresses proliferation, induces apoptosis, and inhibits migration and invasion in human prostate cancer cells. Asian J. Androl. 17, 850–853. 10.4103/1008-682X.142134 25677131 PMC4577603

[B19] Ma J. W. Zhang Y. Li R. Ye J. C. Li H. Y. Zhang Y. K. (2015). Tetrandrine suppresses human glioma growth by inhibiting cell survival, proliferation and tumour angiogenesis through attenuating STAT3 phosphorylation. Eur. J. Pharmacol. 764, 228–239. 10.1016/j.ejphar.2015.06.017 26086859

[B20] Newman D. J. Cragg G. M. (2020). Natural products as sources of new drugs over the nearly four decades from 01/1981 to 09/2019. J. Nat. Prod. 83, 770–803. 10.1021/acs.jnatprod.9b01285 32162523

[B21] Pistritto G. Trisciuoglio D. Ceci C. Garufi A. D’Orazi G. (2016). Apoptosis as anticancer mechanism: Function and dysfunction of its modulators and targeted therapeutic strategies. Aging 8, 603–619. 10.18632/aging.100934 27019364 PMC4925817

[B22] Rishton G. M. (2008). Natural products as a robust source of new drugs and drug leads: Past successes and present day issues. Am. J. Cardiol. 101, 43D–49D. 10.1016/j.amjcard.2008.02.007 18474274

[B23] Sadeghi S. Davoodvandi A. Pourhanifeh M. H. Sharifi N. ArefNezhad R. Sahebnasagh R. (2019). Anti-cancer effects of cinnamon: Insights into its apoptosis effects. Eur. J. Med. Chem. 178, 131–140. 10.1016/j.ejmech.2019.05.067 31195168

[B24] Shi X. Mao Y. Saffiotti U. Wang L. Rojanasakul Y. Leonard S. S. (1995). Antioxidant activity of tetrandrine and its inhibition of quartz‐induced lipid peroxidation. J. Toxicol. Environ. Health 46, 233–248. 10.1080/15287399509532031 7563220

[B25] Siegel R. L. Miller K. D. Fuchs H. E. Jemal A. (2022). Cancer statistics, 2022. CA Cancer J. Clin. 72, 7–33. 10.3322/caac.21708 35020204

[B26] Song J. R. Lan J. J. Chen C. Hu S. C. Song J. R. Liu W. (2018). Design, synthesis and bioactivity investigation of tetrandrine derivatives as potential anti-cancer agents. Medchemcomm 9, 1131–1141. 10.1039/C8MD00125A 30109000 PMC6072089

[B27] Sung H. Ferlay J. Siegel R. L. Laversanne M. Soerjomataram I. Jemal A. (2021). Global cancer statistics 2020: GLOBOCAN estimates of incidence and mortality worldwide for 36 cancers in 185 countries. CA Cancer J. Clin. 71, 209–249. 10.3322/caac.21660 33538338

[B28] Wei X. Qu T. L. Yang Y. F. Xu J. F. Li X. W. Zhao Z. B. (2016). Design and synthesis of new tetrandrine derivatives and their antitumor activities. J. Asian Nat. Prod. Res. 18, 966–975. 10.1080/10286020.2016.1188085 27244089

[B29] Wu C. Z. Lai L. Hu X. Lei R. R. Yang Y. F. (2013). Synthesis and antitumor activity of tetrandrine derivatives. J. Asian Nat. Prod. Res. 15, 993–1002. 10.1080/10286020.2013.823950 23944846

[B30] Yi K. Liu S. Liu P. Luo X. Zhao J. Yan F. (2022). Synergistic antibacterial activity of tetrandrine combined with colistin against MCR-mediated colistin-resistant Salmonella. Biomed. Pharmacother. 149, 112873. 10.1016/j.biopha.2022.112873 35349932

[B31] Zeeshan R. Mutahir Z. (2017). Cancer metastasis - tricks of the trade. Bosn. J. Basic Med. Sci. 17, 172–182. 10.17305/bjbms.2017.1908 28278128 PMC5581965

[B32] Zhang Y. X. Liu X. M. Wang J. Li J. Liu Y. Zhang H. (2015). Inhibition of AKT/FoxO3a signaling induced puma expression in response to p53-independent cytotoxic effects of H1: A derivative of tetrandrine. Cancer Biol. Ther. 16, 965–975. 10.1080/15384047.2015.1040950 25893985 PMC4622009

