# Supplementary material for: A novel class of C14-sulfonate-tetrandrine derivatives as potential chemotherapeutic agents for hepatocellular carcinoma
Source: Front Chem. 2023 Jan 10;10:1107824. doi: 10.3389/fchem.2022.1107824 (PMC9871304; doi:10.3389/fchem.2022.1107824)
Supplement: Supplementary file 2 [file DataSheet1.docx]

**Supplementary Material**

**A Novel Class of C14-Sulfonate-Tetrandrine Derivatives as Potential Chemotherapeutic Agents for Hepatocellular Carcinoma**

Taibai Jiang^1†^, Guangtong Xie^3†^, Zhirui Zeng^4^, Junjie Lan^6^, Hanfei Liu^1^, Jinyu Li^1^, Hai Ren^1*^, Tengxiang Chen^4,5*^, Weidong Pan^1,2*^

^1^School of Basic Medicine/State Key Laboratory of Functions and Applications of Medicinal Plants, Guizhou Medical University, Guiyang 550025, Guizhou, PR China; Key Laboratory of Chemistry for Natural Products of Guizhou Province and Chinese Academy of Sciences, Guiyang, 550014, PR China.

^2^School of Pharmaceutical Sciences, Guizhou University, Guiyang 550025, PR China.

^3^School of Pharmacy, Guizhou University of traditional Chinese Medicine, Guiyang 550025, PR China.

^4^Guizhou Provincial Key Laboratory of Pathogenesis & Drug Research on Common Chronic Diseases, Department of Physiology, School of Basic Medical Sciences, Guizhou Medical University, Guiyang 550009, PR China.

^5^Precision Medicine Research Institute of Guizhou, Affiliated Hospital of Guizhou Medical University, Guiyang 550025, PR China.

^6^Department of Pharmacy, Guizhou Provincial People’s Hospital, Guiyang 550499, PR China.

^†^Equal contribution

**^*^ Correspondence:**

Hai Ren, State Key Laboratory of Functions and Applications of Medicinal Plants, Guizhou Medical University, Guiyang, 550014, PR China; the Key Laboratory of Chemistry for Natural Products of Guizhou Province and Chinese Academy of Sciences, Guiyang, 550014, PR China; renh0206@163.com

Tengxiang Chen, Guizhou Provincial Key Laboratory of Pathogenesis & Drug Research on Common Chronic Diseases, Department of Physiology, School of Basic Medical Sciences, Guizhou Medical University, Guiyang 550009, PR China; txch@gmc.edu.cn

Weidong Pan, State Key Laboratory of Functions and Applications of Medicinal Plants, Guizhou Medical University, Guiyang, 550014, PR China; the Key Laboratory of Chemistry for Natural Products of Guizhou Province and Chinese Academy of Sciences, Guiyang, 550014, PR China; School of Pharmaceutical Sciences, Guizhou University, Guiyang 550025, PR China; wdpan@163.com

Contents

[Table S1: Purity data of intermediatess (HPLC) 3](#_Toc121298104)

[Characterization information of C14-*O*-Sulfonyl-Tetrandrine Derivatives 1-40 5](#_Toc121298105)

[NMR and MS Spectra 25](#_Toc121298106)

# Table S1: Purity data of intermediates (HPLC)

| Compound | Time retention (min) | Purity % (HPLC) |
| --- | --- | --- |
| M1 | 10.334 | 98.64 |
| M2 | 15.171 | 97.33 |
| M3 | 12.363 | 98.08 |

^a^Column: CHIRALPAK AD-H (Lot No. ADH0CE-BV081); Column size (0.46 cm I.D. × 25 cm L.); Mobile phase: n-hexane-ethanol-diethylamine (85: 15: 0.8, V/V/V); Wavelength: UV 254 nm; Flow rate: 0.8 mL/min; Temperature: 25°C; Injection volume: 10 μL.

**M1**


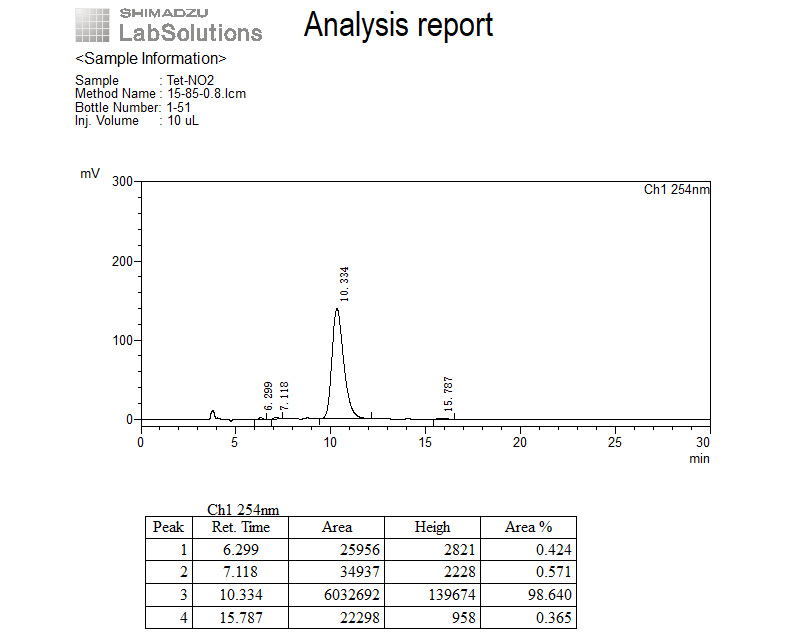


**M2**


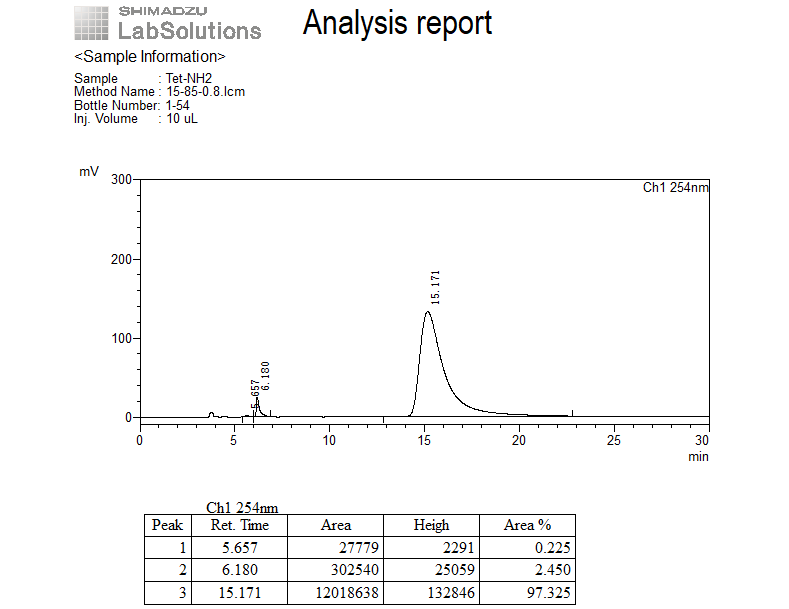


**M3**


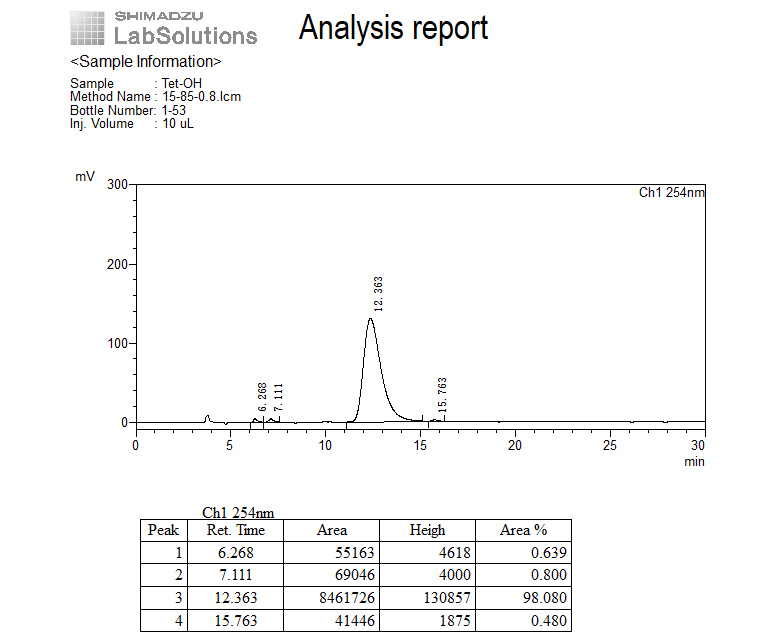


# Characterization information of C14-*O*-Sulfonyl-Tetrandrine Derivatives 1-40

Compound **1** 14-*O*-(benzenesulfonyl)-tetrandrine

light yellow amorphous solid, yield 76.4%; m.p.: 124.3 ℃; ^1^H NMR (600 MHz, CDCl_3_) δ: 7.95*-*7.91 (m, 2H), 7.70*-*7.65 (m, 1H), 7.57*-*7.52 (m, 2H), 7.36 (dd, *J =* 8.4, 2.4 Hz, 1H), 7.09 (dd, *J =* 8.4, 2.4 Hz, 1H), 6.82 (dd, *J =* 8.4, 2.4 Hz, 1H), 6.51 (d, *J =* 3.0 Hz, 2H), 6.34-6.32 (m, 2H), 6.31 (s, 1H), 5.95 (s, 1H), 3.91 (dd, *J =* 10.8, 5.4 Hz, 1H), 3.76 (s, 3H), 3.69 (d, *J =* 10.8 Hz, 1H), 3.67 (s, 3H), 3.52 (m, 1H), 3.43 (m, 1H), 3.36 (s, 3H), 3.28 (dd, *J =* 12.0, 5.4 Hz, 1H), 3.20 (s, 3H), 3.04 (dd, *J =* 14.4, 10.8 Hz, 1H), 2.99*-*2.83 (m, 4H), 2.81*-*2.73 (m, 2H), 2.64 (s, 3H), 2.41 (dd, *J =* 16.8, 5.4 Hz, 1H), 2.27*-*2.24 (m, 4H) ppm; ^13^C NMR (150 MHz, CDCl_3_) δ: 152.6, 151.4, 148.6, 148.3, 147.9, 146.6, 143.5, 141.6, 137.7, 136.1, 135.8, 134.1, 132.7, 130.5, 129.1, 128.6, 128.1, 127.7, 127.6, 127.5, 122.1, 122.1, 122.0, 119.6, 116.0, 112.7, 105.9, 105.8, 63.7, 61.5, 60.3, 55.9, 55.8, 55.8, 45.2, 43.4, 42.5, 42.0, 37.9, 35.0, 25.2, 21.9 ppm. HR-ESIMS: *m/z* 779.2998 [M+H]^+^, calculated for C_44_H_47_N_2_O_9_S: 779.2997.

Compound **2** 14-*O*-(alpha-toluenesulfonyl)-tetrandrine

light yellow amorphous solid, yield 70.5%; m.p.: 130.6 ℃; ^1^H NMR (600 MHz, CDCl_3_) δ: 7.46-7.43 (m, 2H), 7.41-7.38 (m, 3H), 7.36 (dd, *J =* 8.4, 2.4 Hz, 1H), 7.07 (dd, *J =* 8.4, 2.4 Hz, 1H), 6.84 (dd, *J =* 8.4, 2.4 Hz, 1H), 6.66 (s, 1H), 6.57 (s, 1H), 6.52 (s, 1H), 6.35 (dd, *J =* 8.4, 2.4 Hz, 1H), 6.32 (s, 1H), 5.98 (s, 1H), 4.71 (s, 2H), 3.92 (dd, *J =* 10.8, 5.4 Hz, 1H), 3.83 (d, *J =* 10.8 Hz, 1H), 3.76-3.75 (m, 6H), 3.65-3.58 (m, 1H), 3.46-3.42 (m, 1H), 3.38 (s, 3H), 3.29 (dd, *J* = 12.6, 6.0 Hz, 1H), 3.21-3.14 (m, 4H), 2.99-2.88 (m, 4H), 2.82-2.73(m, 2H), 2.64 (s, 3H), 2.49-2.44 (m, 1H), 2.35 (d, *J =* 14.4 Hz, 1H), 2.31 (s, 3H) ppm;^13^C NMR (150 MHz, CDCl_3_) δ: 152.9, 151.8, 148.8, 148.3, 147.3, 143.8, 142.0, 138.0, 135.9, 132.8, 131.3, 130.8, 129.2, 129.1, 128.1, 128.0, 128.0, 127.8, 126.5, 122.2, 122.1, 120.0, 116.2, 112.8, 106.2, 105.5, 63.9, 61.7, 60.5, 56.6, 56.4, 56.0, 56.0, 45.4, 43.9, 42.7, 42.1, 38.2, 35.4, 25.4, 22.0 ppm. HR-ESIMS: *m/z* 793.3154 [M+H]^+^, calculated for C_45_H_49_N_2_O_9_S: 793.3153.

Compound **3** 14-*O*-(2-fluorobenzenesulfonyl)-tetrandrine

White amorphous solid, yield 88.9%; m.p. : 137.6 ℃; ^1^H NMR (600 MHz, CDCl_3_) δ: 7.89-7.86 (m, 1H), 7.70-7.67 (m, 1H), 7.36 (dd, *J =* 8.4, 2.4 Hz, 1H), 7.31-7.28 (m, 2H), 7.07 (dd, *J =* 8.4, 2.4 Hz, 1H), 6.82 (dd, *J =* 8.4, 2.4 Hz, 1H), 6.53 (s, 1H), 6.52 (s, 2H), 6.34 (dd, *J =* 8.4, 2.4 Hz, 1H), 6.31 (s, 1H), 5.96 (s, 1H), 3.91 (dd, *J =* 10.8, 5.4 Hz, 1H), 3.76 (s, 3H), 3.72 (s, 4H), 3.56-3.51 (m, 1H), 3.46-3.41 (m, 1H), 3.37 (s, 3H), 3.28 (dd, *J =* 12.6, 5.4 Hz, 1H), 3.21 (s, 3H), 3.16 (dd, *J =* 13.8, 10.8 Hz, 1H), 2.99-2.86 (m, 3H), 2.85-2.73 (m, 3H), 2.64 (s, 3H), 2.40 (dd, *J =* 16.8, 5.4 Hz, 1H), 2.31 (d, *J =* 13.8 Hz, 1H), 2.26 (s, 3H) ppm; ^13^C NMR (150 MHz, CDCl_3_) δ: 159.6 (d, ^1^*J*_C-F_ =260.3 Hz), 152.6, 151.4, 148.6, 148.4, 147.9, 146.7, 143.5, 141.4, 137.7, 136.61 (d, ^3^*J*_C-F_ =8.3 Hz), 135.8, 132.6, 131.5, 130.5, 128.2, 127.7, 127.7, 127.4, 124.5, 124.5, 124.4, 122.1, 122.1, 122.0, 119.6, 117.51 (d, ^2^*J*_C-F_ =20.7 Hz), 116.0, 112.7, 106.0, 105.7, 63.7, 61.5, 60.3, 56.0, 55.9, 55.8, 45.2, 43.4, 42.6, 42.0, 37.9, 34.9, 25.3, 22.0 ppm. HR-ESIMS: *m/z* 797.2899 [M+H]^+^, calculated for C_44_H_46_FN_2_O_9_S: 797.2903.

Compound **4** 14-*O*-(3-fluorobenzenesulfonyl)-tetrandrine

White amorphous solid, yield 84.4%; m.p. : 119.4 ℃; ^1^H NMR (600 MHz, CDCl_3_) δ: 7.74 (ddd, *J =* 7.8, 1.8, 1.2 Hz, 1H), 7.67 (dt, *J =* 7.2, 2.4 Hz, 1H), 7.54 (td, *J =* 8.4, 5.4 Hz, 1H), 7.39-7.36 (m, 2H), 7.10 (dd, *J =* 7.8, 2.4 Hz, 1H), 6.82 (dd, *J =* 7.8, 2.4 Hz, 1H), 6.52 (s, 2H), 6.43 (s, 1H), 6.33 (dd, *J =* 8.4, 2.4 Hz, 1H), 6.31 (s, 1H), 5.96 (s, 1H), 3.91 (dd, *J =* 10.8, 5.4 Hz, 1H), 3.76 (s, 3H), 3.73 (s, 3H), 3.69 (d, *J =* 10.8 Hz, 1H), 3.53-3.48 (m, 1H), 3.46-3.42 (m, 1H), 3.37 (s, 3H), 3.29 (dd, *J =* 12.0, 5.4 Hz, 1H), 3.21 (s, 3H), 3.01-2.74 (m, 7H), 2.64 (s, 3H), 2.41 (dd, *J =* 16.8, 5.4 Hz, 1H), 2.27-2.25 (m, 4H) ppm; ^13^C NMR (150 MHz, CDCl_3_) δ: 162.2 (d, ^1^*J*_C-F_ =252.5 Hz), 152.6, 151.4, 148.6, 148.4, 147.9, 146.7, 143.5, 141.6, 138.1, 138.1 (d, ^3^*J*_C-F_ =7.3 Hz), 135.8, 132.7, 131.0 (d, ^3^*J*_C-F_ =7.5 Hz), 130.5, 128.1, 127.7, 127.6, 127.4, 124.4 (d, ^4^*J*_C-F_ =3.2 Hz), 122.0, 122.0, 121.4 (d, ^2^*J*_C-F_ =21.1 Hz), 119.6, 116.1, 115.9, 112.7, 106.0, 105.6, 63.8, 61.5, 60.3, 56.0, 55.9, 55.8, 45.3, 43.5, 42.6, 42.0, 38.0, 35.2, 25.2, 21.9 ppm. HR-ESIMS: *m/z* 797.2899 [M+H]^+^, calculated for C_44_H_46_FN_2_O_9_S: 797.2903.

Compound **5** 14-*O*-(4-fluorobenzenesulfonyl)-tetrandrine

White amorphous solid, yield 65.2%; m.p. : 140.9 ℃; ^1^H NMR (600 MHz, CDCl_3_) δ: 7.95-7.92 (m, 2H), 7.37 (dd, *J =* 8.4, 2.4 Hz, 1H), 7.23-7.20 (m, 2H), 7.09 (dd, *J =* 8.4, 2.4 Hz, 1H), 6.81 (dd, *J =* 8.4, 2.4 Hz, 1H), 6.52 (s, 1H), 6.50 (s, 1H), 6.45 (s, 1H), 6.33 (dd, *J =* 8.4, 2.4 Hz, 1H), 6.31 (s, 1H), 5.96 (s, 1H), 3.91 (dd, *J =* 10.8, 5.4 Hz, 1H), 3.76 (s, 3H), 3.74 (s, 3H), 3.67 (d, *J =* 10.2 Hz, 1H), 3.52-3.41 (m, 2H), 3.36 (s, 3H), 3.28 (dd, *J =* 12.6, 6.0 Hz, 1H), 3.21 (s, 3H), 3.00-2.73 (m, 7H), 2.64 (s, 3H), 2.40 (dd, *J =* 16.8, 5.4 Hz, 1H), 2.25-2.22 (m, 4H) ppm; ^13^C NMR (150 MHz, CDCl_3_) δ: 165.9 (d, ^1^*J*_C-F_ =257.4 Hz), 152.6, 151.4, 148.6, 148.4, 148.0, 146.7, 143.5, 141.5, 137.8, 135.9, 132.7, 132.1 (d, ^4^*J*_C-F_ =3.2 Hz), 131.5 (d, ^3^*J*_C-F_ =9.4 Hz), 130.5, 128.1, 127.7, 127.4, 122.0, 122.0, 119.6, 116.5 (d, ^2^*J*_C-F_ =22.9 Hz), 116.0, 112.7, 106.0, 105.8, 63.8, 61.5, 60.3, 56.1, 55.8, 55.8, 45.3, 43.5, 42.6, 42.0, 37.9, 35.1, 25.3, 21.8 ppm. HR-ESIMS: *m/z* 797.2902 [M+H]^+^, calculated for C_44_H_46_FN_2_O_9_S: 797.2903.

Compound **6** 14-*O*-(2-chlorobenzenesulfonyl)-tetrandrine

White amorphous solid, yield 79.1%; m.p. : 118.0 ℃; ^1^H NMR (600 MHz, CDCl_3_) δ: 8.01 (dd, *J =* 7.8, 1.2 Hz, 1H), 7.65 (dd, *J =* 8.4, 1.2 Hz, 1H), 7.61 (td, *J =* 7.2, 1.8 Hz, 1H), 7.42 (td, *J =* 7.8, 1.2 Hz, 1H), 7.36 (dd, *J =* 8.4, 2.4 Hz, 1H), 7.07 (dd, *J =* 8.4, 2.4 Hz, 1H), 6.82 (dd, *J =* 8.4, 2.4 Hz, 1H), 6.55 (s, 1H), 6.52 (s, 1H), 6.36*-*6.34 (m, 2H), 6.30 (s, 1H), 5.96 (s, 1H), 3.91 (dd, *J =* 10.8, 6.0 Hz, 1H), 3.76-3.74 (m, 4H), 3.64 (s, 3H), 3.55-3.50 (m, 1H), 3.45-3.41 (m, 1H), 3.37 (s, 3H), 3.28 (dd, *J =* 12.0, 5.4 Hz, 1H), 3.23-3.20 (m, 4H), 2.99*-*2.87 (m, 3H), 2.84*-*2.73 (m, 3H), 2.64 (s, 3H), 2.41*-*2.34 (m, 2H), 2.27 (s, 3H) ppm; ^13^C NMR (150 MHz, CDCl_3_) δ: 152.6, 151.4, 148.6, 148.4, 147.8, 146.7, 143.4, 141.5, 137.7, 135.9, 134.9, 134.6, 133.5, 132.6, 132.3, 132.2, 130.5, 128.1, 127.7, 127.6, 127.1, 122.2, 122.1, 122.0, 119.5, 116.1, 112.7, 106.0, 105.5, 63.7, 61.4, 60.4, 55.9, 55.9, 55.8, 45.2, 43.5, 42.6, 42.1, 38.0, 35.0, 25.2, 22.1 ppm. HR-ESIMS: *m/z* 813.2602 [M+H]^+^, calculated for C_44_H_46_ClN_2_O_9_S: 813.2607.

Compound **7** 14-*O*-(2-chlorobenzensulfonly)-tetrandrine

White amorphous solid, yield 85.8%; m.p. : 117.6 ℃; ^1^H NMR (600 MHz, CDCl_3_) δ: 7.94 (t, *J =* 1.8 Hz, 1H), 7.83 (ddd, *J =* 7.8, 1.8, 1.2 Hz, 1H), 7.64 (ddd, *J =* 7.8, 1.8, 1.2 Hz, 1H), 7.50 (t, *J =* 7.8 Hz, 1H), 7.37 (dd, *J =* 8.4, 2.4 Hz, 1H), 7.10 (dd, *J =* 8.4, 2.4 Hz, 1H), 6.82 (dd, *J =* 8.4, 2.4 Hz, 1H), 6.53 (s, 1H), 6.52 (s, 1H), 6.41 (s, 1H), 6.34 (dd, *J =* 8.4, 2.4 Hz, 1H), 6.31 (s, 1H), 5.96 (s, 1H), 3.93 (dd, *J =* 10.8, 6.0 Hz, 1H), 3.76 (s, 3H), 3.73 (s, 3H), 3.70 (d, *J =* 10.2 Hz, 1H), 3.52-3.42 (m, 2H), 3.37 (s, 3H), 3.29 (dd, *J =* 12.6, 5.4 Hz, 1H), 3.21 (s, 3H), 3.02-2.88 (m, 4H), 2.86-2.74 (m, 3H), 2.64 (s, 3H), 2.42 (dd, *J =* 16.8, 5.4 Hz, 1H), 2.28-2.26 (m, 4H) ppm; ^13^C NMR (150 MHz, CDCl_3_) δ: 152.6, 151.4, 148.6, 148.5, 147.9, 146.7, 143.5, 141.5, 137.9, 137.7, 135.8, 135.4, 134.2, 132.7, 130.5, 130.4, 128.6, 128.1, 127.6, 127.5, 127.4, 126.6, 122.0, 122.0, 122.0, 119.6, 116.1, 112.7, 106.0, 105.7, 63.7, 61.4, 60.3, 56.0, 55.9, 55.8, 45.1, 43.5, 42.4, 42.0, 37.9, 35.1, 25.1, 21.9 ppm. HR-ESIMS: *m/z* 813.2605 [M+H]^+^, calculated for C_44_H_46_ClN_2_O_9_S : 813.2607.

Compound **8** 14-*O*-(4-chlorobenzenesulfonyl)-tetrandrine

White amorphous solid, yield 65.7%; m.p. : 123.4 ℃; ^1^H NMR (600 MHz, CDCl_3_) δ: 7.85 (dt, *J =* 8.4, 2.4 Hz, 2H), 7.52 (d, *J =* 8.4, 2.4 Hz, 1H), 7.37 (dd, *J =* 8.4, 2.4 Hz, 1H), 7.09 (dd, *J =* 8.4, 2.4 Hz, 1H), 6.81 (dd, *J =* 8.4, 2.4 Hz, 1H), 6.52 (s, 1H), 6.51 (s, 1H), 6.44 (s, 1H), 6.33 (dd, *J =* 8.4, 2.4 Hz, 1H), 6.31 (s, 1H), 5.96 (s, 1H), 3.92 (dd, *J =* 10.8, 5.4 Hz, 1H), 3.76 (s, 3H), 3.74 (s, 3H), 3.68 (d, *J =* 10.2 Hz, 1H), 3.51-3.42 (m, 2H), 3.37 (s, 3H), 3.29 (dd, *J =* 12.1, 5.4 Hz, 1H), 3.21 (s, 3H), 3.01*-*2.73 (m, 8H), 2.64 (s, 3H), 2.40 (dd, *J =* 16.8, 5.4 Hz, 1H), 2.25-2.22 (m, 4H) ppm; ^13^C NMR (150 MHz, CDCl_3_) δ: 152.6, 151.4, 148.6, 148.4, 147.9, 146.7, 143.5, 141.5, 140.9, 137.7, 135.8, 134.6, 132.7, 130.5, 130.0, 129.4, 128.1, 127.6, 127.4, 122.0, 122.0, 119.6, 116.0, 112.7, 106.0, 105.7, 63.8, 61.5, 60.3, 56.1, 55.9, 55.8, 45.9, 45.2, 43.5, 42.5, 42.0, 38.0, 35.1, 25.2, 21.9 ppm. HR-ESIMS: *m/z* 813.2610 [M+H]^+^, calculated for C_44_H_46_ClN_2_O_9_S: 813.2607.

Compound **9** 14-*O*-(2-bromobenzenesulfonyl)-tetrandrine

light yellow amorphous solid, yield 88.2%; m.p. : 145.7 ℃; ^1^H NMR (600 MHz, CDCl_3_) δ: 8.03 (dd, *J =* 7.8, 1.8 Hz, 1H), 7.87 (dd, *J =* 7.8, 1.2 Hz, 1H), 7.51 (td, *J =* 7.8, 1.8 Hz, 1H), 7.46 (td, *J =* 7.8, 1.2 Hz, 1H), 7.36 (dd, *J =* 8.4, 2.4 Hz, 1H), 7.07 (dd, *J =* 8.4, 2.4 Hz, 1H), 6.82 (dd, *J =* 8.4, 2.4 Hz, 1H), 6.55 (s, 1H), 6.52 (s, 1H), 6.35 (dd, *J =* 8.4, 2.4 Hz, 1H), 6.33 (s, 1H), 6.30 (s, 1H), 5.96 (s, 1H), 3.91 (dd, *J =* 10.8, 5.4 Hz, 1H), 3.77-3.75 (m, 4H), 3.63 (s, 3H), 3.56-3.48 (m, 1H), 3.47-3.40 (m, 1H), 3.37 (s, 3H), 3.28 (dd, *J =* 12.1, 6.0 Hz, 1H), 3.25-3.21 (m, 4H), 3.00-2.88 (m, 3H), 2.84-2.73 (m, 3H), 2.64 (s, 3H), 2.41-2.35 (m, 2H), 2.27 (s, 3H) ppm; ^13^C NMR (151 MHz, CDCl_3_) δ: 152.6, 151.4, 148.6, 148.3, 147.8, 146.7, 143.4, 141.6, 137.7, 136.5, 135.8, 135.8, 134.8, 132.6, 132.5, 130.5, 128.1, 127.7, 127.6, 127.6, 122.2, 122.1, 122.0, 121.4, 119.5, 116.1, 112.7, 106.0, 105.5, 63.8, 61.4, 60.3, 55.9, 55.9, 55.8, 45.3, 43.5, 42.6, 42.1, 38.0, 35.0, 29.7, 25.2, 22.1 ppm. HR-ESIMS: *m/z* 857.2094 [M+H]^+^, calculated for C_44_H_46_BrN_2_O_9_S: 857.2102.

Compound **10** 14-*O*-(4-bromo-benzenesulfonyl)-tetrandrine

light yellow amorphous solid, yield 80.9%; m.p. : 119.1 ℃; ^1^H NMR (600 MHz, CDCl_3_) δ: 7.78 (dt, *J =* 8.4, 2.4 Hz, 2H), 7.68 (dt, *J =* 8.4, 2.4 Hz, 2H), 7.37 (dd, *J =* 8.4, 1.8 Hz, 1H), 7.09 (dd, *J =* 8.4, 2.4 Hz, 1H), 6.81 (dd, *J =* 8.4, 2.4 Hz, 1H), 6.52 (s, 1H), 6.51 (s, 1H), 6.43 (s, 1H), 6.33 (dd, *J =* 8.4, 1.8 Hz, 1H), 6.31 (s, 1H), 3.91 (dd, *J =* 10.8, 5.4 Hz, 1H), 3.76 (s, 3H), 3.74 (s, 3H), 3.67 (d, *J =* 10.8 Hz, 1H), 3.51-341 (m, 2H), 3.36 (s, 3H), 3.28 (dd, *J =* 12.6, 5.4 Hz, 1H), 3.21 (s, 3H), 3.01*-*2.72 (m, 8H), 2.64 (s, 3H), 2.40 (dd, *J =* 16.8, 5.4 Hz, 1H), 2.25-2.22 (m, 4H) ppm; ^13^C NMR (150 MHz, CDCl_3_) δ: 152.6, 151.4, 148.6, 148.4, 147.9, 146.7, 143.5, 141.5, 137.7, 135.8, 135.1, 132.7, 132.4, 130.5, 130.1, 129.4, 128.1, 127.6, 127.4, 122.0, 122.0, 119.6, 116.0, 112.7, 105.9, 105.7, 63.7, 61.5, 60.3, 56.1, 55.8, 55.8, 45.2, 43.5, 42.5, 42.0, 37.9, 35.2, 25.2, 21.8 ppm. HR-ESIMS: *m/z* 857.2100 [M+H]^+^, calculated for C_44_H_46_BrN_2_O_9_S: 857.2102.

Compound **11** 14-*O*-(3-nitrobenzenesulfonyl)-tetrandrine

Orange amorphous solid, yield 82.6%; m.p. : 124.0 ℃; ^1^H NMR (600 MHz, CDCl_3_) δ: 8.79 (t, *J =* 2.4 Hz, 1H), 8.51 (ddd, *J =* 7.8, 2.4, 1.2 Hz, 1H), 8.25 (ddd, *J =* 7.8, 2.4, 1.2 Hz, 1H), 7.77 (t, *J =* 7.8 Hz, 1H), 7.37 (dd, *J =* 7.8, 2.4 Hz, 1H), 7.09 (dd, *J =* 8.4, 2.4 Hz, 1H), 6.81 (dd, *J =* 8.4, 2.4 Hz, 1H), 6.57 (s, 1H), 6.51 (s, 2H), 6.33 (dd, *J =* 8.4, 2.4 Hz, 1H), 6.30 (s, 1H), 5.96 (s, 1H), 3.92 (dd, *J =* 10.8, 6.0 Hz, 1H), 3.78 (s, 3H), 3.76 (s, 3H), 3.66 (d, *J =* 10.2 Hz, 1H), 3.49-3.40 (m, 2H), 3.36 (s, 3H), 3.28 (dd, *J =* 12.6, 5.4 Hz, 1H), 3.20 (s, 3H), 3.00-2.72 (m, 7H), 2.64 (s, 3H), 2.38 (dd, *J =* 16.8, 5.4 Hz, 1H), 2.24 (s, 3H), 2.20 (d, *J =* 13.2 Hz, 1H) ppm; ^13^C NMR (150 MHz, CDCl_3_) δ: 152.5, 151.5, 148.7, 148.6, 148.2, 148.0, 146.9, 143.5, 141.3, 138.3, 137.8, 135.9, 133.9, 132.7, 130.6, 130.6, 128.5, 128.0, 127.7, 127.7, 127.2, 123.7, 122.0, 121.9, 121.8, 119.6, 116.2, 112.7, 106.0, 105.7, 63.7, 61.5, 60.3, 56.2, 55.8, 55.8, 45.2, 43.7, 42.6, 42.0, 37.9, 35.3, 25.3, 21.8 ppm. HR-ESIMS: *m/z* 824.2847 [M+H]^+^, calculated for C_44_H_46_N_3_O_11_S: 824.2848.

Compound **12** 14-*O*-(4-nitrobenzenesulfonyl)-tetrandrine

Orang amorphous solid, yield 84%; m.p. : 122.5 ℃; ^1^H NMR (600 MHz, CDCl_3_) δ: 8.37 (dt, *J =* 9.0, 2.4 Hz, 2H), 8.13 (dt, *J =* 9.0, 2.4 Hz, 2H), 7.37 (dd, *J =* 8.4, 2.4 Hz, 1H), 7.09 (dd, *J =* 8.4 , 2.4 Hz, 1H), 6.80 (dd, *J =* 8.4, 2.4 Hz, 1H), 6.59 (s, 1H), 6.52 (s, 1H), 6.50 (s, 1H), 6.32 (dd, *J =* 8.4 , 2.4 Hz, 1H), 6.31 (s, 1H), 5.96 (s, 1H), 3.91 (dd, *J =* 10.8, 5.4 Hz, 1H), 3.78 (s, 3H), 3.76 (s, 3H), 3.65 (d, *J =* 10.8 Hz, 1H), 3.46-3.41 (m, 2H), 3.36 (s, 3H), 3.28 (dd, *J =* 12.6, 5.4 Hz, 1H), 3.20 (s, 3H), 2.99-2.73 (m, 7H), 2.64 (s, 3H), 2.38 (dd, *J =* 16.8, 5.4 Hz, 1H), 2.25 (s, 3H), 2.19 (dd, *J =* 13.2 Hz, 1H) ppm; ^13^C NMR (151 MHz, CDCl_3_) δ: 152.5, 151.5, 150.9, 148.6, 148.6, 148.0, 146.9, 143.5, 141.8, 141.4, 137.8, 135.9, 132.7, 130.6, 129.9, 128.0, 127.7, 127.7, 127.1, 124.2, 122.0, 121.9, 121.8, 119.6, 116.1, 112.7, 105.9, 105.6, 63.7, 61.5, 60.3, 56.2, 55.8, 55.8, 45.2, 43.6, 42.5, 42.0, 37.9, 35.3, 25.2, 21.8 ppm. HR-ESIMS: *m/z* 824.2843 [M+H]^+^, calculated for C_44_H_46_N_3_O_11_S: 824.2848.

Compound **13** 14-*O*-(3-cyanobenzenesulfonyl)-tetrandrine

White amorphous solid, yield 86.9%; m.p. : 119.4 ℃; ^1^H NMR (600 MHz, CDCl_3_) δ: 8.24 (t, *J =* 1.8 Hz, 1H), 8.16 (dt, *J =* 7.8, 1.2, Hz, 1H), 7.94 (dt, *J =* 7.8, 1.2 Hz, 1H), 7.70 (t, *J =* 7.8 Hz, 1H), 7.37 (dd, *J =* 8.4, 2.4 Hz, 1H), 7.10 (dd, *J =* 8.4, 2.4 Hz, 1H), 6.81 (dd, *J =* 8.4, 2.4 Hz, 1H), 6.52 (s, 3H), 6.33 (dd, *J =* 8.4, 2.4 Hz, 1H), 6.31 (s, 1H), 5.97 (s, 1H), 3.93 (dd, *J =* 10.8, 5.4 Hz, 1H), 3.78 (s, 3H), 3.76 (s, 3H), 3.67 (d, *J =* 10.8 Hz, 1H), 3.47-3.41 (m, 2H), 3.36 (s, 3H), 3.29 (dd, *J =* 12.6, 5.4 Hz, 1H), 3.20 (s, 3H), 3.00-2.75 (m, 7H), 2.64 (s, 3H), 2.40 (dd, *J =* 16.8, 5.4 Hz, 1H), 2.25 (s, 3H), 2.21 (d, *J =* 13.2 Hz, 1H) ppm; ^13^C NMR (150 MHz, CDCl_3_) δ: 152.6, 151.5, 148.6, 148.6, 147.9, 146.9, 143.5, 141.3, 137.9, 137.8, 137.1, 135.8, 132.7, 132.3, 132.0, 130.6, 130.3, 128.0, 127.6, 127.5, 127.2, 122.0, 121.9, 121.8, 119.7, 116.7, 116.1, 113.9, 112.7, 106.0, 105.6, 63.6, 61.5, 60.3, 56.2, 55.8, 55.8, 45.1, 43.6, 42.4, 42.0, 37.9, 35.2, 25.1, 21.8 ppm. HR-ESIMS: *m/z* 804.2943 [M+H]^+^, calculated for C_45_H_46_N_3_O_9_S: 804.2949.

Compound **14** 14-*O*-(4-cyanobenzenesulfonyl)-tetrandrine

White amorphous solid, yield 86.8%; m.p. : 145.5 ℃; ^1^H NMR (600 MHz, CDCl_3_) δ: 8.06 (d, *J =* 8.4 Hz, 2H), 7.85 (d, *J =* 8.4 Hz, 2H), 7.39 (dd, *J =* 8.4, 2.4 Hz, 1H), 7.10 (dd, *J =* 8.4, 2.4 Hz, 1H), 6.82 (dd, *J =* 8.4, 2.4 Hz, 1H), 6.55 (s, 1H), 6.53 (s, 1H), 6.51 (s, 1H), 6.34 (dd, *J =* 8.4, 2.4 Hz, 1H), 6.33 (s, 1H), 5.98 (s, 1H), 3.94 (dd, *J =* 10.4, 5.4 Hz, 1H), 3.79 (s, 3H), 3.78 (s, 3H), 3.66 (d, *J =* 10.2 Hz, 1H), 3.48-3.41 (m, 2H), 3.37 (s, 3H), 3.30 (dd, *J =* 12.6, 5.4 Hz, 1H), 3.22 (s, 3H), 3.00-2.76 (m, 7H), 2.65 (s, 3H), 2.39 (dd, *J =* 16.8, 5.4 Hz, 1H), 2.26 (s, 3H), 2.21 (d, *J =* 13.2 Hz, 1H) ppm; ^13^C NMR (150 MHz, CDCl_3_) δ: 152.5, 151.5, 148.6, 148.6, 148.0, 146.8, 143.5, 141.4, 140.3, 137.8, 135.9, 132.8, 132.7, 130.6, 129.2, 128.0, 127.7, 127.5, 127.1, 122.0, 121.9, 121.8, 119.6, 117.8, 116.9, 116.1, 112.7, 105.9, 105.6, 63.7, 61.5, 60.3, 56.2, 55.8, 55.8, 45.1, 43.5, 42.4, 42.0, 37.9, 35.3, 25.1, 21.7 ppm. HR-ESIMS: *m/z* 804.2947 [M+H]^+^, calculated for C_45_H_46_N_3_O_9_S: 804.2949.

Compound **15** 14-*O*-(2-(trifluoromethyl)benzenesulfonyl)-tetrandrine

White amorphous solid, yield 72.5%; m.p. : 134.5 ℃; ^1^H NMR (600 MHz, CDCl_3_) δ: 8.21 (dd, *J =* 7.8, 1.2 Hz, 1H), 8.01 (dd, *J =* 7.8, 1.2 Hz, 1H), 7.82 (t, *J =* 7.8 Hz, 1H), 7.74 (t, *J =* 7.8 Hz, 1H), 7.37 (dd, *J =* 8.4, 1.8 Hz, 1H), 7.08 (dd, *J =* 8.4, 24 Hz, 1H), 6.82 (dd, *J =* 8.4, 2.4 Hz, 1H), 6.56 (s, 1H), 6.52 (s, 1H), 6.35 (dd, *J =* 8.4, 1.8 Hz, 1H), 6.34 (s, 1H), 6.30 (s, 1H), 5.97 (s, 1H), 3.92 (dd, *J =* 10.8, 5.4 Hz, 1H), 3.75 (s, 4H), 3.67 (s, 3H), 3.48-3.37 (m, 2H), 3.37 (s, 3H), 3.29 (dd, *J =* 12.6, 5.4 Hz, 1H), 3.21 (s, 3H), 3.14 (dd, *J =* 14.4, 10.8 Hz, 1H), 3.00-2.85 (m, 3H), 2.83-2.72 (m, 3H), 2.64 (s, 3H), 2.38-2.34 (m, 2H), 2.25 (s, 3H) ppm; ^13^C NMR (150 MHz, CDCl_3_) δ: 152.7, 151.4, 148.6, 148.4, 147.8, 146.7, 143.5, 141.6, 137.7, 135.8, 135.3, 134.1, 132.6, 132.6, 132.3, 130.5, 129.2 (q, ^2^*J*_C-F_ = 33.8 Hz), 128.7 (q, ^3^*J*_C-F_ = 6.1 Hz), 128.1, 127.6, 127.5, 122.3 (q, ^1^*J*_C-F_ = 272.9 Hz), 122.1, 122.1, 122.0, 121.4, 119.6, 116.1, 112.7, 106.0, 105.5, 63.7, 61.4, 60.4, 56.0, 55.9, 55.8, 45.2, 43.4, 42.5, 42.0, 38.0, 35.0, 25.2, 22.2 ppm. HR-ESIMS: *m/z* 847.2871 [M+H]^+^, calculated for C_45_H_46_F_3_N_2_O_9_S: 847.2871.

Compound **16** 14-*O*-(3-(trifluoromethyl)benzenesulfonyl)-tetrandrine

White amorphous solid, yield 79.5%; m.p. : 119.2 ℃; ^1^H NMR (600 MHz, CDCl_3_) δ: 8.20 (s, 1H), 8.14 (d, *J =* 8.4 Hz, 1H), 7.93 (d, *J =* 7.8 Hz, 1H), 7.72 (t, *J =* 7.8 Hz, 1H), 7.37 (dd, *J =* 8.4, 2.4 Hz, 1H), 7.09 (dd, *J =* 8.4, 2.4 Hz, 1H), 6.82 (dd, *J =* 8.4, 2.4 Hz, 1H), 6.52 (s, 1H), 6.52 (s, 1H), 6.41 (s, 1H), 6.34 (dd, *J =* 8.4, 2.4 Hz, 1H), 6.30 (s, 1H), 5.96 (s, 1H), 3.92 (dd, *J =* 10.8, 5.4 Hz, 1H), 3.76 (s, 3H), 3.72 (s, 3H), 3.67 (d, *J =* 10.8 Hz, 1H), 3.48-3.41 (m, 2H), 3.37 (s, 3H), 3.28 (dd, *J =* 12.6, 5.4 Hz, 1H), 3.20 (s, 3H), 2.99-2.87 (m, 4H), 2.84-2.73 (m, 3H), 2.64 (s, 3H), 2.38 (dd, *J =* 16.8, 4.8 Hz, 1H), 2.25 (s, 3H), 2.23 (d, *J =* 13.8 Hz, 1H) ppm; ^13^C NMR (150 MHz, CDCl_3_) δ: 152.6, 151.4, 148.6, 148.6, 147.9, 146.8, 143.5, 141.3, 137.8, 137.5, 135.9, 132.7, 131.9 (q, ^2^*J*_C-F_ = 34.0 Hz), 131.8, 130.7 (q, ^3^*J*_C-F_ = 3.5 Hz), 130.5, 130.0, 128.1, 127.7, 127.5, 125.7 (q, ^3^*J*_C-F_ = 3.9 Hz), 123.9, 122.0, 121.9, 121.2 (q, ^1^*J*_C-F_ = 271.7 Hz), 119.6, 116.1, 112.7, 106.0, 105.7, 63.7, 61.5, 60.3, 56.0, 55.9, 55.8, 45.3, 43.6, 42.6, 42.0, 37.9, 35.2, 25.3, 21.8 ppm. HR-ESIMS: *m/z* 847.2872 [M+H]^+^, calculated for C_45_H_46_F_3_N_2_O_9_S: 847.2871.

Compound **17** 14-*O*-(4-(trifluoromethyl)benzene-1-sulfonyl)-tetrandrine

White amorphous solid, yield 74.4%; m.p. : 130.1 ℃; ^1^H NMR (600 MHz, CDCl_3_) δ: 8.09 (d, *J =* 7.8 Hz, 2H), 7.86*-*7.82 (d, *J =* 8.4 Hz, 2H), 7.39 (dd, *J =* 8.4, 2.4 Hz, 1H), 7.10 (dd, *J =* 8.4, 2.4 Hz, 1H), 6.83 (dd, *J =* 8.4, 2.4 Hz, 1H), 6.53 (s, 2H), 6.41 (s, 1H), 6.35 (dd, *J =* 8.4, 1.8 Hz, 1H), 6.33 (s, 1H), 5.98 (s, 1H), 3.93 (dd, *J =* 10.8, 5.4 Hz, 1H), 3.77 (s, 3H), 3.72 (s, 3H), 3.69 (d, *J =* 10.8 Hz, 1H), 3.50*-*3.43 (m, 2H), 3.38 (s, 3H), 3.30 (dd, *J =* 12.6, 5.4 Hz, 1H), 3.22 (s, 3H), 3.00*-*2.75 (m, 7H), 2.65 (s, 3H), 2.39 (dd, *J =* 16.8, 5.4 Hz, 1H), 2.28*-*2.25 (m, 4H) ppm; ^13^C NMR (150 MHz, CDCl_3_) δ: 152.6, 151.5, 148.6, 148.5, 148.0, 146.7, 143.5, 141.4, 139.8, 137.8, 135.9, 135.7 (q, ^2^*J*_C-F_ = 33.2 Hz), 132.7, 130.6, 129.2, 128.1, 127.7, 127.3, 126.3(q, ^3^*J*_C-F_ = 3.4 Hz), 123.0 (q, ^1^*J*_C-F_ = 273.3 Hz), 122.0, 121.9, 120.3, 119.6, 116.1, 112.7, 105.9, 105.6, 63.7, 61.5, 60.3, 56.0, 55.9, 55.8, 45.2, 43.5, 42.6, 42.0, 38.0, 35.3, 25.3, 21.8 ppm. HR-ESIMS: *m/z* 847.2864 [M+H]^+^, calculated for C_45_H_46_F_3_N_2_O_9_S: 847.2871.

Compound **18** 14-*O*-(4-(Trifluoromethoxy)benzenesulfonyl)-tetrandrine

White amorphous solid, yield 83.6%; m.p. : 134.3 ℃; ^1^H NMR (600 MHz, CDCl_3_) δ: 7.99 (dt, *J =* 9.0, 3.0 Hz, 2H), 7.39*-*7.36 (m, 3H), 7.09 (dd, *J =* 8.4, 2.4 Hz, 1H), 6.82 (dd, *J =* 8.4, 2.4 Hz, 1H), 6.52 (s, 2H), 6.39 (s, 1H), 6.33 (dd, *J =* 8.4, 2.4 Hz, 1H), 6.31 (s, 1H), 5.96 (s, 1H), 3.91 (dd, *J =* 10.8, 5.4 Hz, 1H), 3.76 (s, 3H), 3.71 (s, 3H), 3.67 (d, *J =* 10.2 Hz, 1H), 3.50*-*3.41 (m, 2H), 3.36 (s, 3H), 3.28 (dd, *J =* 12.6, 5.4 Hz, 1H), 3.21 (s, 3H), 2.99*-*2.87 (m, 4H), 2.85*-*2.73 (m, 2H), 2.64 (s, 3H), 2.38 (dd, *J =* 16.8, 5.4 Hz, 1H), 2.26*-*2.23 (m, 4H) ppm; ^13^C NMR (150 MHz, CDCl_3_) δ: 153.2, 152.6, 151.4, 148.6, 148.4, 148.0, 146.7, 143.5, 141.5, 137.8, 135.9, 134.4, 132.7, 130.9, 130.5, 128.1, 127.7, 127.4, 122.0, 122.0, 121.9, 120.9, 120.2 (q, ^1^*J*_C-F_ = 258.5 Hz), 119.6, 116.1, 112.7, 105.9, 105.7, 63.7, 61.5, 60.3, 55.9, 55.9, 55.8, 45.2, 43.5, 42.6, 42.0, 38.0, 35.2, 25.3, 21.8 ppm. HR-ESIMS: *m/z* 863.2813 [M+H]^+^, calculated for C_45_H_46_F_3_N_2_O_10_S: 863.2819.

Compound **19** 14-*O*-(4-Toluenesulfonyl)-tetrandrine

White amorphous solid, yield 81.2%; m.p. : 140.9 ℃; ^1^H NMR (600 MHz, CDCl_3_) δ: 7.79 (dt, J = 8.4, 1.8 Hz, 2H), 7.36 (dd, *J =* 8.4, 2.4 Hz, 1H), 7.34-7.32 (m, 2H), 7.09 (dd, *J =* 8.4, 2.4 Hz, 1H), 6.82 (dd, *J =* 8.4, 2.4 Hz, 1H), 6.52 (s, 1H), 6.51 (s, 1H), 6.36 (s, 1H), 6.33 (dd, *J =* 8.4, 2.4 Hz, 1H), 6.31 (s, 1H), 5.95 (s, 1H), 3.91 (dd, *J =* 10.8, 5.4 Hz, 1H), 3.76 (s, 3H), 3.71-3.68 (m, 4H), 3.56-3.51 (m, 1H), 3.45-3.41 (m, 1H), 3.37 (s, 3H), 3.28 (dd, *J =* 12.6, 5.4 Hz, 1H), 3.21 (s, 3H), 3.04 (dd, *J =* 14.4, 10.8 Hz, 1H), 2.97-2.84 (m, 4H), 2.81-2.73 (m, 2H), 2.64 (s, 3H), 2.44-2.40 (m, 4H), 2.26-2.23 (m, 4H) ppm; ^13^C NMR (150 MHz, CDCl_3_) δ: 152.7, 151.4, 148.6, 148.2, 147.9, 146.5, 145.2, 143.5, 141.7, 137.7, 135.8, 133.2, 132.6, 130.5, 129.7, 128.7, 128.1, 127.7, 127.6, 127.5, 122.2, 122.1, 122.0, 119.6, 115.9, 112.7, 106.0, 106.0, 63.8, 61.5, 60.3, 55.9, 55.9, 55.8, 45.2, 43.5, 42.6, 42.0, 37.9, 35.0, 25.3, 21.9, 21.7 ppm. HR-ESIMS: *m/z* 793.3151 [M+H]^+^, calculated for C_45_H_49_N_2_O_9_S: 793.3153.

Compound **20** 14-*O*-(4-methoxybenzenesulfonyl)-tetrandrine

White amorphous solid, yield 79.8%; m.p. : 132.5 ℃; ^1^H NMR (600 MHz, CDCl_3_) δ: 7.83 (dt, *J =* 9.0,3.0 Hz, 2H), 7.36 (dd, *J =* 8.4, 2.4 Hz, 1H), 7.09 (dd, *J =* 8.4, 2.4 Hz, 1H), 6.98 (dt, *J =* 9.0, 3.0 Hz, 2H), 6.82 (dd, *J =* 8.4, 2.4 Hz, 1H), 6.52 (s, 1H), 6.50 (s, 1H), 6.41 (s, 1H), 6.33 (dd, *J =* 8.4, 2.4 Hz, 1H), 6.31 (s, 1H), 5.95 (s, 1H), 3.91 (dd, *J =* 10.8, 5.4 Hz, 1H), 3.87 (s, 3H), 3.76 (s, 3H), 3.71-3.69 (m, 4H), 3.57-3.52 (m, 1H), 3.45-3.41 (m, 1H), 3.37 (s, 3H), 3.28 (dd, *J =* 12.6, 5.4 Hz, 1H), 3.21 (s, 3H), 3.03 (dd, *J =* 14.4, 10.8 Hz, 1H), 2.99-2.85 (m, 4H), 2.81-2.73 (m, 2H), 2.64 (s, 3H), 2.42 (dd, *J =* 16.8, 5.4 Hz, 1H), 2.26-2.23 (m, 4H) ppm; ^13^C NMR (150 MHz, CDCl_3_) δ: 164.0, 152.7, 151.4, 148.6, 148.2, 147.9, 146.5, 143.5, 141.7, 137.7, 135.8, 132.6, 130.9, 130.5, 128.1, 127.7, 127.7, 127.5, 127.5, 122.2, 122.1, 122.0, 119.6, 115.9, 114.2, 112.7, 106.0, 105.9, 63.8, 61.5, 60.3, 56.0, 55.9, 55.8, 55.8, 45.2, 43.5, 42.6, 42.0, 38.0, 35.0, 25.3, 21.9 ppm. HR-ESIMS: *m/z* 809.3101 [M+H]^+^, calculated for C_45_H_49_N_2_O_10_S: 809.3102.

Compound **21** 14-*O*-(4-tert-butylbenzenesulfonyl)-tetrandrine

Beige amorphous solid, yield 73%; m.p. : 122.4 ℃; ^1^H NMR (600 MHz, CDCl_3_) δ: 7.83 (dt, *J =* 8.4, 2.4 Hz, 2H), 7.54 (dt, *J =* 9.0, 2.4 Hz, 2H), 7.37 (dd, *J =* 8.4, 2.4 Hz, 1H), 7.09 (dd, *J =* 8.4, 2.4 Hz, 1H), 6.82 (dd, *J =* 8.4, 2.4 Hz, 1H), 6.51 (m, 2H), 6.34 (dd, *J =* 8.4, 2.4 Hz, 1H), 6.30 (s, 1H), 6.27 (s, 1H), 5.94 (s, 1H), 3.91 (dd, *J =* 10.8, 5.4 Hz, 1H), 3.76 (s, 3H), 3.70 (d, *J =* 9.6 Hz, 1H), 3.64 (s, 3H), 3.52-3.41 (m, 2H), 3.37 (s, 3H), 3.29 (dd, *J =* 12.6, 5.4 Hz, 1H), 3.20 (s, 3H), 2.99-2.83 (m, 5H), 2.81-2.73 (m, 2H), 2.64 (s, 3H), 2.40 (dd, *J =* 16.8, 5.4 Hz, 1H), 2.26 (s, 3H), 2.20 (d, *J =* 14.4 Hz, 1H), 1.32 (s, 9H) ppm; ^13^C NMR (150 MHz, CDCl_3_) δ: 158.3, 152.7, 151.4, 148.6, 148.2, 147.8, 146.5, 143.4, 141.7, 137.7, 135.8, 133.1, 132.6, 130.5, 128.6, 128.0, 127.6, 127.4, 126.1, 122.1, 122.0, 119.6, 115.9, 112.7, 106.2, 105.9, 63.8, 61.4, 55.9, 55.8, 55.8, 45.2, 43.5, 42.5, 42.0, 38.0, 35.3, 34.9, 31.0, 25.2, 22.0 ppm. HR-ESIMS: *m/z* 835.3624 [M+H]^+^, calculated for C_48_H_55_N_2_O_9_S: 835.3623.

Compound **22** 14-*O*-(4-acetamidobenzenesulfonyl)-tetrandrine

light yellow amorphous solid, yield 81.5%; m.p. : 158.4 ℃; ^1^H NMR (600 MHz, CDCl_3_) δ: 8.15 (s, 1H), 7.79 (d, *J =* 9.0 Hz, 2H), 7.71 (d, *J =* 9.0 Hz, 2H), 7.33 (dd, *J =* 8.4, 2.4 Hz, 1H), 7.07 (dd, *J =* 8.4, 2.4 Hz, 1H), 6.80 (dd, *J =* 8.4, 2.4 Hz, 1H), 6.51 (s, 1H), 6.50 (s, 1H), 6.45 (s, 1H), 6.33-6.32 (m, 2H), 5.95 (s, 1H), 3.90 (dd, *J =* 10.8, 5.4 Hz, 1H), 3.75 (s, 3H), 3.70-3.69 (m, 4H), 3.59-3.54 (m, 1H), 3.44-3.39 (m, 1H), 3.36 (s, 3H), 3.26 (dd, *J =* 12.6, 5.4 Hz, 1H), 3.21 (s, 3H), 3.02-2.86 (m, 5H), 2.81-2.71 (m, 2H), 2.63 (s, 3H), 2.45-2.41 (m, 1H), 2.26 (s, 3H), 2.21 (d, *J =* 14.4 Hz, 1H), 2.14 (s, 3H) ppm; ^13^C NMR (150 MHz, CDCl_3_) δ: 169.1, 152.6, 151.4, 148.5, 148.3, 147.9, 146.7, 143.6, 143.4, 141.7, 137.7, 135.9, 132.7, 130.5, 130.0, 129.8, 128.0, 127.9, 127.8, 127.3, 122.0, 122.0, 119.6, 119.0, 115.9, 112.7, 106.0, 106.0, 63.7, 61.5, 60.4, 56.1, 55.8, 45.2, 43.5, 42.6, 41.9, 37.7, 35.1, 29.7, 25.4, 24.6, 21.8 ppm. HR-ESIMS: *m/z* 836.3205 [M+H]^+^, calculated for C_46_H_50_N_3_O_10_S: 836.3211.

Compound **23** 14-*O*-(biphenyl-4-sulfonyl)-tetrandrine

White amorphous solid, yield 82.2%; m.p. : 148.7 ℃; ^1^H NMR (600 MHz, CDCl_3_) δ: 7.97 (d, *J =* 8.4 Hz, 2H), 7.74 (d, *J =* 8.4 Hz, 2H), 7.59-7.57 (m, 2H), 7.50-7.48 (m, 2H), 7.45-7.43 (m, 1H), 7.37 (dd, *J =* 8.4, 2.4 Hz, 1H), 7.09 (dd, *J =* 8.4, 2.4 Hz, 1H), 6.82 (dd, *J =* 8.4, 2.4 Hz, 1H), 6.52 (s, 1H), 6.51 (s, 1H), 6.42 (s, 1H), 6.33 (dd, *J* = 8.3, 2.2 Hz, 1H), 6.31 (s, 1H), 5.95 (s·, 1H), 3.92 (dd, *J* = 10.8, 5.4 Hz, 1H), 3.76 (s, 3H), 3.71 (d, *J =* 11.4 Hz, 1H), 3.69 (s, 3H), 3.57-3.51 (m, 1H), 3.48-3.43 (m, 1H), 3.37 (s, 3H), 3.30 (dd, *J =* 12.6, 5.4 Hz, 1H), 3.19 (s, 3H), 3.02 (dd, *J =* 14.4, 10.8 Hz, 1H), 2.99-2.87 (m, 4H), 2.81-2.75 (m, 2H), 2.64 (s, 3H), 2.42 (dd, *J =* 16.8, 6.0 Hz, 1H), 2.27 (s, 3H), 2.24 (d, *J =* 14.4 Hz, 1H) ppm; ^13^C NMR (150 MHz, CDCl_3_) δ: 152.7, 151.4, 148.7, 148.3, 147.8, 147.1, 146.6, 143.5, 141.7, 138.9, 137.7, 135.6, 135.4, 134.6, 132.6, 130.5, 129.2, 129.2, 128.9, 128.0, 127.6, 127.4, 127.3, 122.1, 122.1, 122.0, 119.6, 115.9, 112.7, 106.0, 105.9, 63.7, 61.4, 60.3, 56.0, 55.8, 55.8, 45.0, 43.5, 42.3, 42.0, 38.0, 35.0, 29.7, 25.0, 22.0 ppm. HR-ESIMS: *m/z* 855.3310 [M+H]^+^, calculated for C_50_H_51_N_2_O_9_S: 855.3309.

Compound **24** 14-*O*-(2-naphthalenesulfonyl)-tetrandrine

light yellow amorphous solid, yield 71.1%; m.p. : 130.0 ℃; ^1^H NMR (600 MHz, CDCl_3_) δ: 8.45 (d, *J =* 1.2 Hz, 1H), 8.01 (d, *J =* 9.0 Hz, 1H), 7.96*-*7.93 (m, 3H), 7.71-7.68 (m, 1H), 7.66-7.63 (m, 1H), 7.36 (dd, *J =* 8.4, 2.4 Hz, 1H), 7.08 (dd, *J =* 8.4, 2.4 Hz, 1H), 6.80 (dd, *J =* 8.4, 2.4 Hz, 1H), 6.52 (s, 1H), 6.51 (s, 1H), 6.35 (dd, *J =* 8.4, 2.4 Hz, 1H), 6.29 (s, 1H), 6.25 (s, 1H), 5.94 (s, 1H), 3.90 (dd, *J =* 10.8, 5.4 Hz, 1H), 3.76 (s, 3H), 3.71 (d, *J =* 10.8 Hz, 1H), 3.59-3.54 (m, 1H), 3.46 (s, 3H), 3.44*-*3.41 (m, 1H), 3.36 (s, 3H), 3.27 (dd, *J =* 12.6, 5.4 Hz, 1H), 3.20 (s, 3H), 3.13 (dd, *J =* 14.4, 10.8 Hz, 1H), 2.98-2.83 (m, 4H), 2.80-2.73 (m, 2H), 2.64 (s, 3H), 2.37 (dd, *J =* 16.8, 6.0 Hz, 1H), 2.28 (d, *J =* 13.8 Hz, 1H), 2.26 (s, 3H) ppm; ^13^C NMR (150 MHz, CDCl_3_) δ: 152.6, 151.4, 148.6, 148.3, 147.8, 146.5, 143.4, 141.7, 137.7, 135.8, 135.3, 133.0, 132.6, 131.8, 130.6, 130.5, 129.6, 129.5, 129.4, 128.1, 128.0, 127.9, 127.6, 127.6, 127.6, 123.1, 122.1, 122.0, 119.6, 116.0, 112.7, 105.9, 105.8, 63.7, 61.5, 60.3, 55.9, 55.8, 55.8, 45.2, 43.5, 42.5, 42.0, 38.0, 35.1, 25.2, 22.0 ppm. HR-ESIMS: *m/z* 829.3149 [M+H]^+^, calculated for C_48_H_49_N_2_O_9_S: 829.3153.

Compound **25** 14-*O*-(8-quinolinesulfonyl)-tetrandrine

light yellow amorphous solid, yield 77.4%; m.p. : 169.9 ℃; ^1^H NMR (600 MHz, CDCl_3_) δ: 9.20 (dd, *J =* 4.2, 1.8 Hz, 1H), 8.48 (dd, *J =* 7.2, 1.2 Hz, 1H), 8.30 (dd, *J =* 8.4, 1.8 Hz, 1H), 8.17 (dd, *J =* 8.4, 1.2 Hz, 1H), 7.65 (t, *J =* 7.8 Hz, 1H), 7.59 (dd, *J =* 8.4, 4.2 Hz, 1H), 7.35 (dd, *J =* 7.8, 1.8 Hz, 1H), 7.05 (dd, *J =* 8.4, 2.4 Hz, 1H), 6.81 (dd, *J =* 8.4, 2.4 Hz, 1H), 6.53 (s, 1H), 6.51 (s, 1H), 6.46 (s, 1H), 6.34 (dd, *J =* 8.4, 2.4 Hz, 1H), 6.29 (s, 1H), 5.95 (s, 1H), 3.91 (dd, *J =* 10.8, 5.4 Hz, 1H), 3.79 (d, *J =* 10.2 Hz, 1H), 3.76 (s, 3H), 3.54 (s, 3H), 3.52-3.47 (m, 1H), 3.45-3.40 (m, 1H), 3.37 (s, 3H), 3.35-3.32 (m, 1H), 3.27 (dd, *J =* 12.6, 5.4 Hz, 1H), 3.21 (s, 3H), 2.98-2.85 (m, 3H), 2.80-2.72 (m, 3H), 2.63 (s, 3H), 2.35 (dd, *J =* 16.8, 5.4 Hz, 1H), 2.28 (d, *J =* 14.4 Hz, 1H), 2.25 (s, 3H) ppm; ^13^C NMR (150 MHz, CDCl_3_) δ: 152.7, 152.4, 151.4, 148.5, 148.2, 147.8, 146.7, 144.1, 143.5, 141.8, 137.8, 136.6, 135.8, 135.1, 133.8, 133.7, 132.6, 130.5, 129.0, 127.9, 127.7, 127.7, 127.4, 125.3, 122.6, 122.2, 122.1, 121.9, 119.6, 115.9, 112.7, 106.1, 105.9, 63.7, 61.4, 60.3, 55.9, 55.8, 55.8, 45.2, 43.4, 42.5, 41.9, 37.9, 34.7, 25.2, 22.4 ppm. HR-ESIMS: *m/z* 830.3106 [M+H]^+^, calculated for C_47_H_48_N_3_O_9_S: 830.3106.

Compound **26** 14-*O*-(3,5-dichlorobenzenesulfonyl)-tetrandrine

White amorphous solid, yield 77.5%; m.p. : 114.7 ℃; ^1^H NMR (600 MHz, CDCl_3_) δ: 7.85 (d, *J* = 1.8 Hz, 2H), 7.65 (t, *J* = 1.8 Hz, 1H), 7.37 (dd, *J =* 8.4, 1.8 Hz, 1H), 7.11 (dd, *J =* 8.4, 3.0 Hz, 1H), 6.82 (dd, *J =* 8.4, 3.0 Hz, 1H), 6.55 (s, 1H), 6.52 (s, 1H), 6.45 (s, 1H), 6.34 (dd, *J =* 8.4, 1.8 Hz, 1H), 6.32 (s, 1H), 5.97 (s, 1H), 3.92 (dd, *J =* 10.8, 5.4 Hz, 1H), 3.78 (s, 3H), 3.76 (s, 3H), 3.69 (d, *J =* 10.8 Hz, 1H), 3.51-3.41 (m, 2H), 3.37 (s, 3H), 3.28 (dd, *J =* 12.0, 5.4 Hz, 1H), 3.21 (s, 3H), 3.01-2.88 (m, 4H), 2.86-2.72 (m, 3H), 2.64 (s, 3H), 2.42 (dd, *J =* 16.8, 5.4 Hz, 1H), 2.29 (m, 1H), 2.26 (s, 3H) ppm; ^13^C NMR (150 MHz, CDCl_3_) δ: 152.6, 151.5, 148.6, 148.6, 148.0, 146.9, 143.5, 141.3, 139.1, 137.8, 136.2, 135.9, 134.0, 132.7, 130.6, 128.1, 127.8, 127.5, 126.8, 122.0, 122.0, 122.0, 119.7, 116.3, 112.7, 106.0, 105.5, 63.7, 61.5, 60.4, 56.1, 55.9, 55.8, 45.2, 43.7, 42.6, 42.1, 38.0, 35.3, 25.3, 21.9 ppm. HR-ESIMS: *m/z* 847.2218 [M + H]^+^, calculated for C_44_H_45_Cl_2_N_2_O_9_S: 847.2217.

Compound **27** 14-*O*-(2,4-dichlorobenzenesulfonyl)-tetrandrine

White amorphous solid, yield 88.7%; m.p. : 128.2 ℃; ^1^H NMR (600 MHz, CDCl_3_) δ: 7.95 (d, *J =* 8.4 Hz, 1H), 7.66 (d, *J =* 2.4 Hz, 1H), 7.40 (dd, *J =* 8.4, 2.4 Hz, 1H), 7.37 (dd, *J =* 8.4, 2.4 Hz, 1H), 7.07 (dd, *J =* 8.4, 2.4 Hz, 1H), 6.83 (dd, *J =* 8.4, 2.4 Hz, 1H), 6.55 (s, 1H), 6.52 (s, 1H), 6.41 (s, 1H), 6.35 (dd, *J =* 8.4, 2.4 Hz, 1H), 6.31 (s, 1H), 5.96 (s, 1H), 3.94 (dd, *J =* 10.8, 5.4 Hz, 1H), 3.76 (s, 3H), 3.71 (s, 3H), 3.52-3.44 (m, 2H), 3.38 (s, 3H), 3.32 (dd, *J =* 12.6, 5.4 Hz, 1H), 3.21 (s, 3H), 3.18 (dd, *J =* 14.4, 10.2 Hz, 1H), 3.00-2.90 (m, 3H), 2.85-2.76 (m, 3H), 2.66 (s, 3H), 2.40 (dd, *J =* 16.8, 5.4 Hz, 2H), 2.35 (d, *J =* 14.4 Hz, 1H), 2.28 (s, 3H) ppm; ^13^C NMR (150 MHz, CDCl_3_) δ: 152.7, 151.5, 148.7, 148.5, 147.8, 146.8, 143.5, 141.4, 141.0, 137.8, 135.7, 134.5, 133.2, 133.1, 132.6, 132.0, 130.6, 128.1, 127.4, 127.3, 122.1, 122.0, 119.6, 116.1, 112.7, 106.0, 105.6, 63.7, 61.4, 60.4, 56.1, 55.9, 55.8, 45.1, 43.5, 42.4, 42.0, 38.0, 35.0, 25.0, 22.1 ppm. HR-ESIMS: *m/z* 847.2216 [M+H]^+^, calculated for C_44_H_46_FN_2_O_9_S: 847.2217.

Compound **28** 14-*O*-(2,4,6-trimethylbenzenesulfonyl)-tetrandrine

White amorphous solid, yield 68.6%; m.p. : 121.9 ℃; ^1^H NMR (600 MHz, CDCl_3_) δ: 7.37 (dd, *J =* 8.4, 2.4 Hz, 1H), 7.07 (dd, *J =* 8.4, 2.4 Hz, 1H), 7.02 (s, 2H), 6.83 (dd, *J =* 8.4, 2.4 Hz, 1H), 6.56 (s, 1H), 6.52 (s, 1H), 6.36 (dd, *J =* 8.4, 2.4 Hz, 1H), 6.31 (s, 1H), 5.98 (s, 1H), 5.96 (s, 1H), 3.92 (dd, *J =* 10.8, 6.0 Hz, 1H), 3.79-3.77 (m, 1H), 3.76 (s, 3H), 3.62-3.57 (m, 1H), 3.53 (s, 3H), 3.46-3.42 (m, 1H), 3.38 (s, 3H), 3.31-3.25 (m, 2H), 3.21 (s, 3H), 3.00*-*2.85 (m, 4H), 2.81*-*2.74 (m, 2H), 2.64 (s, 3H), 2.59 (s, 6H), 2.44 (dd, *J =* 16.8, 6.0 Hz, 1H), 2.39 (d, *J =* 14.4 Hz, 1H), 2.34 (s, 3H), 2.29 (s, 3H) ppm; ^13^C NMR (150 MHz, CDCl_3_) δ: 152.7, 151.4, 148.6, 148.0, 147.7, 146.5, 143.7, 143.4, 141.7, 140.5, 137.7, 135.8, 132.6, 131.8, 131.7, 130.5, 127.6, 127.6, 122.2, 122.0, 119.5, 116.0, 112.7, 106.0, 105.2, 63.8, 61.4, 60.4, 55.9, 55.8, 55.7, 45.2, 43.6, 42.5, 42.1, 38.0, 34.9, 25.2, 22.9, 22.2, 21.1 ppm. HR-ESIMS: *m/z* 821.3468 [M+H]^+^, calculated for C_47_H_53_N_2_O_9_S: 821.3466.

Compound **29** 14-*O*-(2,4,6-triisopropylbenzenesulfonyl )-tetrandrine

light yellow amorphous solid, yield 55.9%; m.p. : 132.6 ℃; ^1^H NMR (600 MHz, CDCl_3_) δ: 7.36 (dd, *J =* 7.8, 2.4 Hz, 1H), 7.24 (s, 2H), 7.01 (dd, *J =* 8.4, 2.4 Hz, 1H), 6.83 (dd, *J =* 8.4, 2.4 Hz, 1H), 6.56 (s, 1H), 6.52 (s, 1H), 6.37 (dd, *J =* 8.4, 2.4 Hz, 1H), 6.29 (s, 1H), 5.98 (s, 2H), 4.07 (p, *J =* 6.6 Hz, 2H), 3.92 (dd, *J =* 10.8, 5.4 Hz, 1H), 3.81 (d, *J =* 10.2 Hz, 1H), 3.75 (s, 3H), 3.56*-*3.48 (m, 1H), 3.47 (s, 3H), 3.45*-*3.41 (m, 1H), 3.39 (s, 3H), 3.34*-*3.27 (m, 2H), 3.20 (s, 3H), 2.97*-*2.89 (m, 4H), 2.81*-*2.74(m, 3H), 2.64 (s, 3H), 2.41 (m 2H), 2.28 (s, 3H), 1.27 (t, *J =* 6.6 Hz, 12H), 1.16 (d, *J =* 6.6 Hz, 6H) ppm; ^13^C NMR (150 MHz, CDCl_3_) δ: 154.2, 152.8, 151.4, 151.2, 148.7, 148.1, 147.6, 146.5, 143.5, 141.8, 137.7, 135.8, 132.6, 131.0, 130.5, 128.0, 127.6, 123.9, 122.2, 121.9, 119.6, 116.1, 112.7, 106.0, 105.5, 63.7, 61.2, 60.4, 55.9, 55.9, 55.5, 45.2, 43.6, 42.5, 42.1, 38.1, 34.7, 34.4, 29.9, 29.7, 25.2, 24.8, 24.5, 23.7, 23.6, 22.5 ppm. HR-ESIMS: *m/z* 905.4410 [M+H]^+^, calculated for C_53_H_65_N_2_O_9_S: 905.4405.

Compound **30** 14-*O*-(pyridine-3-sulfonyl)-tetrandrine

White amorphous solid, yield 59.6%; m.p. : 129.2 ℃; ^1^H NMR (600 MHz, CDCl_3_) δ: 9.12 (dd, *J =* 2.4, 1.2 Hz, 1H), 8.88 (dd, *J =* 4.8, 1.8 Hz, 1H), 8.18 (dt, *J =* 7.8, 1.8 Hz, 1H), 7.49 (ddd, *J =* 8.4, 4.8, 1.2 Hz, 1H), 7.37 (dd, *J =* 8.4, 2.4 Hz, 1H), 7.09 (dd, *J =* 8.4, 2.4 Hz, 1H), 6.81 (dd, *J =* 8.4, 2.4 Hz, 1H), 6.53 (s, 1H), 6.52 (s, 1H), 6.50 (s, 1H), 6.32 (dd, *J =* 8.4, 2.4 Hz, 1H), 6.31 (s, 1H), 5.95 (s, 1H), 3.92 (dd, *J =* 10.8, 5.4 Hz, 1H), 3.76 (s, 3H), 3.76 (s, 3H), 3.66 (d, *J =* 10.2 Hz, 1H), 3.50-3.42 (m, 2H), 3.36 (s, 3H), 3.29 (dd, *J =* 12.6, 5.4 Hz, 1H), 3.20 (s, 3H), 2.99-2.84 (m, 5H), 2.82-2.74 (m, 2H), 2.64 (s, 3H), 2.40 (dd, *J =* 16.8, 5.4 Hz, 1H), 2.25 (s, 3H), 2.21 (d, *J =* 13.8 Hz, 1H) ppm; ^13^C NMR (151 MHz, CDCl_3_) δ: 154.5, 152.6, 151.5, 149.2, 148.6, 148.5, 148.0, 146.8, 143.5, 141.3, 137.8, 136.1, 135.8, 132.9, 132.7, 130.5, 128.1, 127.7, 127.6, 127.2, 123.6, 122.0, 121.9, 121.9, 119.6, 116.1, 112.7, 106.0, 105.7, 63.7, 61.5, 60.3, 56.1, 55.9, 55.8, 45.2, 43.5, 42.5, 42.0, 37.9, 35.2, 25.2, 21.8 ppm. HR-ESIMS: *m/z* 780.2950 [M+H]^+^, calculated for C_43_H_46_N_3_O_9_S: 780.2949.

Compound **31** 14-*O*-(2-chloropyridine-5-sulfonyl)-tetrandrine

light yellow amorphous solid, yield 75.9%; m.p. : 116.6 ℃; ^1^H NMR (600 MHz, CDCl_3_) δ: 8.88 (dd, *J =* 2.4, 0.6 Hz, 1H), 8.12 (dd, *J =* 8.4, 2.4 Hz, 1H), 7.49 (dd, *J =* 8.4, 0.6 Hz, 1H), 7.37 (dd, *J =* 8.4, 2.4 Hz, 1H), 7.09 (dd, *J =* 8.4, 2.4 Hz, 1H), 6.81 (dd, *J =* 8.4, 2.4 Hz, 1H), 6.61 (s, 1H), 6.52 (s, 1H), 6.51 (s, 1H), 6.34*-*6.31 (m, 2H), 5.97 (s, 1H), 3.91 (dd, *J =* 10.8, 5.8 Hz, 1H), 3.81 (s, 3H), 3.76 (s, 3H), 3.65 (d, *J =* 10.2 Hz, 1H), 3.48*-*3.40 (m, 2H), 3.36 (s, 3H), 3.28 (dd, *J =* 12.6, 5.4 Hz, 1H), 3.20 (s, 3H), 2.99*-*2.72 (m, 7H), 2.64 (s, 3H), 2.40 (dd, *J =* 16.8z, 5.4 Hz, 1H), 2.25 (s, 3H), 2.23*-*2.19 (m, 1H) ppm; ^13^C NMR (150 MHz, CDCl_3_) δ: 156.9, 152.5, 151.5, 149.5, 148.6, 148.5, 148.0, 146.9, 143.5, 141.2, 138.5, 137.8, 135.9, 132.7, 131.9, 130.6, 128.1, 127.8, 127.7, 127.0, 124.7, 121.9, 121.9, 121.8, 119.6, 116.2, 112.7, 106.0, 105.5, 63.7, 61.5, 60.3, 56.2, 55.8, 55.8, 45.2, 43.6, 42.6, 42.0, 37.9, 35.3, 25.3, 21.7 ppm. HR-ESIMS: *m/z* 814.2552 [M+H]^+^, calculated for C_43_H_45_ClN_3_O_9_S: 814.2560.

Compound **32** 14-*O*-(2-thiophenesulfonyl)-tetrandrine

White amorphous solid, yield 61.8%; m.p. : 142.5 ℃; ^1^H NMR (600 MHz, CDCl_3_) δ: 7.72 (dd, *J =* 4.8, 1.2 Hz, 1H), 7.65 (dd, *J =* 3.6, 1.2 Hz, 1H), 7.36 (dd, *J =* 8.4, 2.4 Hz, 1H), 7.12 (dd, *J =* 5.4, 4.2 Hz, 1H), 7.09 (dd, *J =* 8.4, 2.4 Hz, 1H), 6.82 (dd, *J =* 8.4, 2.4 Hz, 1H), 6.52 (s, 1H), 6.51 (s, 2H), 6.33 (dd, *J =* 8.4, 2.4 Hz, 1H), 6.31 (s, 1H), 5.95 (s, 1H), 3.91 (dd, *J =* 10.8, 5.4 Hz, 1H), 3.76 (s, 6H), 3.69 (d, *J =* 10.2 Hz, 1H), 3.61-3.55 (m, 1H), 3.46-3.41 (m, 1H), 3.37 (s, 3H), 3.28 (dd, *J =* 12.6, 5.4 Hz, 1H), 3.20 (s, 3H), 3.01 (dd, *J =* 14.4, 10.8 Hz, 1H), 2.97-2.88 (m, 4H), 2.81-2.74 (m, 2H), 2.64 (s, 3H), 2.45-2.41 (m, 1H), 2.27 (s, 3H), 2.23 (d, *J =* 13.2 Hz, 1H) ppm; ^13^C NMR (150 MHz, CDCl_3_) δ: 152.6, 151.4, 148.6, 148.4, 147.9, 146.7, 143.5, 141.8, 137.7, 135.8, 135.5, 135.3, 134.4, 132.7, 130.5, 128.1, 127.6, 127.6, 127.5, 122.1, 122.0, 122.0, 119.6, 115.9, 112.7, 106.0, 105.8, 63.7, 61.5, 60.3, 56.0, 55.8, 55.8, 45.2, 43.5, 42.5, 42.0, 37.9, 35.0, 25.2, 21.8 ppm. HR-ESIMS: *m/z* 785.2556 [M+H]^+^, calculated for C_42_H_45_N_2_O_9_S_2_: 785.2561.

Compound **33** 14-*O*-(5-chlorothiophene-2-sulfonyl)-tetrandrine

White amorphous solid, yield 87.3%; m.p. : 118.8 ℃; ^1^H NMR (600 MHz, CDCl3) δ: 7.45 (d, *J =* 3.6 Hz, 1H), 7.37 (dd, *J =* 8.4, 2.4 Hz, 1H), 7.10 (dd, *J =* 8.4, 2.4 Hz, 1H), 6.95 (d, *J =* 4.2 Hz, 1H), 6.82 (dd, *J =* 8.4, 2.4 Hz, 1H), 6.61 (s, 1H), 6.52 (s, 2H), 6.33 (dd, *J =* 8.4, 2.4 Hz, 1H), 6.32 (s, 1H), 5.97 (s, 1H), 3.91 (dd, *J =* 10.8, 5.4 Hz, 1H), 3.81 (s, 3H), 3.76 (s, 3H), 3.68 (d, *J =* 10.2 Hz, 1H), 3.59-3.53 (m, 1H), 3.46-3.41 (m, 1H), 3.37 (s, 3H), 3.28 (dd, *J =* 12.6, 5.4 Hz, 1H), 3.21 (s, 3H), 3.01-2.88 (m, 5H), 2.82-2.74 (m, 2H), 2.64 (s, 3H), 2.45-2.40 (m, 1H), 2.26-2.24 (m, 4H) ppm; ^13^C NMR (150 MHz, CDCl3) δ: 152.6, 151.4, 148.6, 148.5, 148.0, 146.8, 143.5, 141.7, 139.9, 137.7, 135.9, 134.8, 133.3, 132.7, 130.5, 128.1, 127.7, 127.4, 126.9, 122.0, 121.9, 119.6, 116.0, 112.7, 106.0, 105.6, 63.8, 61.5, 60.3, 56.1, 55.8, 55.8, 45.3, 43.5, 42.6, 42.0, 37.9, 35.1, 25.3, 21.8 ppm. HR-ESIMS: *m/z* 819.2169 [M+H]^+^, calculated for C_42_H_44_ClN_2_O_9_S_2_: 819.2171.

Compound **34** 14-*O*-(2,4,6- trifluoromethanesulfonyl)-tetrandrine

light yellow amorphous solid, yield 86.7%; m.p. : 113.8 ℃; ^1^H NMR (600 MHz, CDCl_3_) δ: 7.37 (dd, *J =* 8.4, 2.4 Hz, 1H), 7.10 (dd, *J =* 8.4, 2.4 Hz, 1H), 6.83 (dd, *J =* 8.4, 2.4 Hz, 1H), 6.82 (s, 1H), 6.59 (s, 1H), 6.53 (s, 1H), 6.34 (dd, *J =* 8.4, 2.4 Hz, 1H), 6.32 (s, 1H), 5.99 (s, 1H), 3.95-3.93 (m, 4H), 3.76 (s, 3H), 3.72 (d, *J =* 10.8 Hz, 1H), 3.56*-*3.49 (m, 1H), 3.48-3.43 (m, 1H), 3.37 (s, 3H), 3.30 (dd, *J =* 12.6, 6.0 Hz, 1H), 3.21 (s, 3H), 3.04 (dd, *J =* 14.4, 10.8 Hz, 1H), 3.00*-*2.87 (m, 4H), 2.84*-*2.75 (m, 2H), 2.65 (s, 3H), 2.45*-*2.42 (m, 2H), 2.28 (s, 3H) ppm; ^13^C NMR (150 MHz, CDCl_3_) δ: 152.5, 151.6, 149.1, 148.7, 148.2, 147.3, 143.6, 141.8, 137.8, 135.9, 132.74, 130.6, 128.5, 127.8, 127.6, 126.9, 121.9, 121.9, 121.8, 119.8, 118.7 (q, ^1^*J*_C-F_ = 318.4 Hz), 116.1, 112.7, 106.0, 105.2, 63.7, 61.6, 60.3, 56.3, 55.8, 45.2, 43.7, 42.5, 42.1, 38.0, 35.7, 25.2, 21.7 ppm. HR-ESIMS: *m/z* 771.2558 [M+H]^+^, calculated for C_39_H_42_F_3_N_2_O_9_S: 771.2557.

Compound **35** 14-*O*-(dimethylsulfamoyl)-tetrandrine

White amorphous solid, yield 28.5%; m.p. : 200.0 ℃; ^1^H NMR (600 MHz, CDCl_3_) δ: 7.36 (dd, *J =* 8.4, 2.4 Hz, 1H), 7.08 (dd, *J =* 8.4, 2.4 Hz, 1H), 6.93 (s, 1H), 6.87 (dd, *J =* 8.4, 2.4 Hz, 1H), 6.57 (s, 1H), 6.53 (s, 1H), 6.35 (dd, *J =* 8.4, 2.4 Hz, 1H), 6.31 (s, 1H), 5.98 (s, 1H), 3.97-3.94 (m, 4H), 3.82 (d, *J =* 7.2 Hz, 1H), 3.76 (s, 3H), 3.60-3.54 (m, 1H), 3.50-3.45 (m, 1H), 3.39 (s, 3H), 3.33 (dd, *J =* 12.6, 5.4 Hz, 1H), 3.24-3.20 (m, 4H), 3.03 (s, 6H), 3.00-2.91 (m, 4H), 2.83-2.77 (m, 2H), 2.65 (s, 3H), 2.49-2.45 (m, 1H), 2.40 (d, *J =* 14.4 Hz, 1H), 2.30 (s, 3H) ppm; ^13^C NMR (150 MHz, CDCl_3_) δ: 152.9, 151.5, 148.7, 148.2, 147.9, 147.0, 143.6, 142.0, 137.9, 135.5, 132.5, 130.6, 127.9, 127.5, 127.3, 126.7, 122.2, 122.0, 119.7, 115.8, 112.7, 106.4, 105.9, 63.7, 61.6, 60.4, 56.3, 55.9, 55.9, 45.1, 44.2, 42.4, 42.2, 38.8, 38.0, 34.6, 29.7, 25.0, 22.6 ppm. HR-ESIMS: *m/z* 746.3102 [M+H]^+^, calculated for C_40_H_48_N_3_O_9_S: 746.3106.

Compound **36** 14-*O*-(ethanesulfonyl)-tetrandrine

light yellow amorphous solid, yield 88.1%; m.p. : 131.6 ℃; ^1^H NMR (600 MHz, CDCl_3_) δ: 7.36 (dd, *J =* 8.4, 2.4 Hz, 1H), 7.08 (dd, *J =* 8.4, 2.4 Hz, 1H), 6.99 (s, 1H), 6.85 (dd, *J =* 8.4, 2.4 Hz, 1H), 6.58 (s, 1H), 6.53 (s, 1H), 6.35 (dd, *J =* 8.4, 2.4 Hz, 1H), 6.31 (s, 1H), 5.98 (s, 1H), 3.94-3.92 (m, 4H), 3.76 (d, *J =* 10.8 Hz,, 1H), 3.75 (s, 3H), 3.60-3.55 (m, 1H), 3.48-3.42 (m, 2H), 3.38 (s, 3H), 3.35-3.28 (m, 2H), 3.20 (s, 3H), 3.15 (dd, *J =* 14.4, 10.2 Hz,, 1H), 2.98-2.88 (m, 4H), 2.83-2.74 (m, 2H), 2.64 (s, 3H), 2.47-2.44 (m, 1H), 2.37 (d, *J =* 13.8 Hz, 1H), 2.29 (s, 3H), 1.54 (t, *J =* 7.8 Hz, 3H) ppm; ^13^C NMR (150 MHz, CDCl_3_) δ: 152.8, 151.5, 148.6, 148.3, 148.1, 147.1, 143.5, 141.3, 137.8, 135.8, 132.6, 130.5, 128.0, 127.8, 127.7, 126.7, 122.1, 122.1, 121.9, 119.8, 116.1, 112.7, 105.9, 105.8, 63.7, 61.5, 60.3, 56.3, 55.9, 55.8, 45.2, 43.9, 42.5, 42.0, 37.9, 35.2, 29.7, 25.2, 22.0, 8.1 ppm. HR-ESIMS: *m/z* 731.2995 [M+H]^+^, calculated for C_40_H_47_N_2_O_9_S: 731.2997.

Compound **37** 14-*O*-(1-propanesulfonyl)-tetrandrine

light yellow amorphous solid, yield 91.4%; m.p. : 129.2 ℃; ^1^H NMR (600 MHz, CDCl_3_) δ: 7.37 (dd, *J =* 8.4, 2.4 Hz, 1H), 7.09 (dd, *J =* 8.4, 2.4 Hz, 1H), 7.01 (s, 1H), 6.85 (dd, *J =* 8.4, 2.4 Hz, 1H), 6.58 (s, 1H), 6.53 (s, 1H), 6.35 (dd, *J =* 8.4, 2.4 Hz, 1H), 6.32 (s, 1H), 5.98 (s, 1H), 3.95-3.92 (m, 4H), 3.79 (d, *J =* 10.2 Hz, 1H), 3.76 (s, 3H), 3.62-3.56 (m, 1H), 3.48-3.40 (m, 2H), 3.38 (s, 3H), 3.31 (dd, *J =* 12.6, 6.0 Hz, 1H), 3.28-3.23 (m, 1H), 3.20 (s, 3H), 3.15 (dd, *J =* 14.4, 10.8 Hz, 1H), 3.00-2.88 (m, 4H), 2.83-2.75 (m, 2H), 2.65 (s, 3H), 2.46 (dd, *J =* 16.8, 6.0 Hz, 1H), 2.36 (d, *J =* 14.4 Hz, 1H), 2.29 (s, 3H), 2.06-1.99 (m, 2H), 1.12 (t, *J =* 7.8 Hz, 3H) ppm; ^13^C NMR (150 MHz, CDCl_3_) δ: 152.8, 151.5, 148.6, 148.2, 148.1, 147.1, 143.6, 141.4, 137.8, 135.7, 132.6, 130.6, 128.0, 127.7, 127.5, 126.5, 122.1, 122.0, 119.8, 116.1, 112.7, 106.0, 105.6, 63.7, 61.5, 60.3, 56.3, 55.9, 55.8, 52.1, 45.2, 43.8, 42.5, 42.0, 38.0, 35.2, 25.2, 22.0, 17.2, 13.1 ppm. HR-ESIMS: *m/z* 745.3154 [M+H]^+^, calculated for C_41_H_49_N_2_O_9_S: 745.3153.

Compound **38** 14-*O*-(1-butanesulfonyl)-tetrandrine

light yellow amorphous solid, yield 85.6%; m.p. : 123.4 ℃; ^1^H NMR (600 MHz, CDCl_3_) δ: 7.38 (dd, *J =* 8.4, 2.4 Hz, 1H), 7.09 (dd, *J =* 8.4, 2.4 Hz, 1H), 6.99 (s, 1H), 6.87 (dd, *J =* 8.4, 2.4 Hz, 1H), 6.57 (s, 1H), 6.54 (s, 1H), 6.35 (dd, *J =* 8.4, 2.4 Hz, 1H), 6.32 (s, 1H), 6.00 (s, 1H), 4.01 (dd, *J =* 10.8, 5.4 Hz, 1H), 3.94 (s, 3H), 3.76 (s, 3H), 3.63*-*3.53 (m, 2H), 3.46*-*3.41 (m, 2H), 3.40 (s, 3H), 3.32*-*3.26 (m, 1H), 3.20 (s, 3H), 3.19*-*3.14 (m, 1H), 3.03*-*2.89 (m, 3H), 2.89*-*2.80 (m, 2H), 2.70 (s, 3H), 2.51-2.47 (m, 1H), 2.38 (d, *J =* 14.4 Hz, 1H), 2.31 (s, 3H), 2.00-1.95 (m, 2H), 1.52 (sext, *J =* 7.8 Hz, 2H), 0.98 (t, *J =* 7.8 Hz, 3H) ppm; ^13^C NMR (150 MHz, CDCl_3_) δ: 152.9, 151.6, 149.0, 148.2, 147.9, 147.3, 143.8, 141.3, 137.8, 135.0, 132.6, 130.6, 127.9, 127.1, 126.4, 122.2, 122.0, 119.8, 116.1, 112.6, 106.0, 105.8, 63.9, 61.5, 60.3, 56.4, 55.9, 55.8, 50.4, 45.1, 43.9, 42.2, 41.9, 38.3, 35.0, 25.4, 24.6, 22.1, 21.6, 13.6 ppm. HR-ESIMS: *m/z* 759.3312 [M+H]^+^, calculated for C_42_H_51_N_2_O_9_S: 759.3310.

Compound **39** 14-*O*-(cyclopropanesulfonyl)-tetrandrine

light yellow amorphous solid, yield 80.4%; m.p. : 132.6 ℃; ^1^H NMR (600 MHz, CDCl_3_) δ: 7.37 (dd, *J =* 8.4, 2.4 Hz, 1H), 7.09 (dd, *J =* 8.4, 2.4 Hz, 1H), 6.99 (s, 1H), 6.86 (dd, *J =* 8.4, 2.4 Hz, 1H), 6.56 (s, 1H), 6.53 (s, 1H), 6.35 (dd, *J =* 8.4, 2.4 Hz, 1H), 6.32 (s, 1H), 5.99 (s, 1H), 3.94-3.91 (m, 4H), 3.78 (d, *J =* 10.2 Hz, 1H), 3.76 (s, 3H), 3.66-3.60 (m, 1H), 3.47-3.42 (m, 1H), 3.38 (s, 3H), 3.29 (dd, *J =* 12.6, 5.4 Hz, 1H), 3.22-3.18 (s, 4H), 3.00-2.89 (m, 4H), 2.83-2.74 (m, 3H), 2.65 (s, 3H), 2.50-2.42 (m, 1H), 2.37 (d, *J =* 13.2 Hz, 1H), 2.29 (s, 3H), 1.33-1.23 (m, 2H), 1.12-1.05 (m, 2H) ppm; ^13^C NMR (150 MHz, CDCl_3_) δ: 152.7, 151.5, 148.6, 148.3, 148.0, 146.9, 143.5, 141.7, 137.8, 135.8, 132.6, 130.5, 128.0, 127.7, 127.7, 126.8, 122.1, 122.0, 119.7, 115.8, 112.7, 106.3, 106.0, 63.7, 61.5, 60.3, 56.3, 55.9, 55.8, 45.2, 43.6, 42.5, 42.0, 37.9, 35.1, 29.7, 27.9, 25.2, 22.0, 6.6, 5.9 ppm. HR-ESIMS: *m/z* 743.2997 [M+H]^+^, calculated for C_41_H_47_N_2_O_9_S: 731.2997.

Compound **40** 14-*O*-(2-phthalimidoethanesulfonyl)-tetrandrine

Beige amorphous solid, yield 87.3%; m.p. : 116.3 ℃; ^1^H NMR (600 MHz, CDCl_3_) δ: 7.87 (dd, *J =* 5.4, 3.0 Hz, 2H), 7.74 (dd, *J =* 5.4, 3.0 Hz, 2H), 7.36 (dd, *J =* 8.4, 2.4 Hz, 1H), 7.10 (s, 1H), 7.08 (dd, *J =* 8.4, 2.4 Hz, 1H), 6.84 (dd, *J =* 8.4, 2.4 Hz, 1H), 6.57 (s, 1H), 6.52 (s, 1H), 6.34 (dd, *J =* 8.4, 2.4 Hz, 1H), 6.31 (s, 1H), 5.97 (s, 1H), 4.39-4.29 (m, 2H), 3.99 (s, 3H), 3.94-3.87 (m, 2H), 3.76-3.74 (m, 4H), 3.73-3.70 (m, 1H), 3.61-3.54 (m, 1H), 3.47-3.42 (m, 1H), 3.37 (s, 3H), 3.29 (dd, *J =* 12.6, 6.0 Hz, 1H), 3.19 (s, 3H), 3.14 (dd, *J =* 14.4, 10.8 Hz, 1H), 3.99-2.88 (m, 4H), 2.82-2.73 (m, 2H), 2.64 (s, 3H), 2.48-2.44 (m, 1H), 2.35 (d, *J =* 13.8 Hz, 1H), 2.24 (s, 3H) ppm; ^13^C NMR (150 MHz, CDCl_3_) δ: 167.5, 152.7, 151.5, 148.6, 148.3, 148.1, 147.3, 143.6, 141.3, 137.8, 135.7, 134.3, 132.6, 131.8, 130.5, 128.1, 127.7, 127.6, 126.7, 123.6, 122.0, 122.0, 119.8, 116.2, 112.7, 106.0, 105.4, 77.3, 77.1, 76.9, 63.7, 61.5, 60.3, 56.5, 55.8, 55.8, 47.6, 45.2, 43.7, 42.5, 41.8, 38.0, 35.3, 32.4, 29.7, 25.2, 21.9 ppm. HR-ESIMS: *m/z* 876.3165 [M+H]^+^, calculated for C_48_H_50_N_3_O_11_S: 876.3161.

# NMR and MS Spectra

**Compound M1. C14-nitro-tetrandrine: ^1^H-NMR spectrum**


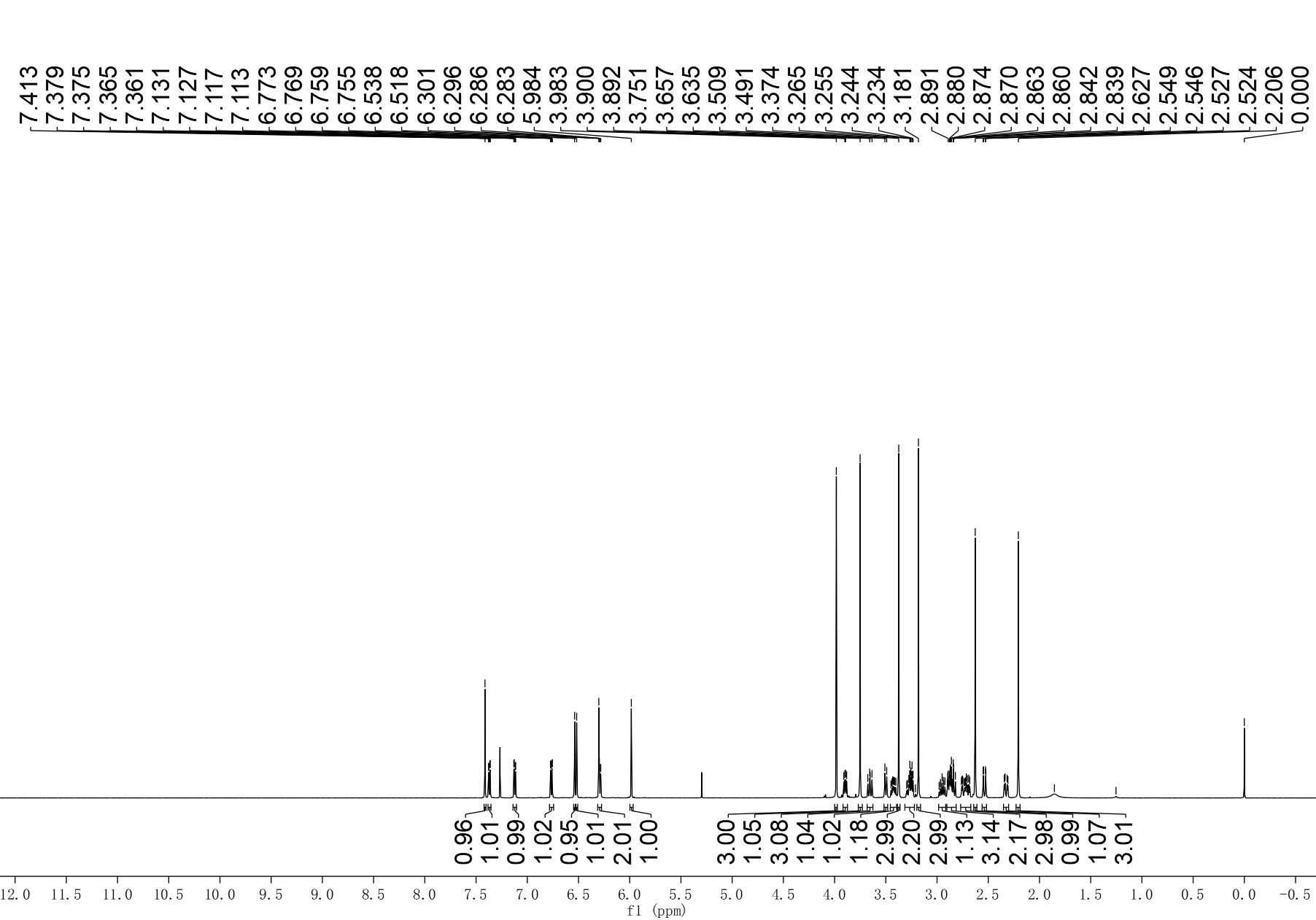


**Compound M1. C14-nitro-tetrandrine: ^13^C-NMR spectrum**

**
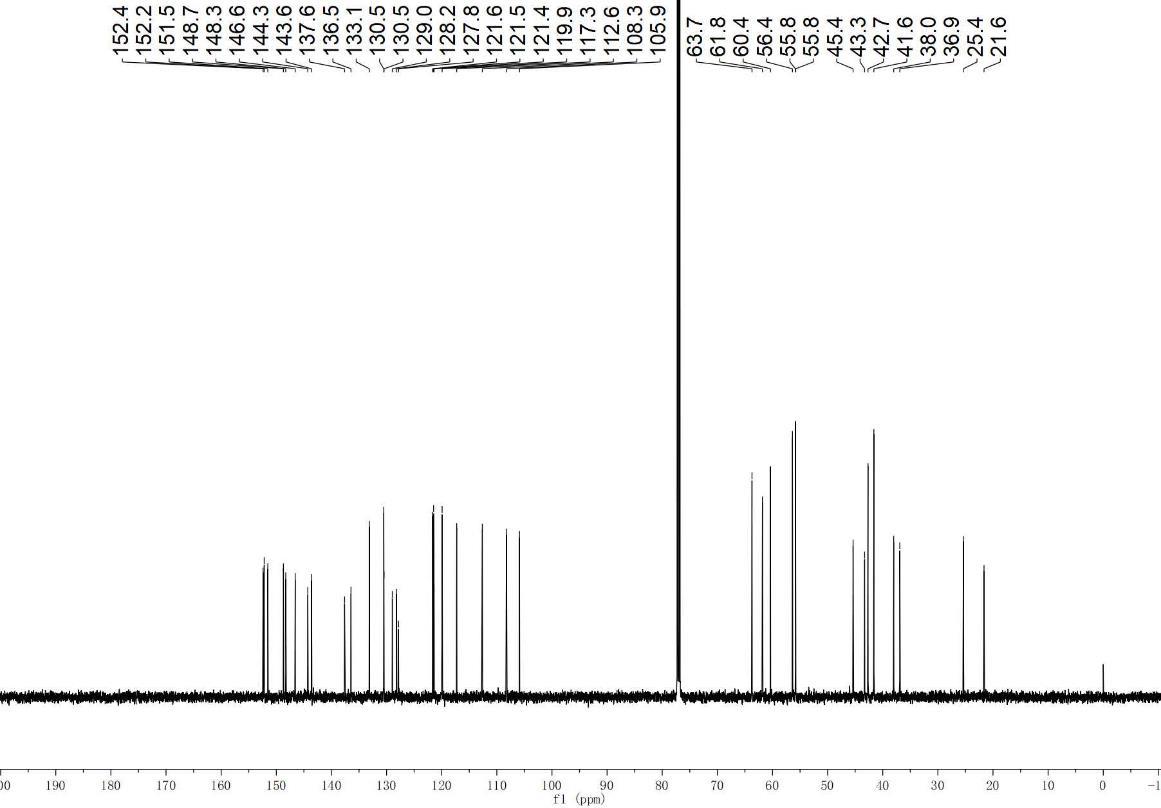
**

**Compound M1. C14-nitro-tetrandrine: HR-ESIMS spectrum**

**Compound M2. C14-amino-tetrandrine: ^1^H-NMR spectrum**

**
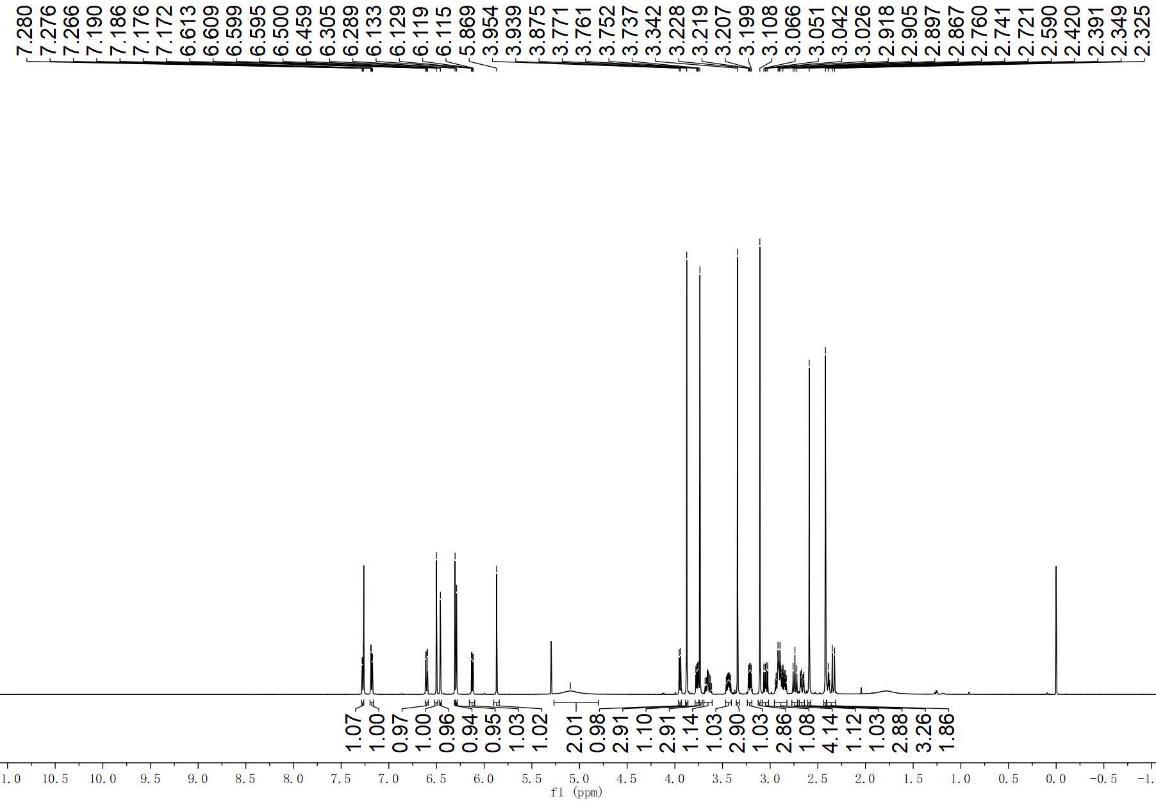
**

**Compound M2. C14-amino-tetrandrine: ^13^C-NMR spectrum**

**
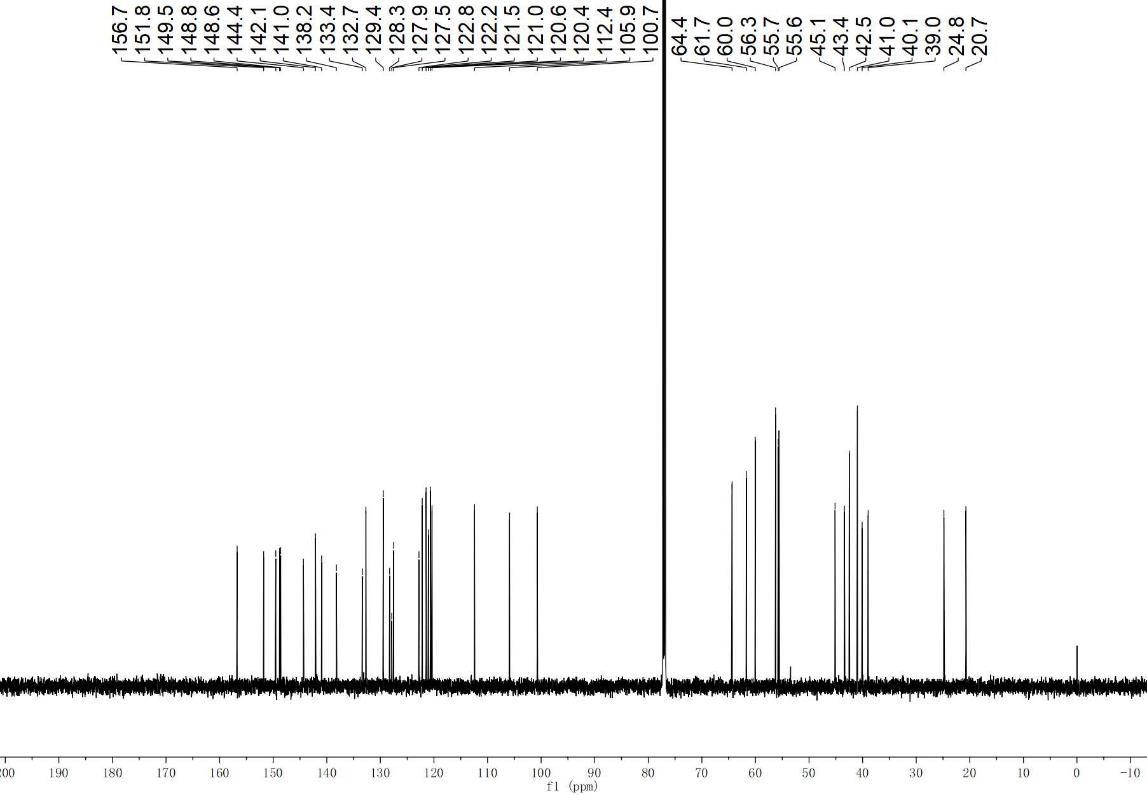
**

**Compound M2. C14-amino-tetrandrine: HR-ESIMS spectrum**

**Compound M3. C14-hydroxyl-tetrandrine: ^1^H-NMR spectrum**

**
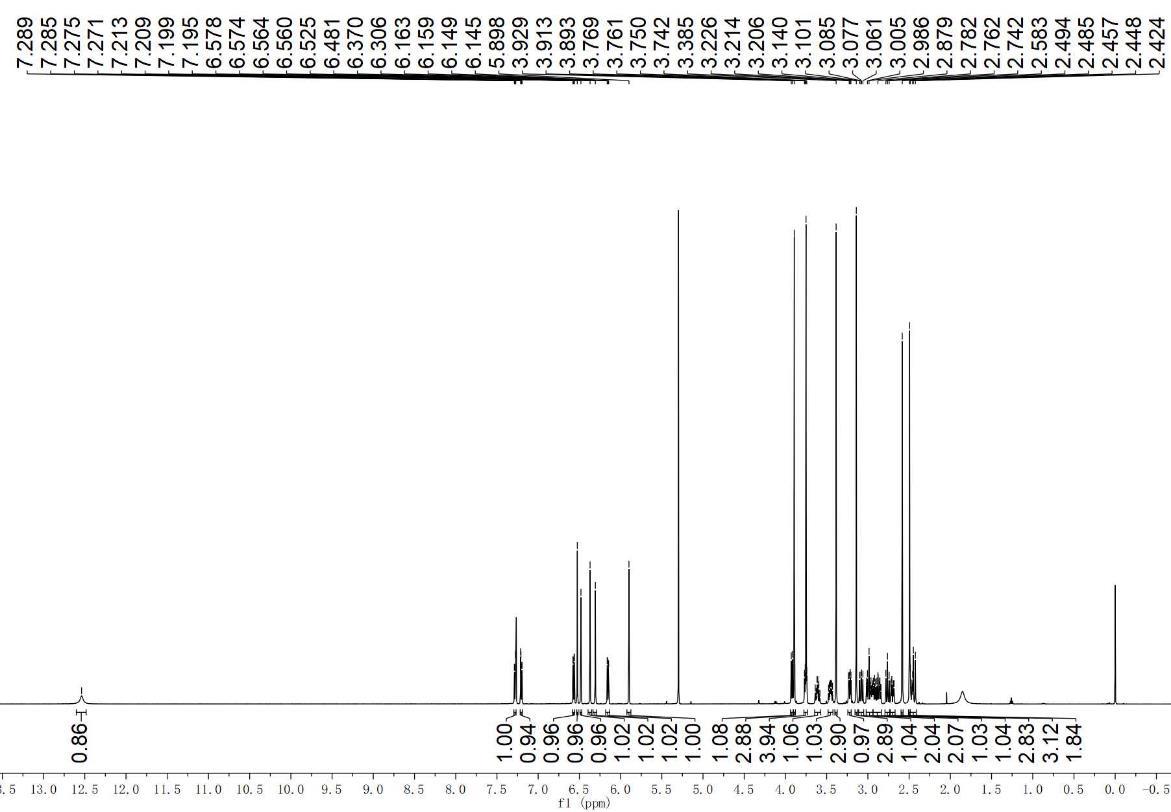
**

**Compound M3. C14-hydroxyl-tetrandrine: ^13^C-NMR spectrum**

**
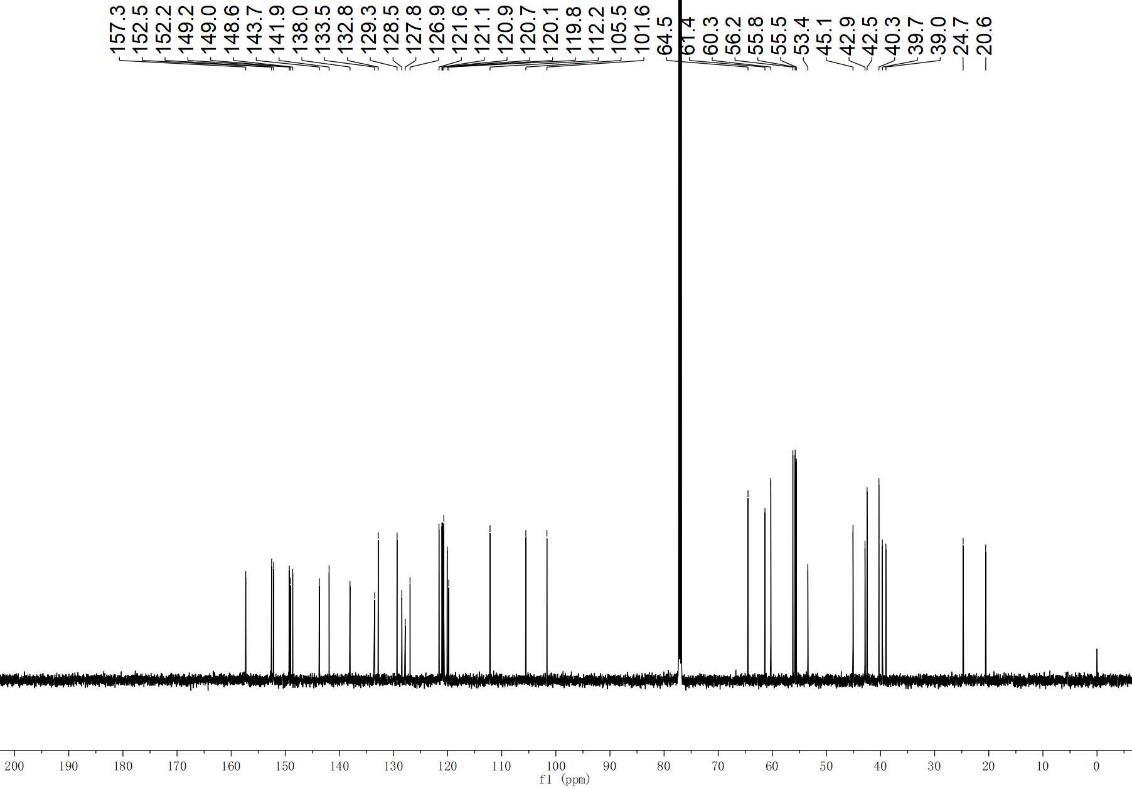
**

**Compound M3. C14-hydroxyl-tetrandrine: HR-ESIMS spectrum**

**Compound 1. 14-*O*-(benzenesulfonyl)-tetrandrine: ^1^H-NMR spectrum**


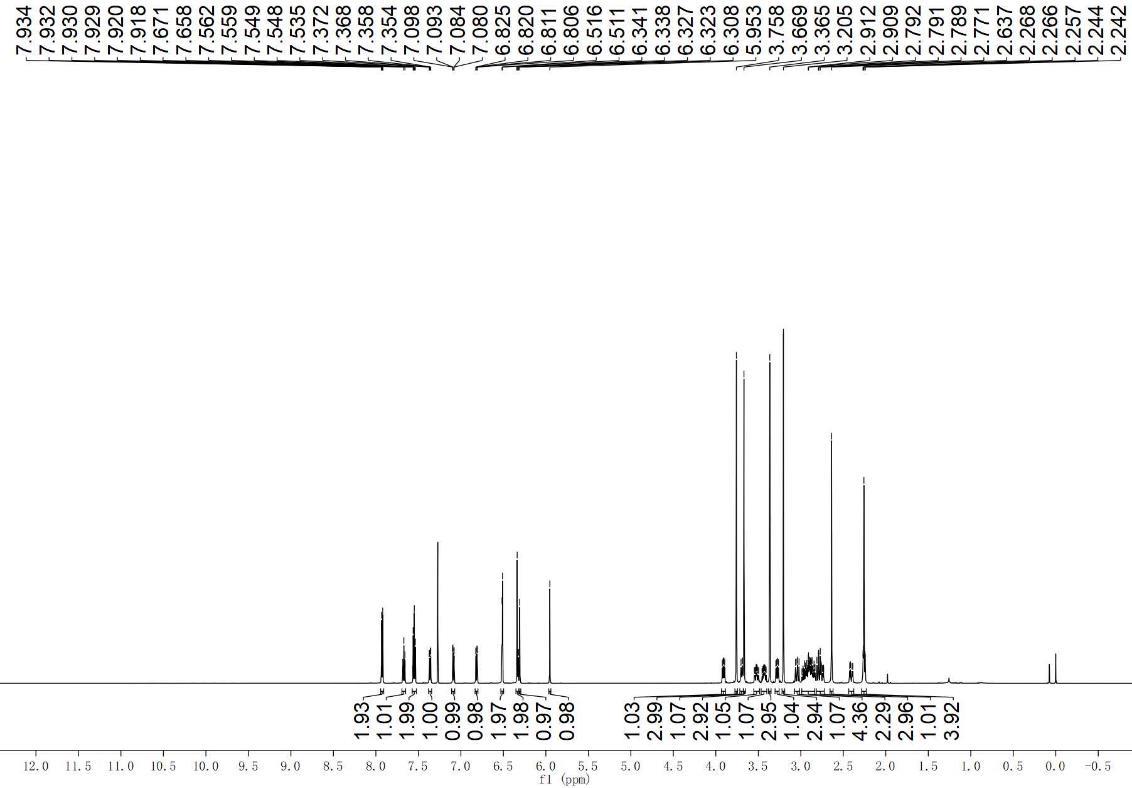


**Compound 1. 14-*O*-(benzenesulfonyl)-tetrandrine: ^13^C-NMR spectrum**


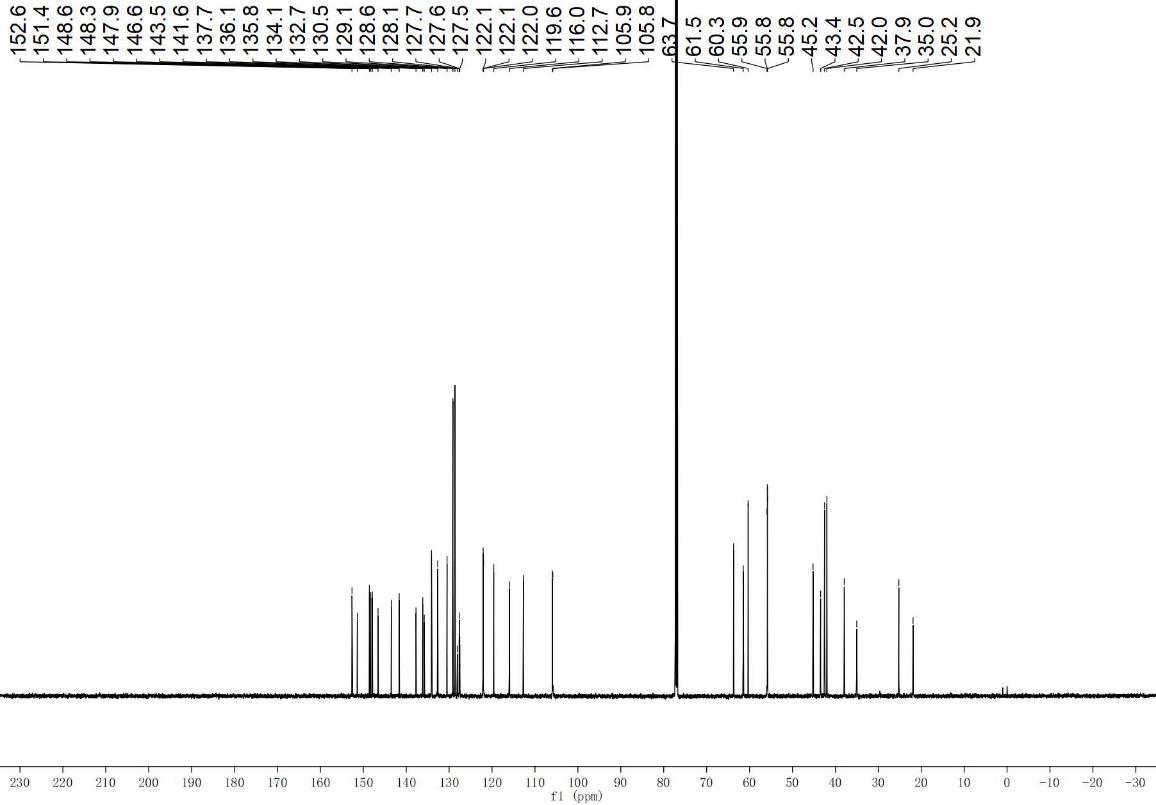


**Compound 1. 14-*O*-(benzenesulfonyl)-tetrandrine: HR-ESIMS spectrum**

**Compound 2.** **14-*O*-(alpha-toluenesulfonyl)-tetrandrine: ^1^H-NMR spectrum**


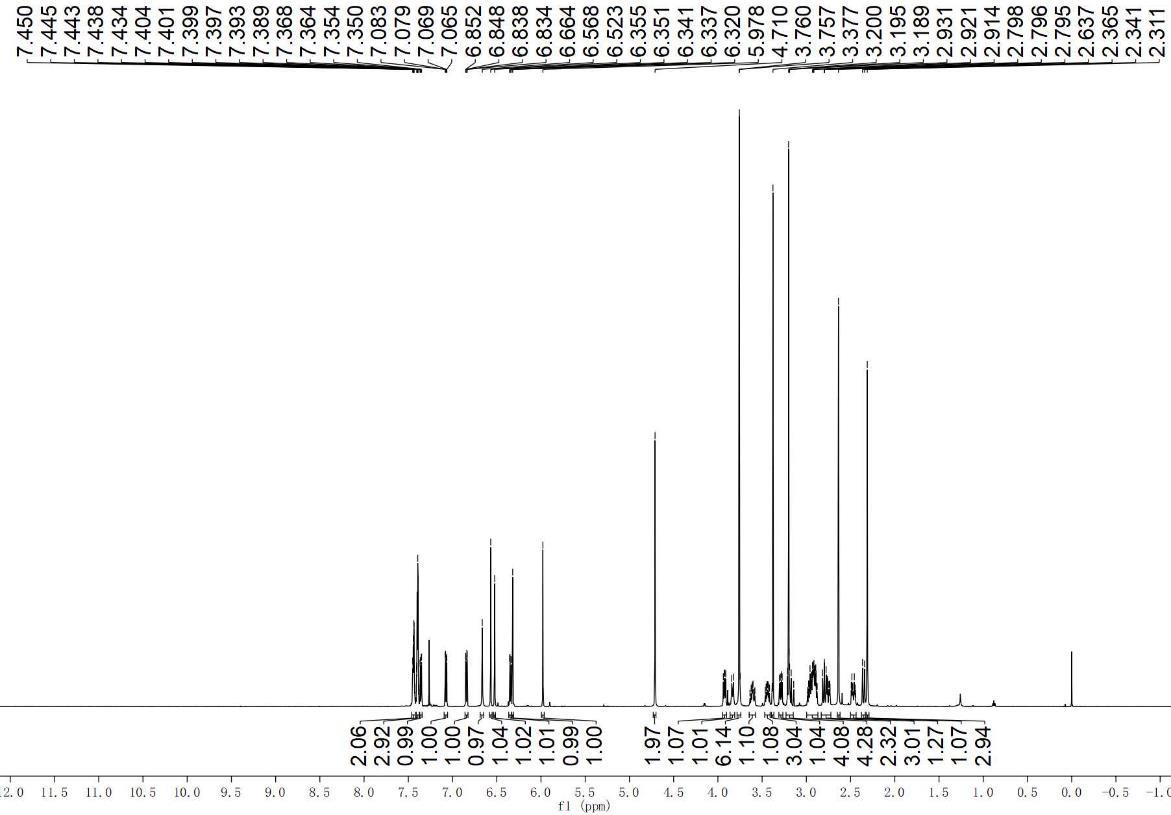


**Compound 2. 14-*O*-(alpha-toluenesulfonyl)-tetrandrine: ^13^C-NMR spectrum**


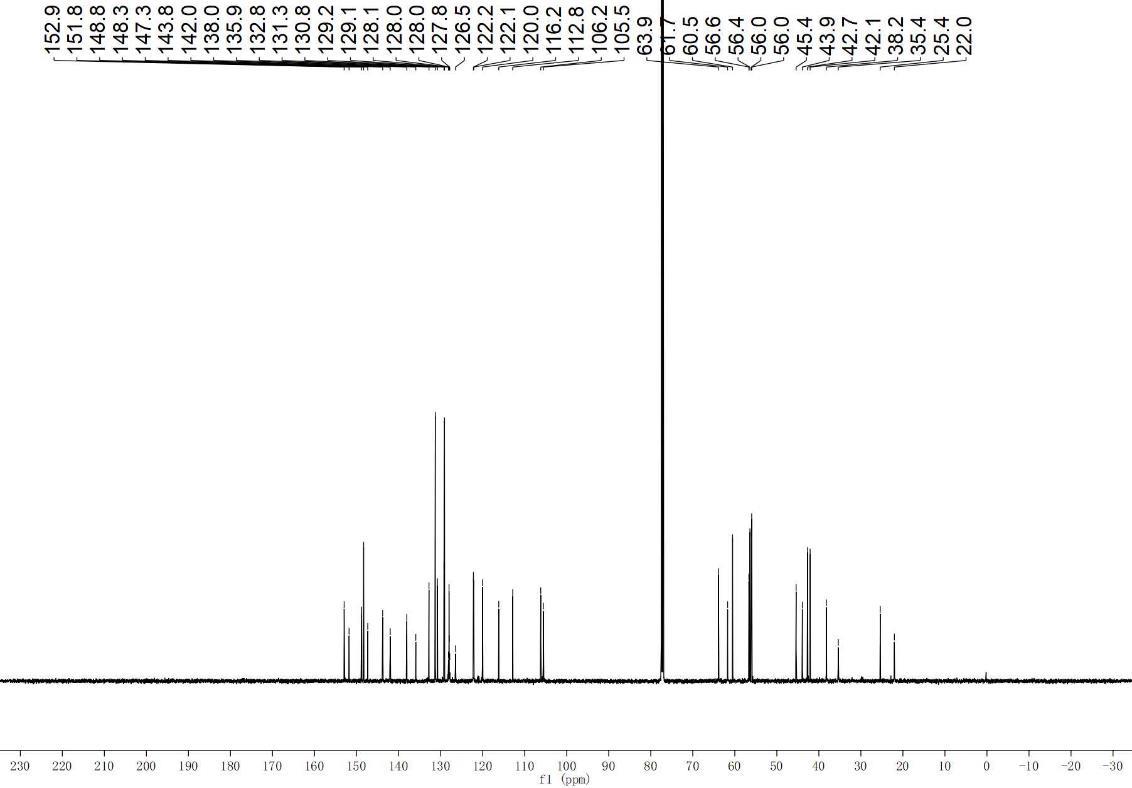


**Compound 2. 14-*O*-(alpha-toluenesulfonyl)-tetrandrine: HR-ESIMS spectrum**

**Compound 3.** **14-*O*-(2-fluorobenzenesulfonyl)-tetrandrine: ^1^H-NMR spectrum**


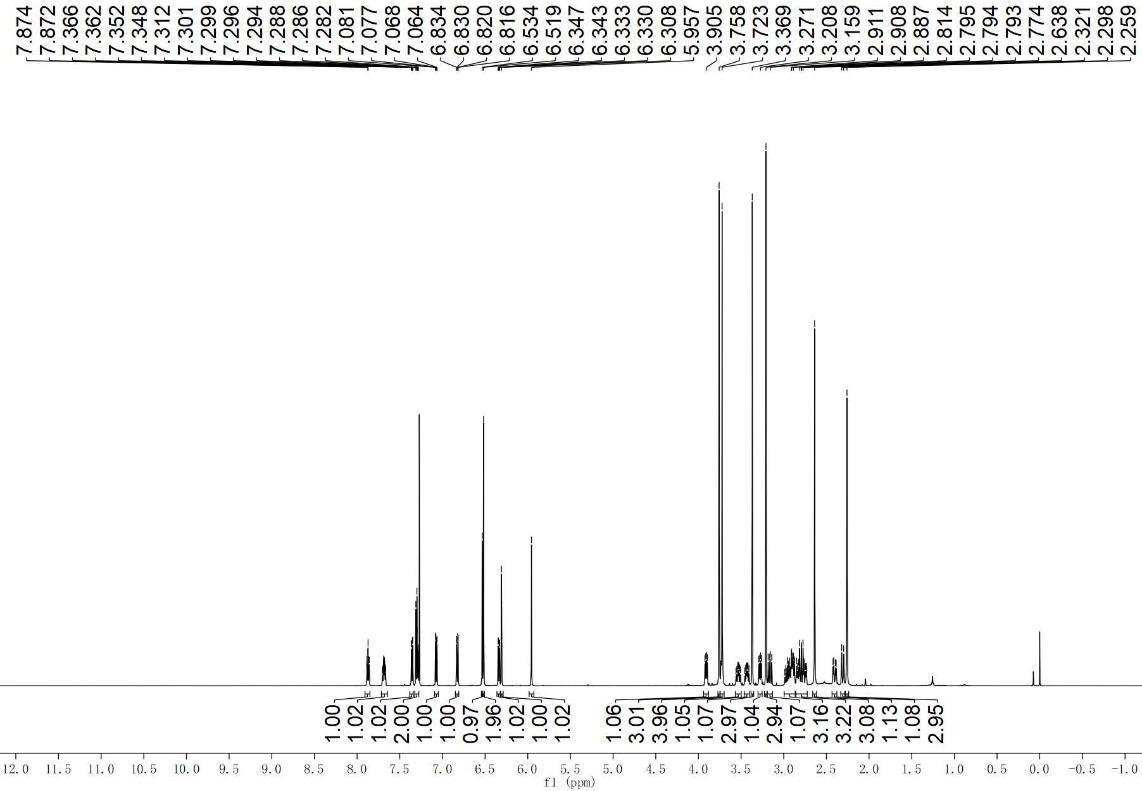


**Compound 3. 14-*O*-(2-fluorobenzenesulfonyl)-tetrandrine: ^13^C-NMR spectrum**


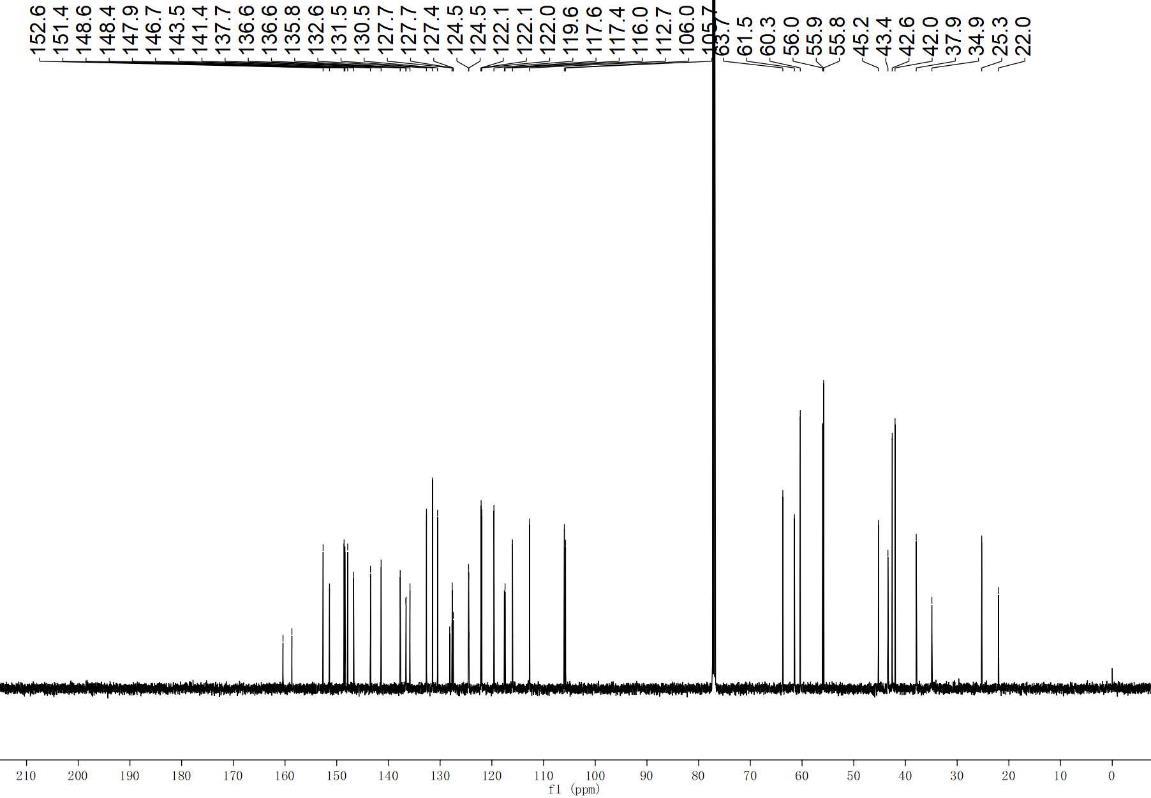


**Compound 3. 14-*O*-(2-fluorobenzenesulfonyl)-tetrandrine: ^19^F-NMR spectrum**

**Compound 3. 14-*O*-(2-fluorobenzenesulfonyl)-tetrandrine: HR-ESIMS spectrum**

**Compound 4** **14-*O*-(3-fluorobenzenesulfonyl)-tetrandrine: ^1^H-NMR spectrum**


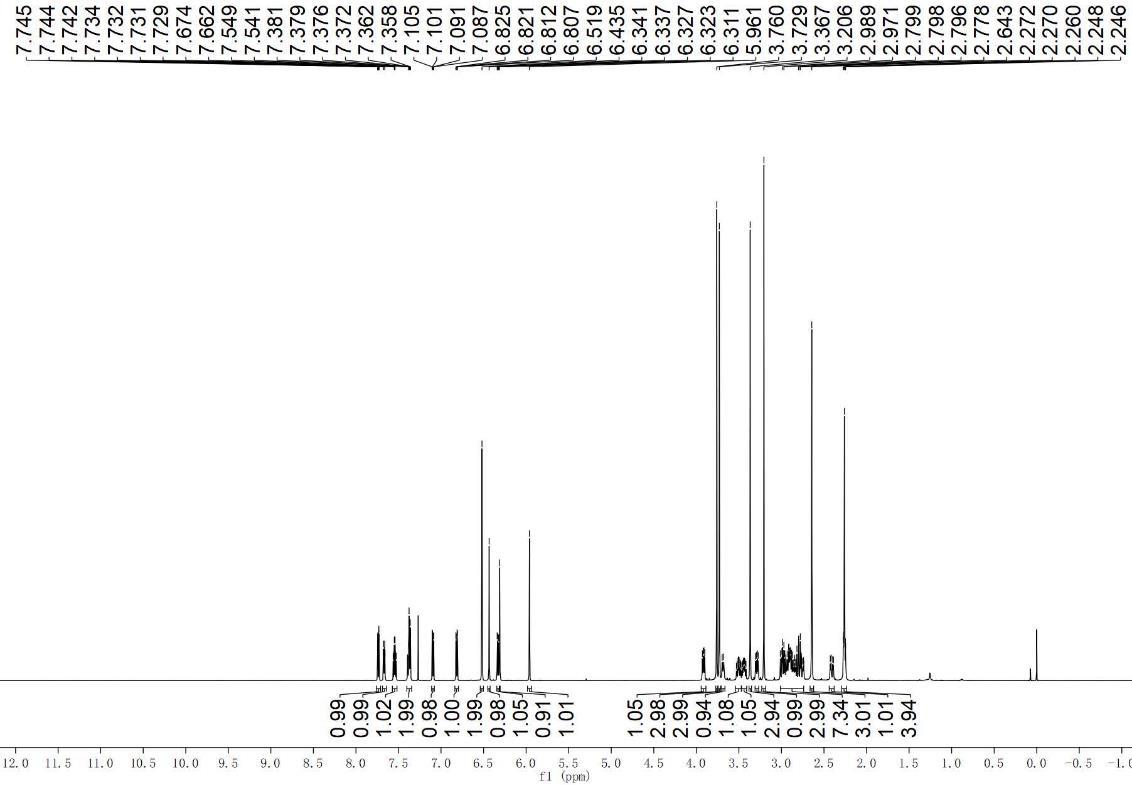


**Compound 4 14-*O*-(3-fluorobenzenesulfonyl)-tetrandrine: ^13^C-NMR spectrum**


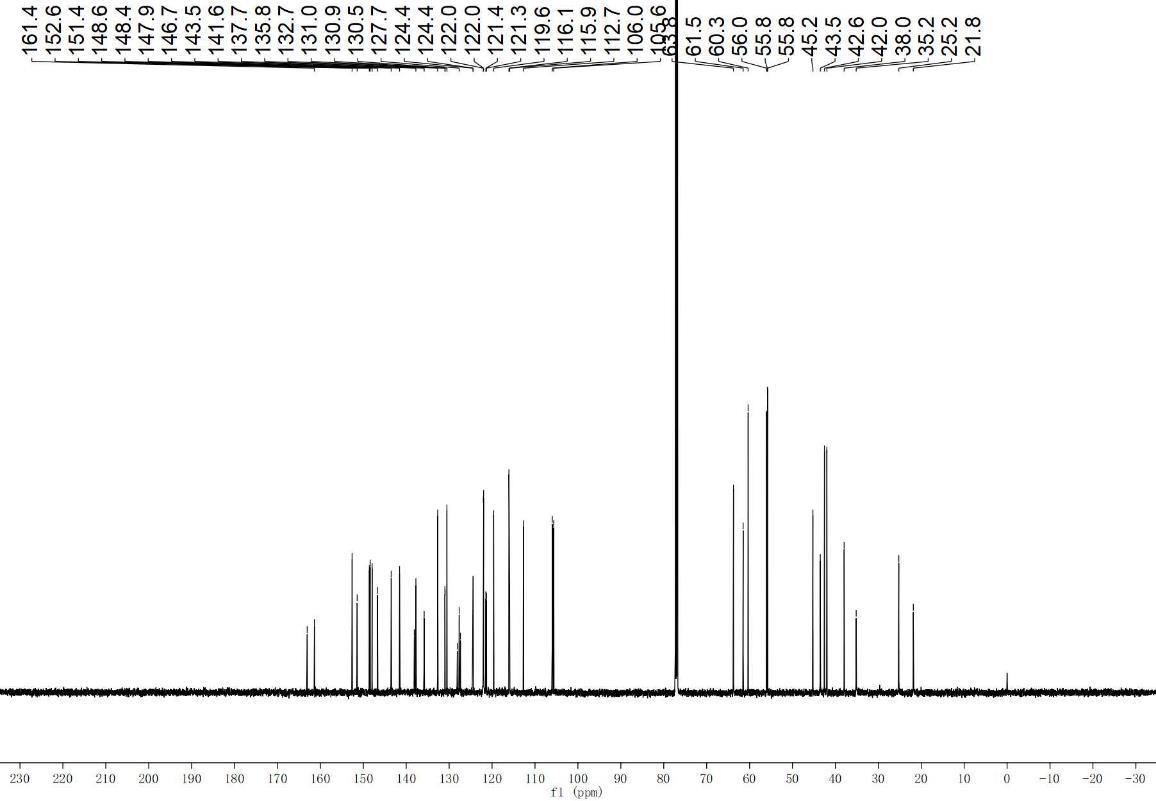


**Compound 4 14-*O*-(3-fluorobenzenesulfonyl)-tetrandrine: ^19^F-NMR spectrum**

**Compound 4 14-*O*-(3-fluorobenzenesulfonyl)-tetrandrine: HR-ESIMS spectrum**

**Compound 5.** **14-*O*-(4-fluorobenzenesulfonyl)-tetrandrine: ^1^H-NMR spectrum**


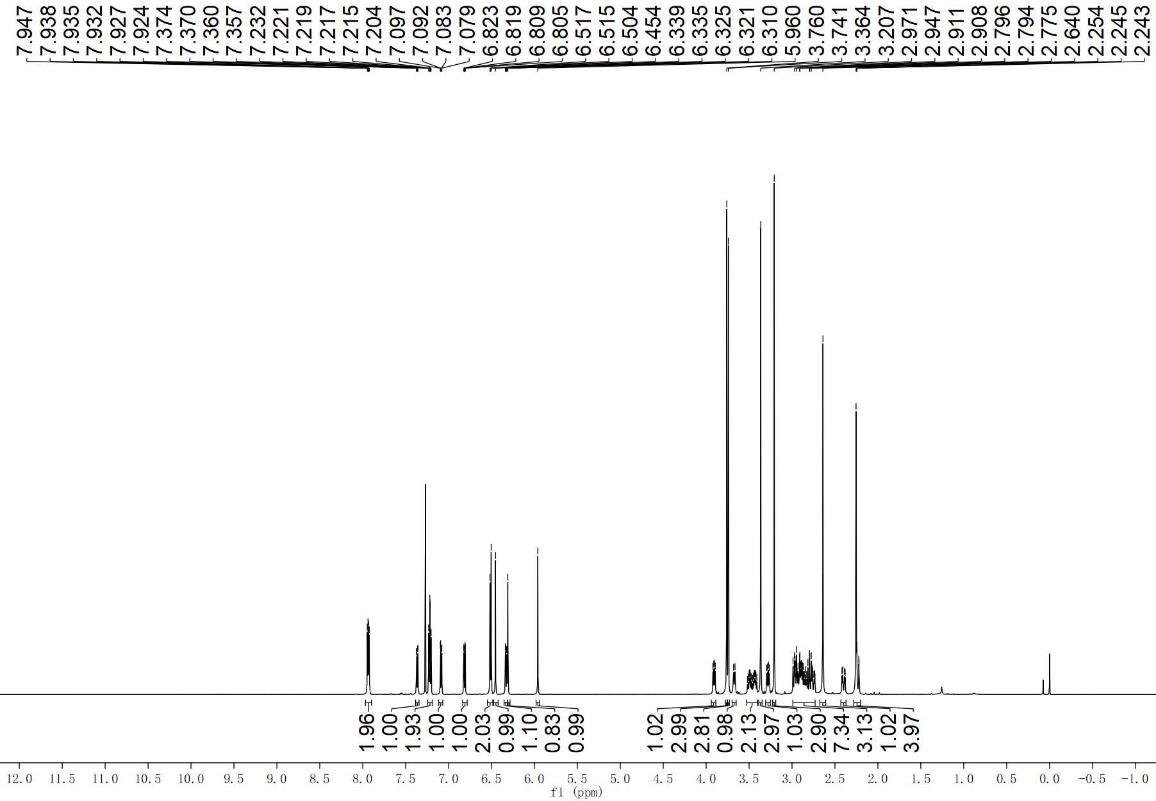


**Compound 5. 14-*O*-(4-fluorobenzenesulfonyl)-tetrandrine: ^13^C-NMR spectrum**


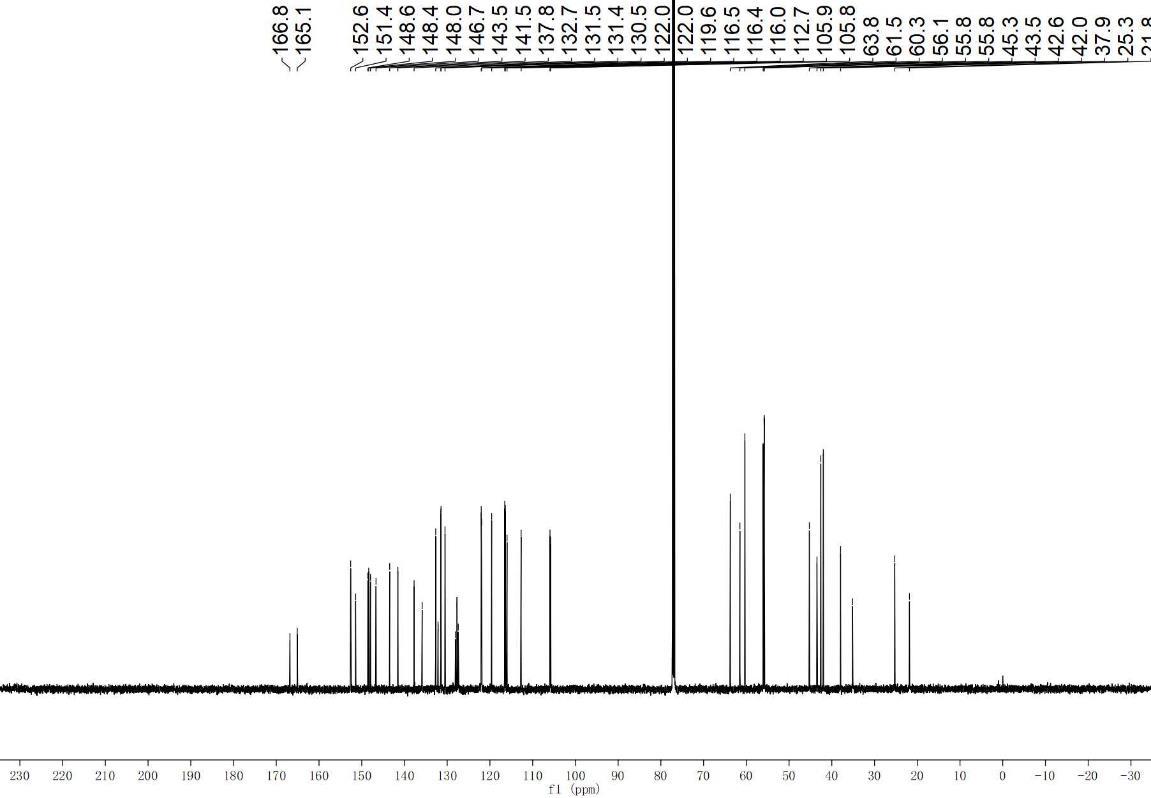


**Compound 5. 14-*O*-(4-fluorobenzenesulfonyl)-tetrandrine: ^19^F-NMR spectrum**

**Compound 5. 14-*O*-(4-fluorobenzenesulfonyl)-tetrandrine: HR-ESIMS spectrum**

**Compound 6.** **14-*O*-(2-chlorobenzenesulfonyl)-tetrandrine: ^1^H-NMR spectrum**


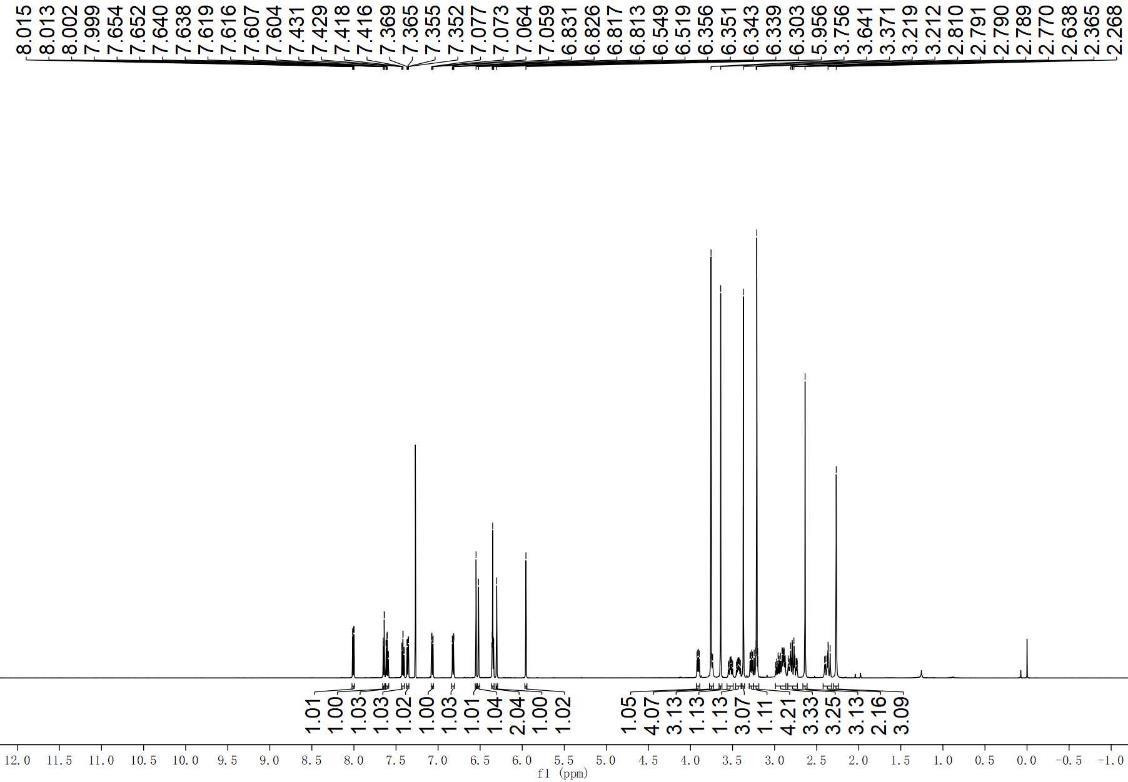


**Compound 6. 14-*O*-(2-chlorobenzenesulfonyl)-tetrandrine: ^13^C-NMR spectrum**


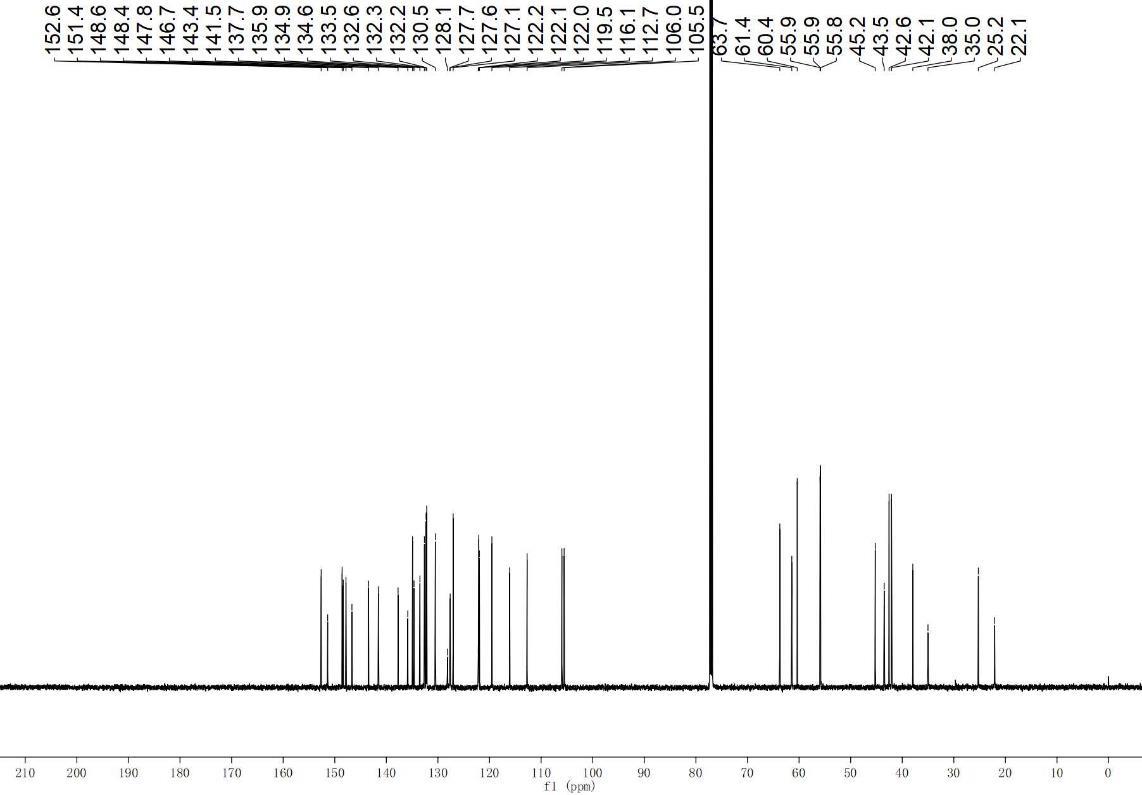


**Compound 6. 14-*O*-(2-chlorobenzenesulfonyl)-tetrandrine: ^1^H-NMR spectrum**

**Compound 7.** **14-*O*-(2-chlorobenzensulfonly)-tetrandrine: ^1^H-NMR spectrum**


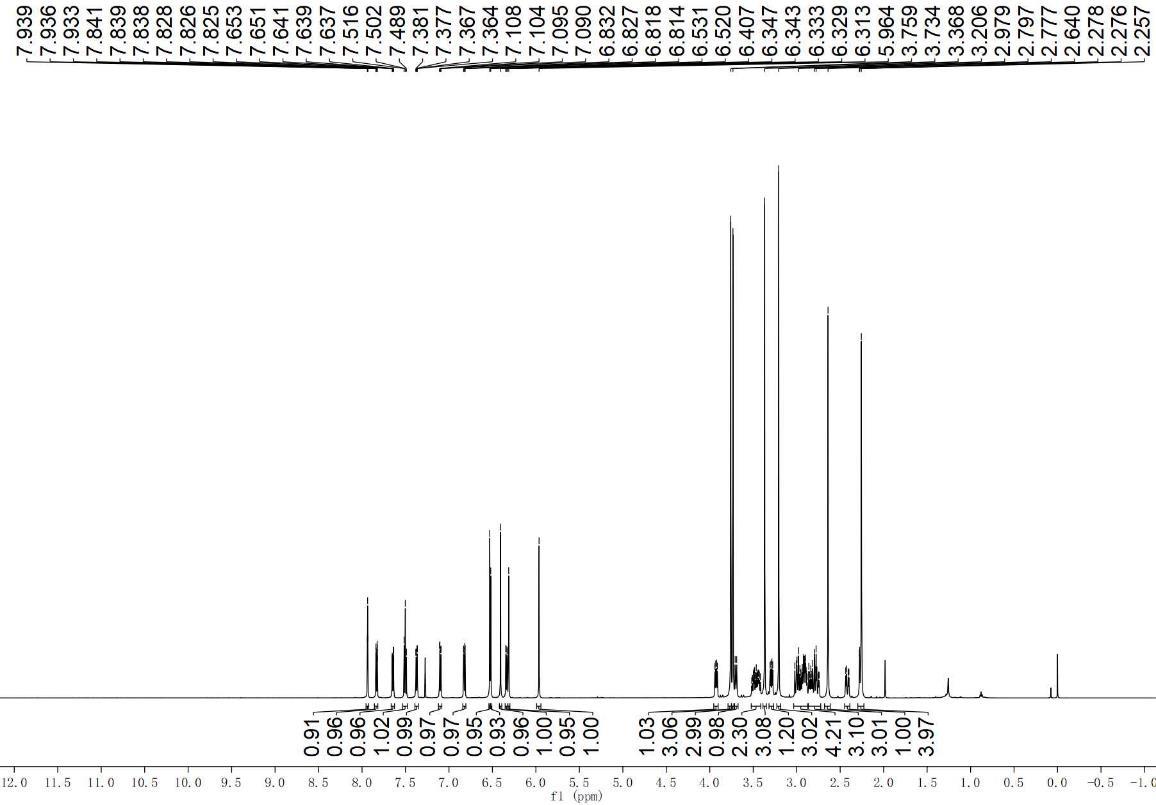


**Compound 7. 14-*O*-(2-chlorobenzensulfonly)-tetrandrine: ^13^C-NMR spectrum**


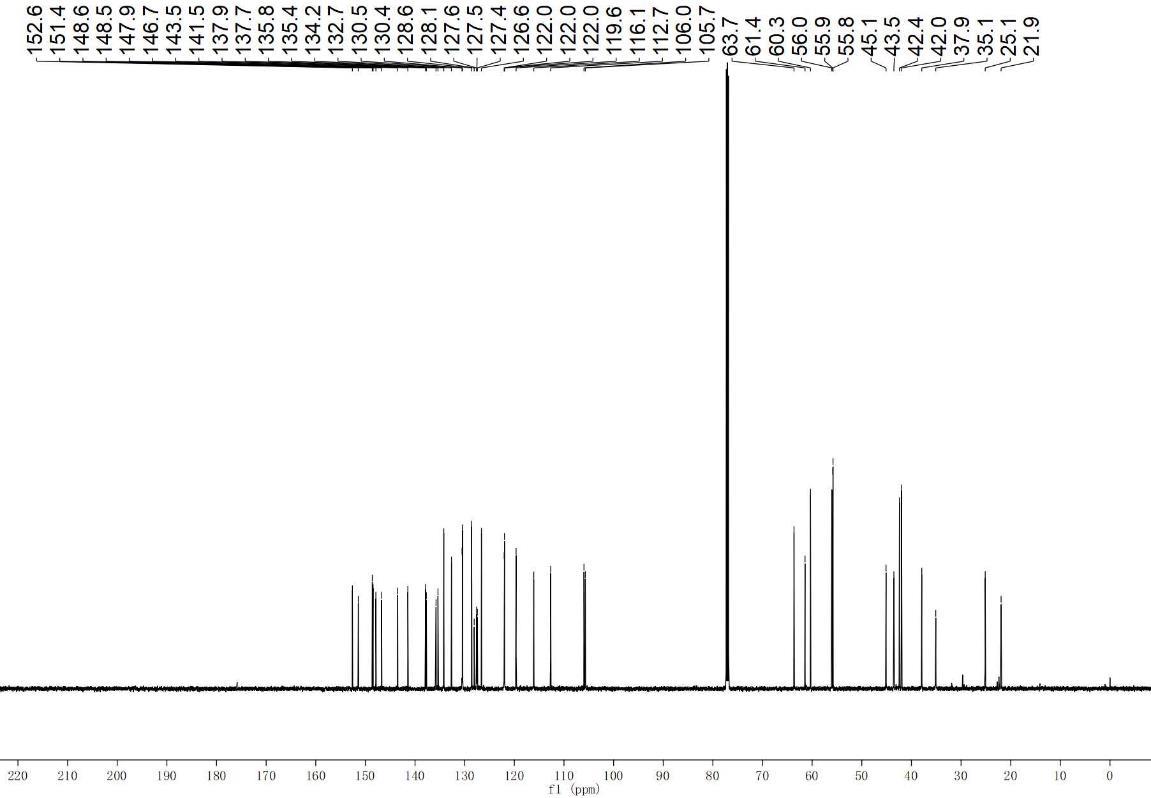


**Compound 7. 14-*O*-(2-chlorobenzensulfonly)-tetrandrine: HR-ESIMS spectrum**

**Compound 8.** **14-*O*-(4-chlorobenzenesulfonyl)-tetrandrine: ^1^H-NMR spectrum**


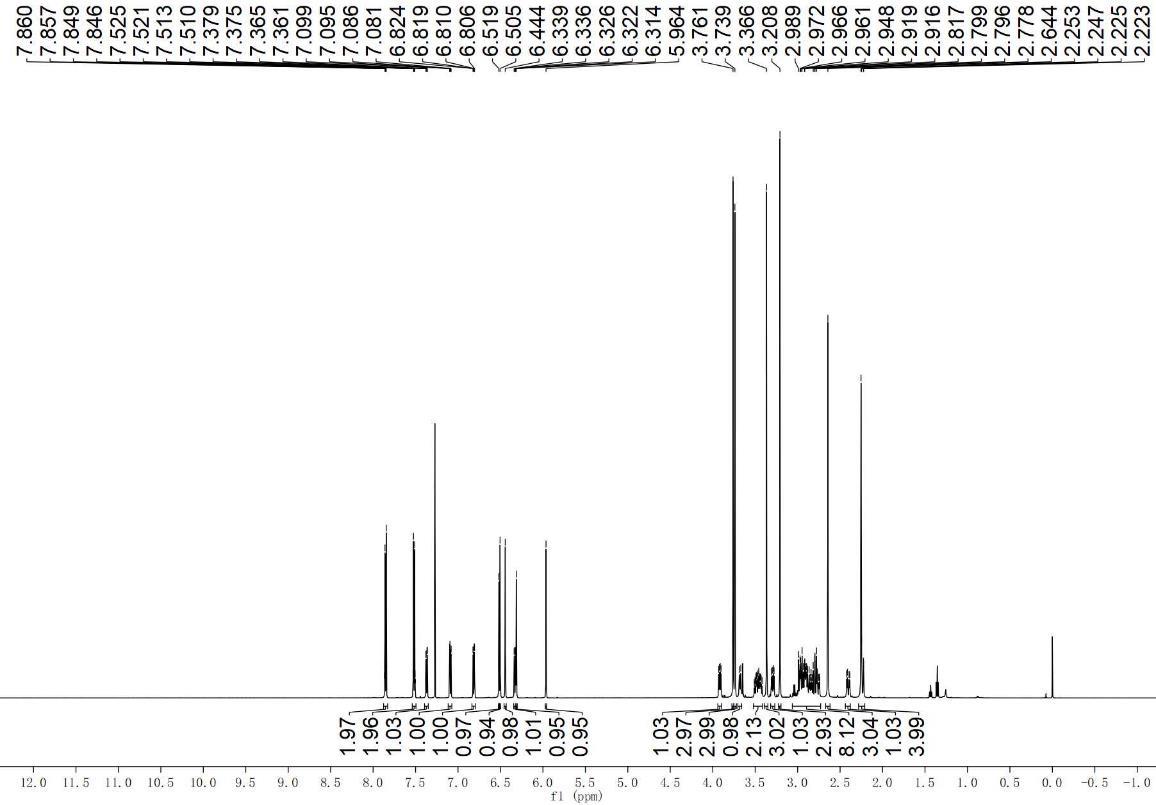


**Compound 8. 14-*O*-(4-chlorobenzenesulfonyl)-tetrandrine: ^13^C-NMR spectrum**


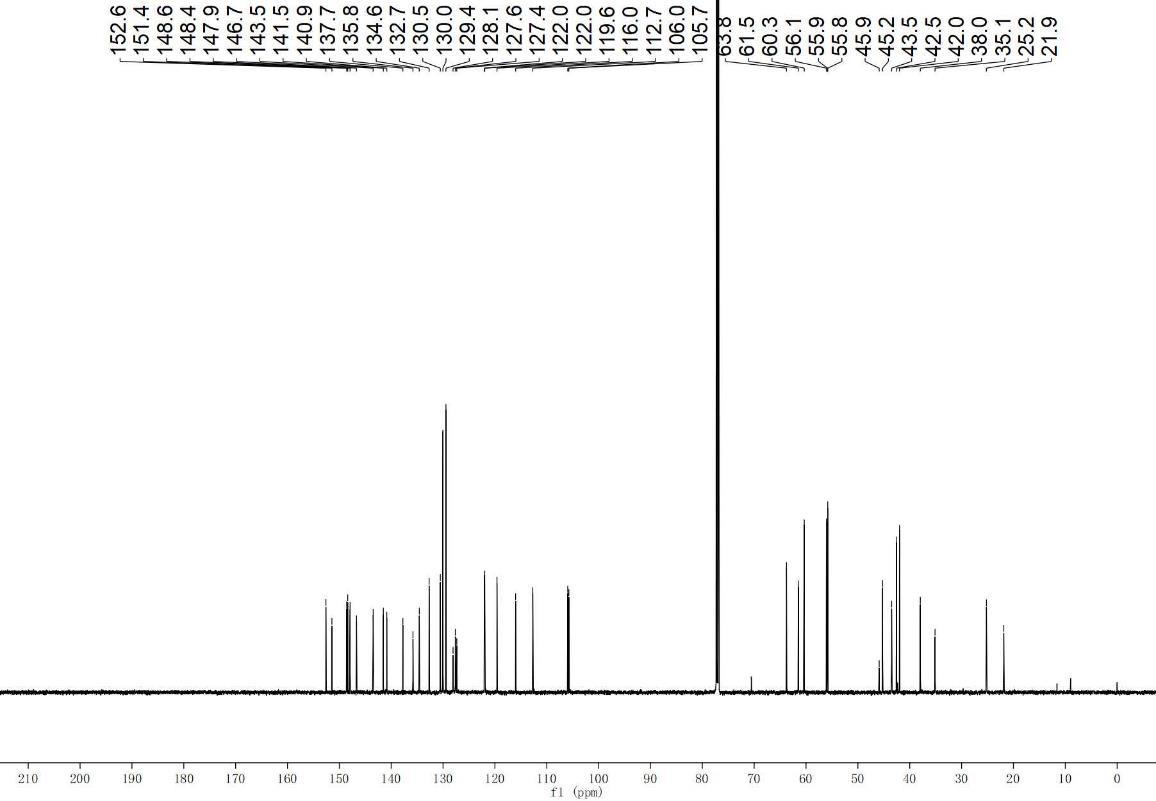


**Compound 8. 14-*O*-(4-chlorobenzenesulfonyl)-tetrandrine: HR-ESIMS spectrum**

**Compound 9.** **14-*O*-(2-bromobenzenesulfonyl)-tetrandrine: ^1^H-NMR spectrum**


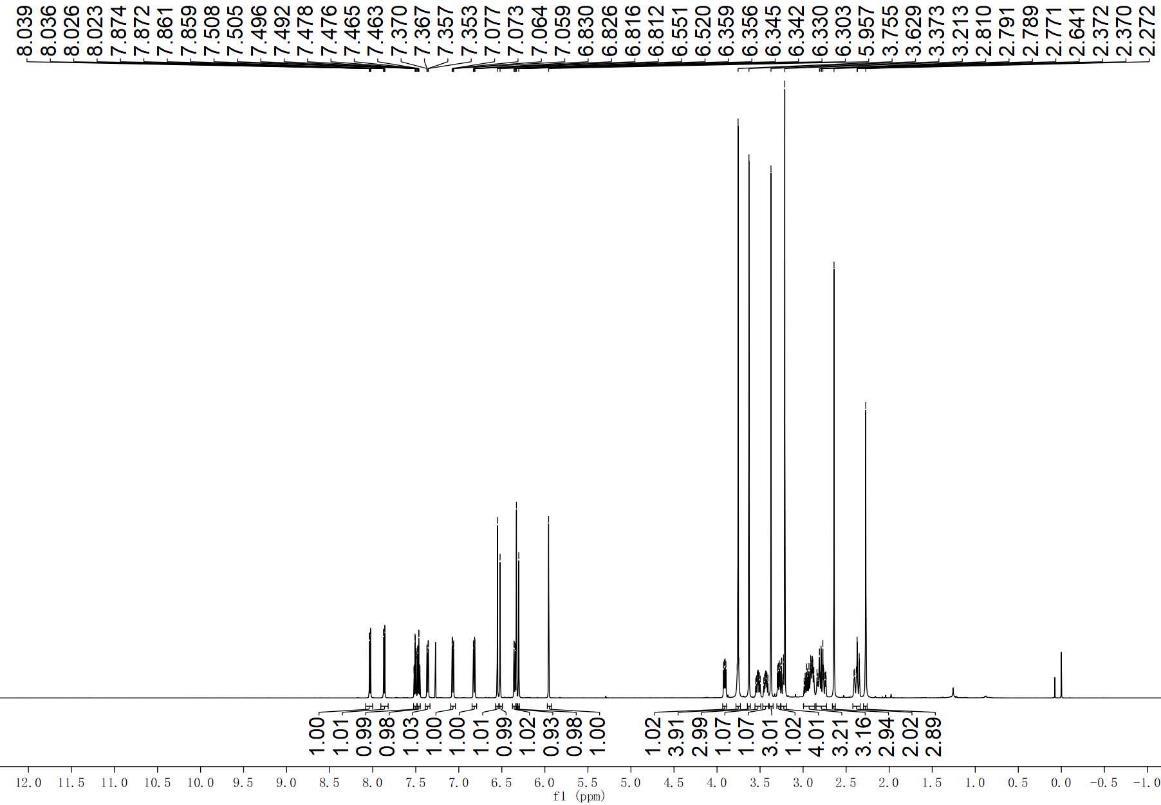


**Compound 9. 14-*O*-(2-bromobenzenesulfonyl)-tetrandrine: ^13^C-NMR spectrum**


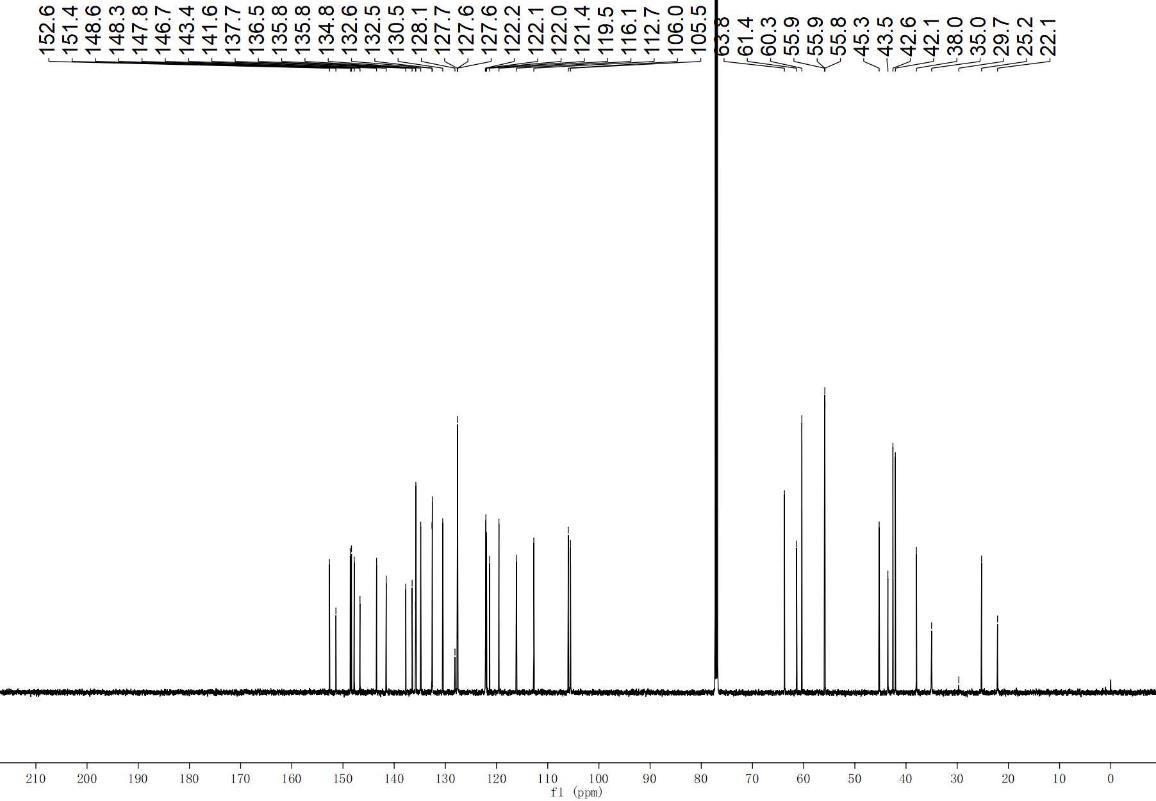


**Compound 9. 14-*O*-(2-bromobenzenesulfonyl)-tetrandrine: HR-ESIMS spectrum**

**Compound 10****. 14-*O*-(4-bromo-benzenesulfonyl)-tetrandrine: ^1^H-NMR spectrum**


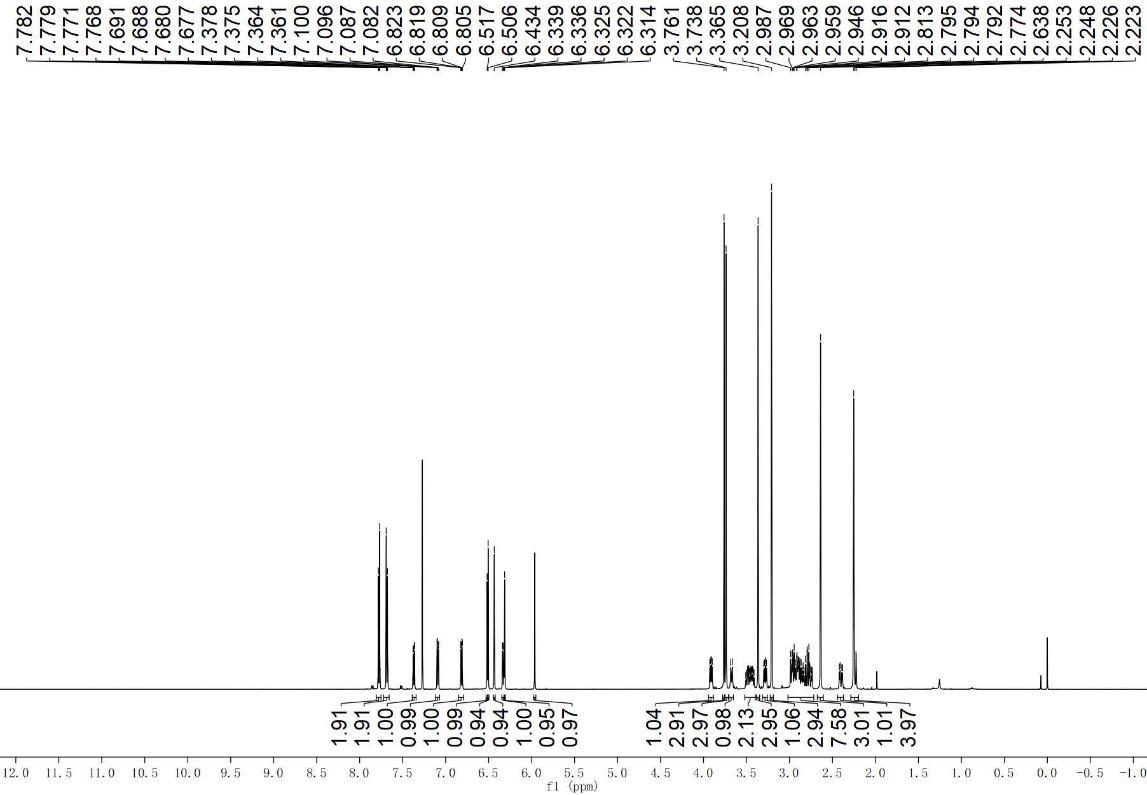


**Compound 10. 14-*O*-(4-bromo-benzenesulfonyl)-tetrandrine: ^13^C-NMR spectrum**


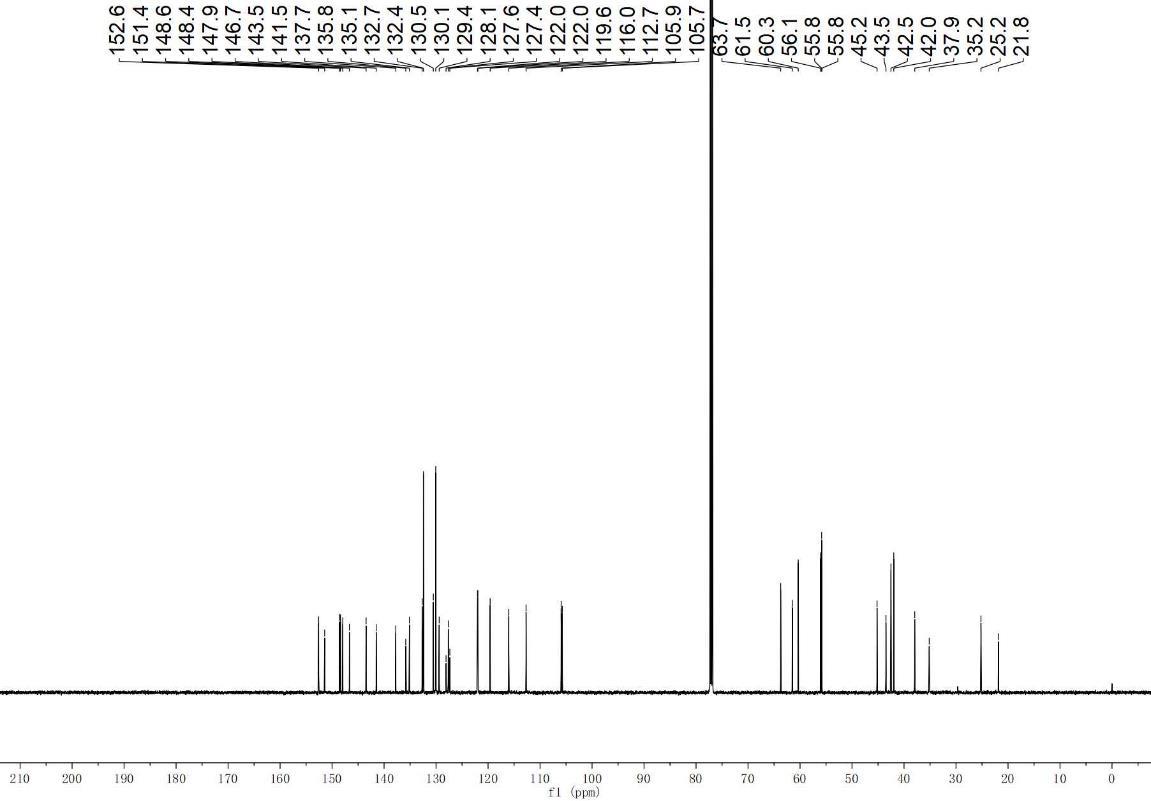


**Compound 10. 14-*O*-(4-bromo-benzenesulfonyl)-tetrandrine: HR-ESIMS spectrum**

**Compound 11****. 14-*O*-(3-nitrobenzenesulfonyl)-tetrandrine: ^1^H-NMR spectrum**


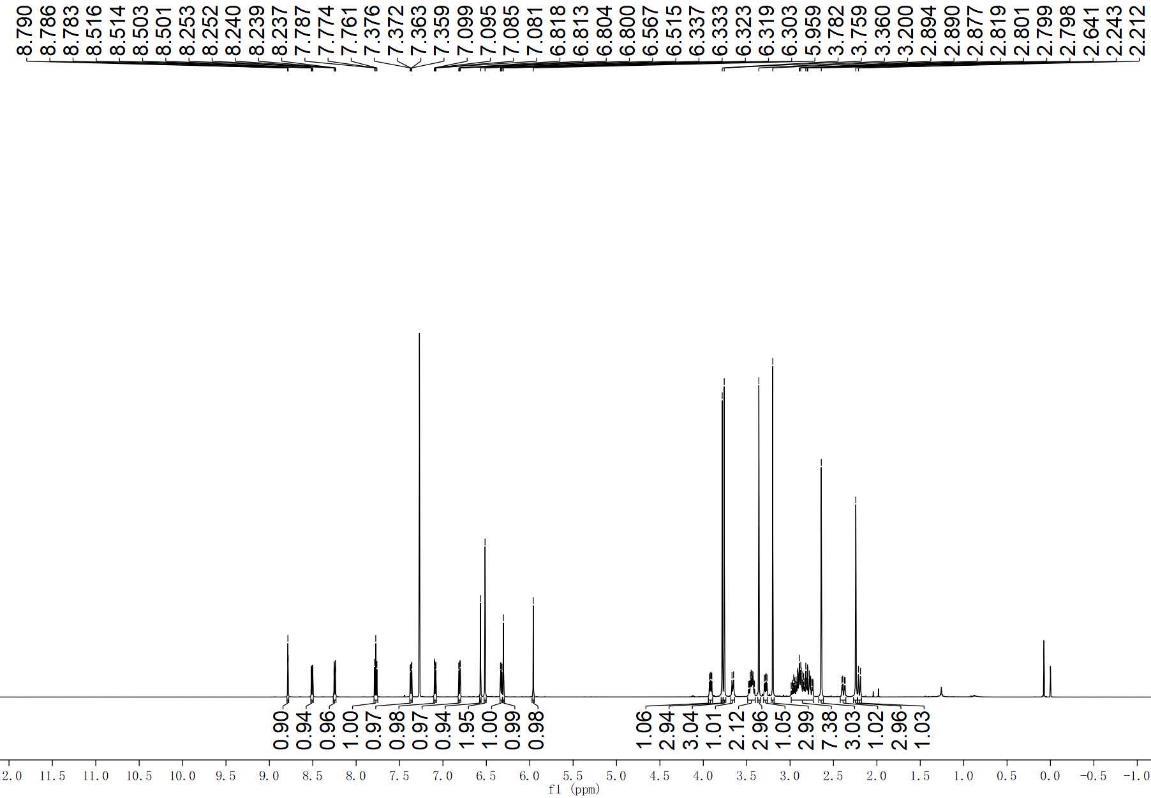


**Compound 11. 14-*O*-(3-nitrobenzenesulfonyl)-tetrandrine: ^13^C-NMR spectrum**


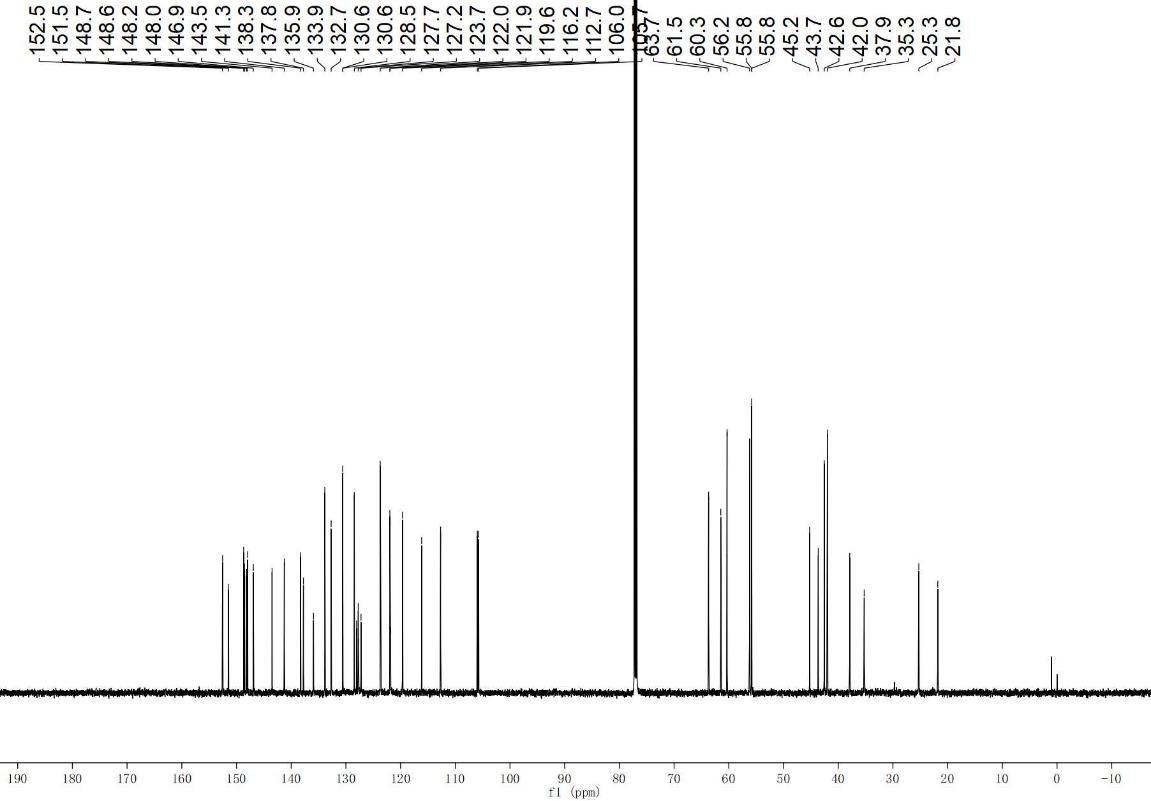


**Compound 11. 14-*O*-(3-nitrobenzenesulfonyl)-tetrandrine: HR-ESIMS spectrum**

**Compound 12****. 14-*O*-(4-nitrobenzenesulfonyl)-tetrandrine: ^1^H-NMR spectrum**


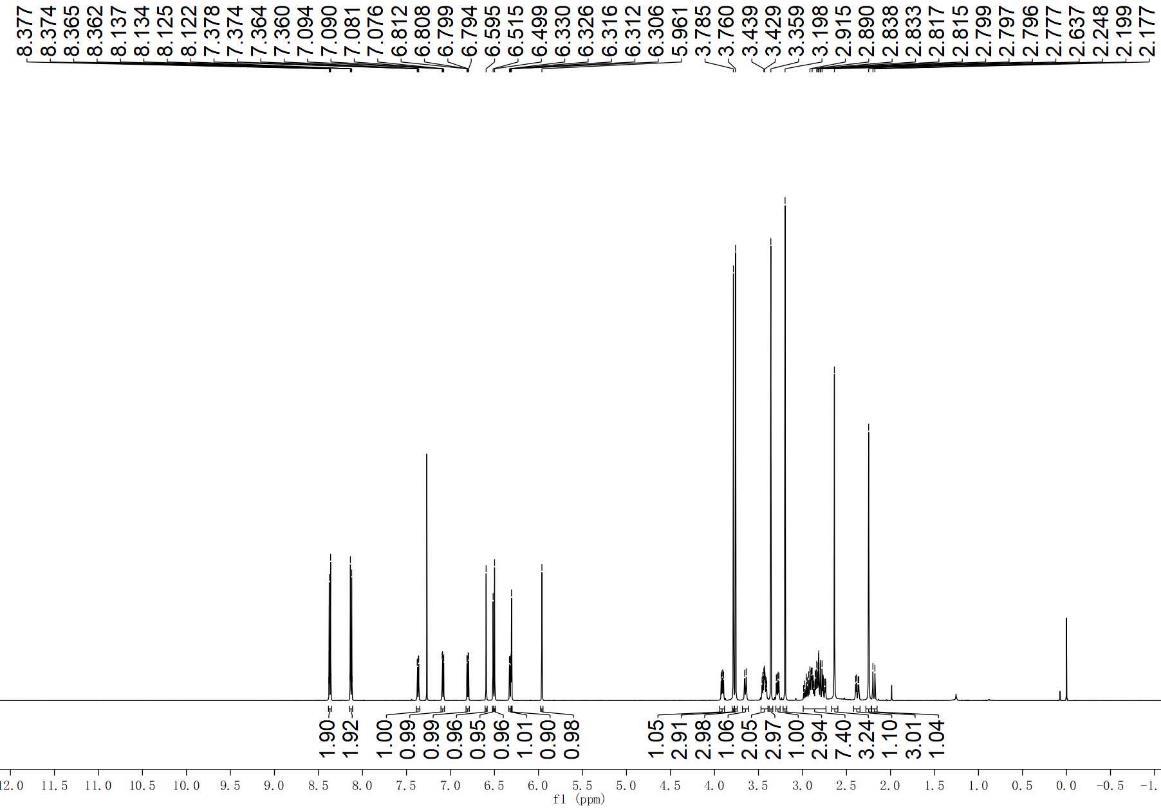


**Compound 12. 14-*O*-(4-nitrobenzenesulfonyl)-tetrandrine: ^13^C-NMR spectrum**


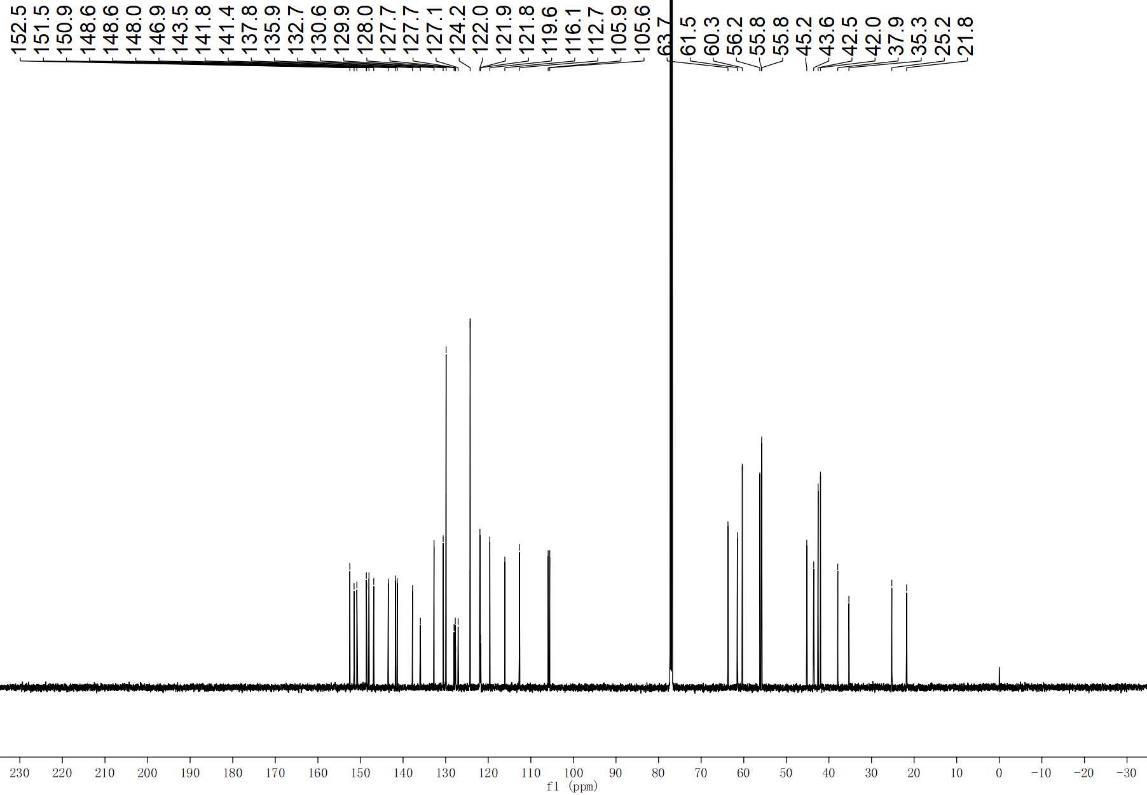


**Compound 12. 14-*O*-(4-nitrobenzenesulfonyl)-tetrandrine: HR-ESIMS spectrum**

**Compound 13** **14-*O*-(3-cyanobenzenesulfonyl)-tetrandrine: ^1^H-NMR spectrum**


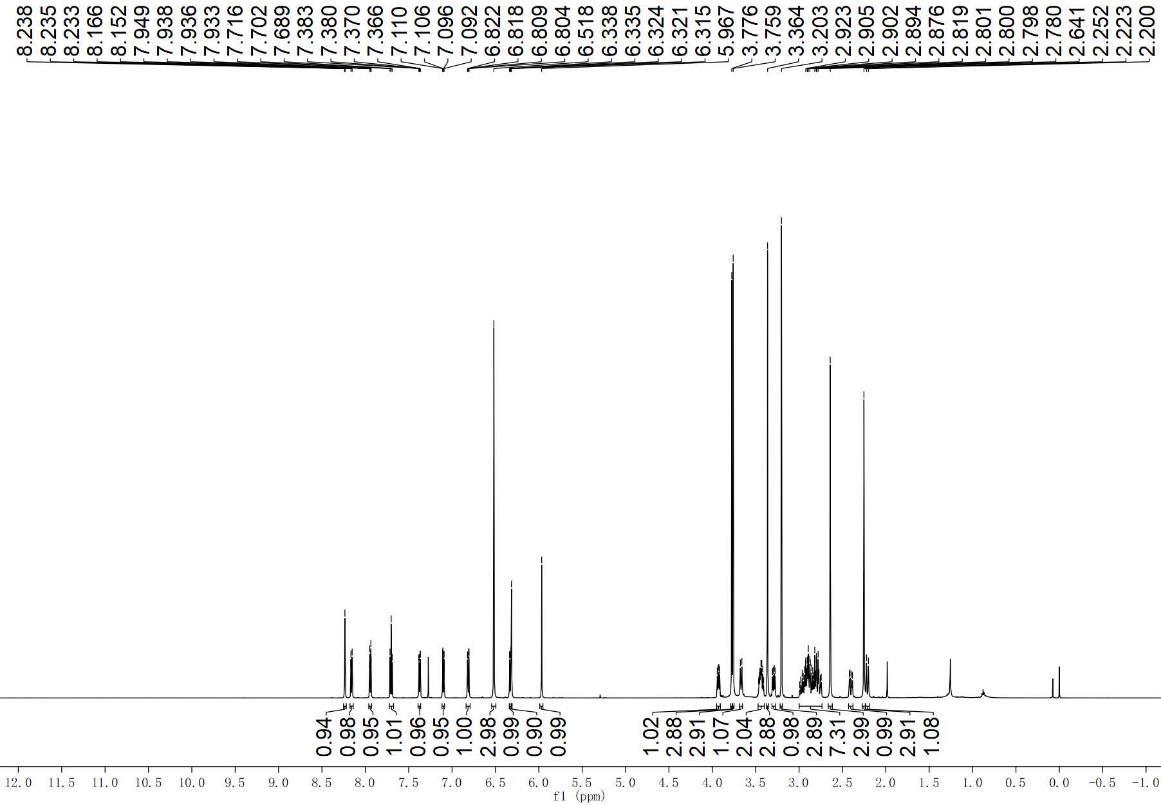


**Compound 13 14-*O*-(3-cyanobenzenesulfonyl)-tetrandrine: ^13^C-NMR spectrum**


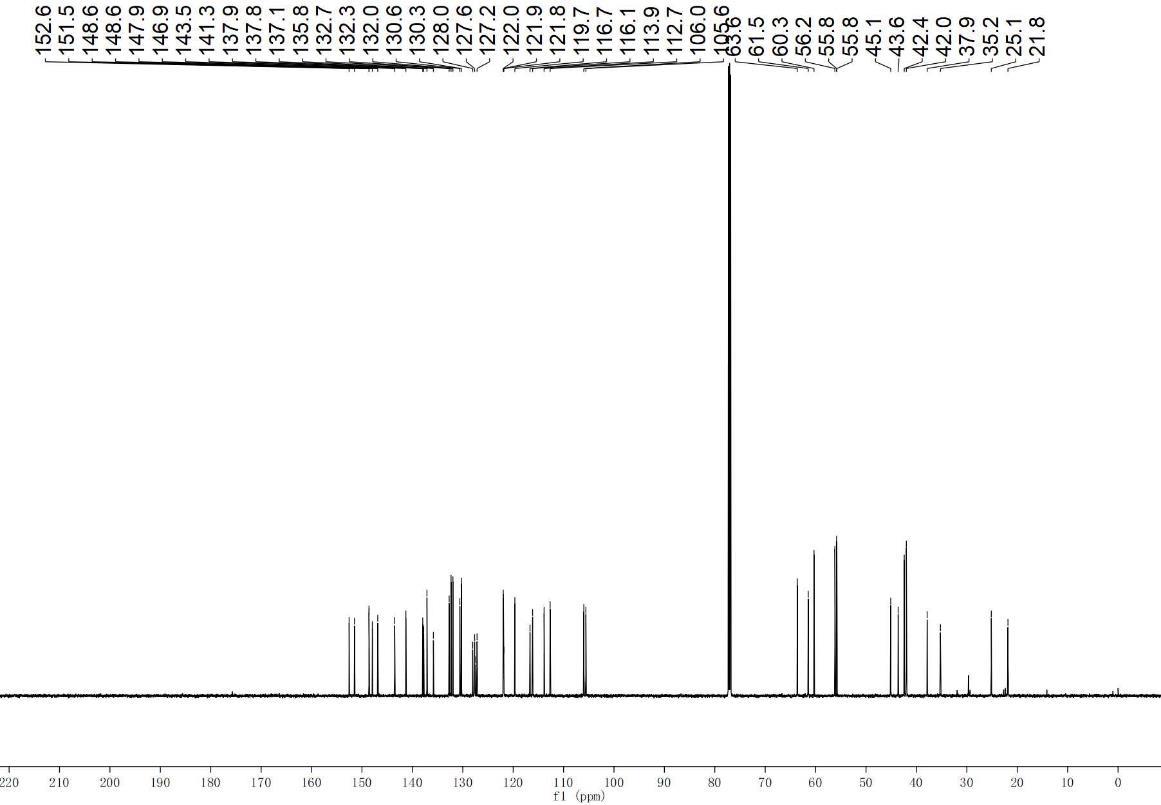


**Compound 13 14-*O*-(3-cyanobenzenesulfonyl)-tetrandrine: HR-ESIMS spectrum**

**Compound 14. 14-*O*-(4-cyanobenzenesulfonyl)-tetrandrine: ^1^H-NMR spectrum**


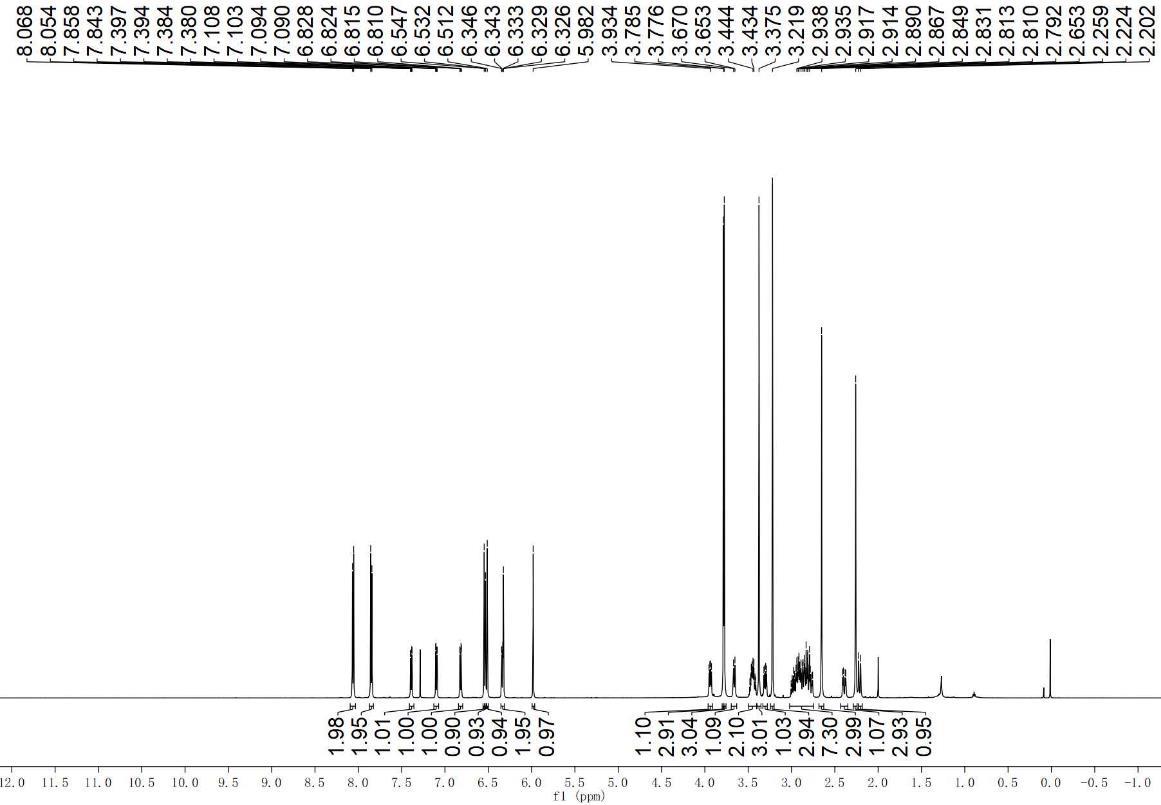


**Compound 14. 14-*O*-(4-cyanobenzenesulfonyl)-tetrandrine: ^13^C-NMR spectrum**


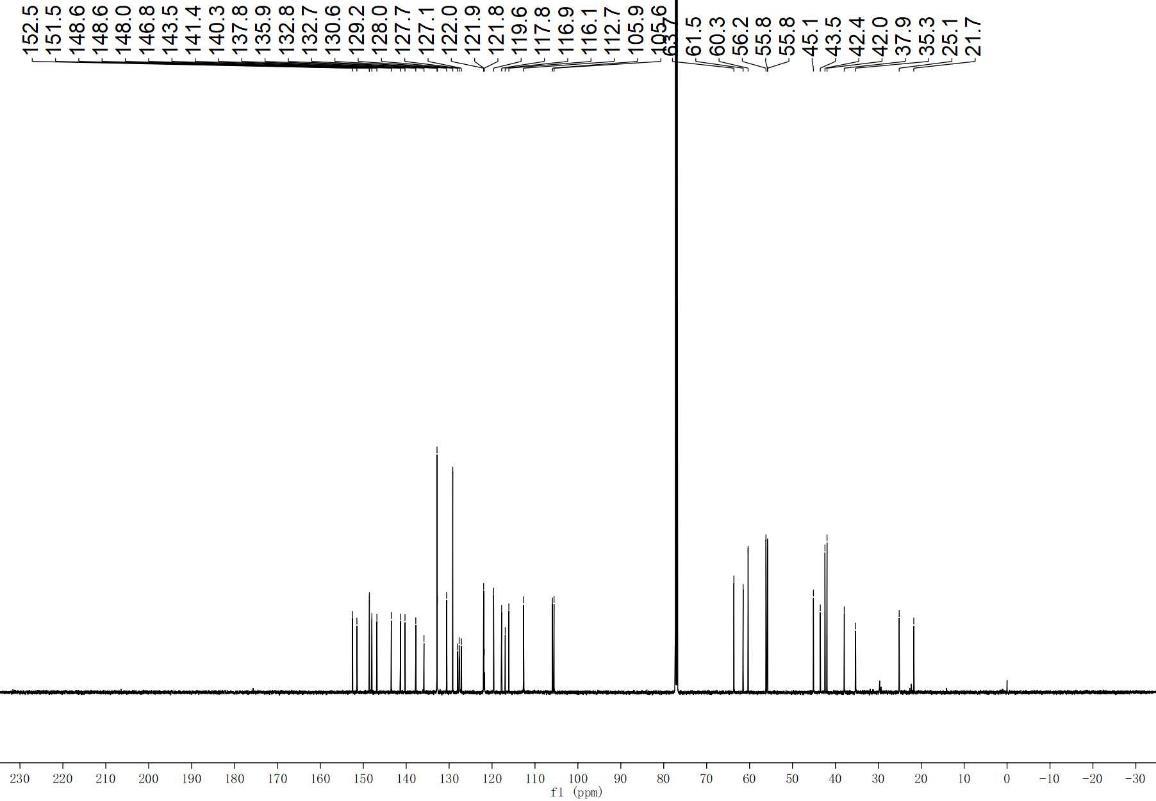


**Compound 14. 14-*O*-(4-cyanobenzenesulfonyl)-tetrandrine: HR-ESIMS spectrum**

**Compound 15****. 14-*O*-(2-(trifluoromethyl)benzenesulfonyl)-tetrandrine: ^1^H-NMR spectrum**


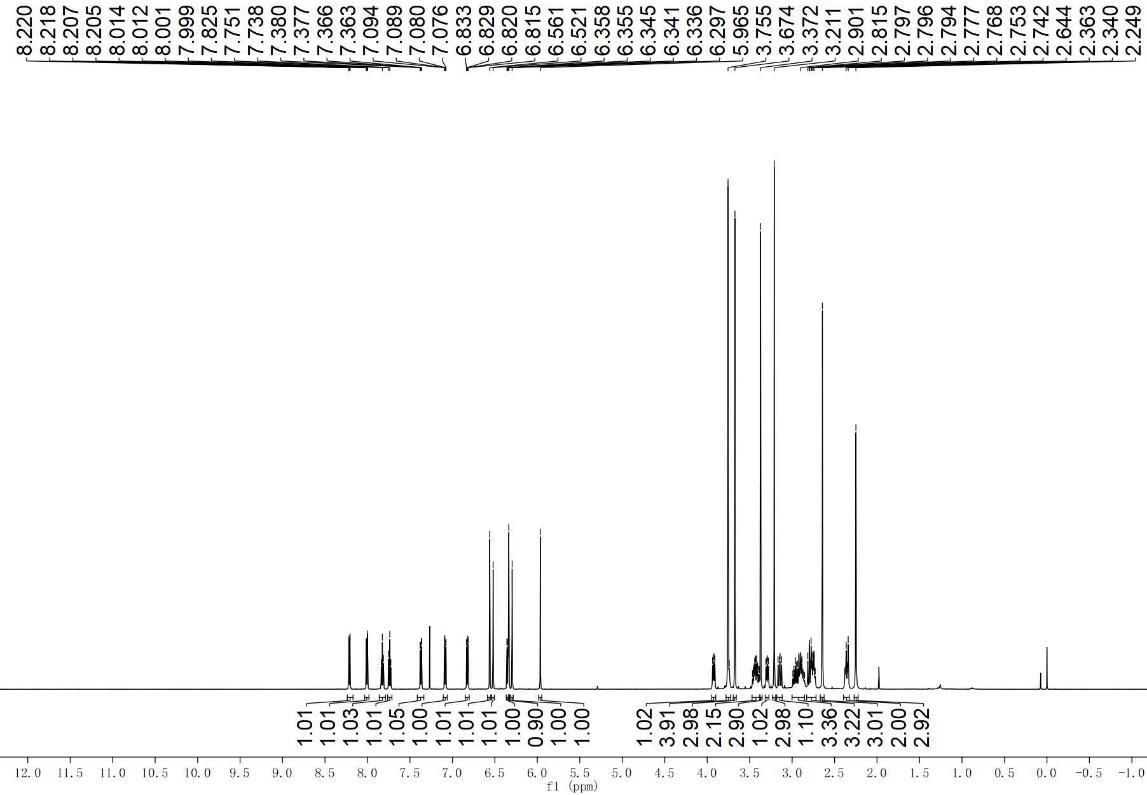


**Compound 15. 14-*O*-(2-(trifluoromethyl)benzenesulfonyl)-tetrandrine: ^13^C-NMR spectrum**


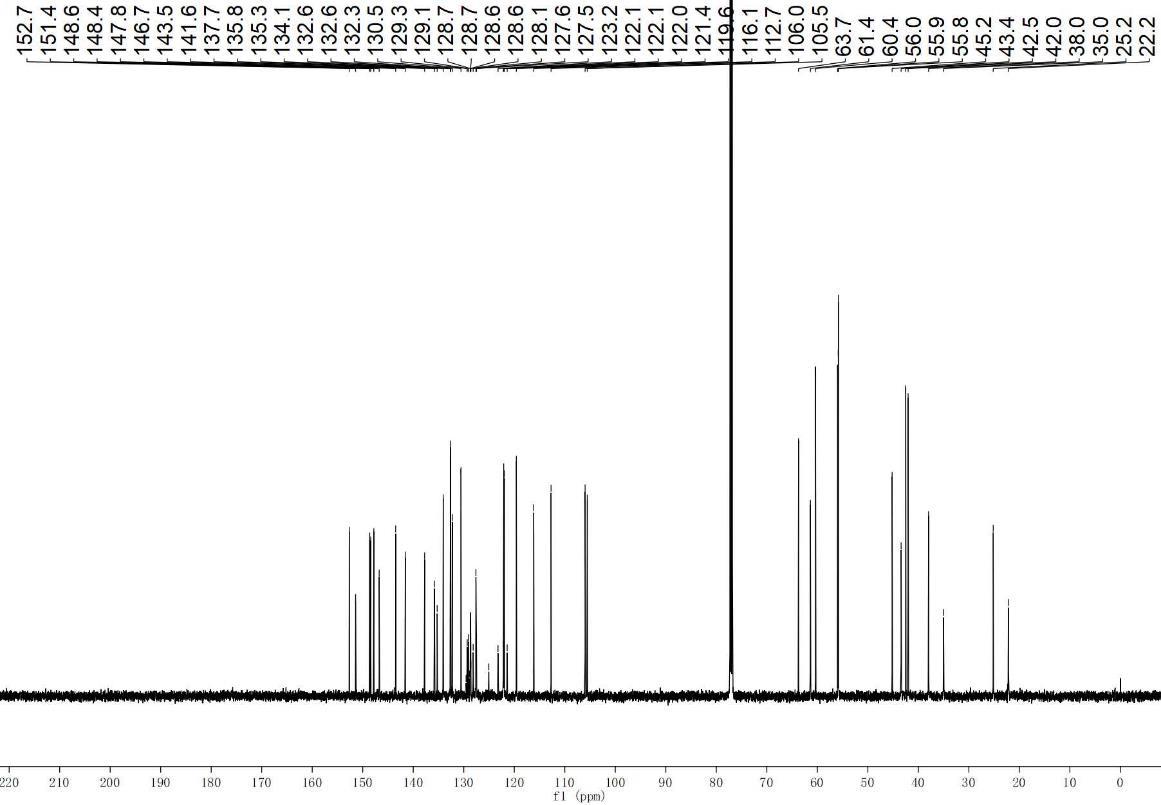


**Compound 15. 14-*O*-(2-(trifluoromethyl)benzenesulfonyl)-tetrandrine: ^19^F-NMR spectrum**


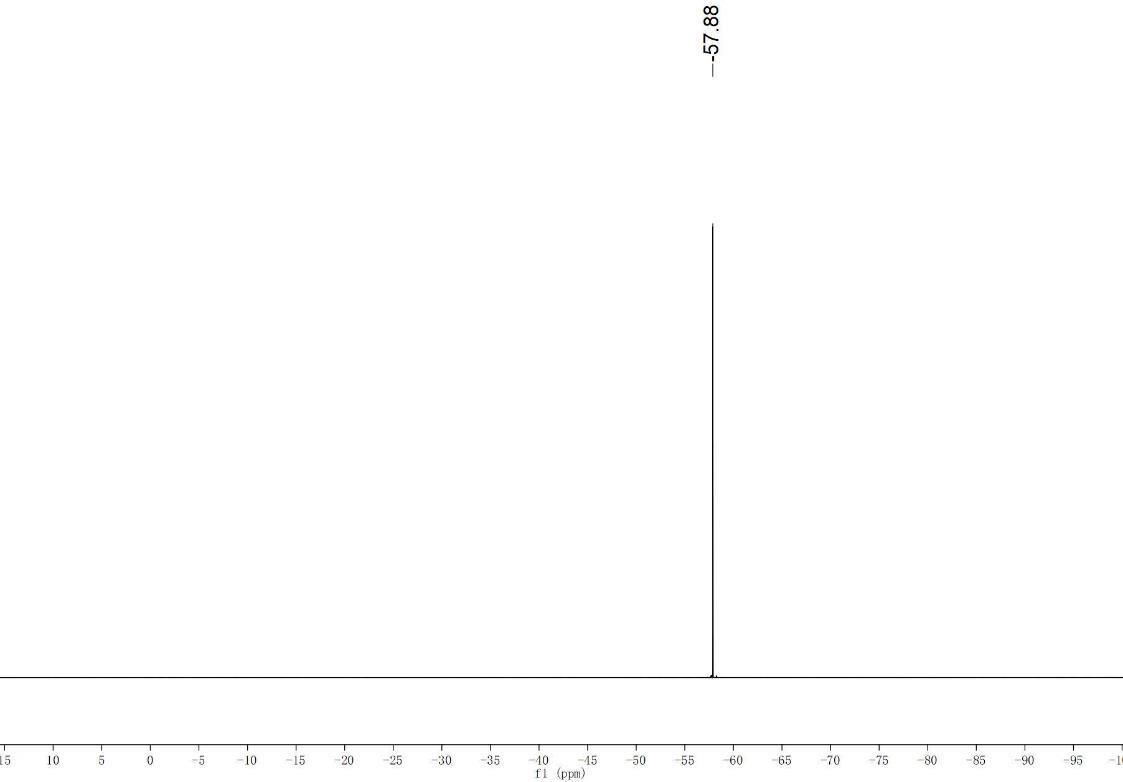


**Compound 15. 14-*O*-(2-(trifluoromethyl)benzenesulfonyl)-tetrandrine: HR-ESIMS spectrum**

**Compound 16****. 14-*O*-(3-(trifluoromethyl)benzenesulfonyl)-tetrandrine: ^1^H-NMR spectrum**


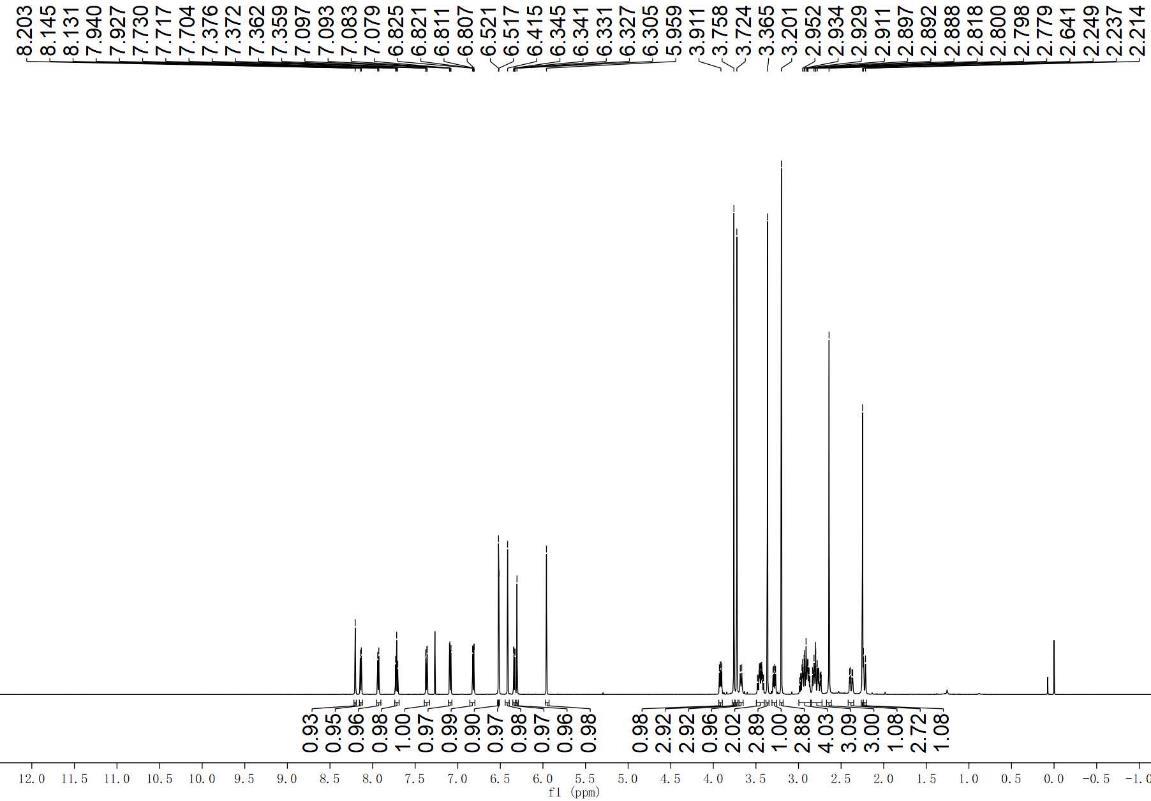


**Compound 16. 14-*O*-(3-(trifluoromethyl)benzenesulfonyl)-tetrandrine: ^13^C-NMR spectrum**


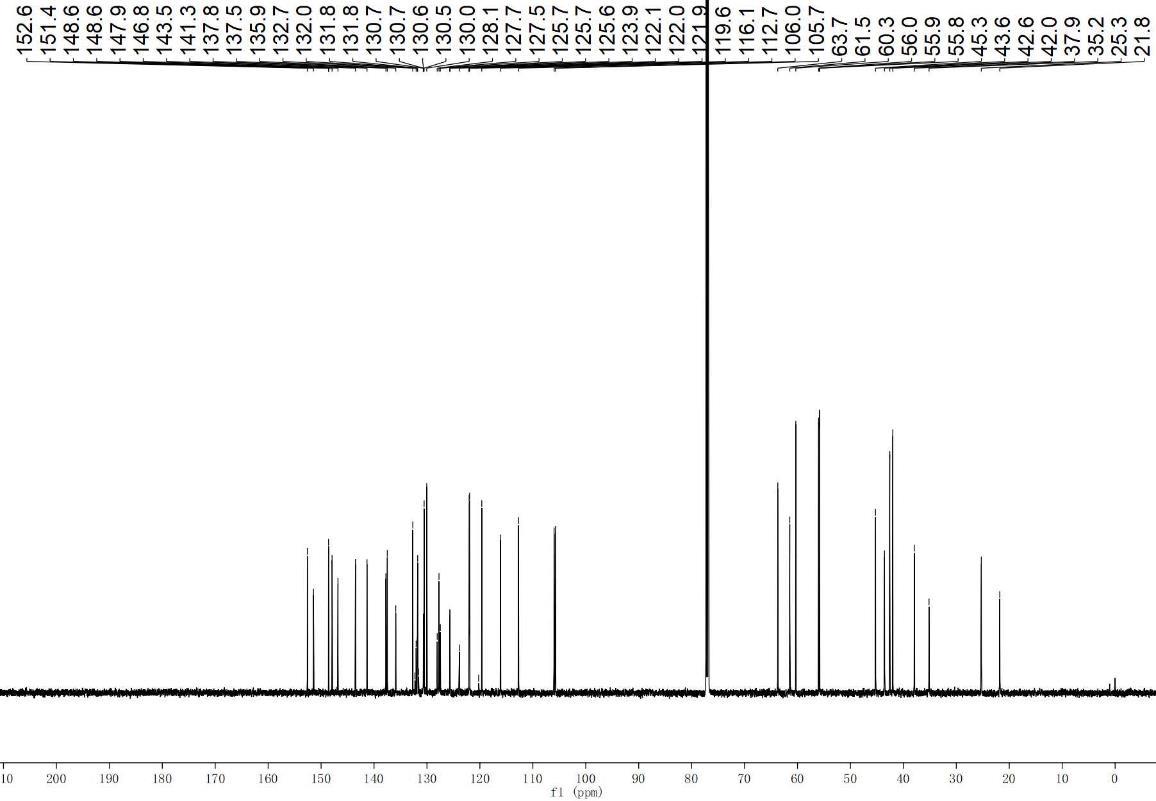


**Compound 16. 14-*O*-(3-(trifluoromethyl)benzenesulfonyl)-tetrandrine: ^19^F-NMR spectrum**


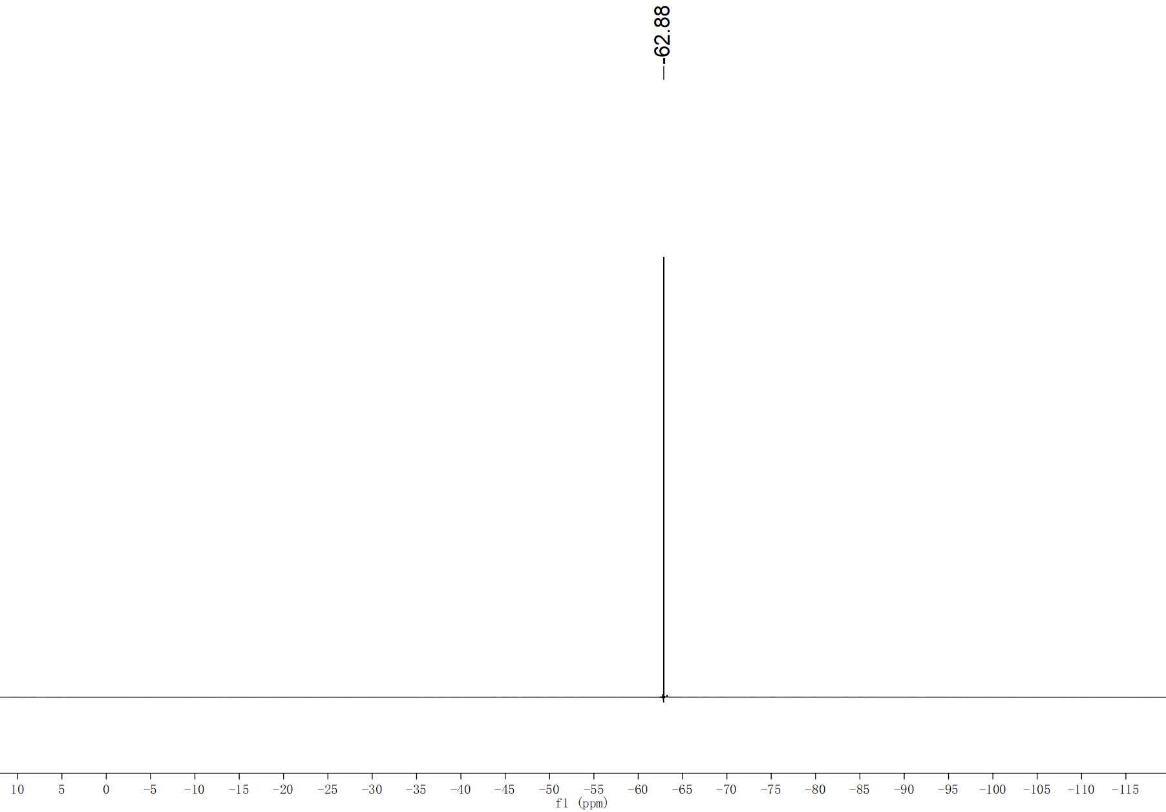


**Compound 16. 14-*O*-(3-(trifluoromethyl)benzenesulfonyl)-tetrandrine: HR-ESIMS spectrum**

**Compound 17.** **14-*O*-(4-(trifluoromethyl)benzene-1-sulfonyl)-tetrandrine: ^1^H-NMR spectrum**


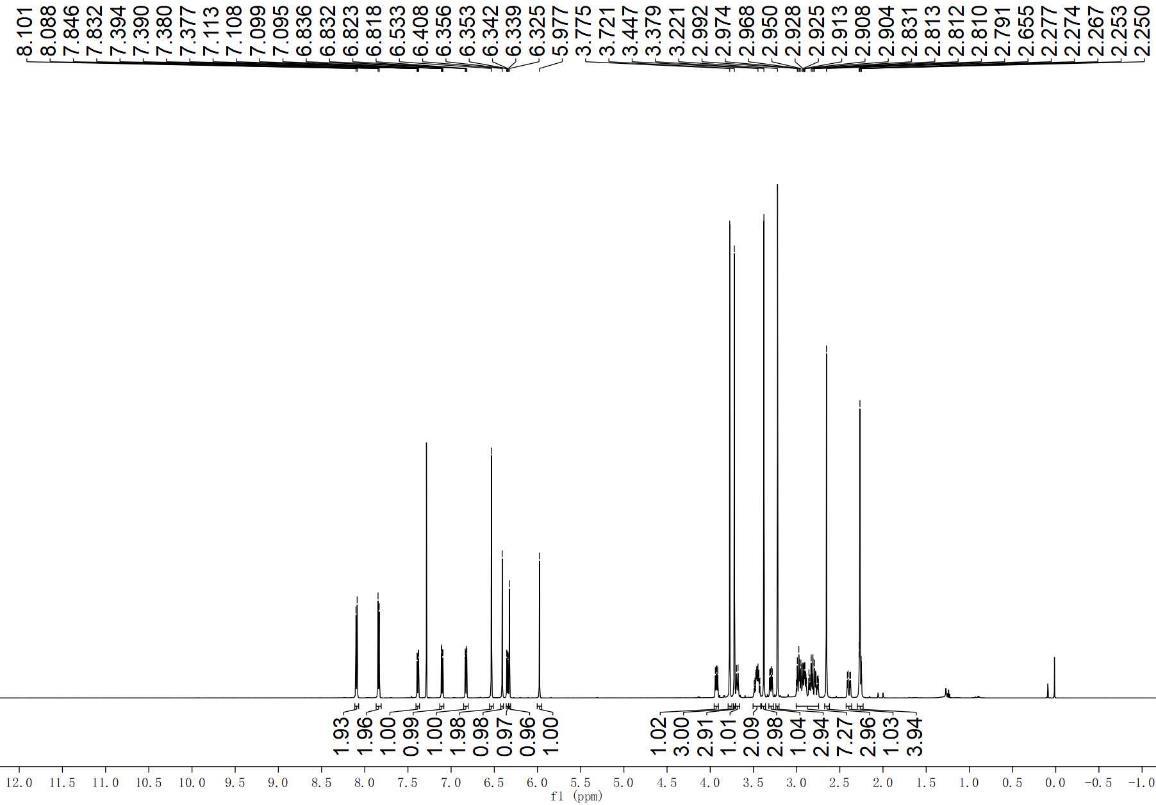


**Compound 17. 14-*O*-(4-(trifluoromethyl)benzene-1-sulfonyl)-tetrandrine: ^13^C-NMR spectrum**


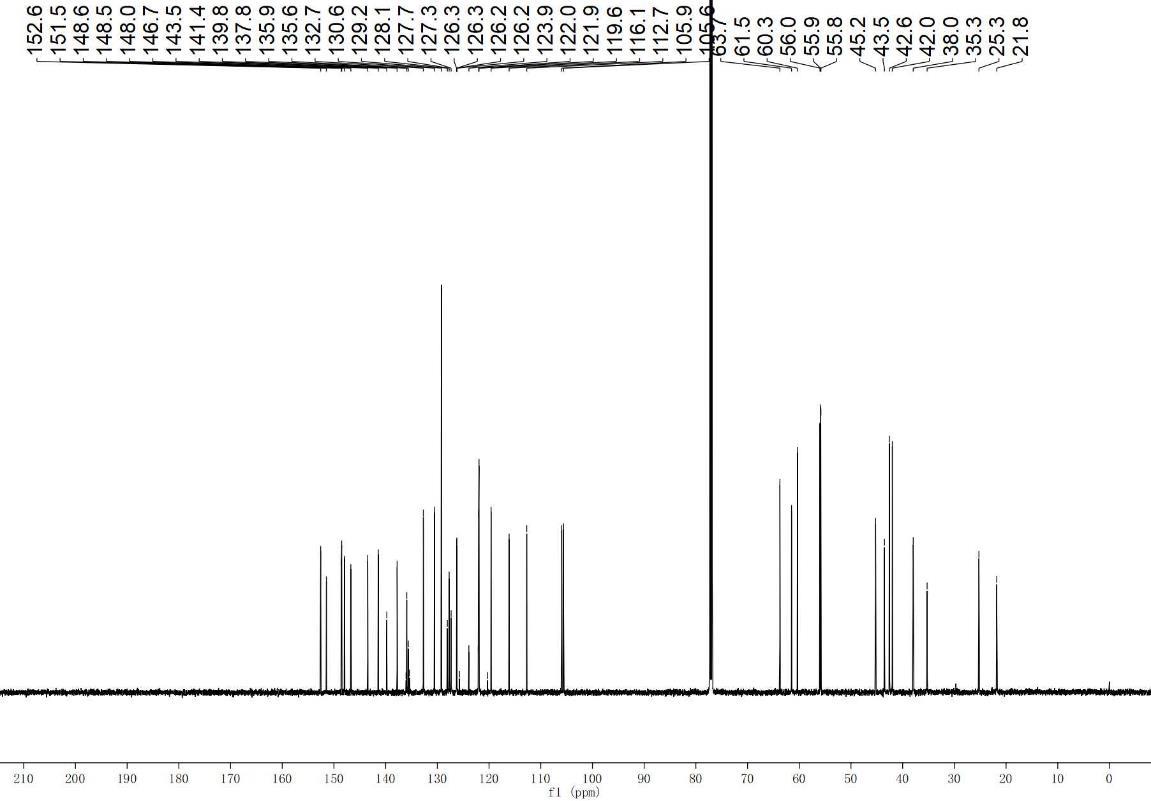


**Compound 17. 14-*O*-(4-(trifluoromethyl)benzene-1-sulfonyl)-tetrandrine: ^19^F-NMR spectrum**


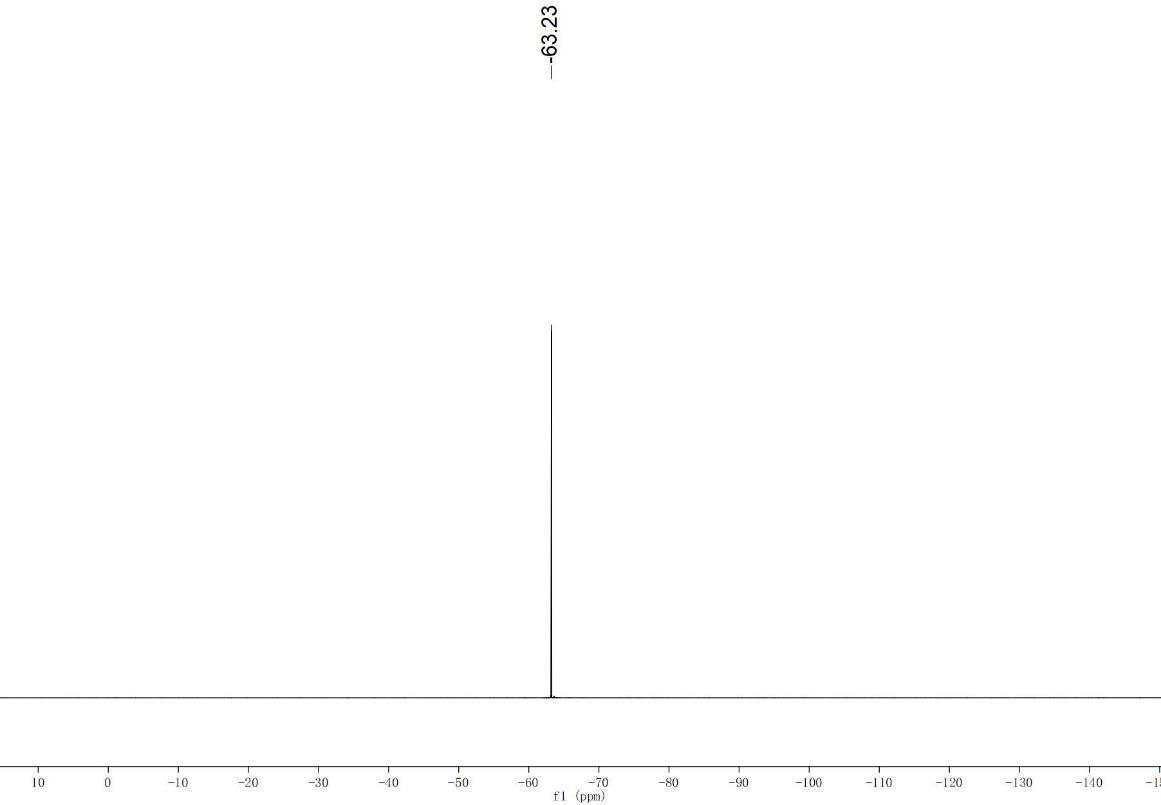


**Compound 17. 14-*O*-(4-(trifluoromethyl)benzene-1-sulfonyl)-tetrandrine: HR-ESIMS spectrum**

**Compound 18.** **14-*O*-(4-(Trifluoromethoxy)benzenesulfonyl)-tetrandrine: ^1^H-NMR spectrum**


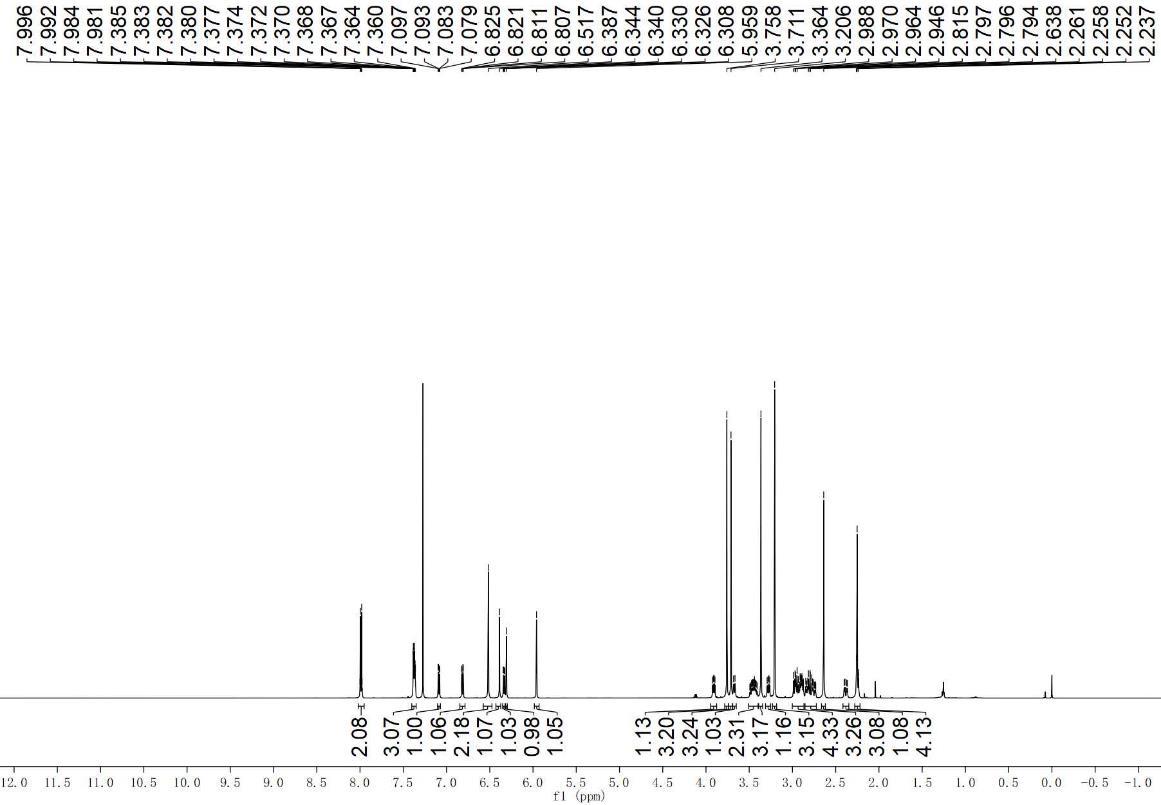


**Compound 18. 14-*O*-(4-(Trifluoromethoxy)benzenesulfonyl)-tetrandrine: ^13^C-NMR spectrum**


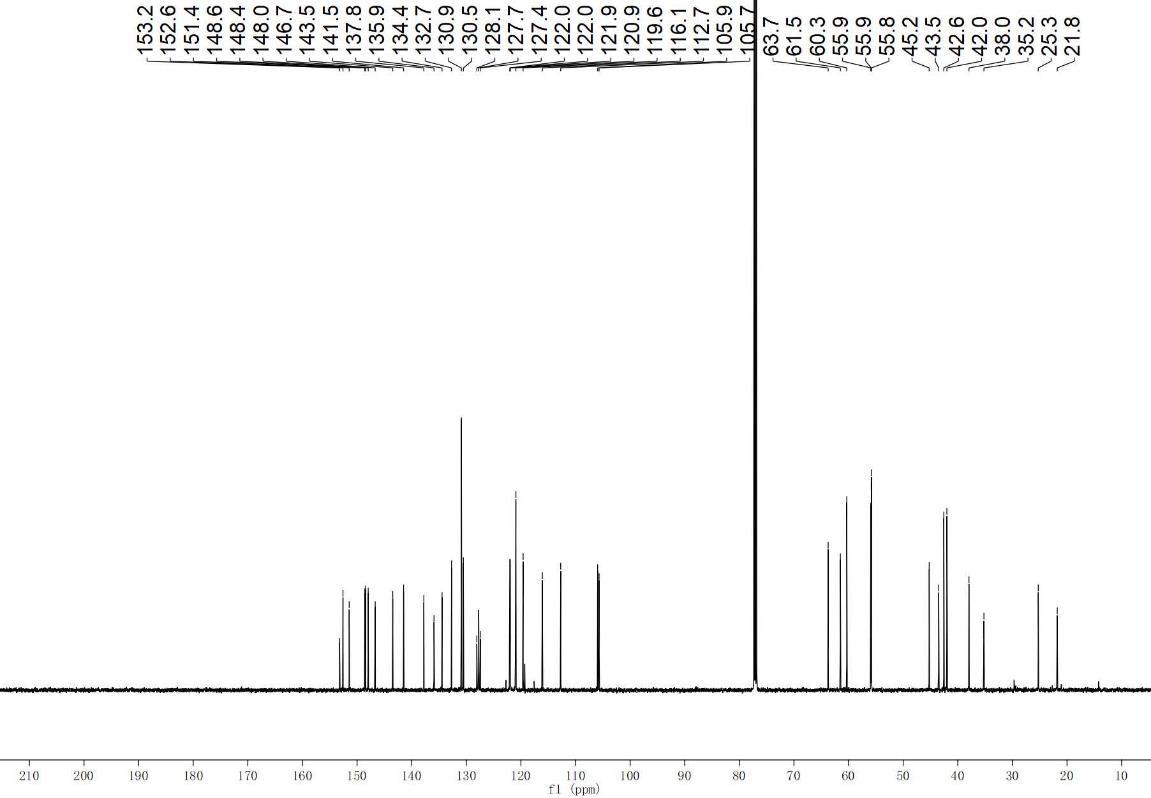


**Compound 18. 14-*O*-(4-(Trifluoromethoxy)benzenesulfonyl)-tetrandrine: ^19^F-NMR spectrum**


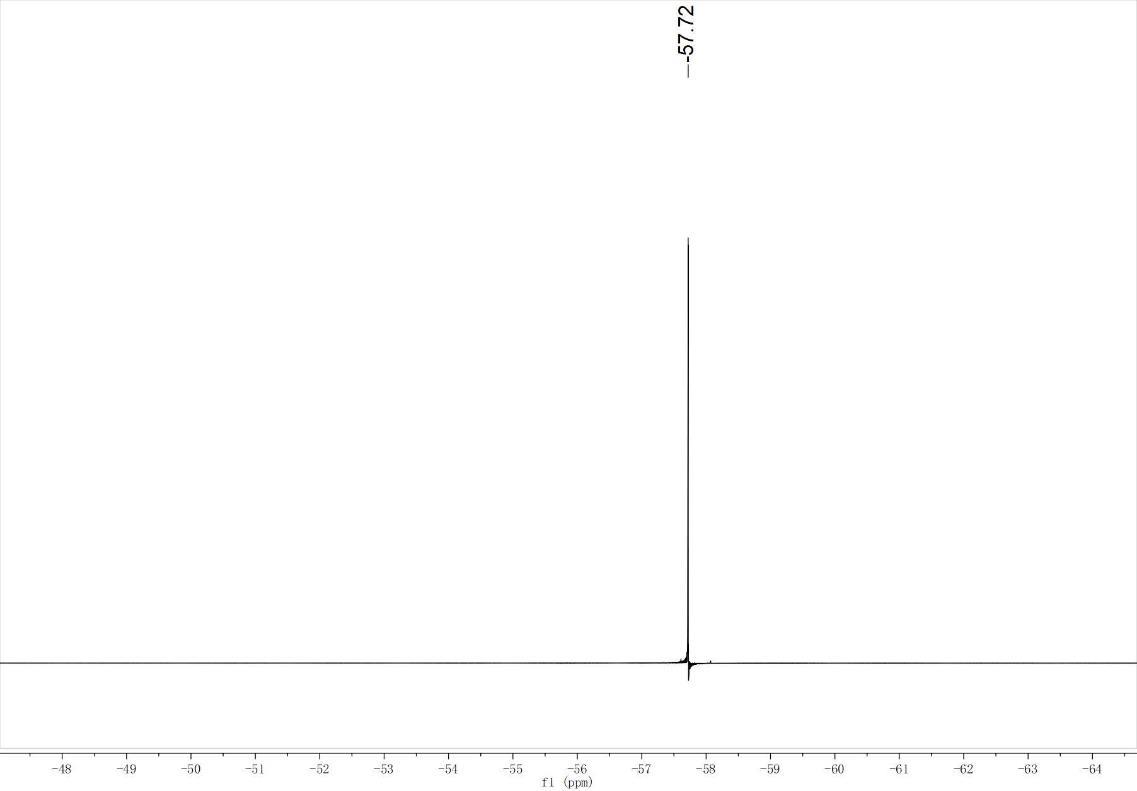


**Compound 18. 14-*O*-(4-(Trifluoromethoxy)benzenesulfonyl)-tetrandrine: HR-ESIMS spectrum**

**Compound 19.** **14-*O*-(4-Toluenesulfonyl)-tetrandrine: ^1^H-NMR spectrum**


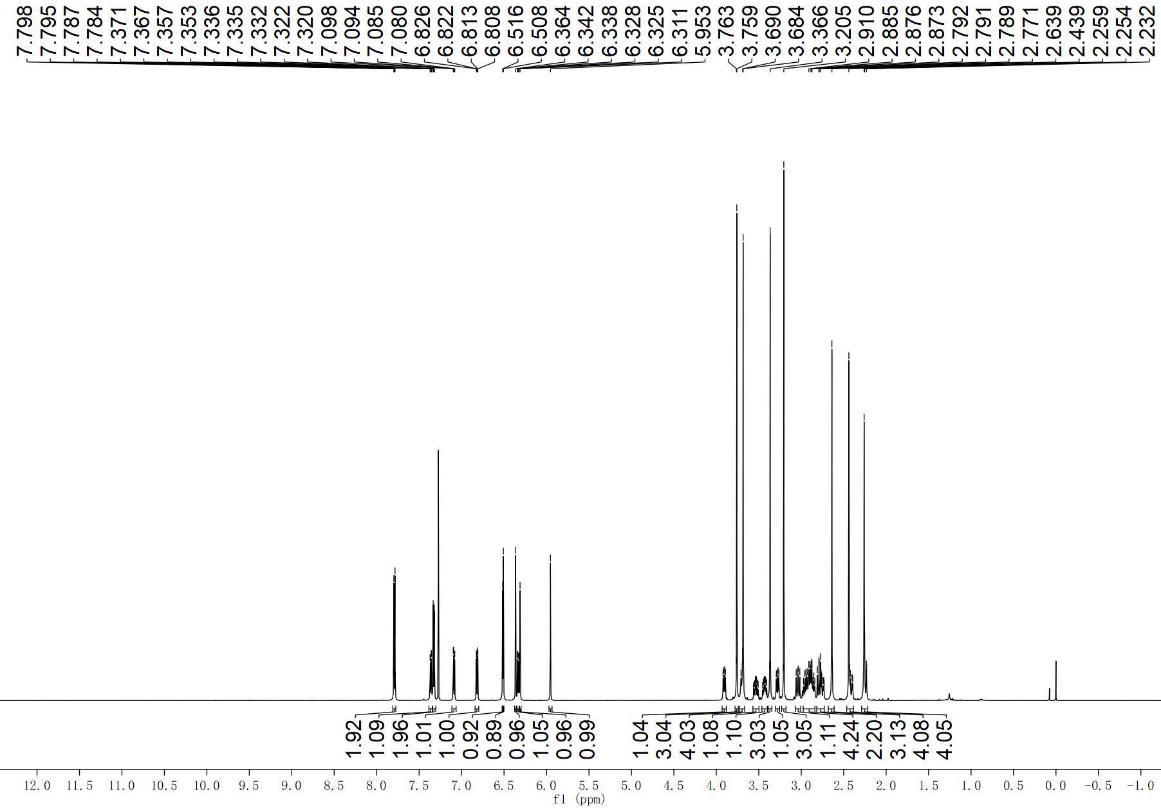


**Compound 19. 14-*O*-(4-Toluenesulfonyl)-tetrandrine: ^13^C-NMR spectrum**


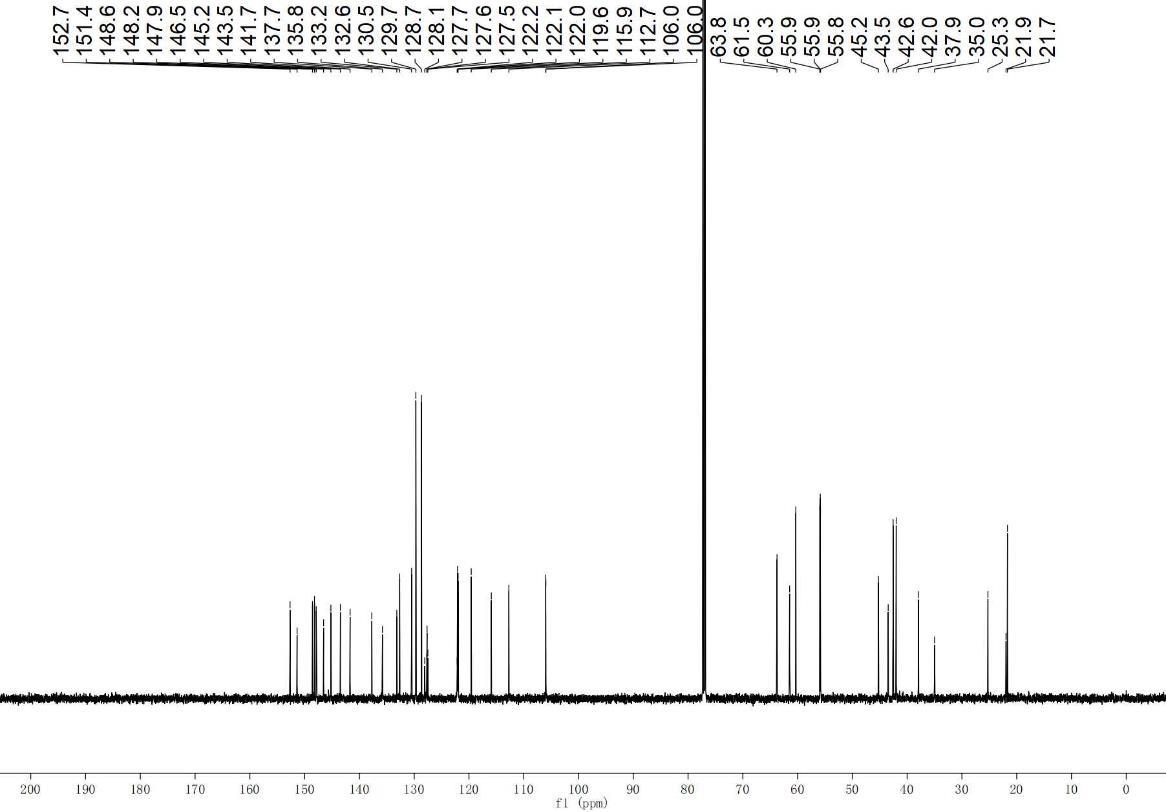


**Compound 19. 14-*O*-(4-Toluenesulfonyl)-tetrandrine: HR-ESIMS spectrum**

**Compound 20.** **14-*O*-(4-methoxybenzenesulfonyl)-tetrandrine: ^1^H-NMR spectrum**


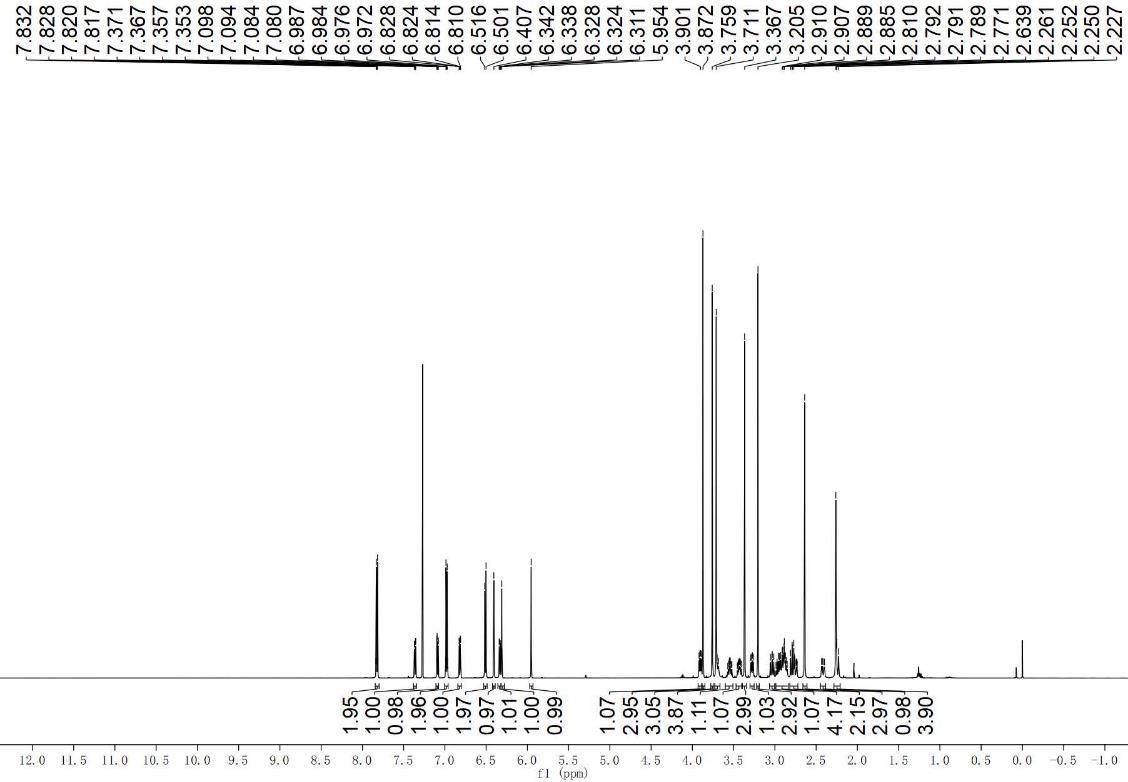


**Compound 20. 14-*O*-(4-methoxybenzenesulfonyl)-tetrandrine: ^13^C-NMR spectrum**


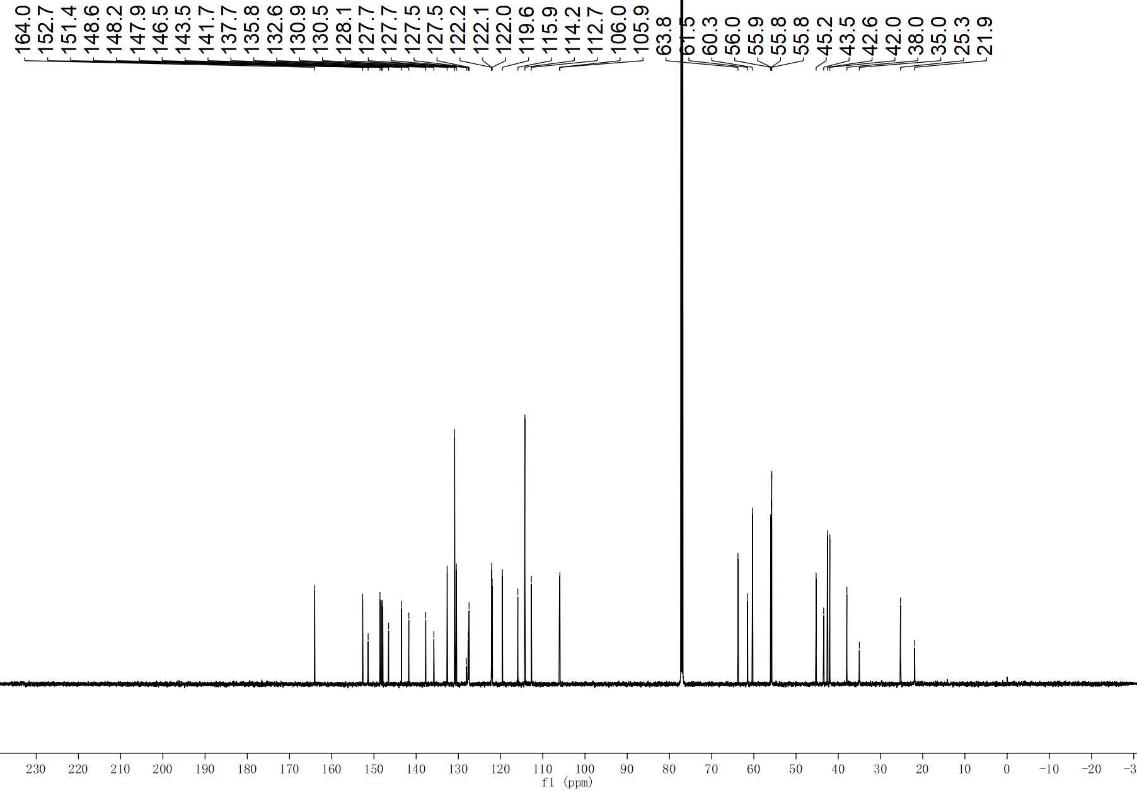


**Compound 20. 14-*O*-(4-methoxybenzenesulfonyl)-tetrandrine: HR-ESIMS spectrum**

**Compound 21.** **14-*O*-(4-tert-butylbenzenesulfonyl)-tetrandrine: ^1^H-NMR spectrum**


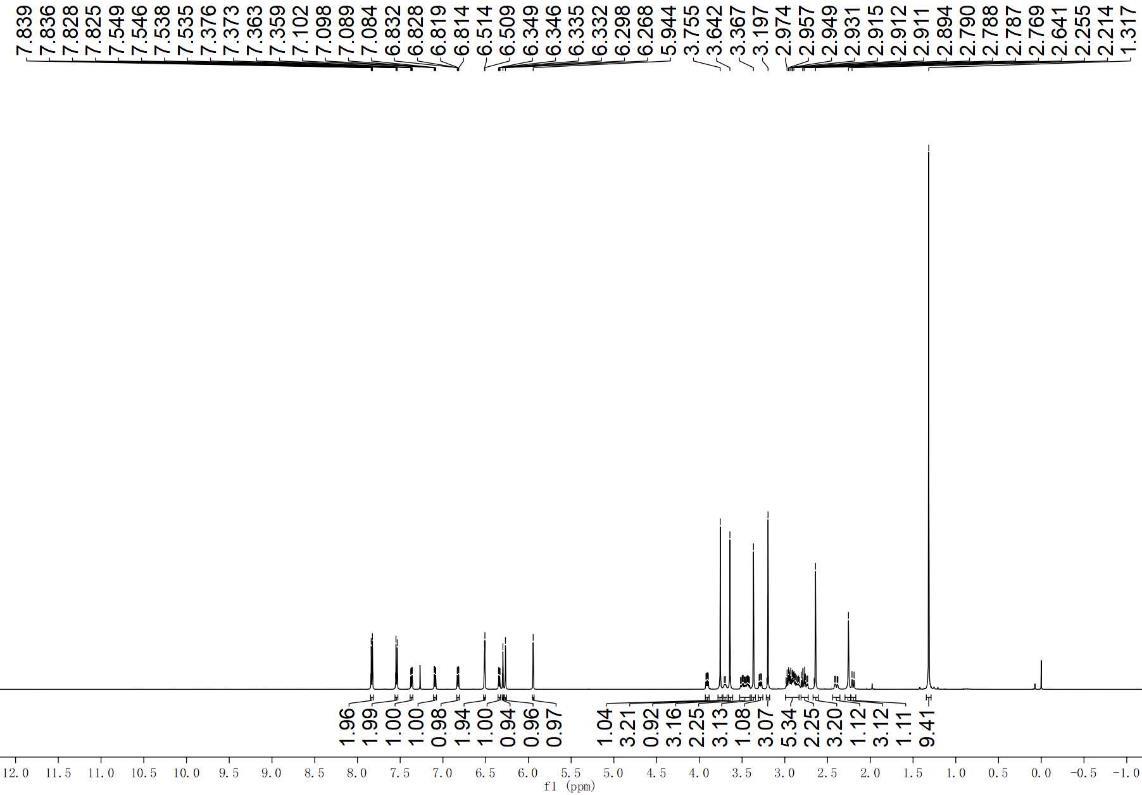


**Compound 21. 14-*O*-(4-tert-butylbenzenesulfonyl)-tetrandrine: ^13^C-NMR spectrum**


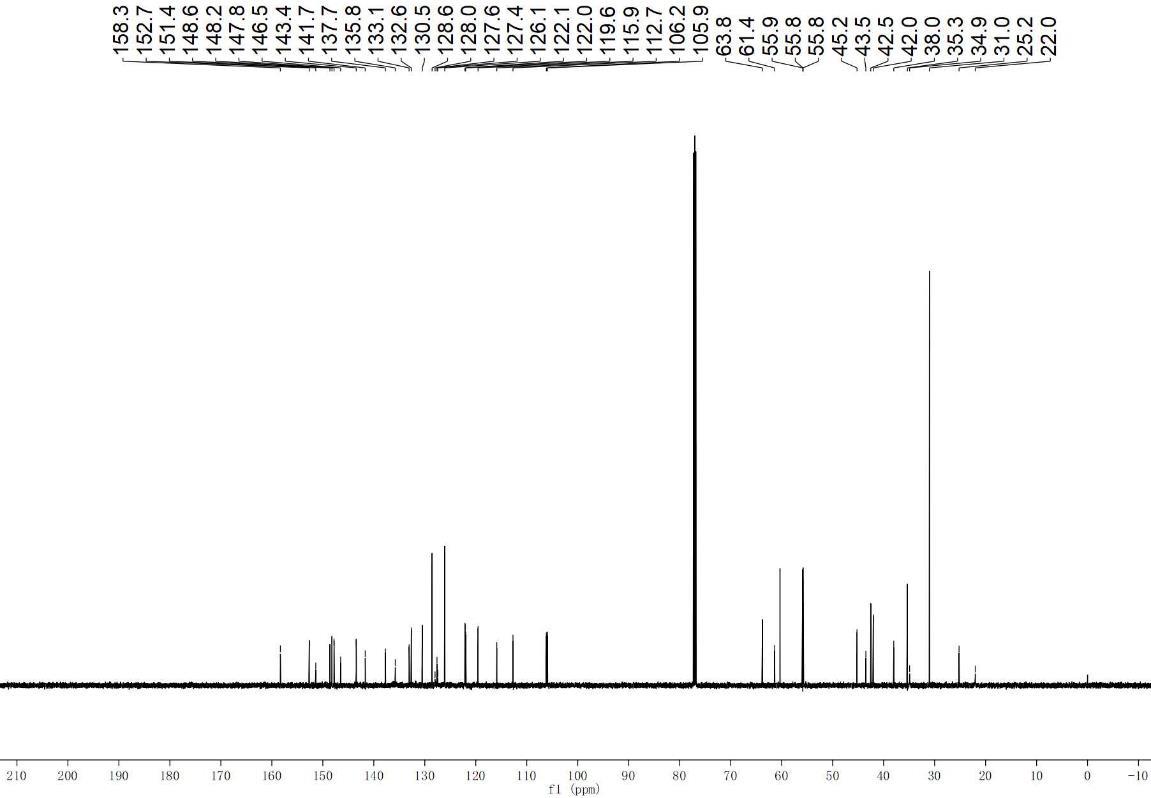


**Compound 21. 14-*O*-(4-tert-butylbenzenesulfonyl)-tetrandrine: HR-ESIMS spectrum**

**Compound 22.** **14-*O*-(4-acetamidobenzenesulfonyl)-tetrandrine: ^1^H-NMR spectrum**


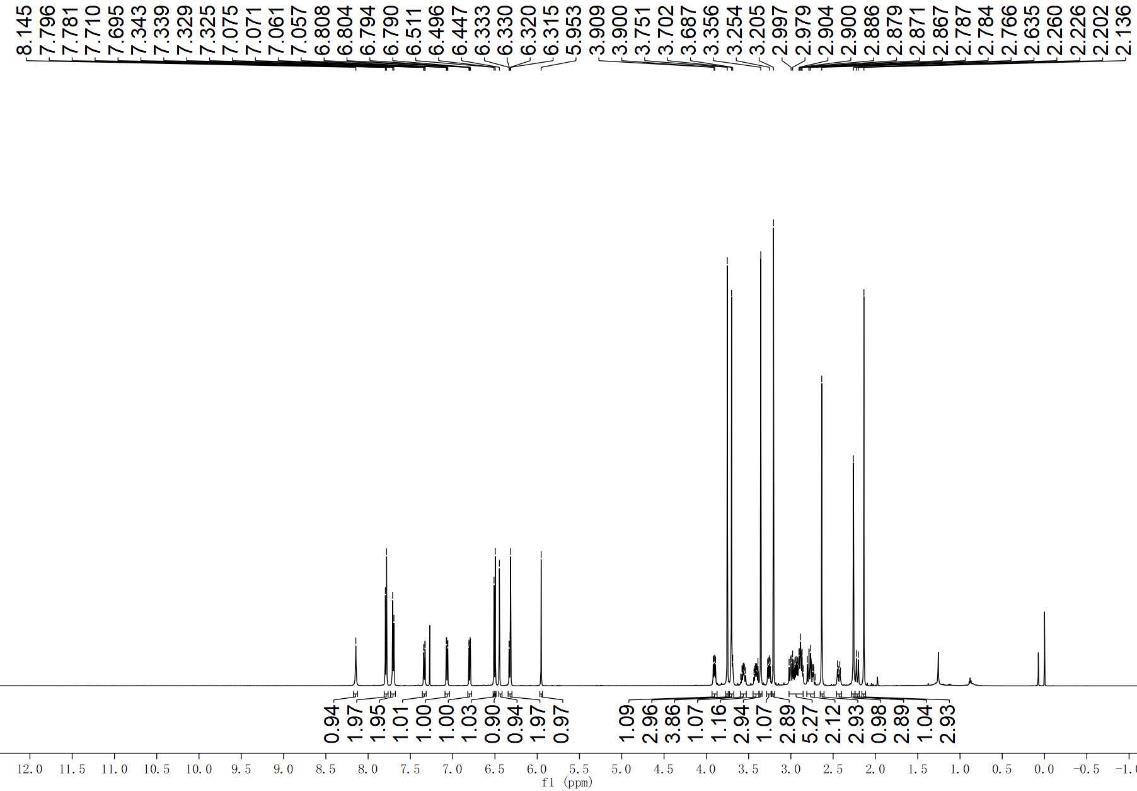


**Compound 22. 14-*O*-(4-acetamidobenzenesulfonyl)-tetrandrine: ^13^C-NMR spectrum**


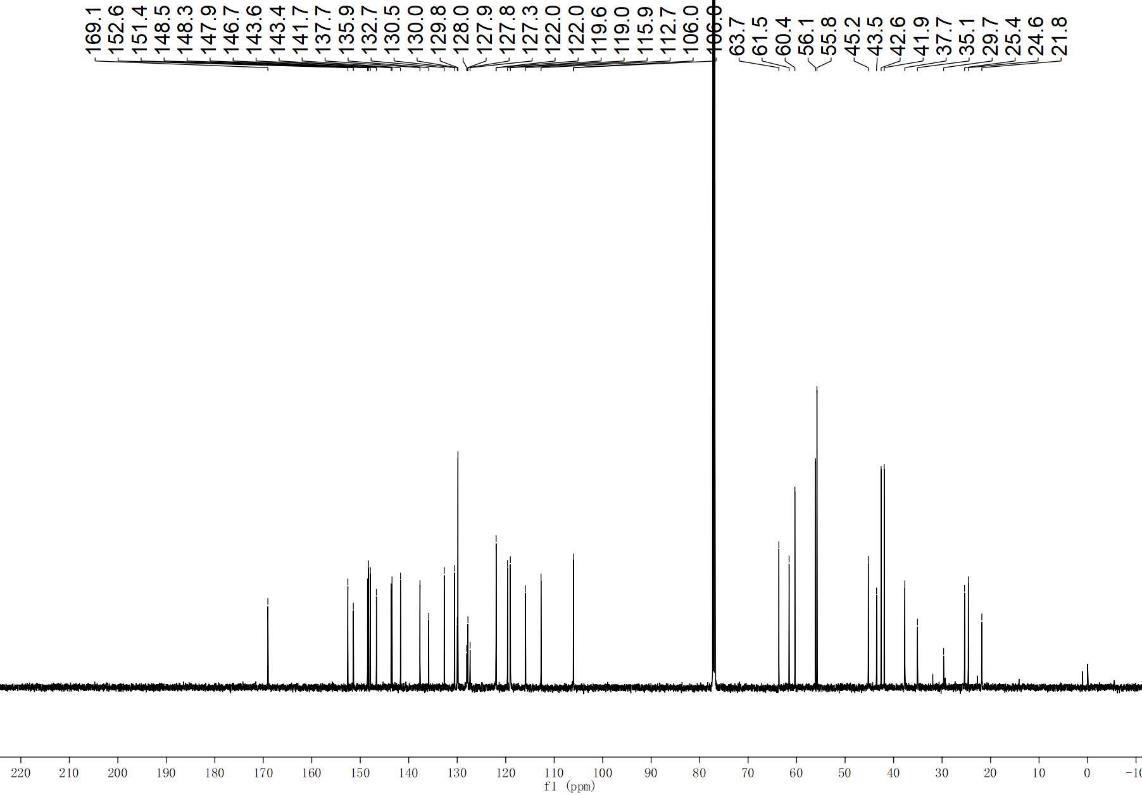


**Compound 22. 14-*O*-(4-acetamidobenzenesulfonyl)-tetrandrine: HR-ESIMS spectrum**

**Compound 23.** **14-*O*-(biphenyl-4-sulfonyl)-tetrandrine: ^1^H-NMR spectrum**


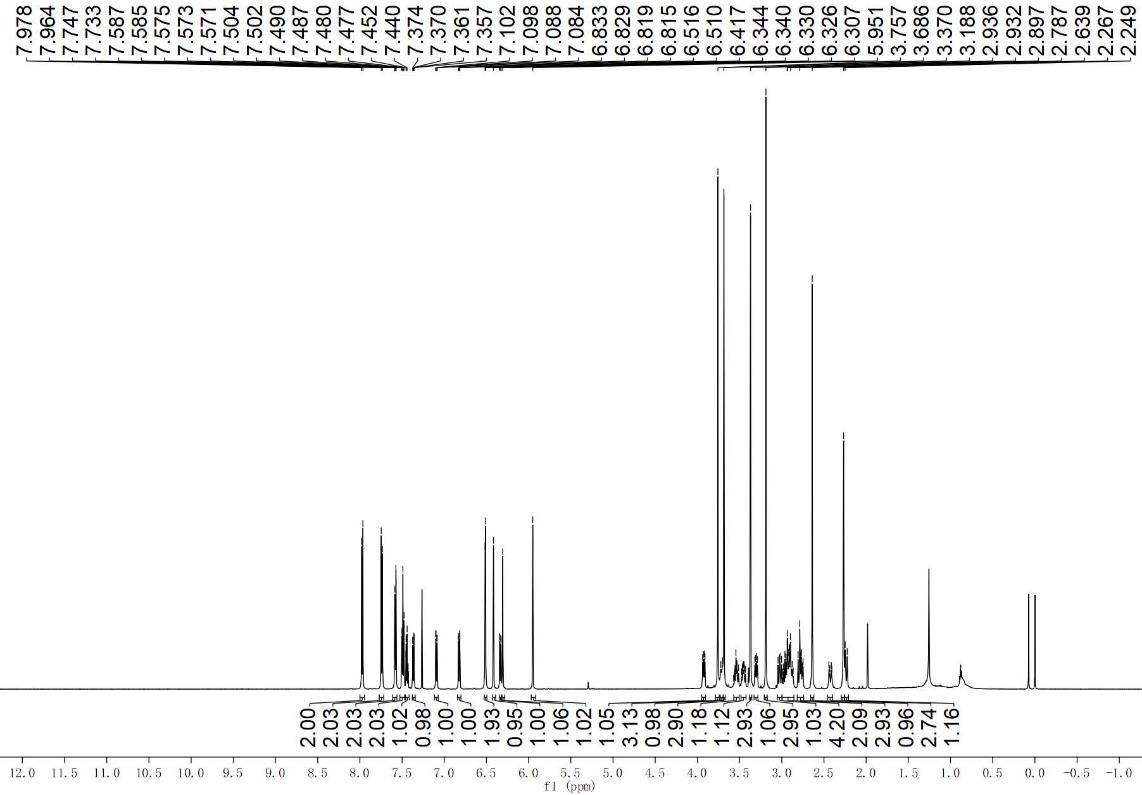


**Compound 23. 14-*O*-(biphenyl-4-sulfonyl)-tetrandrine: ^13^C-NMR spectrum**


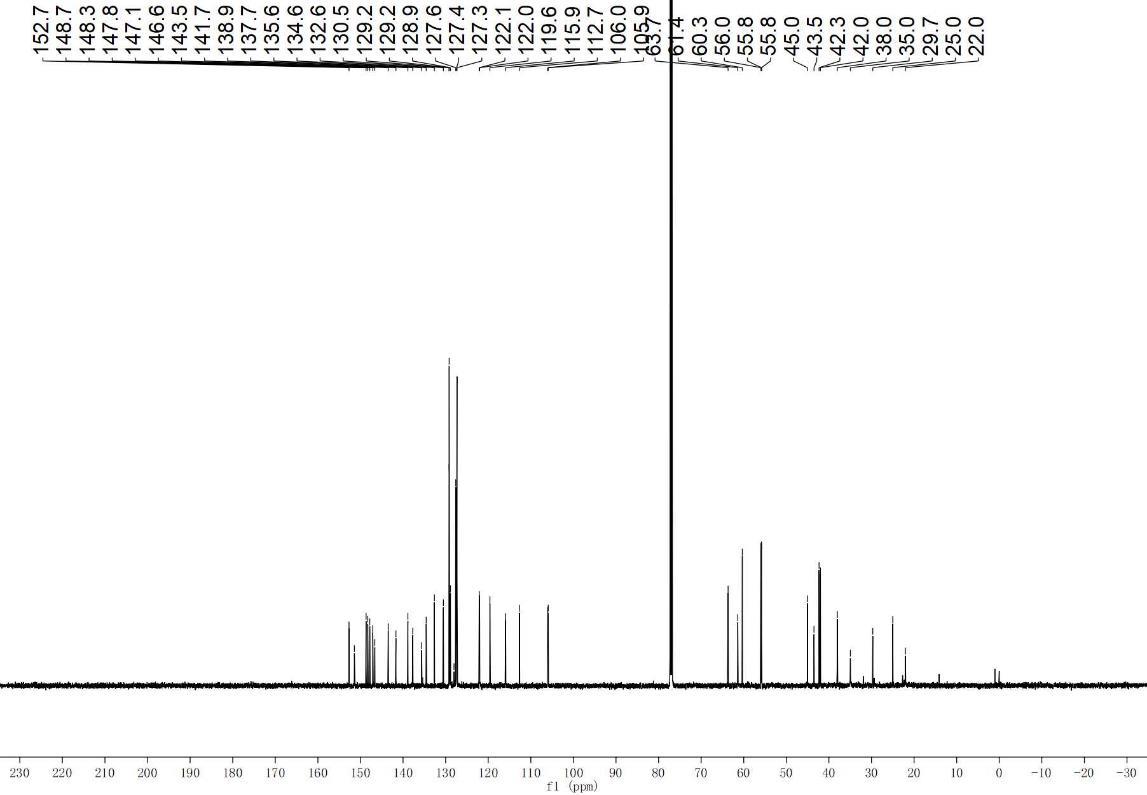


**Compound 23. 14-*O*-(biphenyl-4-sulfonyl)-tetrandrine: HR-ESIMS spectrum**

**Compound 24.** **14-*O*-(2-naphthalenesulfonyl)-tetrandrine: ^1^H-NMR spectrum**


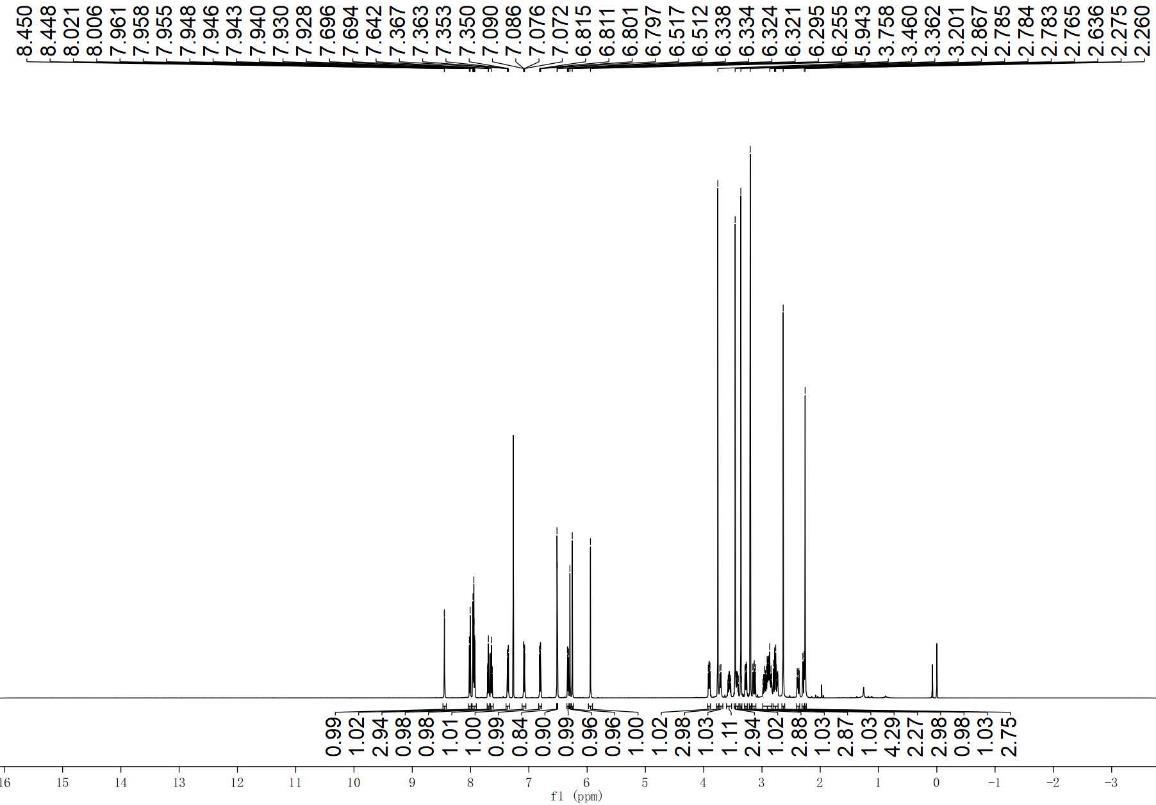


**Compound 24. 14-*O*-(2-naphthalenesulfonyl)-tetrandrine: ^13^C-NMR spectrum**


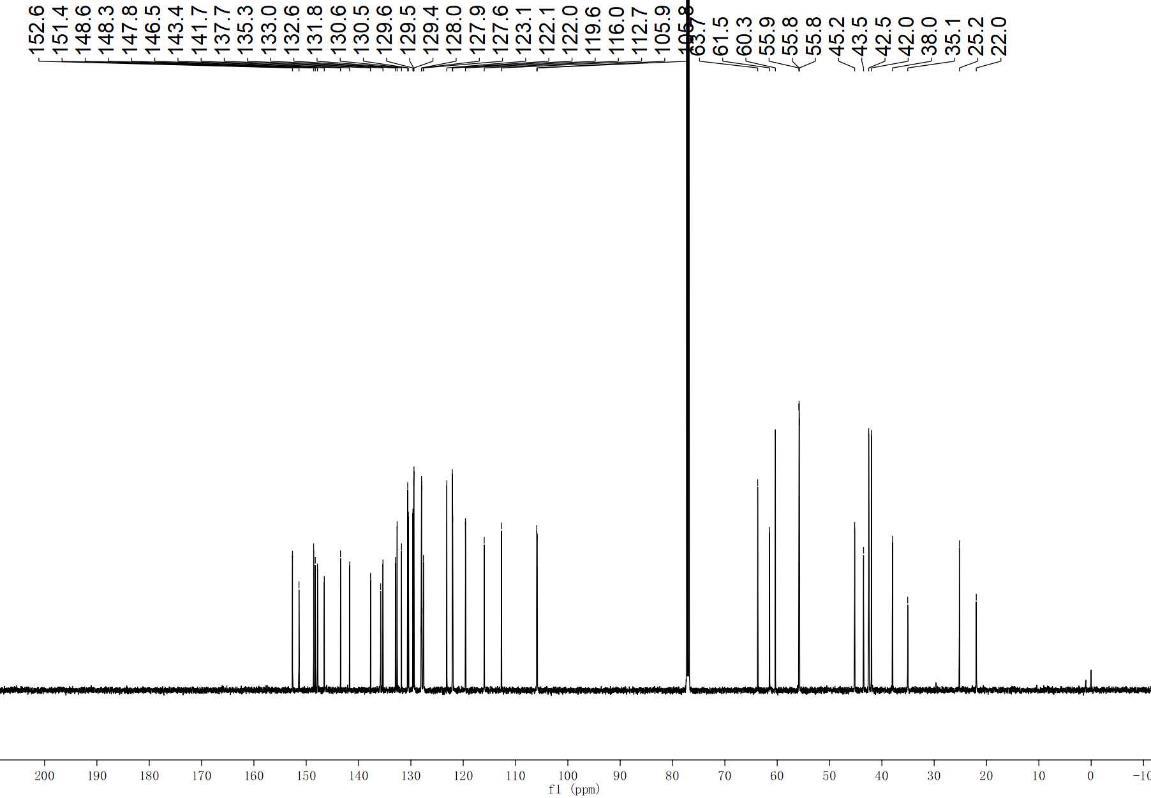


**Compound 24. 14-*O*-(2-naphthalenesulfonyl)-tetrandrine: HR-ESIMS spectrum**

**Compound 25.** **14-*O*-(8-quinolinesulfonyl)-tetrandrine: ^1^H-NMR spectrum**


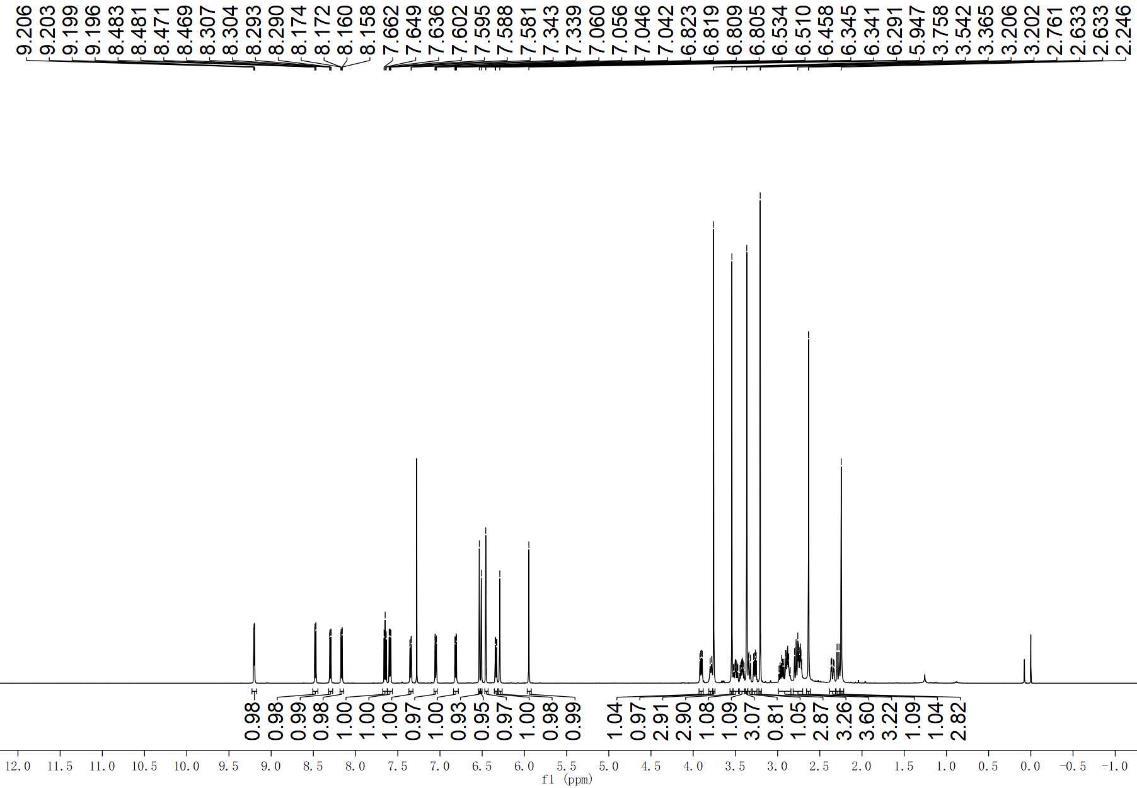


**Compound 25. 14-*O*-(8-quinolinesulfonyl)-tetrandrine: ^13^C-NMR spectrum**


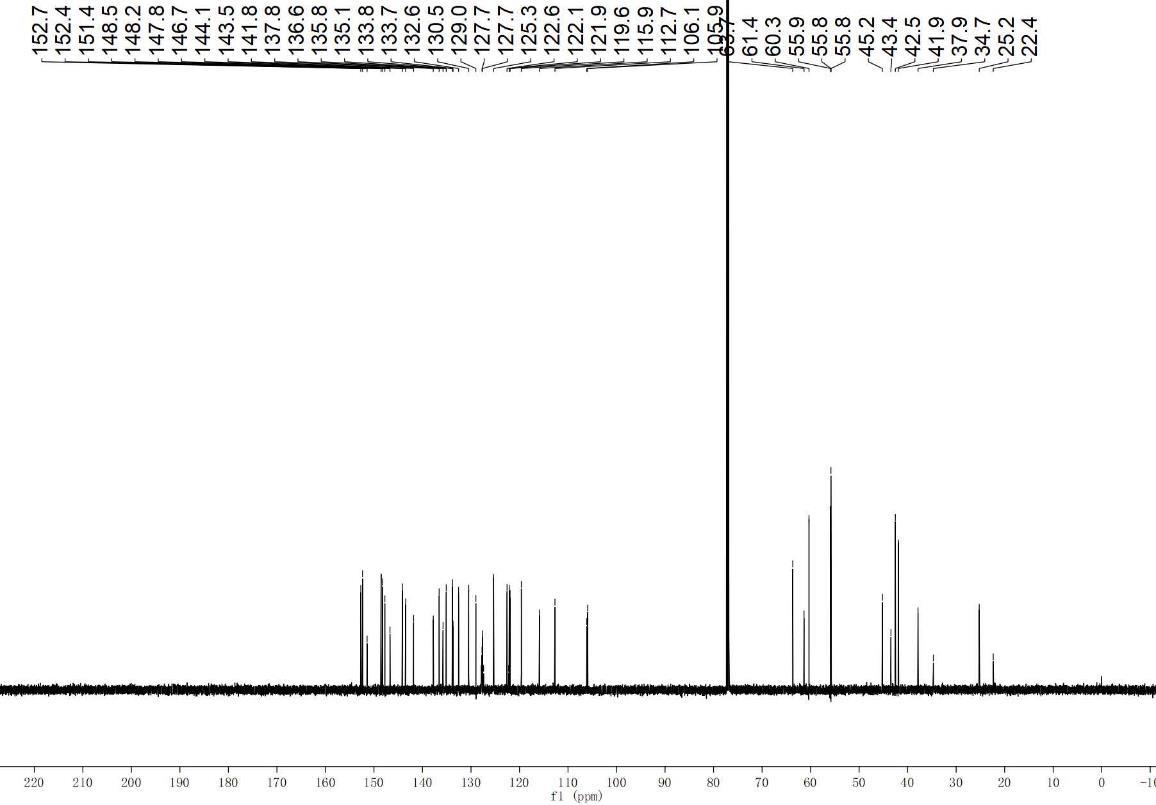


**Compound 25. 14-*O*-(8-quinolinesulfonyl)-tetrandrine: HR-ESIMS spectrum**

**Compound 26. 14-*O*-(3,5-dichlorobenzenesulfonyl)-tetrandrine: ^1^H-NMR spectrum**


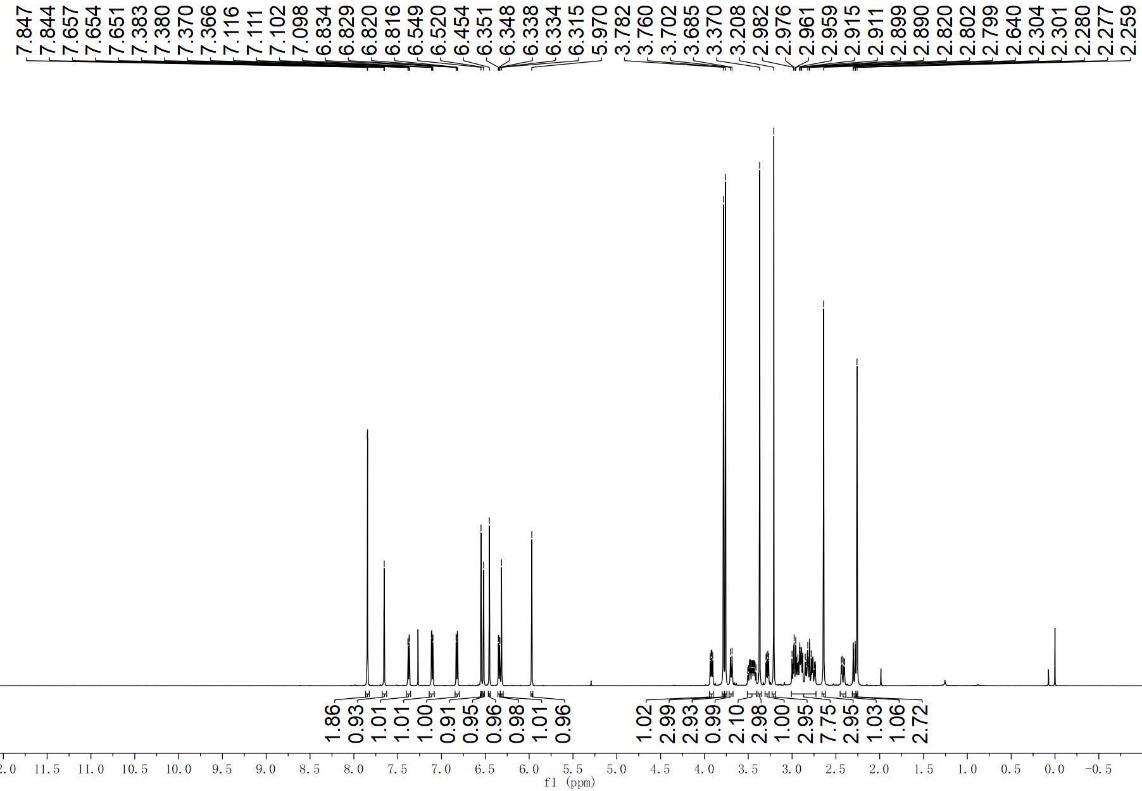


**Compound 26. 14-*O*-(3,5-dichlorobenzenesulfonyl)-tetrandrine: ^13^C-NMR spectrum**


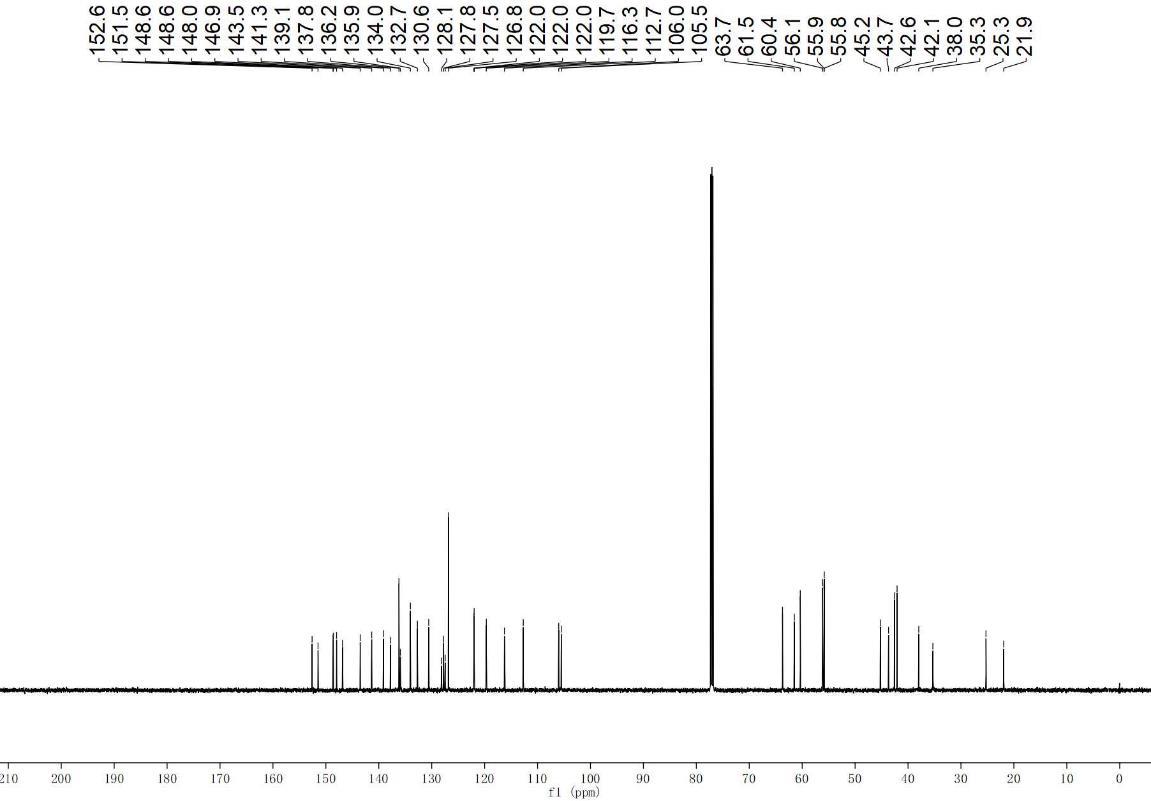


**Compound 26. 14-*O*-(3,5-dichlorobenzenesulfonyl)-tetrandrine: HR-ESIMS spectrum**

**Compound 27.** **14-*O*-(2,4-dichlorobenzenesulfonyl)-tetrandrine: ^1^H-NMR spectrum**


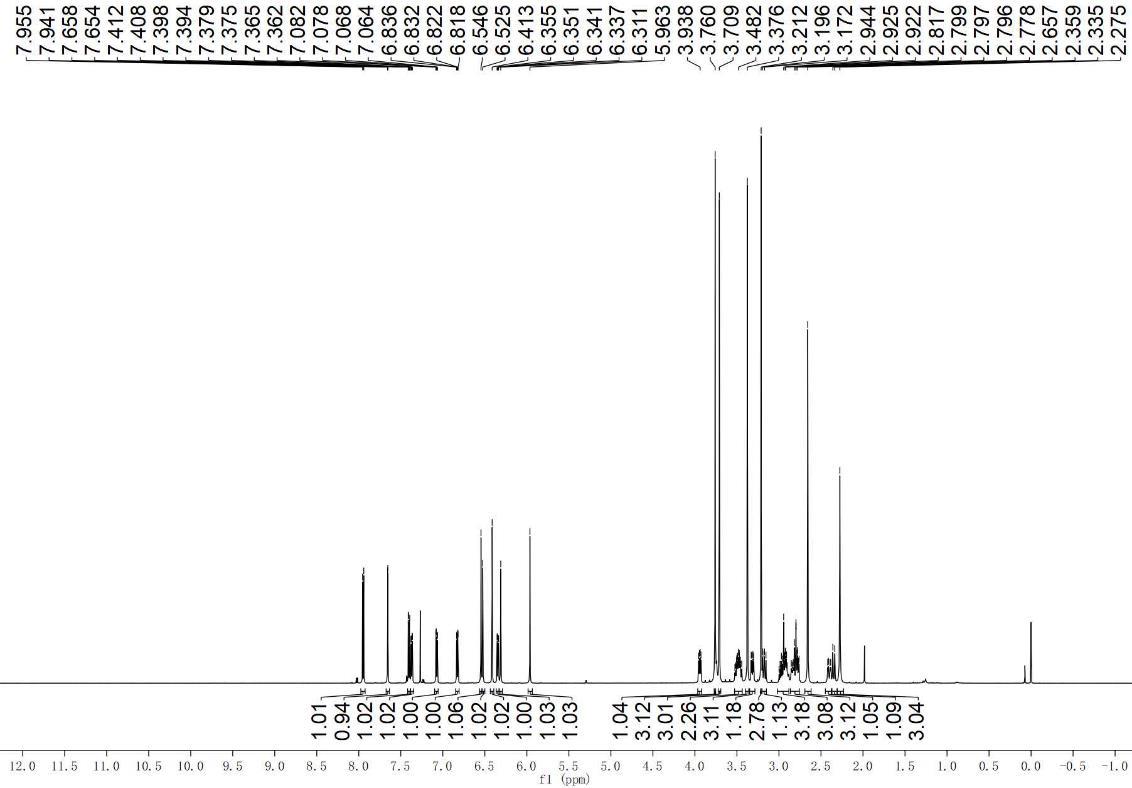


**Compound 27. 14-*O*-(2,4-dichlorobenzenesulfonyl)-tetrandrine: ^13^C-NMR spectrum**


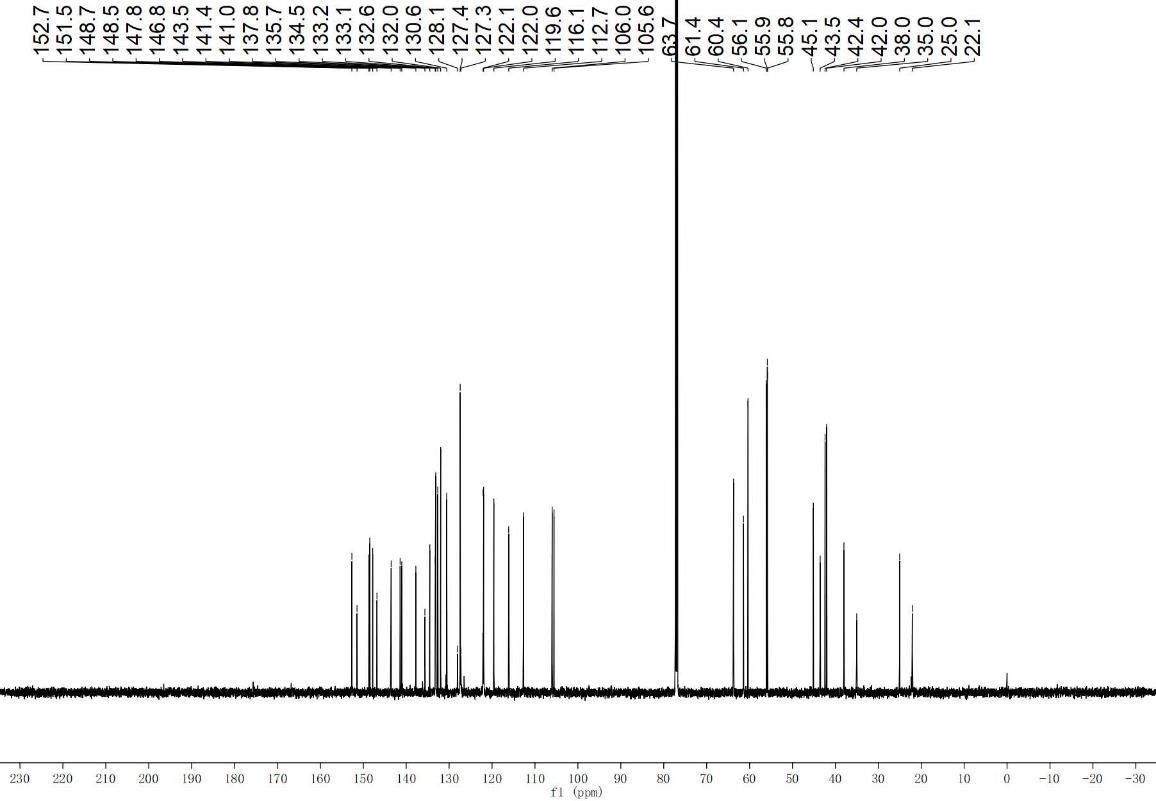


**Compound 27. 14-*O*-(2,4-dichlorobenzenesulfonyl)-tetrandrine: HR-ESIMS spectrum**

**Compound 28. 14-*O*-(2,4,6-trimethylbenzenesulfonyl)-tetrandrine: ^1^H-NMR spectrum**


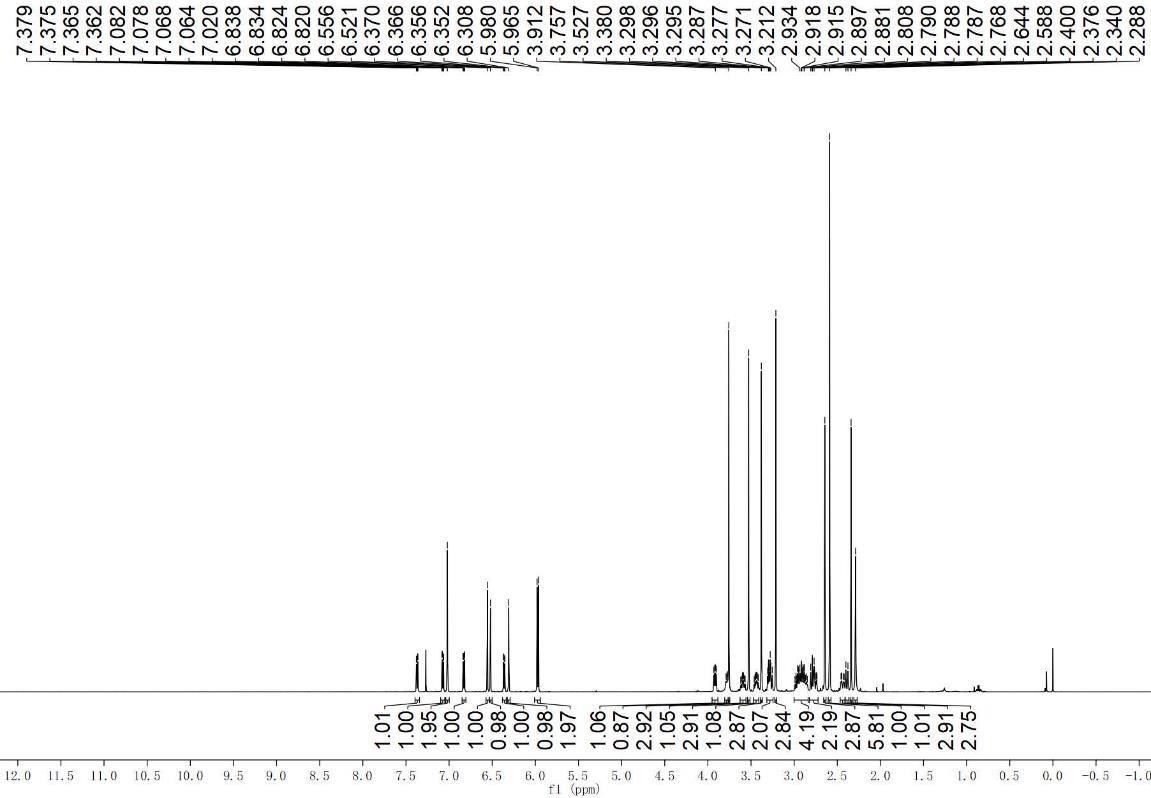


**Compound 28. 14-*O*-(2,4,6-trimethylbenzenesulfonyl)-tetrandrine: ^13^C-NMR spectrum**


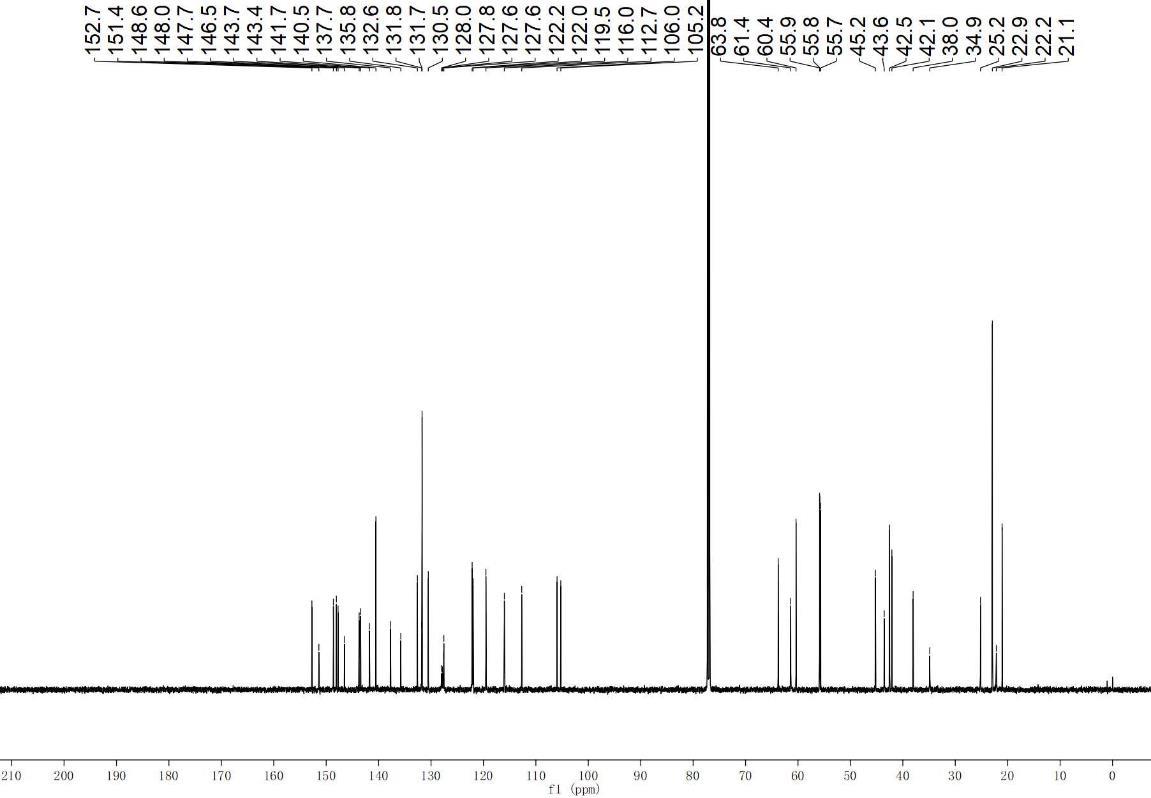


**Compound 28. 14-*O*-(2,4,6-trimethylbenzenesulfonyl)-tetrandrine: HR-ESIMS spectrum**

**Compound 29.** **14-*O*-(2,4,6-triisopropylbenzenesulfonyl )-tetrandrine: ^1^H-NMR spectrum**


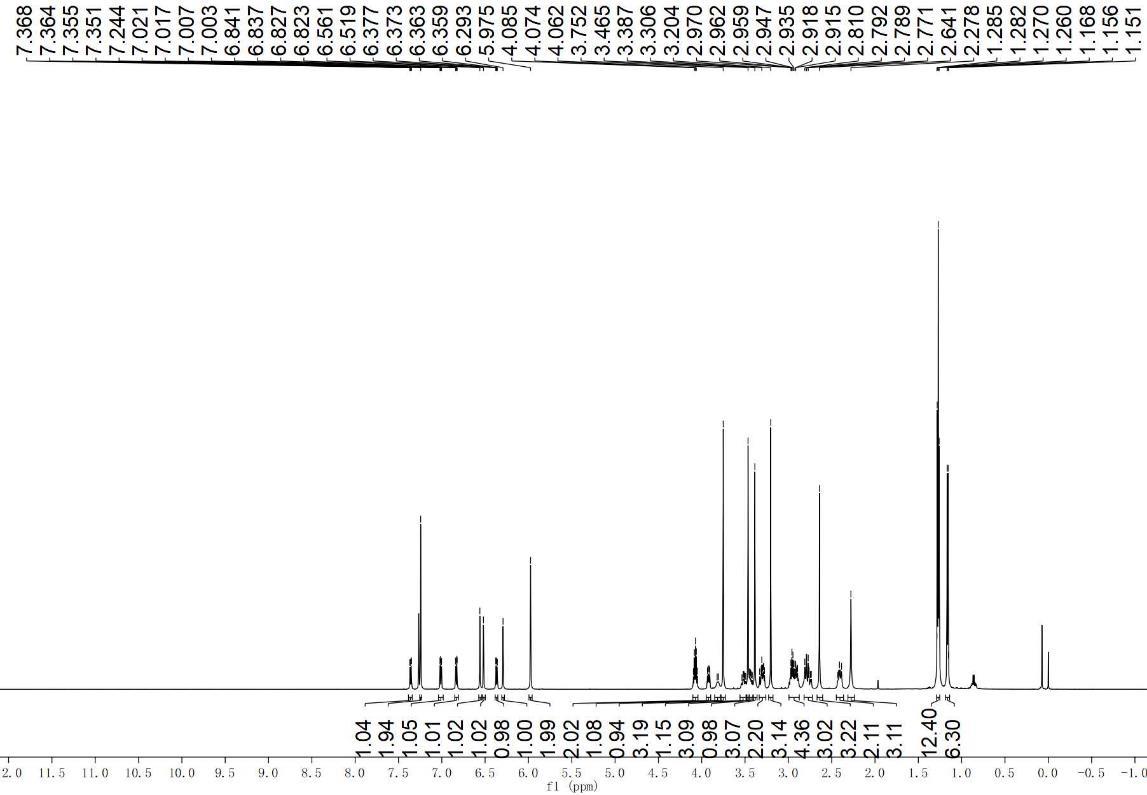


**Compound 29. 14-*O*-(2,4,6-triisopropylbenzenesulfonyl )-tetrandrine: ^13^C-NMR spectrum**


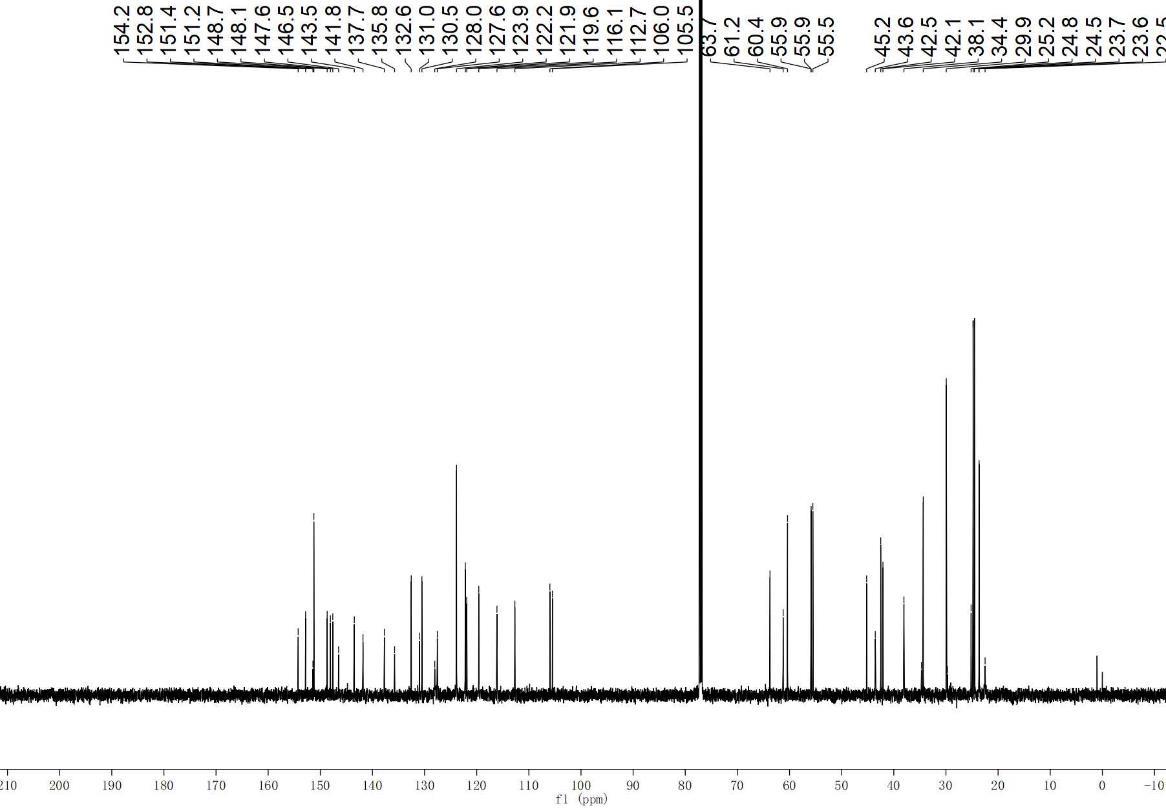


**Compound 29. 14-*O*-(2,4,6-triisopropylbenzenesulfonyl )-tetrandrine: HR-ESIMS spectrum**

**Compound 30.** **14-*O*-(pyridine-3-sulfonyl)-tetrandrine: ^1^H-NMR spectrum**


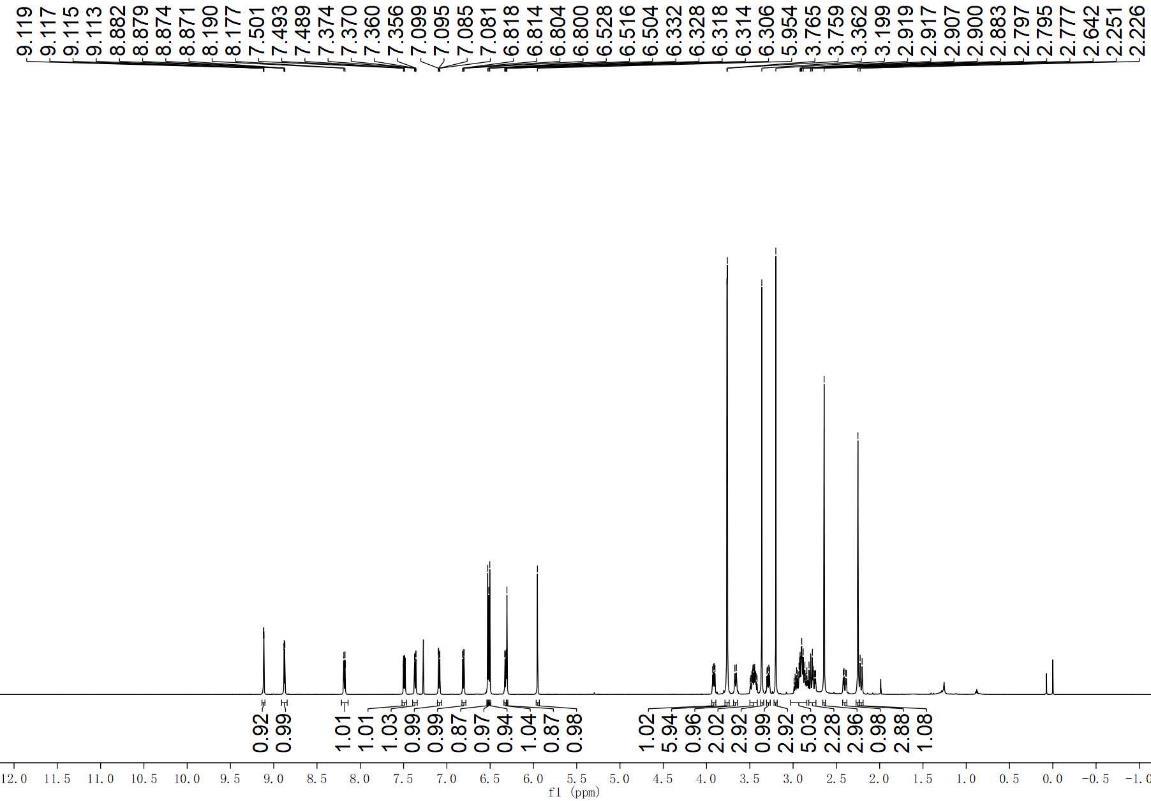


**Compound 30. 14-*O*-(pyridine-3-sulfonyl)-tetrandrine: ^13^C-NMR spectrum**


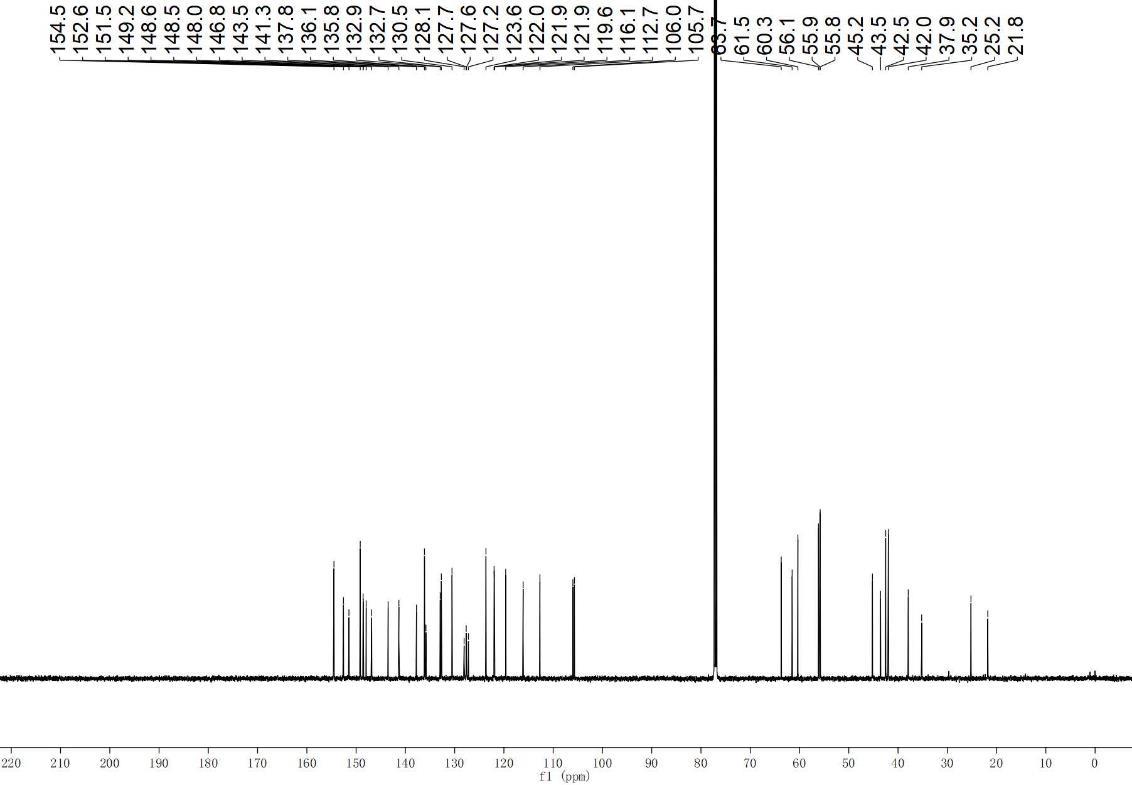


**Compound 30. 14-*O*-(pyridine-3-sulfonyl)-tetrandrine: HR-ESIMS spectrum**

**Compound 31.** **14-*O*-(2-chloropyridine-5-sulfonyl)-tetrandrine: ^1^H-NMR spectrum**


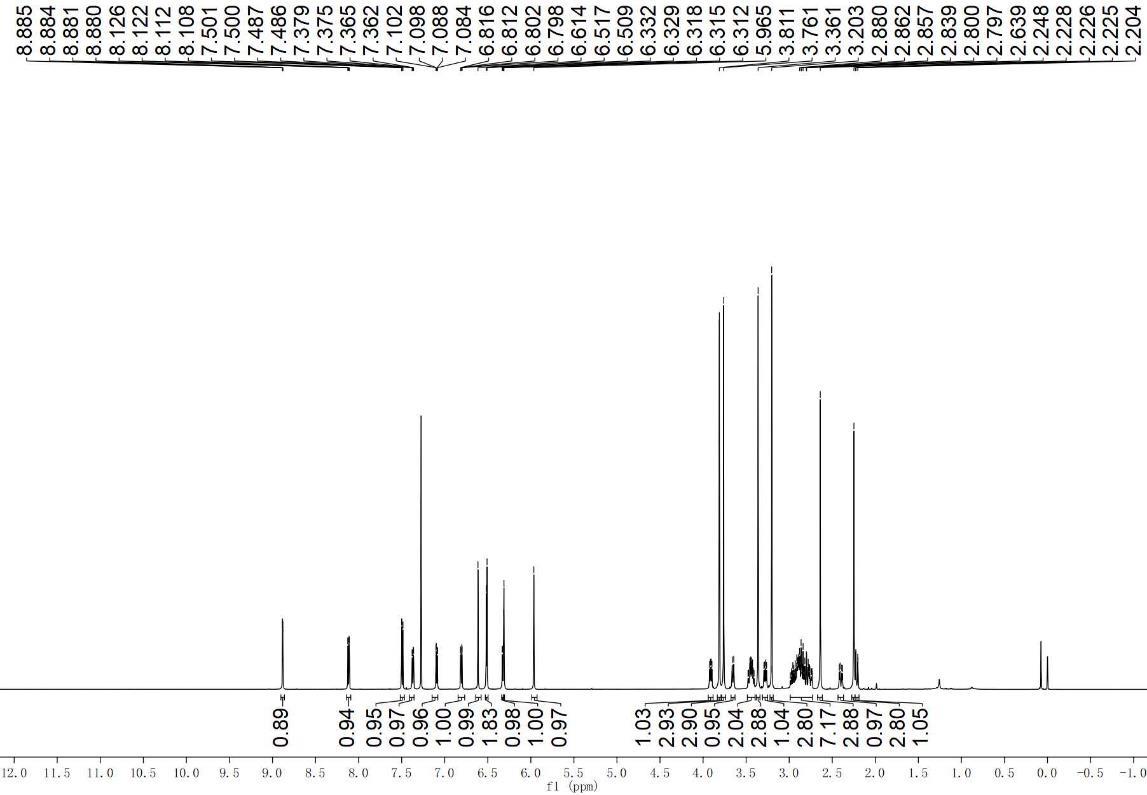


**Compound 31. 14-*O*-(2-chloropyridine-5-sulfonyl)-tetrandrine: ^13^C-NMR spectrum**


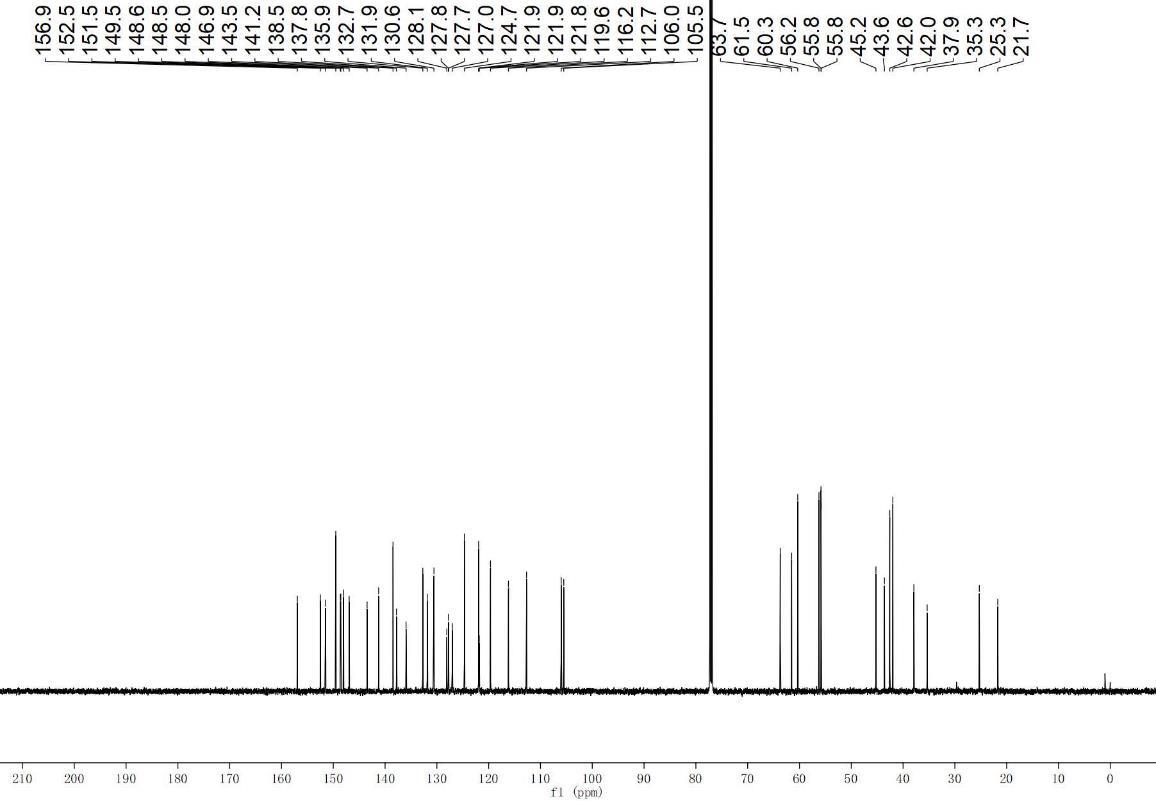


**Compound 31. 14-*O*-(2-chloropyridine-5-sulfonyl)-tetrandrine**

**Compound 32.** **14-*O*-(2-thiophenesulfonyl)-tetrandrine: ^1^H-NMR spectrum**


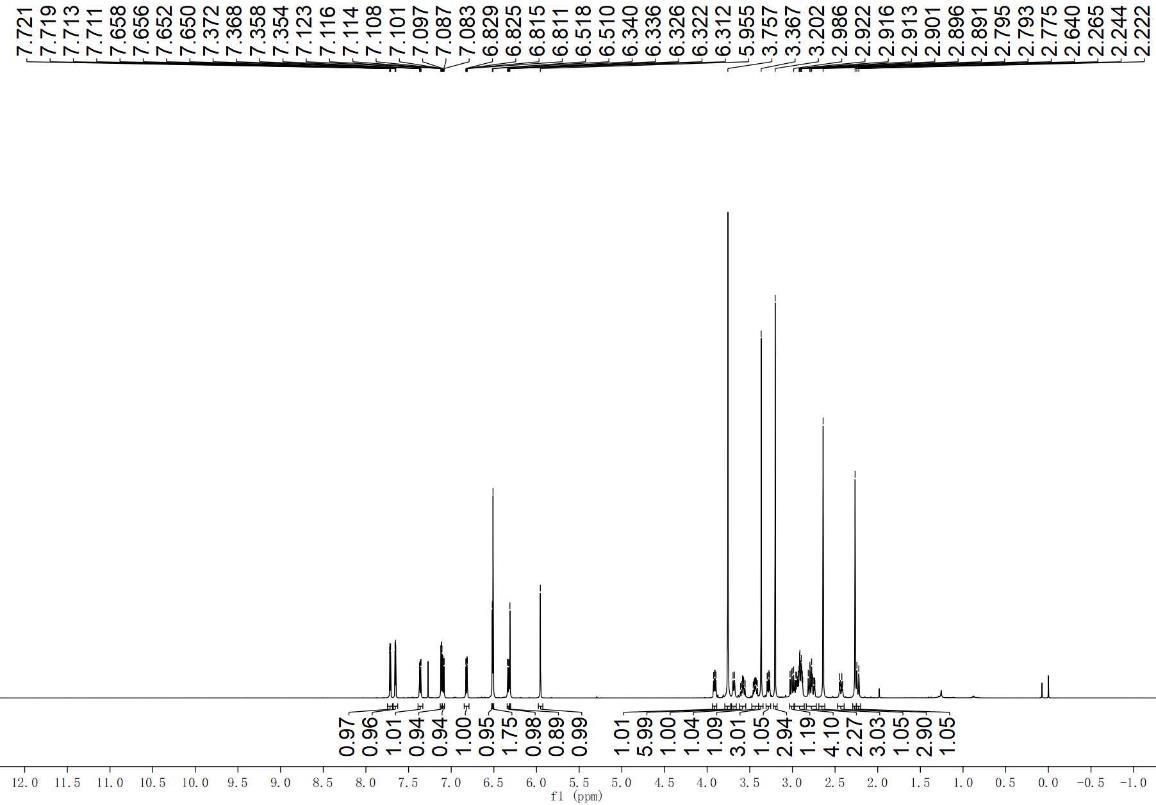


**Compound 32. 14-*O*-(2-thiophenesulfonyl)-tetrandrine: ^13^C-NMR spectrum**


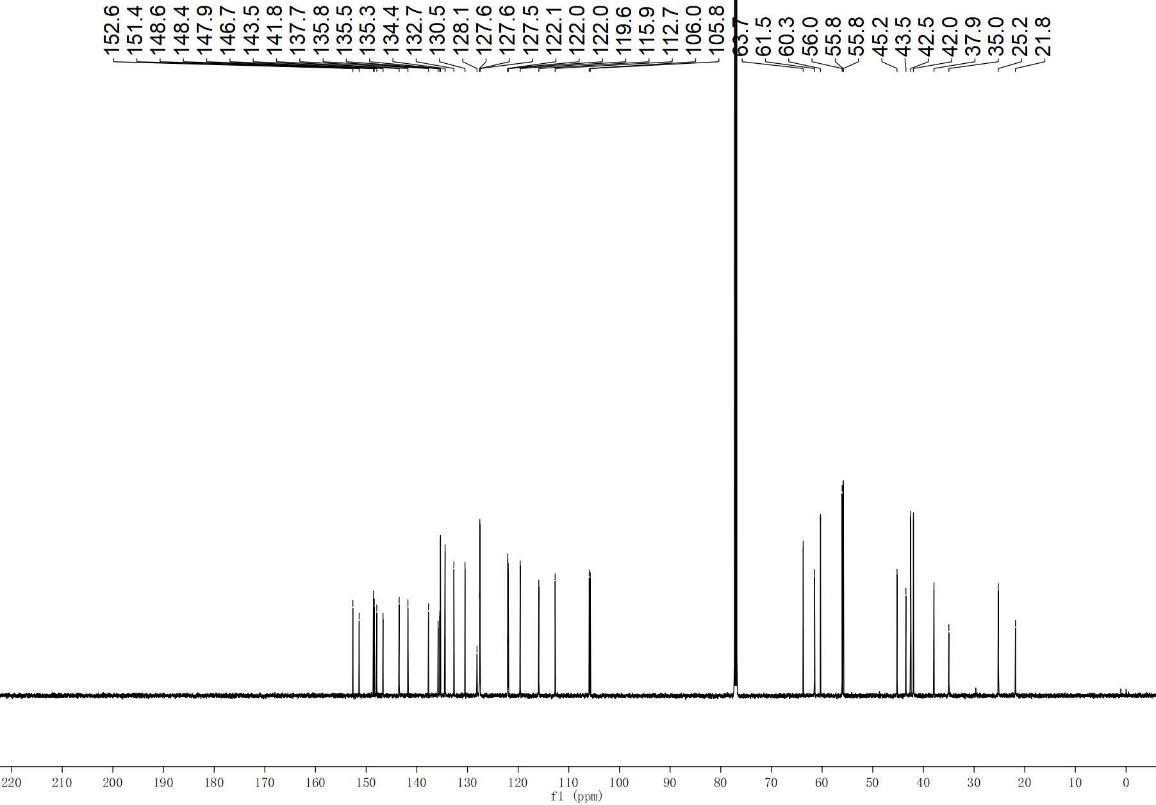


**Compound 32. 14-*O*-(2-thiophenesulfonyl)-tetrandrine: HR-ESIMS spectrum**

**Compound 33.** **14-*O*-(5-chlorothiophene-2-sulfonyl)-tetrandrine: ^1^H-NMR spectrum**


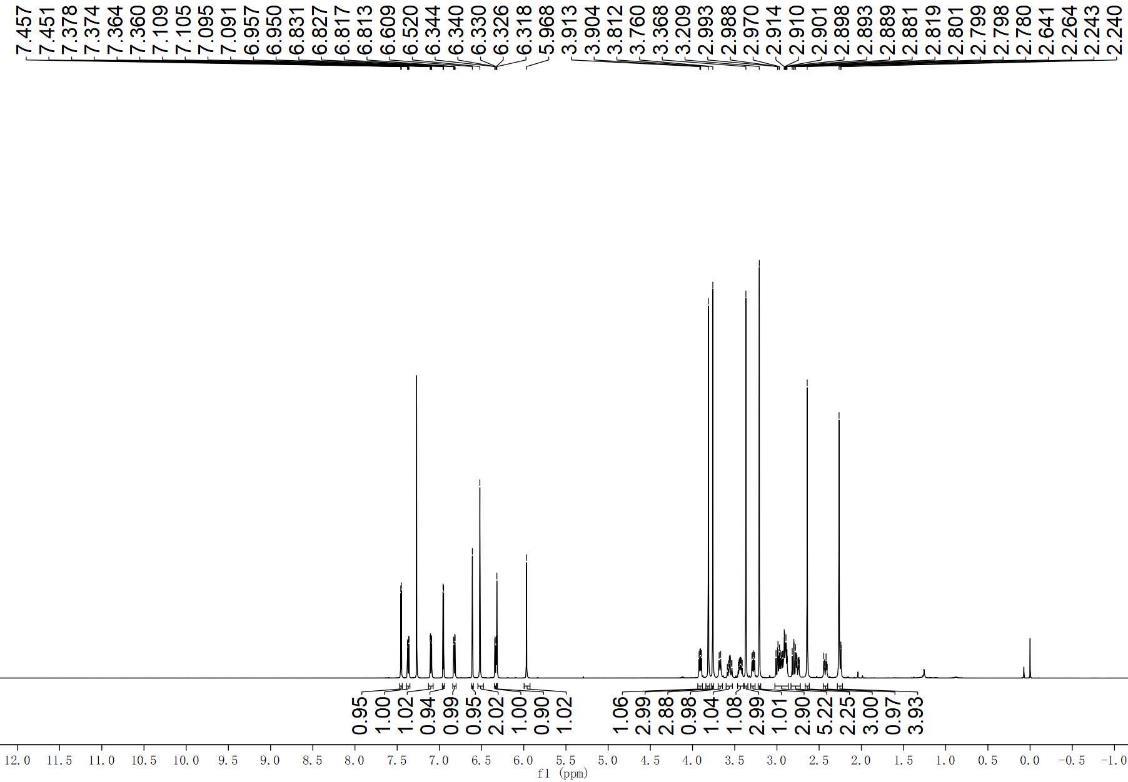


**Compound 33. 14-*O*-(5-chlorothiophene-2-sulfonyl)-tetrandrine: ^13^C-NMR spectrum**


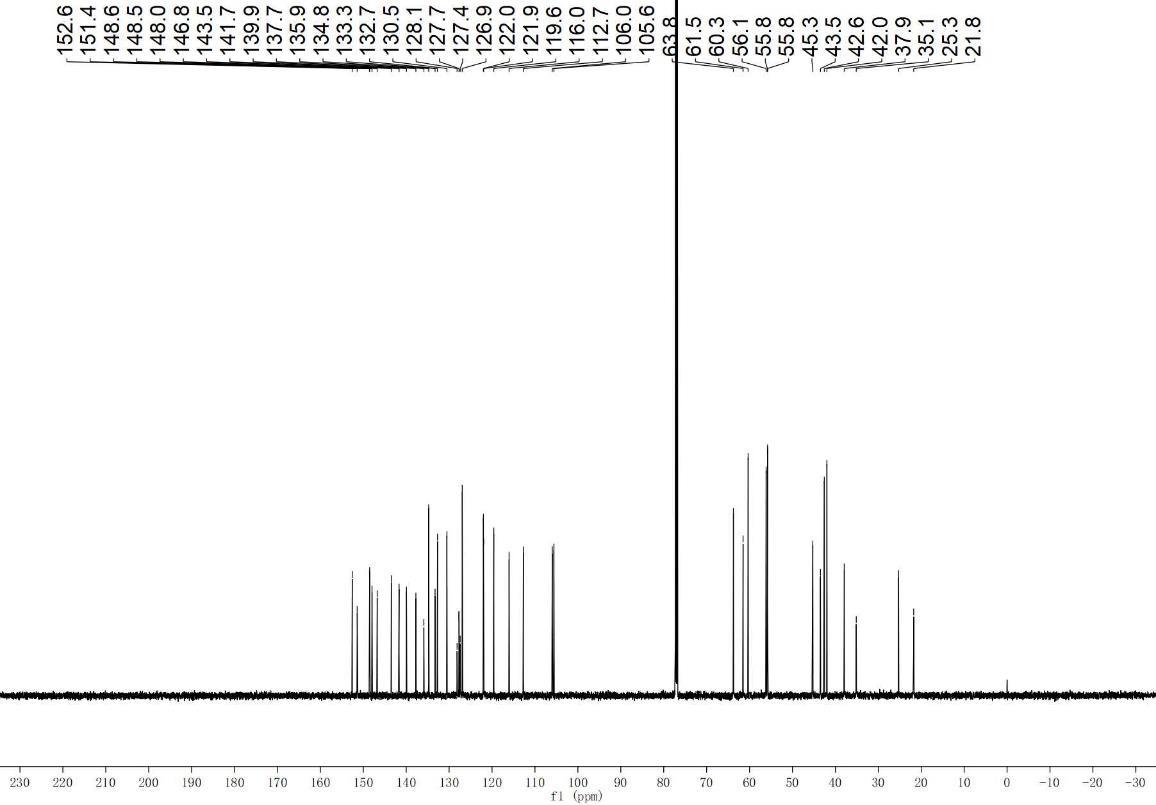


**Compound 33. 14-*O*-(5-chlorothiophene-2-sulfonyl)-tetrandrine**

**Compound 34. 14-*O*-(2,4,6- trifluoromethanesulfonyl)-tetrandrine: ^1^H-NMR spectrum**


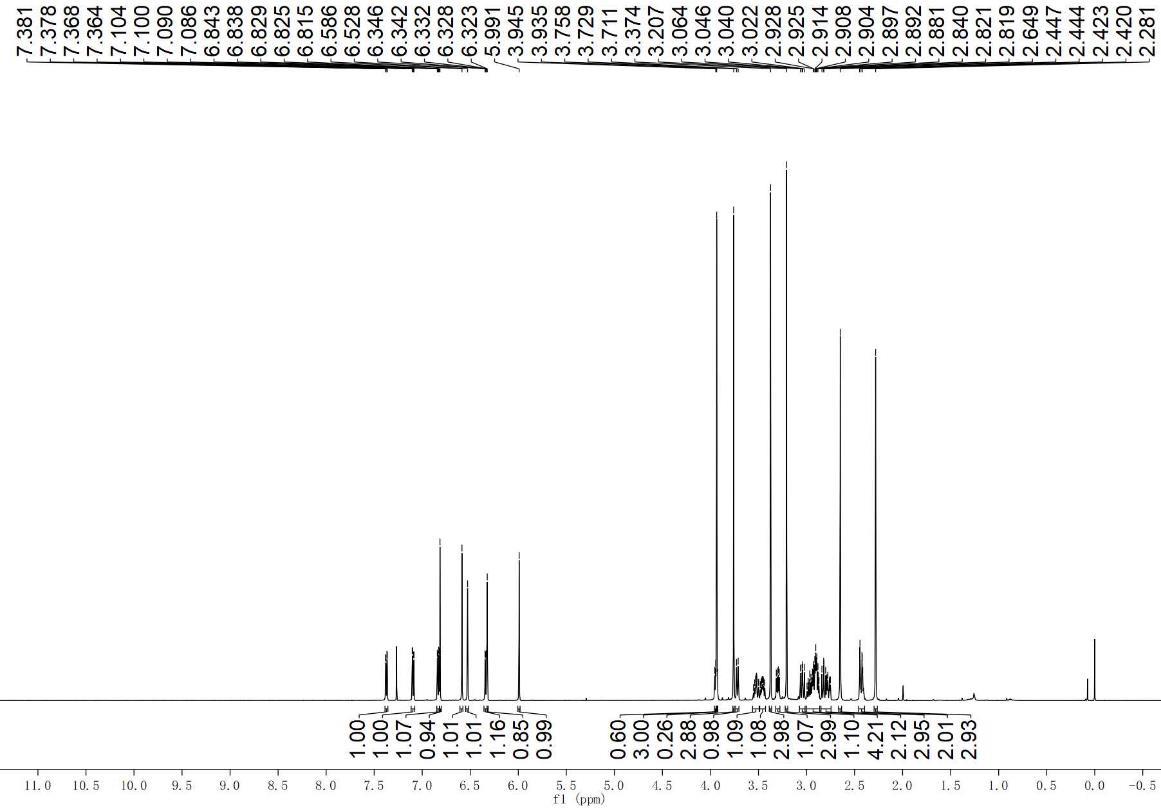


**Compound 34. 14-*O*-(2,4,6- trifluoromethanesulfonyl)-tetrandrine: ^13^C-NMR spectrum**


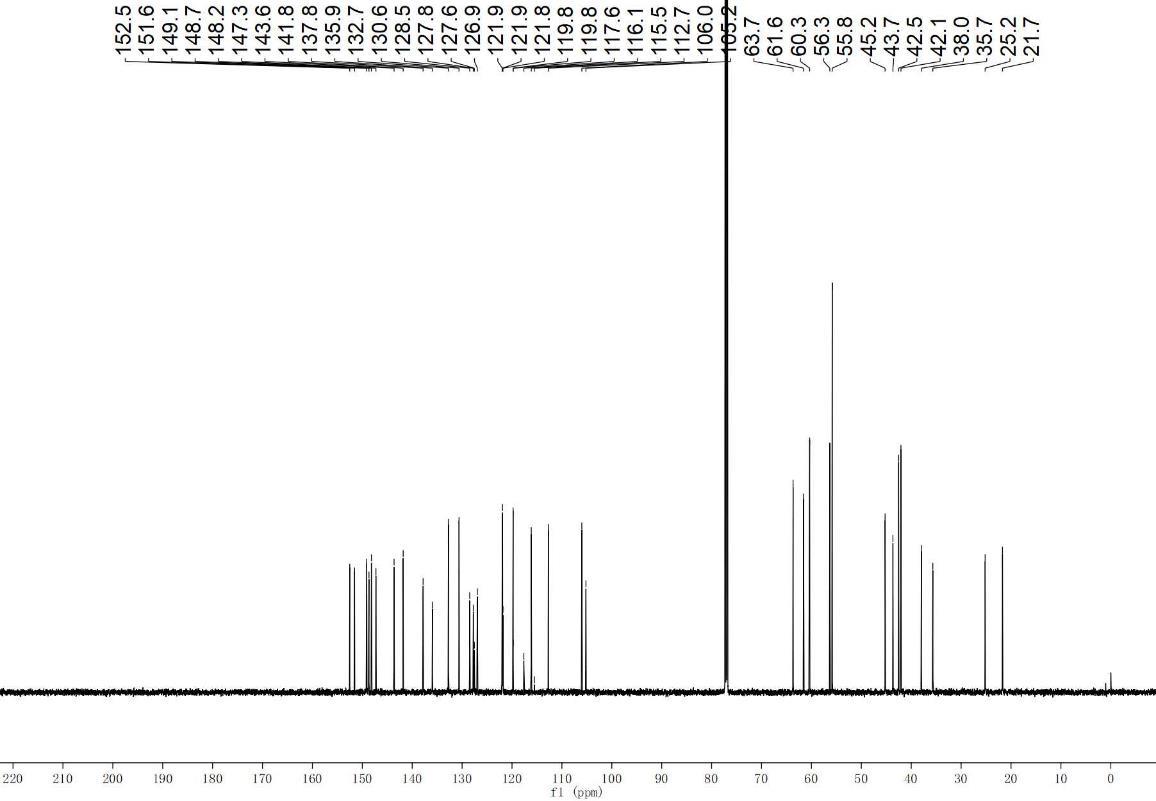


**Compound 34. 14-*O*-(2,4,6- trifluoromethanesulfonyl)-tetrandrine: ^19^F-NMR spectrum**


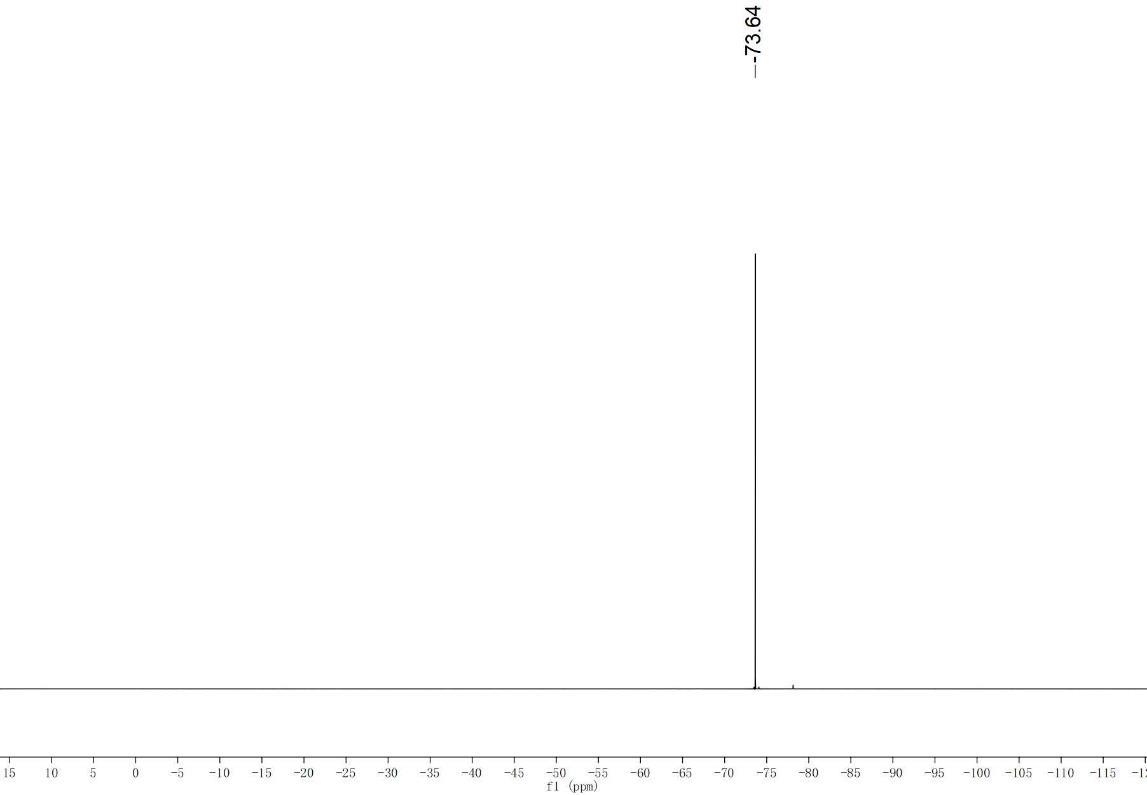


**Compound 34. 14-*O*-(2,4,6- trifluoromethanesulfonyl)-tetrandrine: HR-ESIMS spectrum**

**Compound 35.** **14-*O*-(dimethylsulfamoyl)-tetrandrine: ^1^H-NMR spectrum**


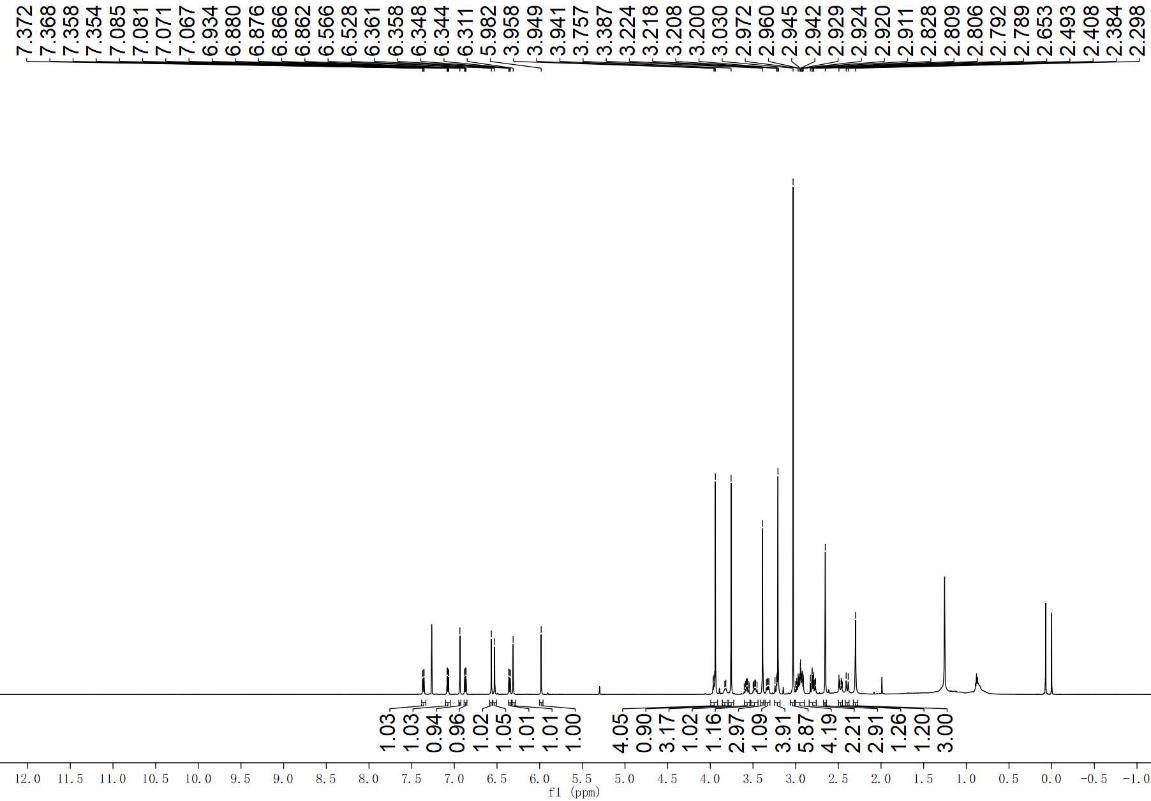


**Compound 35. 14-*O*-(dimethylsulfamoyl)-tetrandrine: ^13^C-NMR spectrum**


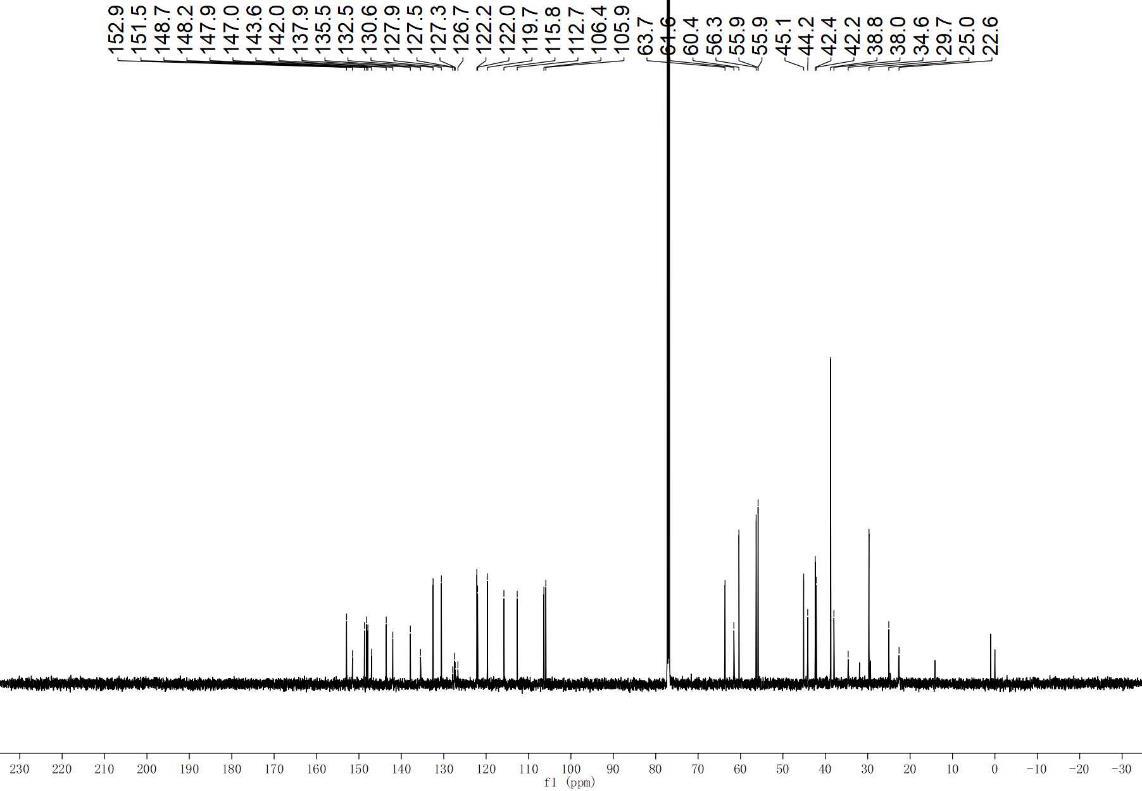


**Compound 35. 14-*O*-(dimethylsulfamoyl)-tetrandrine: HR-ESIMS spectrum**

**Compound 36.** **14-*O*-(ethanesulfonyl)-tetrandrine: ^1^H-NMR spectrum**


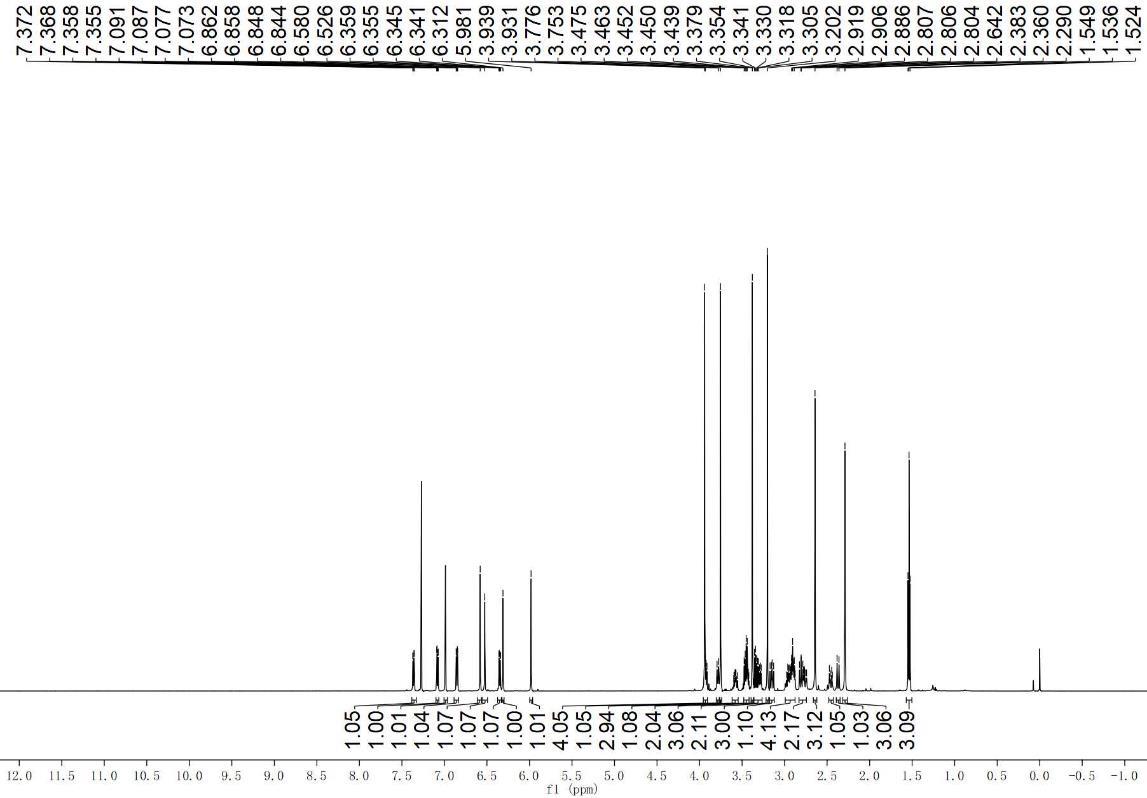


**Compound 36. 14-*O*-(ethanesulfonyl)-tetrandrine: ^13^C-NMR spectrum**


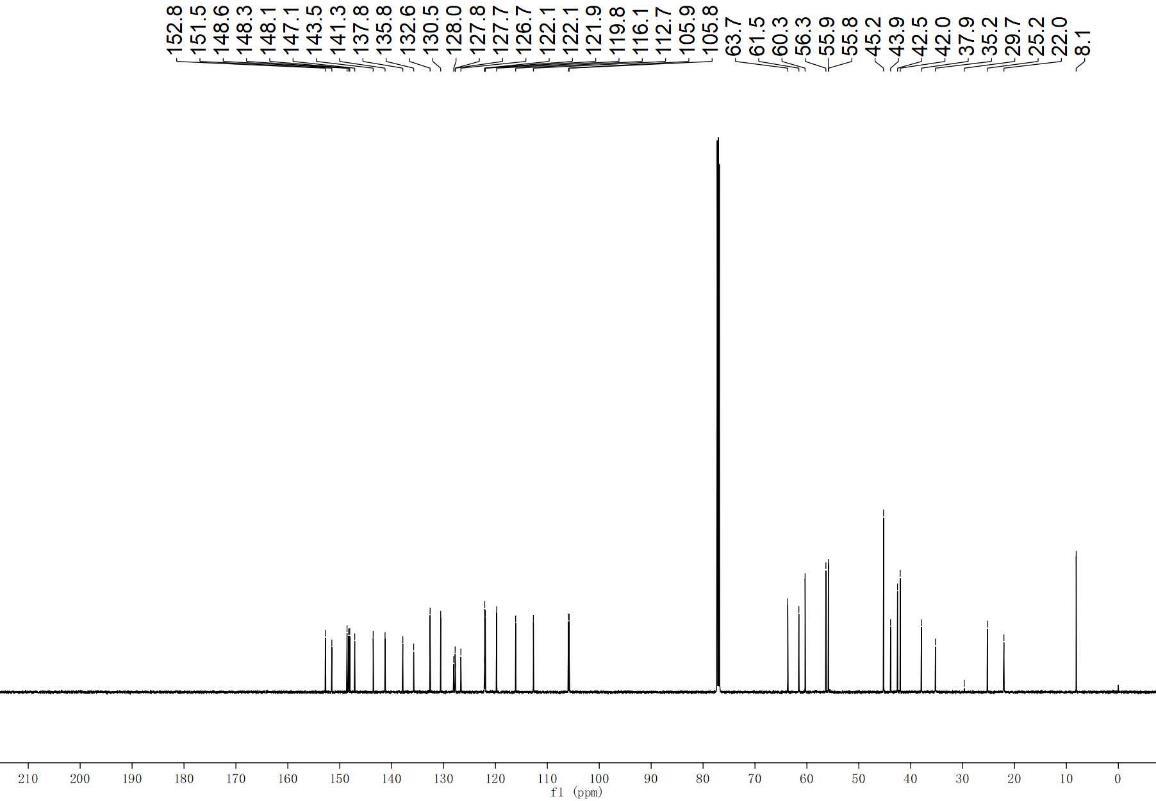


**Compound 36. 14-*O*-(ethanesulfonyl)-tetrandrine: HR-ESIMS spectrum**

**Compound 37.** **14-*O*-(1-propanesulfonyl)-tetrandrine: ^1^H-NMR spectrum**


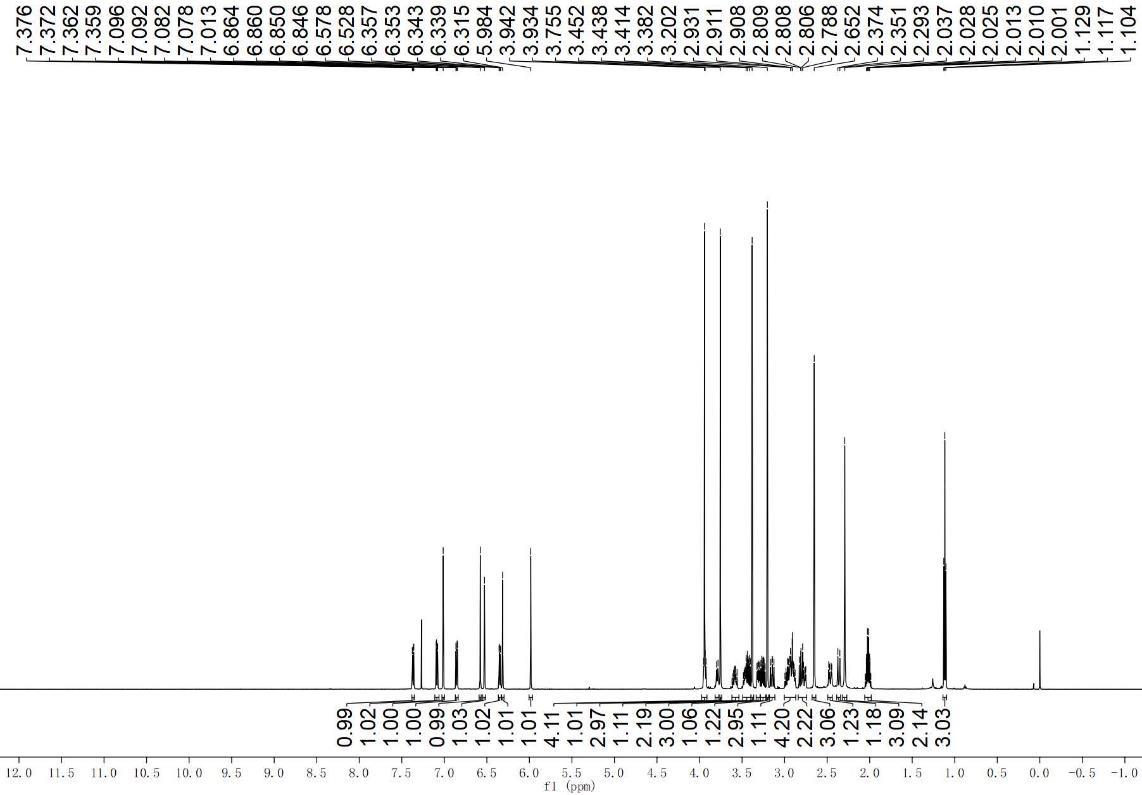


**Compound 37. 14-*O*-(1-propanesulfonyl)-tetrandrine: ^13^C-NMR spectrum**


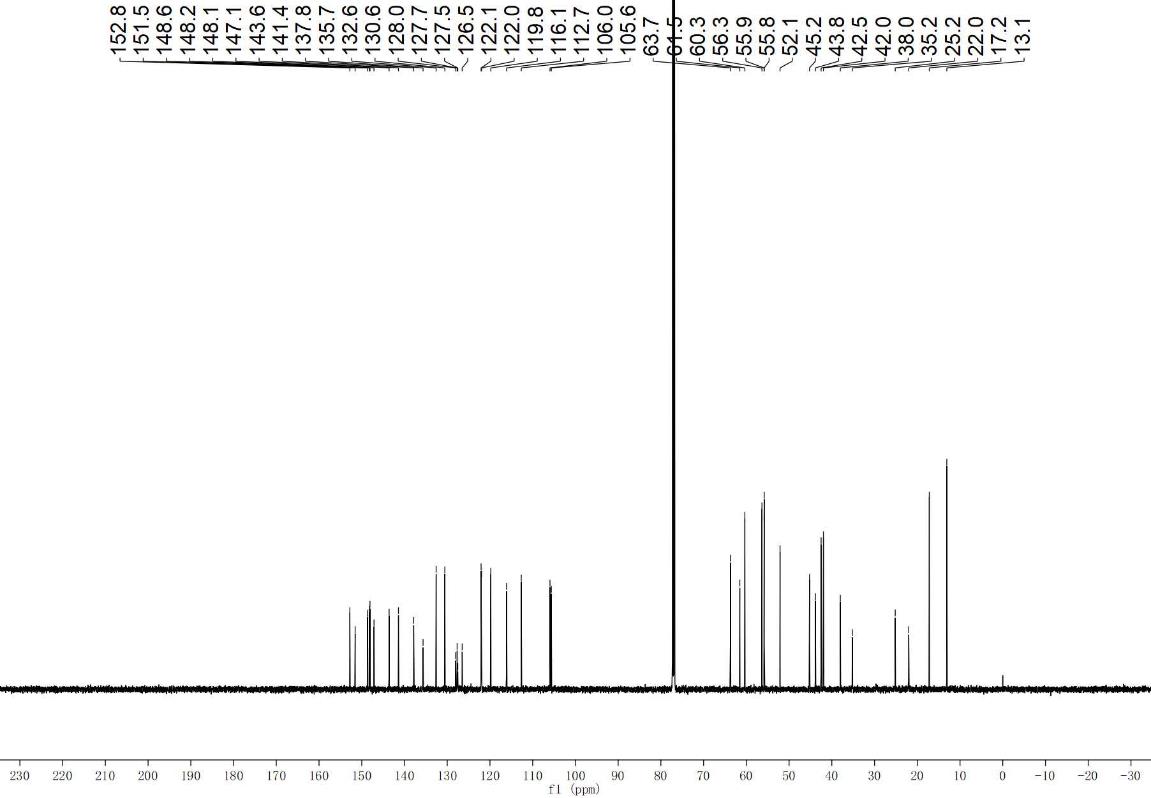


**Compound 37. 14-*O*-(1-propanesulfonyl)-tetrandrine: HR-ESIMS spectrum**

**Compound 38.** **14-*O*-(1-butanesulfonyl)-tetrandrine: ^1^H-NMR spectrum**


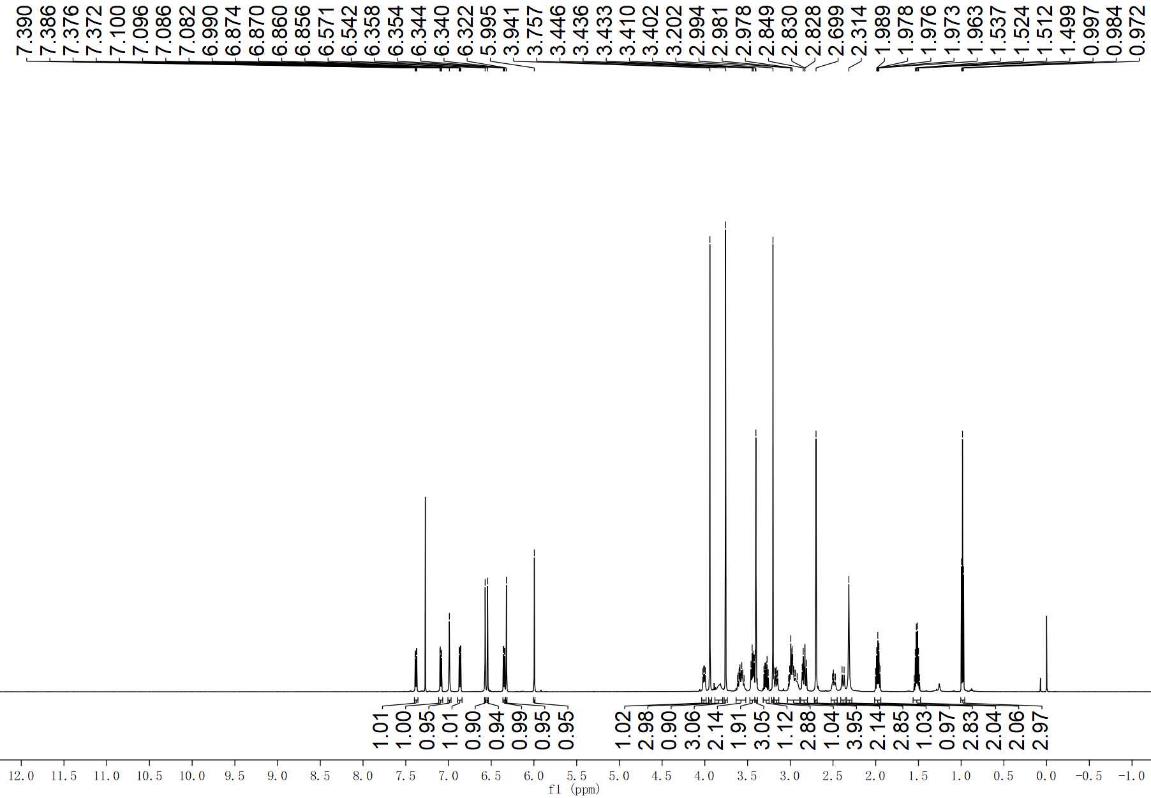


**Compound 38. 14-*O*-(1-butanesulfonyl)-tetrandrine: ^13^C-NMR spectrum**


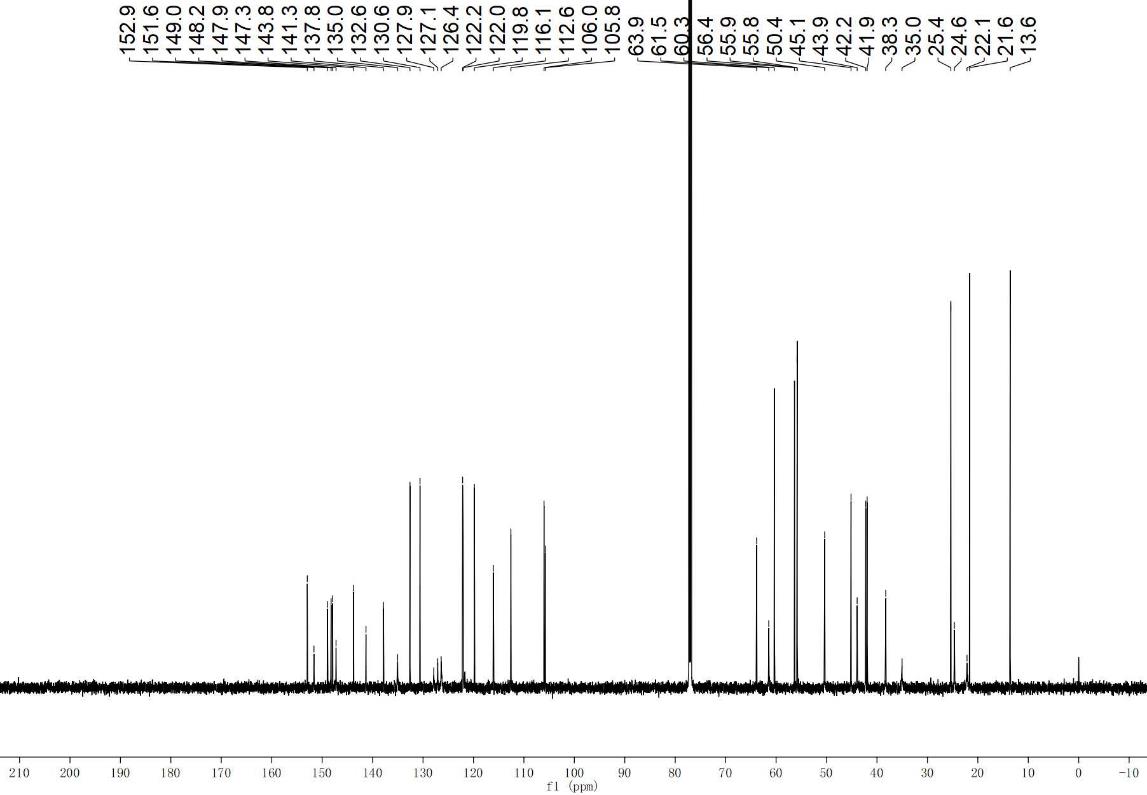


**Compound 38. 14-*O*-(1-butanesulfonyl)-tetrandrine: HR-ESIMS spectrum**

**Compound 39.** **14-*O*-(cyclopropanesulfonyl)-tetrandrine: ^1^H-NMR spectrum**


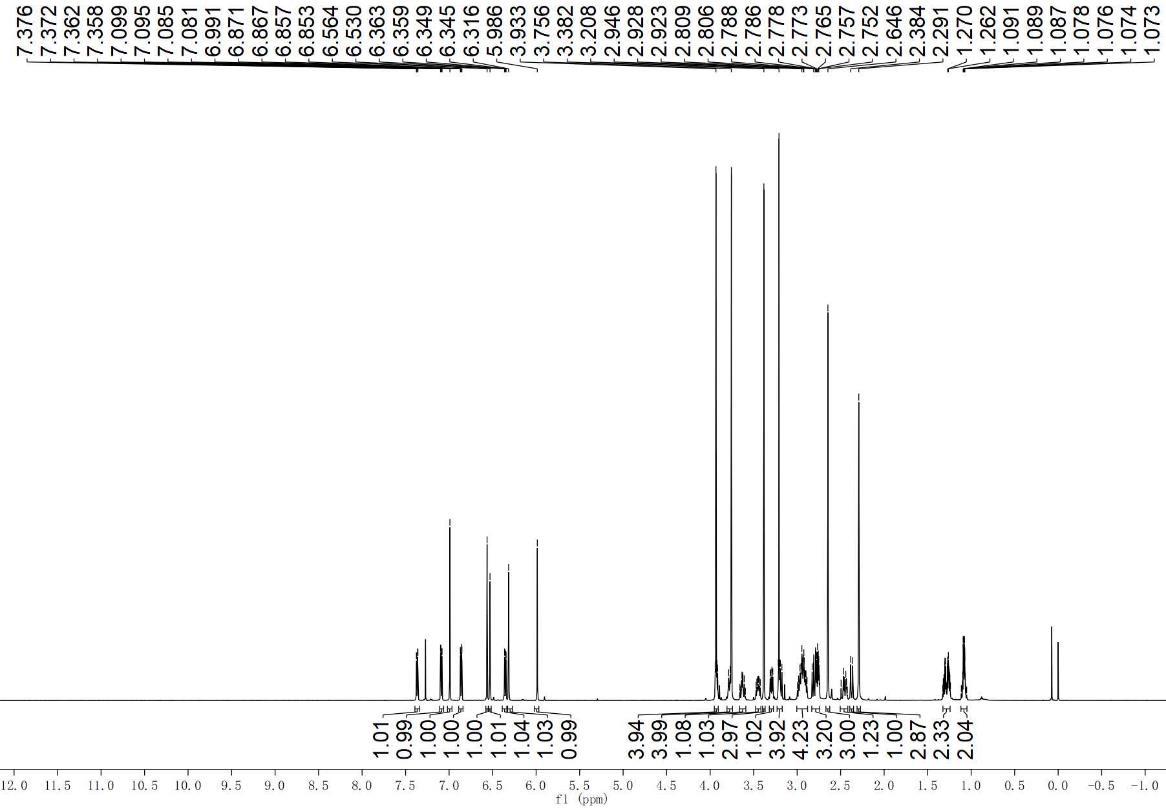


**Compound 39. 14-*O*-(cyclopropanesulfonyl)-tetrandrine: ^13^C-NMR spectrum**


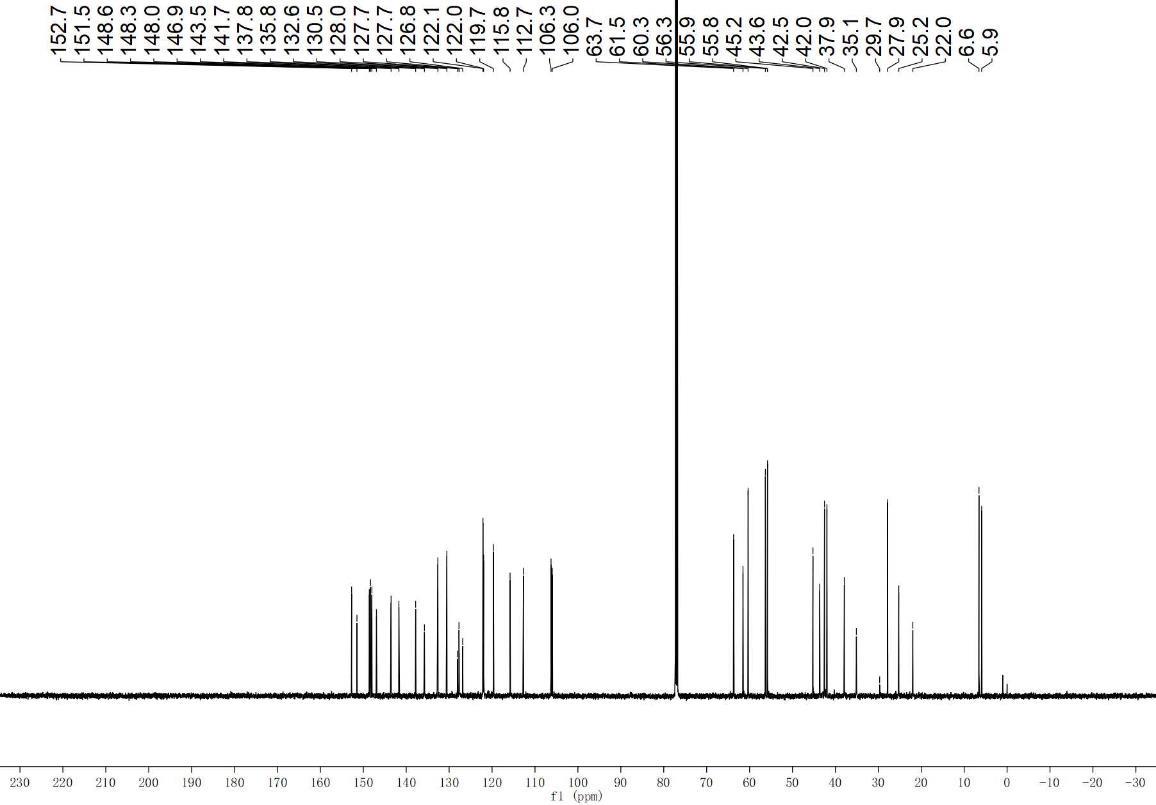


**Compound 39. 14-*O*-(cyclopropanesulfonyl)-tetrandrine: HR-ESIMS spectrum**

**Compound 40.** **14-*O*-(2-phthalimidoethanesulfonyl)-tetrandrine: ^1^H-NMR spectrum**


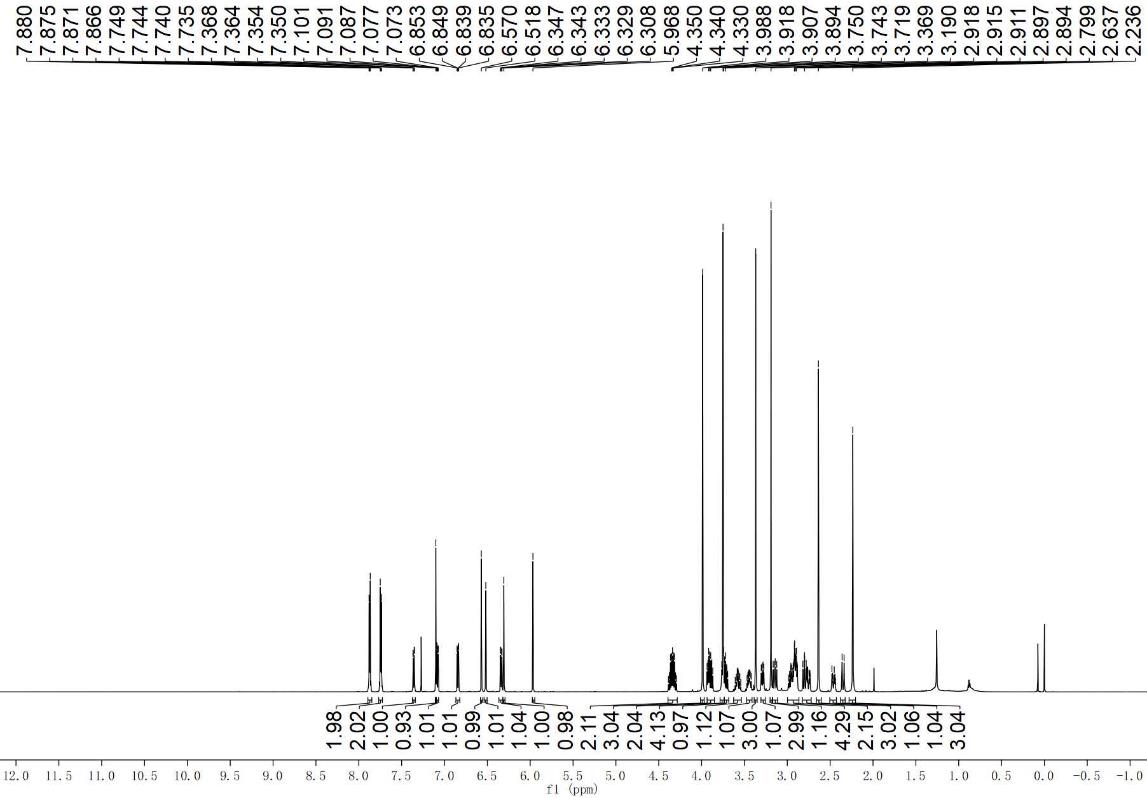


**Compound 40. 14-*O*-(2-phthalimidoethanesulfonyl)-tetrandrine: ^13^C-NMR spectrum**


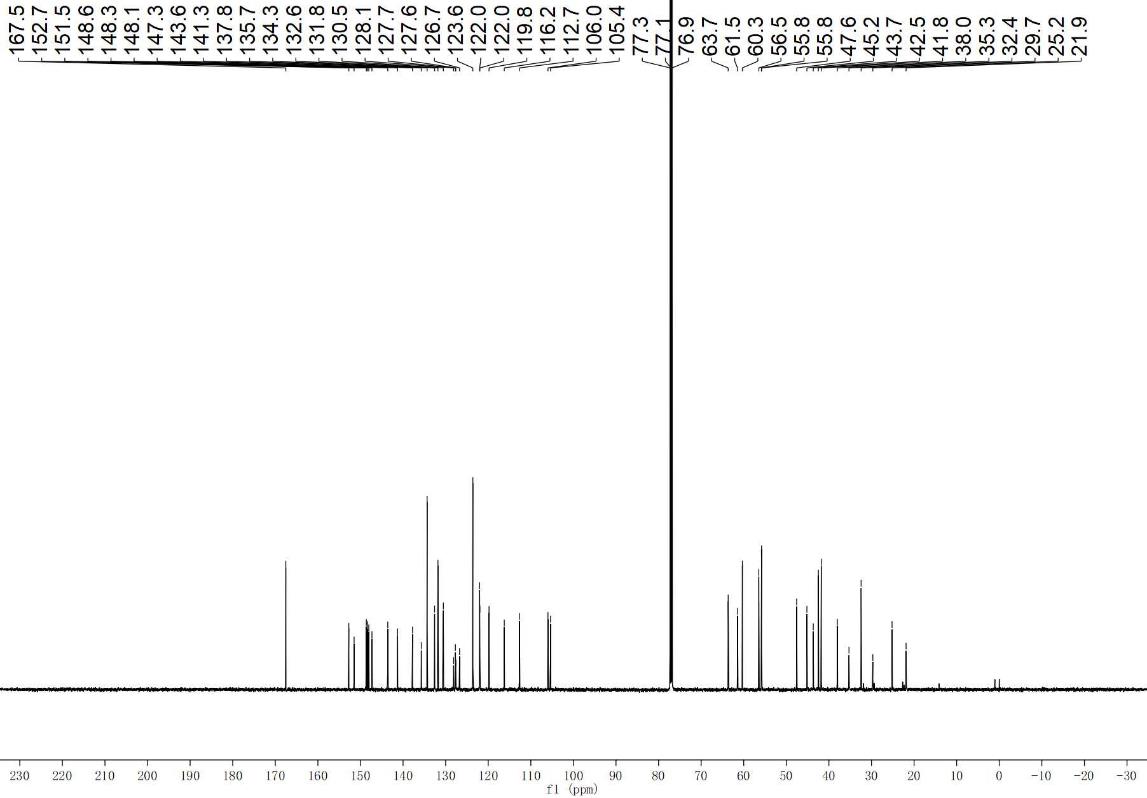


**Compound 40. 14-*O*-(2-phthalimidoethanesulfonyl)-tetrandrine: HR-ESIMS spectrum**
